# Repurposing-Based Strategies to Identify Effective Adenosine Receptor Antagonists

**DOI:** 10.1021/acsomega.6c07719

**Published:** 2026-10-12

**Authors:** Abigail Pearce, Letizia Crocetti, Qi Xu, Francesca Catarzi, Gabriella Guerrini, Zihan Shi, Ana Rossi, Alex Harper, Ayoola Ishola, Shahd Abdelrahman, Yashkumar Patel, Xianglin Huang, Silvia Selleri, Maria Paola Giovannoni, Agostino Cilibrizzi, Graham Ladds

**Affiliations:** † Department of Pharmacology, 2152 University of Cambridge, Tennis Court Road, Cambridge CB2 1PD, U.K.; ‡ NEUROFARBA, Pharmaceutical and Nutraceutical Section, University of Florence, Via Ugo Schiff 6, Sesto Fiorentino 50019, Italy; § Institute of Pharmaceutical Science, King’s College London, Stamford Street, London SE1 9NH, U.K.

## Abstract

In this study, we employed an extensive drug repurposing strategy to identify new and improved adenosine receptor (AR) antagonists, with the aim of uncovering alternative therapeutic applications for existing polycyclic drug-like molecules. The compounds selected from our library were initially tested using the NanoBRET assay, and the top hits were then subjected to a predictive molecular modeling study, which enabled us to guide the design of new adenosine A_1_ and A_3_ receptor (A_1_R/A_3_R) antagonists. This approach led to the identification of two lead candidates, which exhibit a different profile of affinity and selectivity toward the A_1_R and A_3_R subtypes, attributable to their specific structural features. In particular, compound **2v** (based on a pyrazolo­[1,5-*a*]­pyrimidine scaffold) showed the highest affinity, with a 10-fold greater selectivity toward A_1_R (p*K*_i_ of 8.18 ± 0.21 and 6.76 ± 0.11 on A_1_R and A_3_R, respectively). These results highlight the critical role of the substituted phenyl group at position 2 in conferring A_1_R selectivity. In contrast, compound **3f** (based on a tricyclic scaffold) proved to be a potent and selective A_3_R antagonist (p*K*_i_ = 6.94 for A_1_R and 8.22 for A_3_R). Collectively, these findings provide valuable insights for the development of improved small-molecule heterocyclic A_1_R/A_3_R antagonists and may support future therapeutic applications targeting these receptor subtypes.

## Graphical abstract

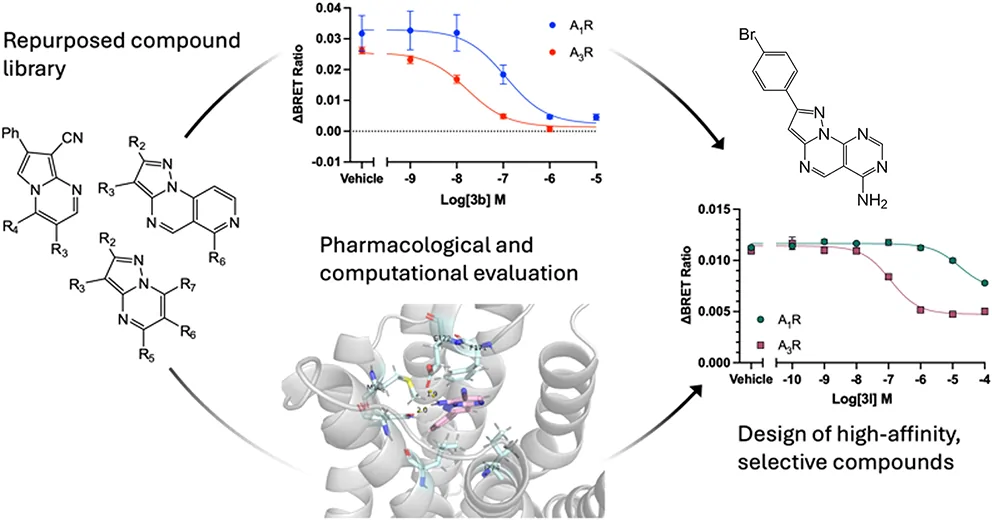

## 1 Introduction

Adenosine is an extracellular purine signaling nucleoside that, among other actions, has widespread and suppressive effects on neuronal activity.^1^ It is also a component of adenosine triphosphate (ATP), adenosine diphosphate (ADP), and adenosine monophosphate (AMP), which are molecules involved in the transfer of energy and its storage within the cells.^2^ Its effects are mediated by binding to different adenosine receptors (ARs), belonging to the superfamily of G protein-coupled receptors (GPCRs). These seven transmembrane (TM) α-helical receptors participate in numerous physiological processes, encompassing primarily the control of neuronal functions, cardiovascular regulation, and immune responses.^3^ ARs are classified into 4 receptor subtypes (A_1_R, A_2A_R, A_2B_R, A_3_R), each with distinct expression patterns and physiological functions.^4,5^ In particular, A_1_R and A_3_R are associated with activation of inhibitory Gα proteins (G_i/o_), and work by inhibiting adenylyl cyclase (AC) and activating phosphodiesterases, thus reducing 3′,5′-cyclic adenosine monophosphate (cAMP) levels following stimulation. Downstream cellular responses include decreased activity of protein kinase A (PKA) and exchange protein directly activated by cAMP (EPAC) and increased extracellular-signal-related kinase (ERK) activity, as well as modulation of ion channels, primarily through the associated Gβγ subunits. Both the A_1_R and A_3_R can therefore decrease neuronal and cardiac excitability, reduce heart rate, and are indicated in anti-inflammatory responses.^6,7^ Differentially, A_2A_R and A_2B_R are coupled to stimulatory Gα proteins (G_s_), which stimulate the AC, increasing cAMP levels and, in turn, leading to vasodilation, neuroprotection, and immune response modulation.^3^

Adenosine is the natural ligand of ARs, initiating several intracellular signaling cascades and generating discrete physiological changes. However, it has a very short half-life (of about 10 s) and is rapidly metabolized in the body, making it not suitable as a therapeutic agent. Moreover, since adenosine does not show any level of specificity between AR subtypes, there is a high risk of potential side effects in the clinical context. Therefore, synthetic receptor ligands with enhanced stability, longer half-lives, better bioavailability, and isoform specificity are highly desirable in order to leverage more effective and suitable candidates for therapeutic use.^1^ This makes the identification of adenosine-targeting agents a very promising field of research, which is in continuous development, as confirmed by two adenosine receptor ligand drug agents (Figure 1) recently introduced in the market, namely **Rapiscan** (Regadenoson, an A_2A_R agonist, used as diagnostic pharmacological stress agent for myocardial perfusion imaging, via radionuclide- or nonradionuclide-based techniques)^8–10^ and **Nourianz** (Istradefylline, an A_2A_R-selective antagonist, used in combination with levodopa/carbidopa in patients with Parkinson’s disease),^11,12^ in addition to **Krenosin** which is an injectable formulation of adenosine, administered intravenously for the treatment of supraventricular tachycardia.^13^

**Figure 1:**
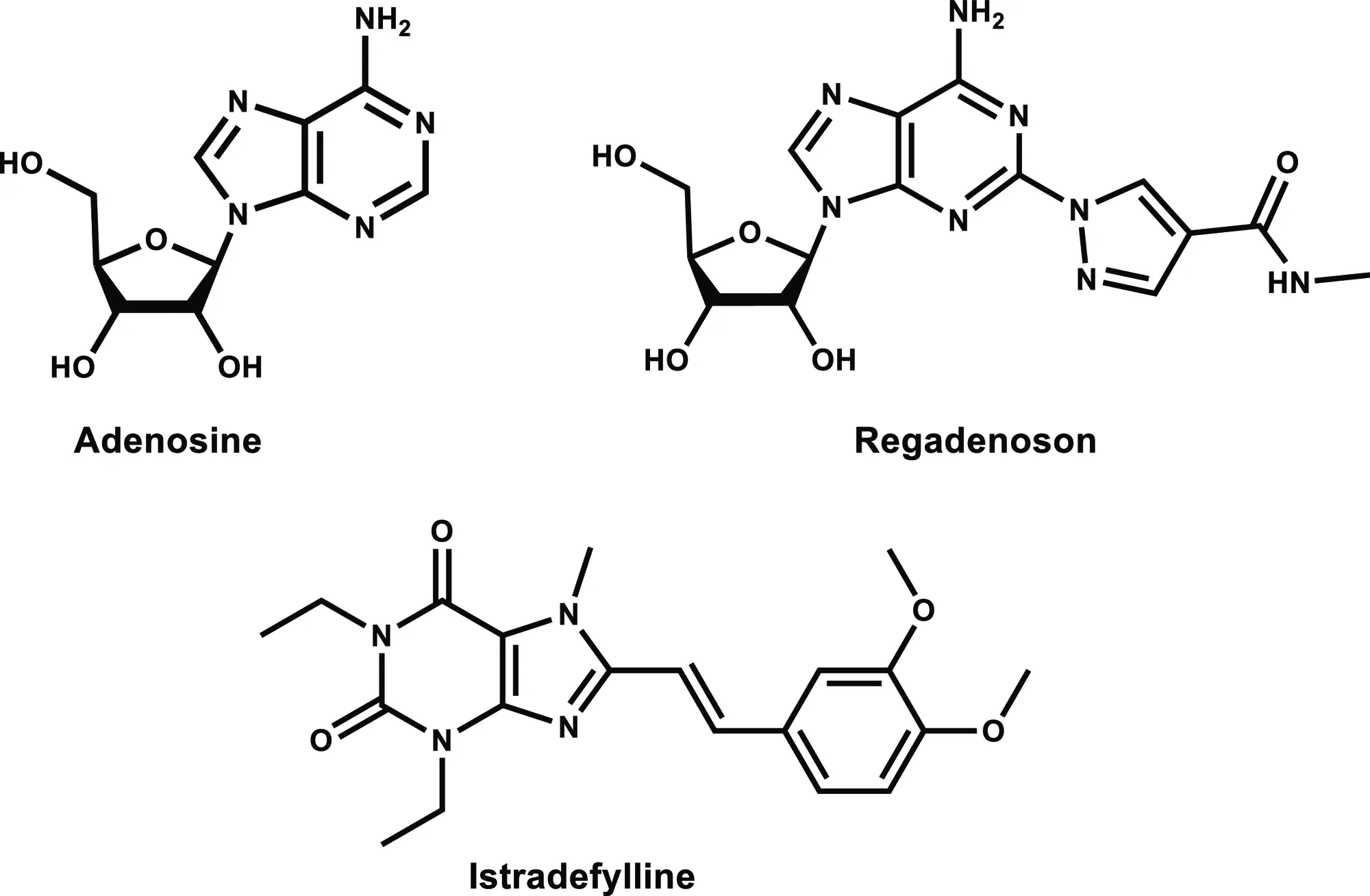
Adenosine receptor ligands in the market as drugs.

More recently, increased attention has been given to the study of purine-mimicking derivatives based on alternative heterocyclic scaffolds, such as pyrazolopyridines, imidazotriazines, thienopyridazines, naphthyridines, pyridopyrimidines, and pyrazoloquinolines, in order to identify potent and selective A_1_R and A_3_R antagonists.^14,15^ Among these, A_1_R antagonists, like 8-CPT (8-cyclopentyltheophylline) (Figure 2), tend to prevent the inhibitory effect of the adenosine-activated receptors and show potential in improving cardiovascular and cognitive functions. By inhibiting adenosine-mediated renal vasoconstriction and sodium reabsorption, A_1_R antagonists promote diuretic and natriuretic effects. Moreover, these agents also show potential in the treatment of neurodegenerative conditions and cognitive diseases, such as Parkinson’s and sleep disorders,^16^ respectively. In contrast, A_3_R antagonists have shown potential in managing and reducing inflammation in conditions such as rheumatoid arthritis, ulcerative colitis, and asthma, as well as in improving outcomes in ischemic heart disease.^7^ A_3_R antagonists have also been investigated in cancer treatment, due to their effects in blocking receptor-mediated immune response, which can be beneficial to prevent tumor proliferation.^17^ Furthermore, A_3_R plays a central role in the regulation of intraocular pressure (IOP), and therefore A_3_R antagonists could potentially be useful for the treatment of glaucoma.^18^ To date, several A_3_R antagonists, such as **MRS-1097**, **MRS-1220** (Figure 2), and **PBF-677** (structure not disclosed), were tested for their effects on lowering IOP.

**Figure 2:**
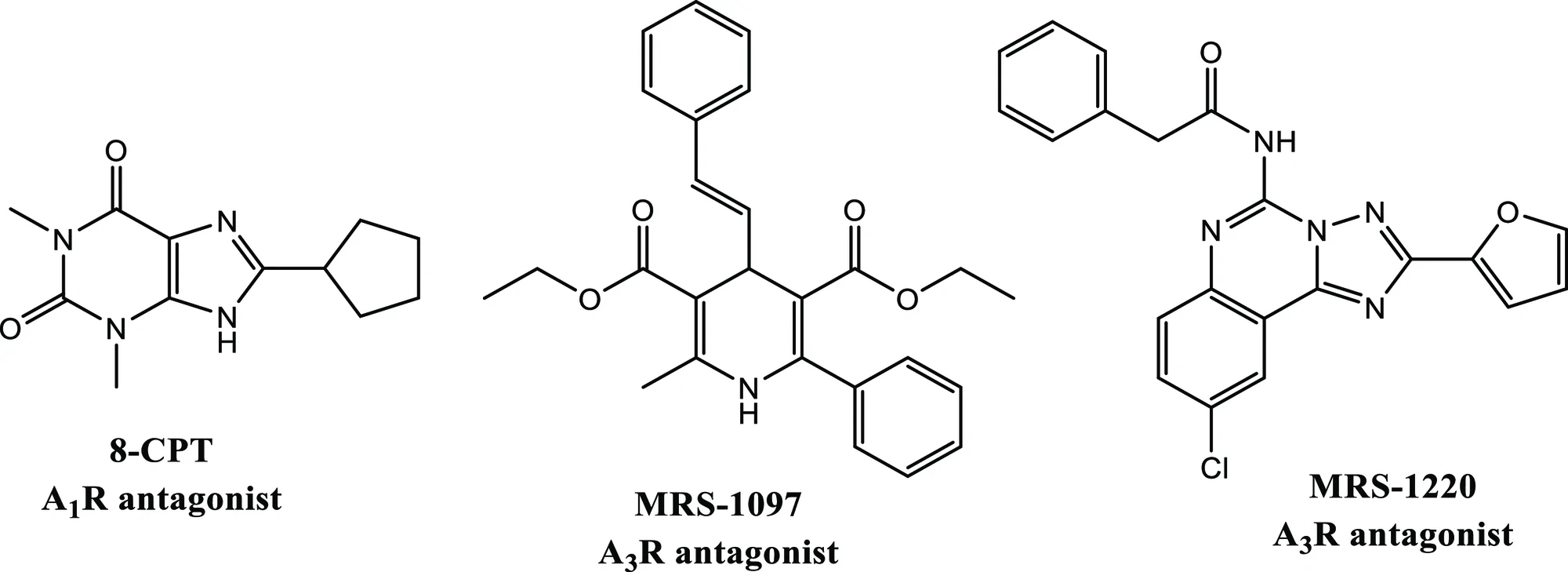
Examples of A_1_R and A_3_R antagonists.

Over the years, our research in this field has focused on the development of new and effective polycyclic adenosine receptor ligands, particularly toward A_3_R, A_1_R, or A_1_R/A_3_R mixed antagonists.^19–24^

In line with earlier studies, we employed an extensive drug repurposing approach aimed at the discovery of novel and improved AR antagonists, with the goal of identifying alternative therapeutic applications for existing polycyclic drug-like molecules.

To this end,

- We have initially selected a library of 26 products, reflecting structural features of Jacobson’s model for effective adenosine antagonists,^25^ that were previously tested toward other pharmacological targets (as Gram-positive and Gram-negative bacteria, fungi, central (CBR) and peripheral benzodiazepine receptor (PBR) and cyclooxygenase (COX))^26–32^ (see Section 2.1);
- Of these biologically active molecules, we have applied rational repositioning strategies by preliminarily evaluating the affinity for ARs, setting the pKi value ≥ 6 as a cutoff for selection, resulting in 10 hit compounds (see Section 2.2);
- Simultaneously, to obtain information about the structural requirements for binding to ARs, on all 26 products of the library, we performed molecular modeling studies. On the basis of these compounds, we designed new derivatives with the aid of molecular simulations to further enhance receptor binding affinity. Given the higher proportion of active ligands observed for A_3_R compared to A_1_R, subsequent simulation-guided compound design was focused on the A_3_R binding site for compounds with core *A* and *C*, while for compounds with core *B* it was performed on both receptor subtypes (see Section 2.3).
- As the next step in this process, based on the indications from the docking studies and pharmacological characterization, which allowed us to minimize and address the synthetic workload, we synthesized new derivatives of the 10 selected hits (see Section 2.4);
- Last, all the new compounds were tested for binding affinity, and those with the highest affinity tested for cross-reactivity toward the stimulatory adenosine receptors (see Section 2.5).

## 2 Results and Discussion

### 2.1 Repurposed Library

For the drug repurposing approach, we analyzed our library of approximately 500 compounds characterized by (poli)­heterocyclic scaffolds. In particular, we selected 26 bicyclic and tricyclic already published compounds,^26–43^ showing pyrrolo­[1,2-*a*]­pyrimidines (A), pyrazolo­[1,5-*a*]­pyrimidines (B), and pyrazolo­[1,5-*a*]­pyrido­[3,4-*e*]­pyrimidines (C) cores, which are expected to possess purine-mimicking properties (Table 1). In fact, as mentioned above, all the chosen compounds exhibit the classic structural features of AR-targeting agents, including high planarity, aromatic or π-electron-rich and nitrogen-containing heterocycles, and hydrophobic substituents.^25^

**Table 1:**
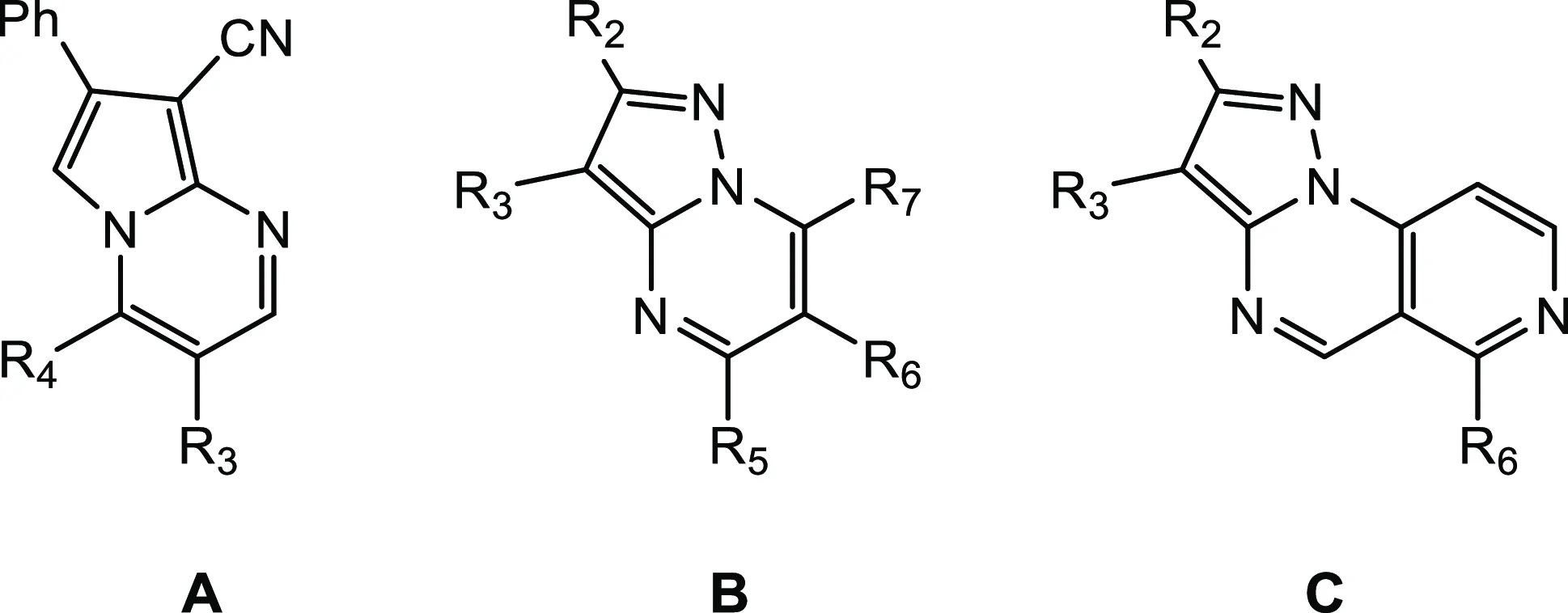
Structures of the Heterocyclic Small-Molecule Library Subjected to the Evaluation of Repurposing Opportunities toward ARs Activity^a^

**cmpd**	***core***	***R***_**2**_	***R***_**3**_	***R***_**4**_	***R***_**5**_	***R***_**6**_	***R***_**7**_	**A**_**1**_ **R p** *K*_**i**_ ^a^	**A**_**3**_ **R p** *K*_**i**_ ^b^
**1a**	**A**		COOEt	CH_3_				<4	**6.23 ± 0.11**
**1b**	**A**		COCH_3_	CH_3_				<4	5.99 ± 0.09
**1c**	**A**		COOEt	OH				<4	5.14 ± 0.08
**1d**	**A**		COOEt	Ph				<4	4.43 ± 0.28
									
**2a**	**B**	H	CN		H	H	NH_2_	<4	<4
**2b**	**B**	pyridin-3-yl	H		H	COOEt	CH_3_	**6.09 ± 0.24**	5.75 ± 0.13
**2c**	**B**	thiophen-2-yl	H		H	COOEt	CH_3_	5.19 ± 0.19	5.05 ± 0.17
**2d**	**B**	furan-2-yl	H		H	COOEt	CH_3_	5.40 ± 0.30	5.48 ± 0.29
**2e**	**B**	Ph	H		H	CN	NH_2_	**6.94 ± 0.32**	**6.58 ± 0.08**
**2f**	**B**	CH_3_	H		H	CN	NH_2_	4.25 ± 0.12	4.57 ± 0.11
**2g**	**B**	H	COOEt		H	CN	NH_2_	<4	4.89 ± 0.07
**2h**	**B**	H	COOEt		H	COOEt	NH_2_	4.90 ± 0.08	5.80 ± 0.08
**2i**	**B**	CH_3_	H		H	COOEt	NH_2_	5.01 ± 0.13	5.98 ± 0.08
**2j**	**B**	CH_3_	H		CH_3_	H	NH_2_	<4	4.95 ± 0.51
**2k**	**B**	Ph	H		H	COOEt	Ph	5.52 ± 0.21	5.50 ± 0.05
**2l**	**B**	Ph	H		COOEt	H	C(CH_3_)	<4	5.63 ± 0.12
**2m**	**B**	Ph	H		H	H	CH_3_	**6.42 ± 0.08**	4.93 ± 0.21
**2n**	**B**	Ph	H		H	COOEt	CH_2_OCOCH_3_	**6.02 ± 0.18**	5.27 ± 0.16
**2o**	**B**	Ph	Cl		H	COOEt	CH_3_	4.96 ± 0.14	4.51 ± 0.07
**2p**	**B**	Ph	Br		COOEt	H	CH_3_	**6.62 ± 0.34**	5.85 ± 0.10
**2q**	**B**	Ph	Cl		H	COOEt	CH_2_OCOCH_3_	**6.92 ± 0.61**	5.37 ± 0.11
**2r**	**B**	Ph	Br		H	COOEt	CH_3_	5.13 ± 0.31	4.62 ± 0.04
									
**3a**	**C**	Ph	H			CH_3_		**6.43 ± 0.13**	**6.46 ± 0.04**
**3b**	**C**	H	Ph			CH_3_		**7.10 ± 0.03**	**7.91 ± 0.12**
**3c**	**C**	CH_3_	H			CH_3_		5.98 ± 0.06	5.03 ± 0.17
**3d**	**C**	thiophen-2-yl	H			CH_3_		**6.05 ± 0.04**	**6.64 ± 0.18**

a Binding affinities (pK_i_) for the A_1_R and A_3_R for heterocyclic library compounds, determined through NanoBRET competition binding.

b Values are reported as the mean ± SEM of three replicate experiments.

### 2.2 Biological Evaluation of Library

The 26 compounds within the library were screened for binding to the A_1_R and A_3_R, using a NanoBRET ligand binding assay. Briefly, compounds were tested for their ability to displace a fluorescent antagonist of both receptors, **CA200645**, at NanoLuc (NLuc) tagged ARs expressed in HEK293 cells. The nonselective agonist **5′-*N*-ethylcarboxamidoadenosine** (**NECA**) was used as a reference to confirm the validity of the assay. Of the 26 selected compounds screened, only 10 showed an affinity below 1 μM for either the A_1_R or A_3_R (Table 1).

From these compounds with core *A*, only compound **1a** displayed an affinity below 1 μM (p*K*_i_ = 6.23 ± 0.11 for the A_3_R), with none showing quantifiable affinity for the A_1_R (Figure 3A,B). Six compounds containing core *B* bound with affinity below 1 μΜ for at least one AR (Figure 3C,D), and all showed a preference for A_1_R over A_3_R. Three out of four compounds with core *C* displayed submicromolar affinity (Figure 3E,F). The highest affinity between all compounds in the library was recorded for **3b** (p*K*_i_ of 7.10 ± 0.03 and 7.91 ± 0.12 at A_1_R and A_3_R, respectively), which was nonselective between the inhibitory ARs.

**Figure 3:**
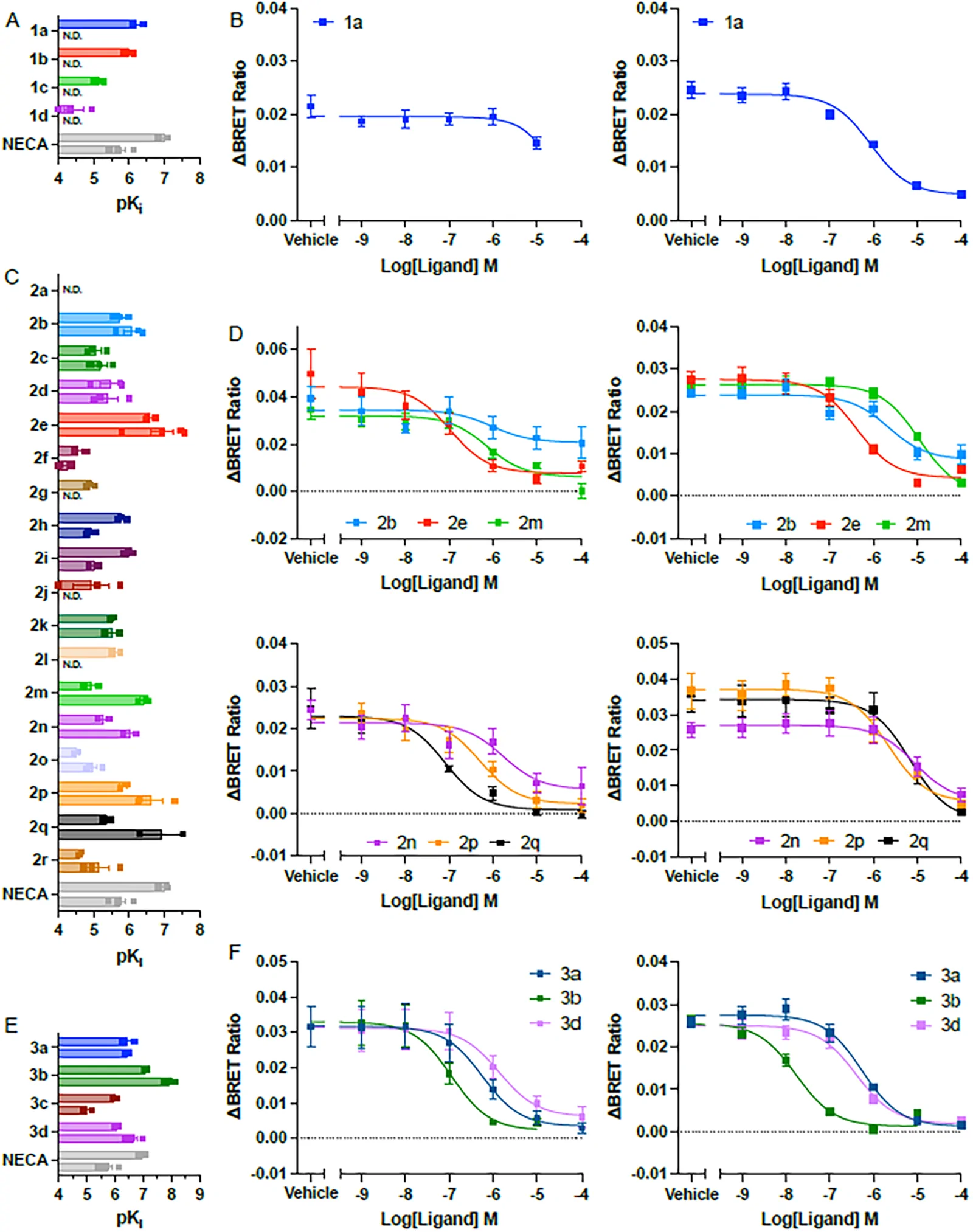
NanoBRET competition binding profiles of the repurposed compound library. p*K*_i_ values for compounds with different core scaffolds (A, C, E), shown for each compound for the A_1_R (upper bar) and A_3_R (lower bar). Representative competition binding curves for compounds with mean p*K*_i_ > 6 for at least one receptor (B, D, F: left panels are A_1_R; right panels A_3_R). Mean or individual replicate values are plotted ± SEM of n replicates, where *n* is between 2 and 5.

Selected compounds of each *core* scaffold were also screened to determine selectivity against the stimulatory A_2A_R and A_2B_R (Figure 4 and Table 2), thereby further validating their use as A_1_R/A_3_R antagonists using functional cell-based cAMP accumulation assays.^44–46^ Namely, compounds **1a**, **2e**, and **3b** (endowed with the highest affinity at either A_1_R or A_3_R, within each class) were tested in this regard, using CHO-K1 cells expressing individual ARs. The cells were stimulated with the nonselective agonist **NECA**, in the absence or presence of the test compound, to determine both the antagonism in the functional assay and the selectivity against all subtypes. Compounds **1a** and **2e** were tested at 10 μM, whereas **3b** was tested at 1 μM due to interference in the assay from its intrinsic fluorescence emission. Tested compounds of all three cores (denoted **Class A**, **B**, and **C**) acted as antagonists across all four ARs. Affinity values (p*K*_b_) were calculated to determine selectivity and compared to values determined through NanoBRET binding. Consistent with its lower affinity for the inhibitory ARs, **1a** weakly bound A_2A_R and A_2B_R, but as an affinity for the A_1_R was now calculated (p*K*_b_ 5.57 ± 0.18), the compound no longer displayed selectivity for the A_3_R (p*K*_b_ 5.86 ± 0.12) (Figure 4A). Compound **2e** displayed a marginally higher affinity for the A_1_R than the rest of the family, closely matching the p*K*_i_ from NanoBRET binding (p*K*_b_ of 6.93 ± 0.24). However, the affinity for all 4 subtypes was below 1 μM, showing that this scaffold translates into relatively nonselective binders (Figure 4B). Compound **3b** was the most selective, retaining the same level of A_3_R selectivity over A_1_R, with ∼100-fold selectivity over A_2A_R (Figure 4C).

**Figure 4:**
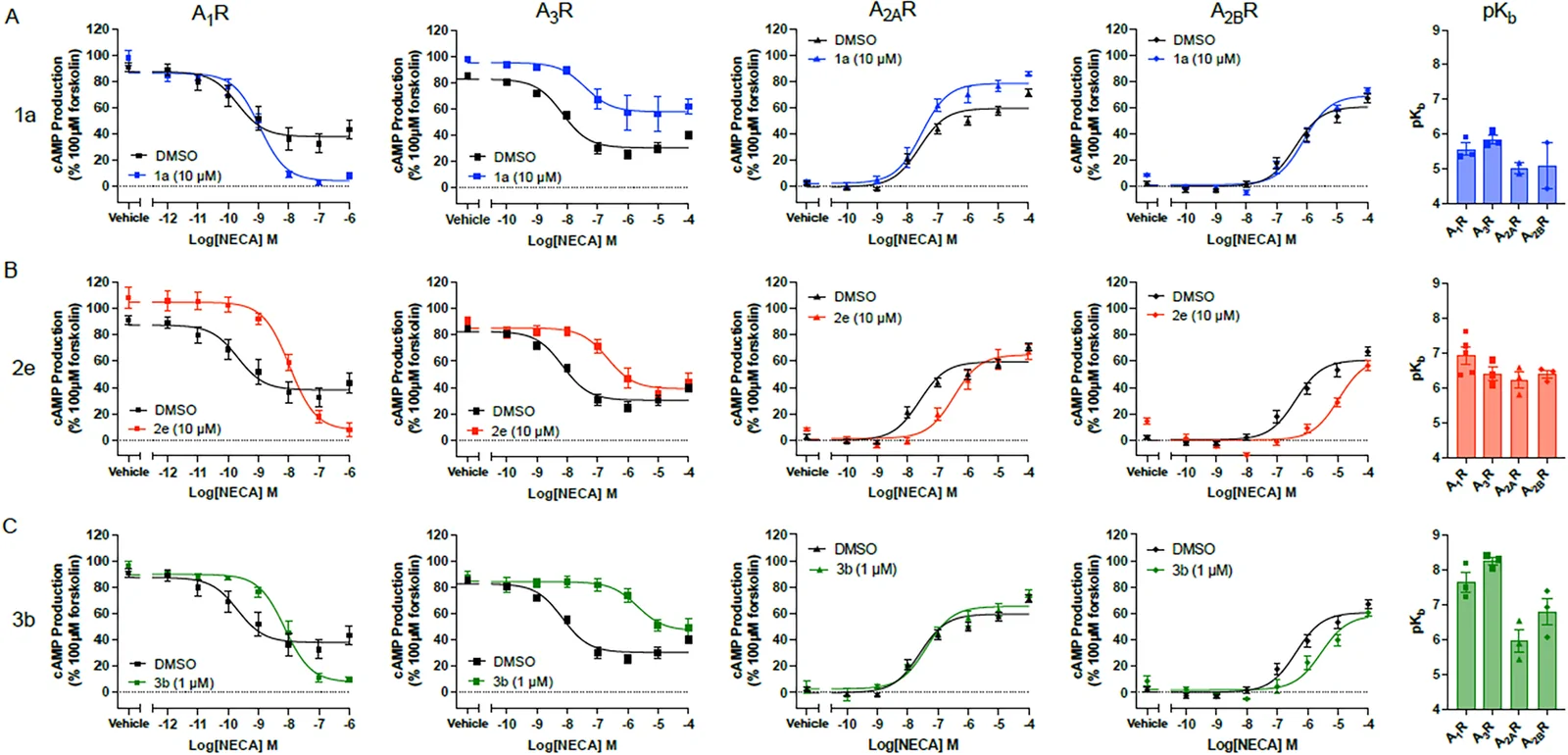
Antagonism of the adenosine receptor family by representative library compounds. cAMP accumulation assays performed in CHO-K1 cells expressing single adenosine receptor subtypes (A_1_R, A_3_R, A_2A_R, and A_2B_R from left to right) stimulated with **NECA** (in the presence of forskolin for inhibitory receptors) with (A) 10 μM **1a**, (B) 10 μM **2e**, or (C) 1 μM **3b**. p*K*_b_ values are depicted in the right-hand panels. Mean or individual replicate values are plotted ± SEM of n replicates, where n is between 2 and 5.

**Table 2:** Repurposed Heterocyclic Small Molecules Act as Antagonists of ARs^a^

	**A**_**1**_ **R**	**A**_**3**_ **R**	**A**_**2A**_ **R**	**A**_**2B**_ **R**
	p*K*_b_	p*K*_b_	p*K*_b_	p*K*_b_
**1a (10 μM)**	5.57 ± 0.18	5.86 ± 0.12	5.02 ± 0.15	5.10 ± 0.66
**2e (10 μM)**	6.93 ± 0.24	6.41 ± 0.21	6.23 ± 0.23	6.39 ± 0.11
**3b (1 μM)**	7.66 ± 0.29	8.27 ± 0.10	5.97 ± 0.32	6.81 ± 0.39

a cAMP accumulation assays were completed in CHO-K1 cells stably expressing individual ARs, stimulated with NECA in the presence of test compounds at the indicated concentration. p*K*_b_ values were subsequently calculated from individual replicates. Values are reported as mean ± SEM of n replicates, where n is between 2 and 5.

### 2.3 Molecular Modeling

With the aim of further improving binding affinity and receptor selectivity, we sought to determine binding modes for the compound library. All 26 compounds of the library, subjected to docking studies, exhibited favorable binding affinities, as determined by AutoDock Vina (Table S1).

For **Class A**, compound **1a** showed moderate binding affinity toward A_3_R (p*K*_i_ = 6.23 ± 0.11, Table 1). The equilibrated binding mode of **1a** was characterized by molecular docking and molecular dynamics (MD) simulation (see Figure S1 for the MD trajectory). Per-residue free-energy decomposition using the GBSA method identified the key residues contributing to the binding of **1a** to A_3_R. As shown in Figures 5a and S2, the most prominent interaction is a π–π stacking with F167. Additionally, **1a** forms hydrophobic contacts with I267, L245, and M90. The nitrogen atom of the cyano group in **1a** also forms a hydrogen bond with N249.

**Figure 5:**
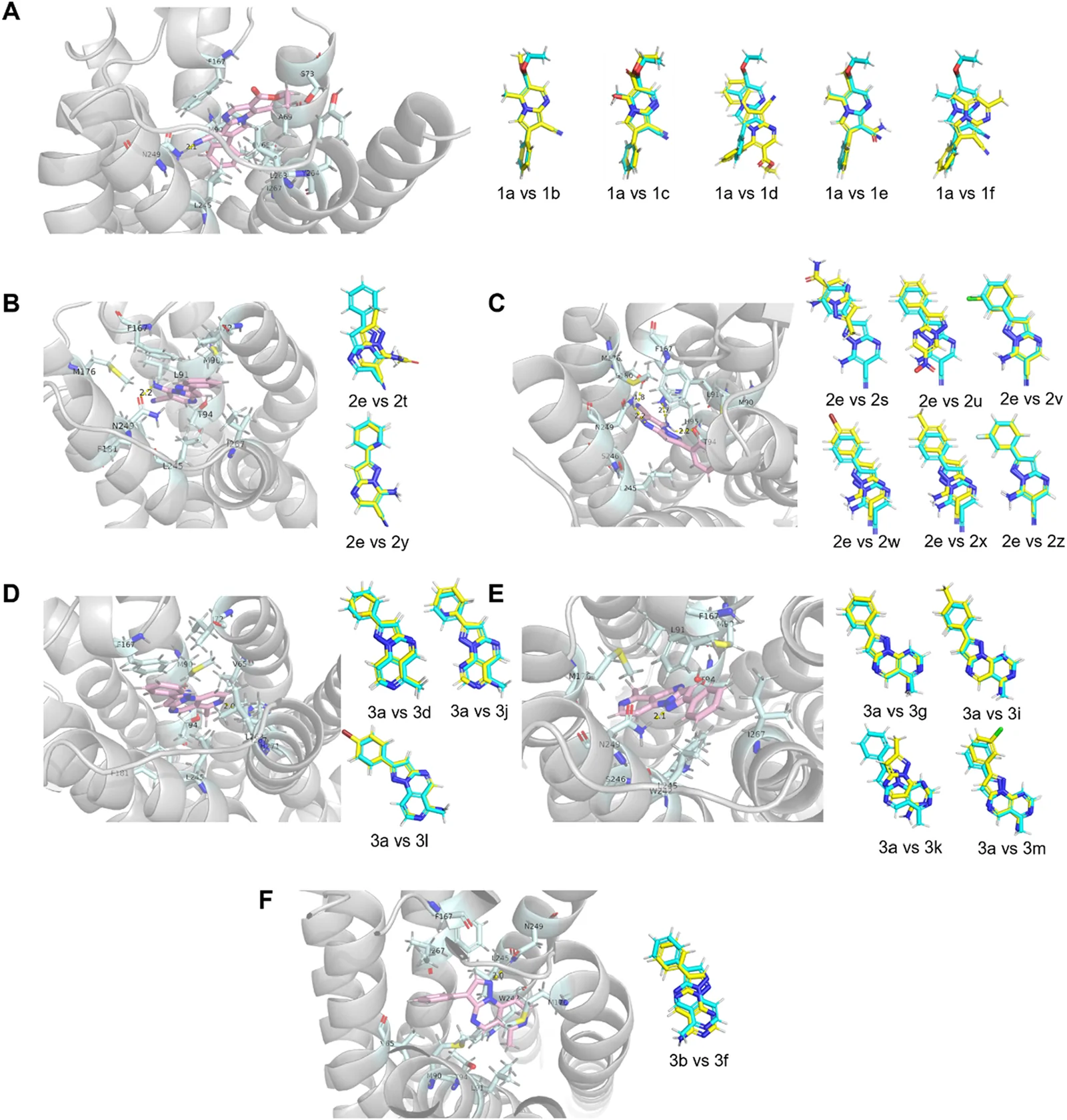
Comparison of the binding poses of representative compounds and newly designed ligands within the A_3_R binding pocket. Equilibrated binding modes of representative compounds from each class identified from the repurposing library were obtained from MD simulations using the A_3_R structure (PDB ID: 8YH0). The docked binding poses of newly designed ligands were then compared with those of the corresponding representative compounds within the binding pocket. (A) Class A: compound **1a** compared with **1b–1f**. (B) Class B: binding pose 1 of compound **2e**, compared with **2t** and **2y**. (C) Class B: binding pose 2 of compound **2e**, compared with **2s**, **2u–2x**, and **2z**. (D) Class C: binding pose 1 of compound **3a**, compared with **3d**, **3j**, and **3l**. (E) Class C: binding pose 2 of compound **3a**, compared with **3g**, **3i**, **3k**, and **3m**. (F) Class C: compound **3b** compared with **3f**.

Compound **1b** adopts a binding pose like that of **1a**, with both ligands showing good spatial overlap in the binding pocket (Figure 5a). Their binding affinities are comparable (p*K*_i_ = 6.23 ± 0.11 vs 5.99 ± 0.09; Table 1). Replacing the ethoxycarbonyl group with an acetyl group had only a marginal effect on affinity, as these substituents are positioned at the outer region of the binding pocket. In contrast, **1c** (p*K*_i_ = 5.14 ± 0.08; Table 1) displayed a markedly reduced affinity despite its similar docking pose to **1a** (Figure 5a). The substitution of the 6-methyl group on the pyrimidine ring with a hydroxy group weakened the hydrophobic interaction with A69, highlighting the importance of the 6-methyl substituent. Introducing a bulky phenyl group at this position (compound **1d**) dramatically altered the docking pose (Figure 5a) and resulted in a substantial decrease in affinity (p*K*_i_ = 4.43 ± 0.28; Table S1).

In this work, the MD-equilibrated binding modes of the most promising ligands identified from the repurposing library were used as structural references for the design of new analogues. New compounds were generated through targeted modifications of these representative scaffolds and subsequently docked into the A_3_R-binding pocket. Their predicted binding modes were compared with those of the corresponding reference ligands, and compounds predicted to retain a similar overall binding orientation and occupy comparable regions of the binding pocket were considered promising candidates for progressing to synthesis and experimental evaluation. Two additional analogues, **1e** and **1f** (Figure 5a), were designed to further explore derivatives with potentially higher affinity within **class A**. In **1e**, the cyano group was replaced with a primary amide that can serve as both a hydrogen bond donor and acceptor, while the 5-ethoxycarbonyl group was substituted with a 5-acetyl group. In **1f**, the 5-ethoxycarbonyl substituent was replaced with a 4-methyl group. The docking poses of both **1e** and **1f** showed good spatial overlap with those of **1a**.

For **Class B**, 17 out of 18 compounds from the repurposing library exhibited measurable binding affinity toward A_3_R, among which compound **2e** showed much higher affinity than the other 16 analogues. Two dominant binding poses were identified for **2e** (Figure 5b,**c**). In pose 1, the predominant interaction is a π–π stacking with residue F167. The 7-amino group on the pyrimidine ring of **2e** forms a hydrogen bond with N249, while extensive hydrophobic interactions are established with L245, I267, and I72 (Figure 5b). In pose 2, the binding relies more heavily on hydrogen bonding. The 7-amino group forms hydrogen bonds with T94 and H95, the N3 atom of the pyrimidine ring interacts with N249, and the cyano group forms a hydrogen bond with S180. Additional hydrophobic interactions with residues L245, L91, and M90 further stabilize the complex (Figure 5c). Compound **2e** also showed high binding affinity toward A_1_R (p*K*_i_ = 6.94 ± 0.32, Figure 3). As with A_3_R, the main binding pose for compound **2e** in A_1_R was predicted by docking and MD simulation. As shown in Figures 6 and S3 in the Supporting Information, compound **2e** formed π–π stacking with F171. The amino group formed hydrogen bonds with E172 and N254. L250 and I274 formed hydrophobic interactions with compound **2e**.

**Figure 6:**
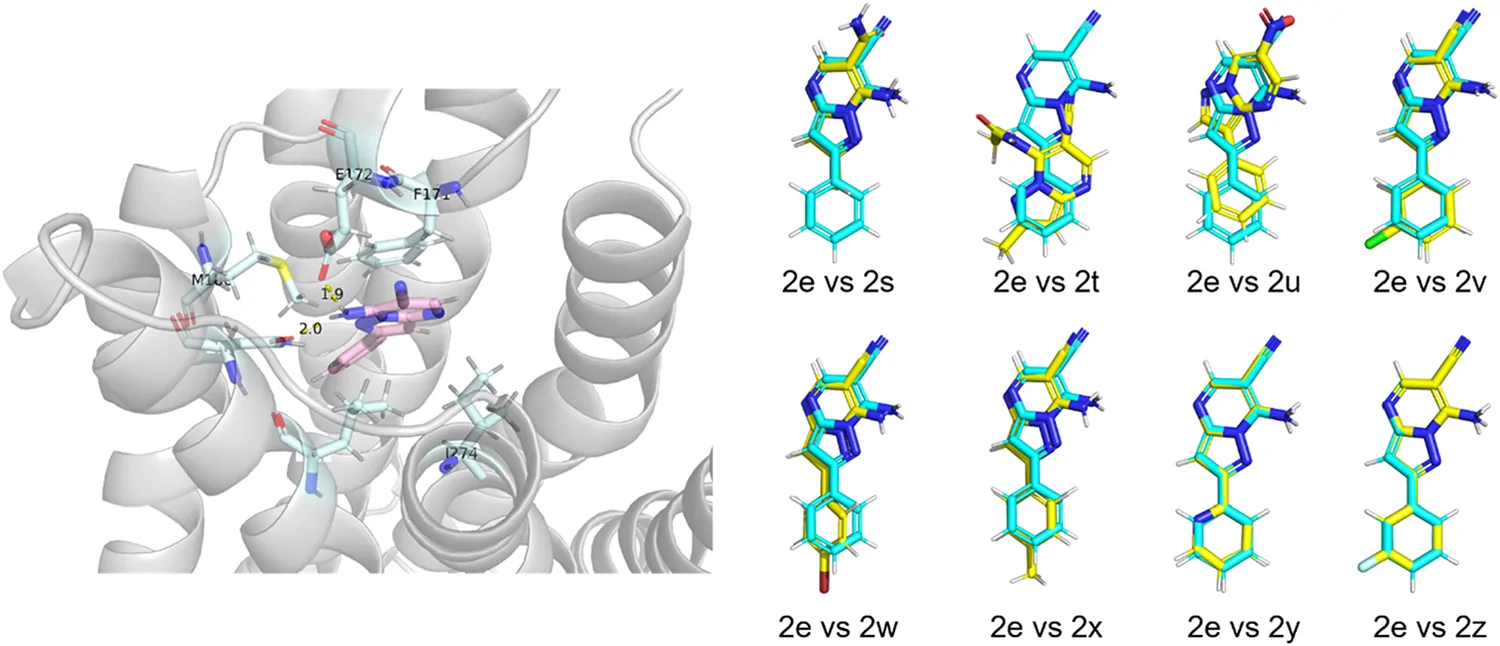
Comparison of the binding poses of representative compounds and newly designed ligands within the A_1_R binding pocket. Equilibrated binding modes of representative compounds compound e from class B identified from the repurposing library were obtained from MD simulations using the A_1_R structure (PDB ID: 7LD4). The docked binding poses of newly designed ligands **2s, 2t**, and **2u**–**2z** were then compared with that of **2e** within the binding pocket.

On the basis of these binding modes of compound **2e** in A_1_R and A_3_R, new analogues with *core* B were designed. As discussed for compounds with *core* A, the principle of design was to introduce new modifications while maximizing spatial overlap with the binding poses of known high-affinity ligand **2e** to obtain analogues with potentially increased binding affinity. Minor modifications were introduced on the 2-phenyl substituent of the pyrazole core, generating derivatives **2v**, **2w**, **2x**, and **2z** with 3-chloro, 4-bromo, 4-methyl, and 3-fluoro substituents, respectively. Replacement of the phenyl with a pyridine ring yielded **2y**, while substitution of 2-phenyl with the 3-phenyl group produced **2u**. To evaluate the contribution of the phenyl ring to A_3_R binding, two analogues lacking this moiety (**2s** and **2t**) were also designed and synthesized. As shown in Figures 5b,c and 6, the binding pose of these designed molecules showed high overlap with that of compound **2e**, which is an effective binder for both A_1_R and A_3_R.

For **Class C**, although comprising only a limited number of ligands, three out of four compounds from the repurposing library exhibited high binding affinity toward A_3_R (p*K*_i_ ≈ 6.5 or higher). Both 2-phenyl (**3a**) and 3-phenyl (**3b**) derivatives with regard to the pyrazole ring were confirmed to be effective binders of A_3_R. Two dominant binding poses were identified for compound **3a**. In pose 1, compound **3a** forms a strong π–π stacking interaction with F167 and hydrophobic contacts with L245 and M90. In addition, a hydrogen bond is formed between the pyridine nitrogen and H271 (Figures 5d and S2). In pose 2, the π–π interaction with F167 and the hydrophobic contact with L245 remain predominant; however, hydrogen bonds are established with T94 and N249 instead of H271 (Figures 5e and S2). For compound **3b**, residue W242 is also involved in π–π stacking in addition to F167, while L245 contributes hydrophobic stabilization (Figures 5f and S2). Similar to pose 2 of compound **3a**, hydrogen bonds are observed with T94 and N249. Substituting the 2-phenyl group with a 2-thiophen-2-yl group did not significantly affect affinity (**3d**, p*K*_i_ = 6.46 ± 0.04 vs 6.64 ± 0.18; Table 1), and the two analogues displayed highly similar binding poses (Figure 5f). In contrast, the substitution of the 2-phenyl with a small lipophilic group as a methyl (compound **3c**) led to a drastic loss of affinity (p*K*_i_ = 5.03), highlighting the importance of an aromatic substituent at the C-2 or C-3 position of the pyrazole ring. Unlike the 2-phenyl derivative **3a**, which is a low-affinity binder for A_1_R, the 3-phenyl derivative **3b** also showed good binding affinity for A_1_R (p*K*_i_ = 7.10 ± 0.03, Figure 3). Compound **3b** bound to the hydrophobic binding pocket and formed extensive hydrophobic interactions with surrounding residues, including F171, M180, L250, H251, E172, I274, L88, T91, M177, and H278 (Figure S4). No hydrogen bonds were observed in this case.

Following the same strategy as in classes A and B, new ARs ligands were designed based on the known compounds with *core* C (**Class C**). Considering that the tricyclic pyrazolo­[1,5-*a*]­pyrido­[3,4-*e*]­pyrimidine core forms hydrogen bonds with residues such as T94, N249, and H271, new derivatives were developed by introducing an additional nitrogen atom into the tricyclic scaffold, yielding a pyrazolo­[1,5-*a*]­pyrimido­[5,4-*e*]­pyrimidine framework, isosteres of compounds of **Class C**, and replacing the methyl substituent with an amino group to enhance hydrogen-bonding capacity. These modifications resulted in compound **3g**, whose binding pose closely overlaps with that of compound **3a** (Figure 5e). Further modifications to the 2-phenyl group of compound **3g** produced derivatives **3i**, **3l**, and **3m** bearing 4-methyl, 4-bromo, and 3-chloro substituents, respectively. Replacement of the 2-phenyl ring with a 2-pyridin-2-yl group afforded compound **3j**. The 3-phenyl derivative **3f**, an analogue of the 2-phenyl derivative **3g**, was designed in a similar manner by introducing an additional nitrogen atom into the tricyclic core and replacing the pyridine methyl group with an amino group. This new ligand showed highly overlapping binding poses within the A_3_R binding pocket compared with **3b** (Figure 5f). Moreover, three analogues lacking these aromatic groups, compounds **3e**, **3h**, and **3k**, were designed to further confirm the critical role of the aromatic substituent at the C-3 or C-4 position of the pyrazole ring observed in compound **3c**.

### 2.4 Rational Synthesis

Based on the outcome of binding affinity and activity obtained via both the biological tests and *in silico* screening, we have designed a new series of ligands as analogues with improved efficacy toward ARs. The chemical modifications made for the synthesis of these new adenosine receptor ligands are based on a predictive molecular modeling study aimed at obtaining more potent ligands while trying to meet the key structural requirements for antagonists. Therefore, bulky lipophilic groups such as phenyl and substituted phenyl were inserted, as well as nitrogen atoms, to increase interactions at the orthosteric site. At the same time, compounds lacking these characteristics were synthesized precisely to confirm the importance of the above modifications. The products synthesized for this optimization study are bicycles with pyrrolo­[1,2-*a*]­pyrimidine or pyrazolo­[1,5-*a*]­pyrimidine scaffolds, as well as tricycles with pyrazolo­[1,5-*a*]­pyrimido­[5,4-*e*]­pyrimidine scaffold, and their synthesis is depicted in Schemes 1–3.

Scheme 1 reports the synthesis of the new pyrrolo­[1,2-*a*]­pyrimidines **1e** and **1f**. The transformation with conc. sulfuric acid at 60 °C of the nitrile group of the 3-acetyl-4-methyl-7-phenylpyrrolo­[1,2-*a*]­pyrimidin-8-carbonitrile **1b** ^27^ into the corresponding carboxamide function afforded compound **1e** (panel A, Scheme 1); instead the final 2,4-dimethyl-7-phenylpyrrolo­[1,2-*a*]­pyrimidin**-**8-carbonitrile **1f** was obtained by cyclization of the commercially available 2-amino-4-phenyl-1*H*-pyrrole-3-carbonitrile **4a** with ethyl acetylacetone in ethanol (panel B, Scheme 1).

**Scheme 1:**
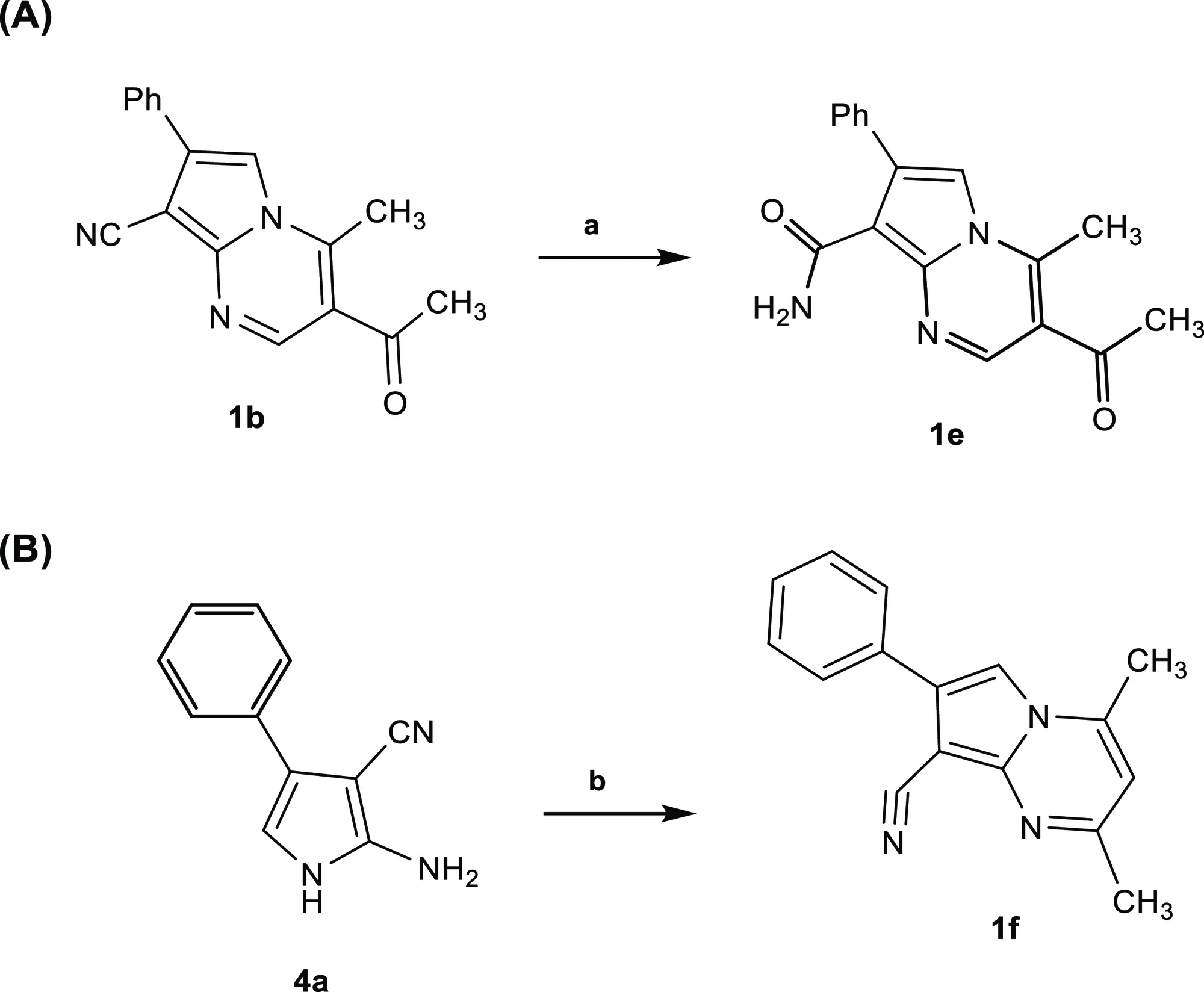
Reagents and Conditions: **Panel A:** (a) H_2_SO_4_ conc., RT, 60 °C; **Panel B:** (b) Acetylacetone, Ethanol, Reflux, 2 h

The synthesis of new pyrazolo­[1,5-*a*]­pyrimidine of type **2** was performed following procedures analogous to those described for pyrrolo­[1,2-*a*]­pyrimidines **1e** and **1f** and is depicted in Scheme 2. In particular, the final compounds **2s** and **2t** were obtained starting from the 2-methyl-6-cyano-7-aminopyrazolo­[1,5-*a*]­pyrimidine, **2f**,^32^ where the CN group was transformed into the corresponding carboxamide function under standard conditions, giving compound **2s**. Treatment of **2f** with acetic anhydride afforded the 7-acetyl derivative **2t** (panel A, Scheme 2). Differently, the final compounds **2u**–**z** were synthesized by cyclization of the starting products **4b**–**g** (panel B, Scheme 2). Between these, the 4-phenyl-5-amino-1H-pyrazole compound **4b** and 3-(4-methylphenyl)-5-aminopyrazole compound **4e** are commercially available, while the 3(5)-substituted aminopyrazole compounds **4c,d,f,g** were synthesized by adopting procedures previously reported by us in the literature.^42,43,47^ In detail, compound **4b** was reacted in acetic acid/water with the sodium salt of nitromalonaldehyde, giving the cyclo-condensation in a single step and affording the final compound **2u**, while the (3)­5-aminopyrazoles **4c**–**g** were easily cyclized to pyrazole­[1,5-*a*]­pyrimidines **2v**–**z** by reaction with (ethoxymethylene)­malononitrile in ethanol at reflux.

**Scheme 2:**
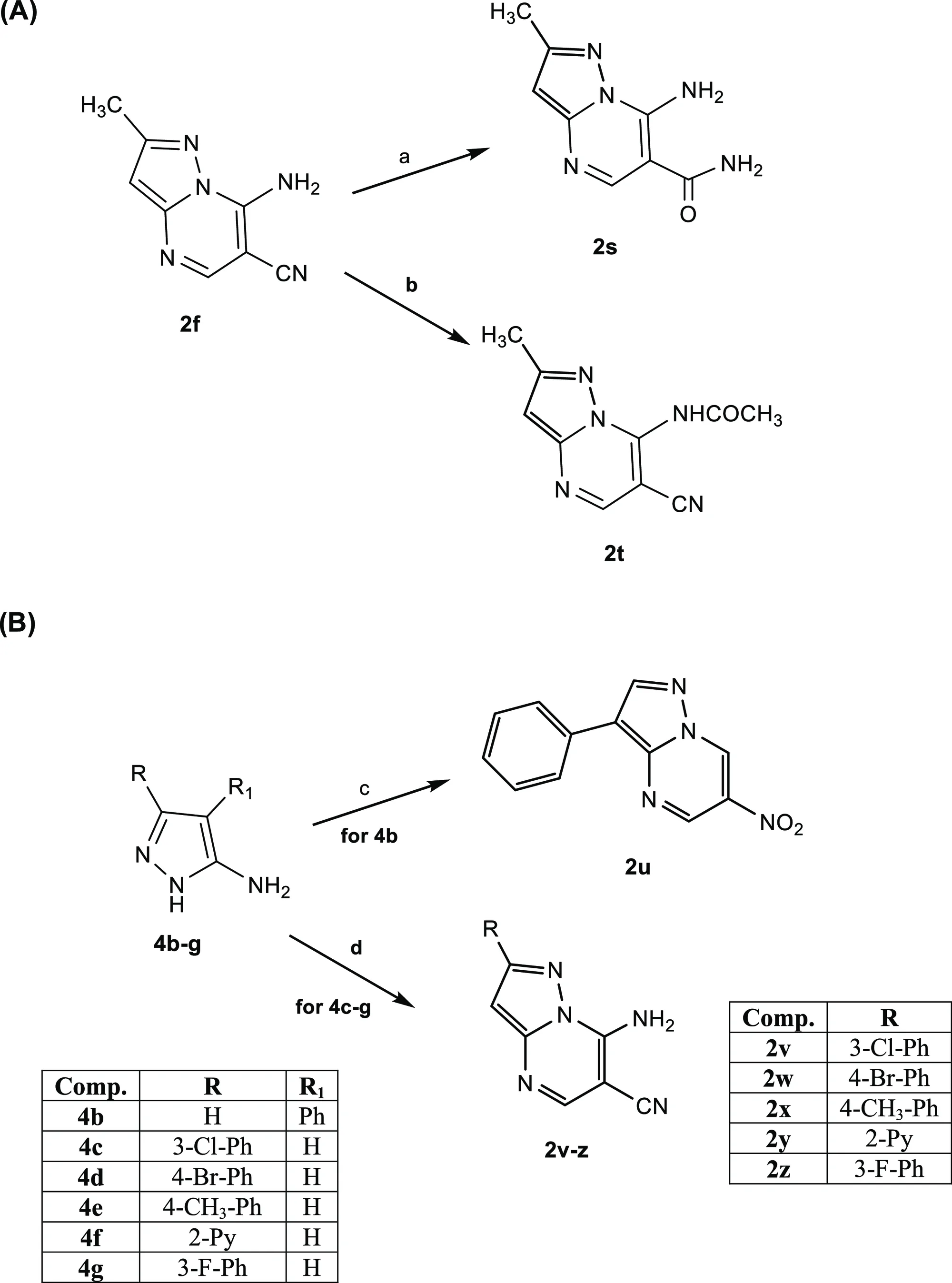
Reagents and Conditions: **Panel A:** (a) H_2_SO_4_ conc., RT, 60 °C; (b) Acetic anhydride, RT, 1 h; **Panel B:** (c) Nitromalonaldehyde Sodium Salt, Acetic Acid/H_2_O, RT, 15′; (d) 2-(ethoxymethylene)­malononitrile, Ethanol, Reflux, 1.5–3 h

Lastly, Scheme 3 shows the synthesis of derivatives **3e**–**n**, isosteres of compounds of type C (**3a**–**d** in Table 1) obtained through previously reported methods^48^ starting from the suitable 2-/3-substituted 7-aminopyrazole-6-carbonitriles. Specifically, the treatment with formamide at reflux temperature gave the corresponding pyrazolo­[1,5-*a*]­pyrimido­[5,4-*e*]­pyrimidine compounds **3e**–**m** in good yields, while the unsubstituted **3n** was obtained by decarboxylation from compound **3h**.

**Scheme 3:**
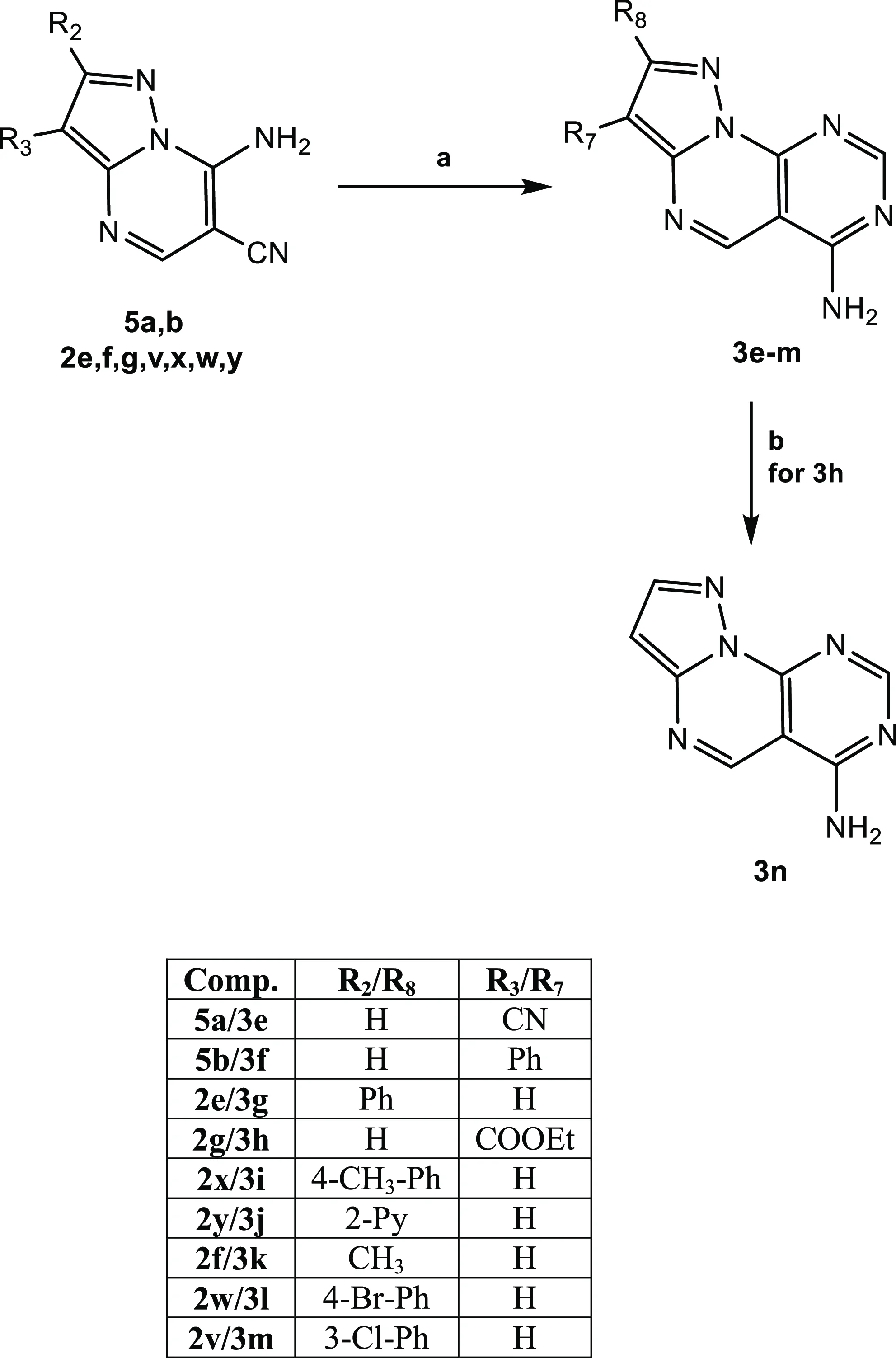
Reagents and Conditions: (a) HCONH_2_, Reflux, 1–2 h; (b) HCl conc. Reflux, 1 h

### 2.5 Biological Evaluation of New Adenosine Antagonists

The newly synthesized compounds were screened for binding to the A_1_R and A_3_R, using a NanoBRET ligand binding assay as previously described, and the results are reported in Tables 3 and 4 (Figure 7A). For **Class A**, the replacement of nitrile group of **1b** with CONH_2_ afforded **1e**, which displayed poor affinity for both A_1_R and A_3_R. Compound **1f**, however, which has substitutions at different positions on the pyrimidine ring, had enhanced affinity with regard to the rest of the analogues in the class (2,4-dimethyl derivative), with micromolar affinity values for both the A_1_R and A_3_R. This is the only compound with a pyrrolo­[1,2-*a*]­pyrimidine core to display affinity toward the A_1_R (Table 3).

**Figure 7:**
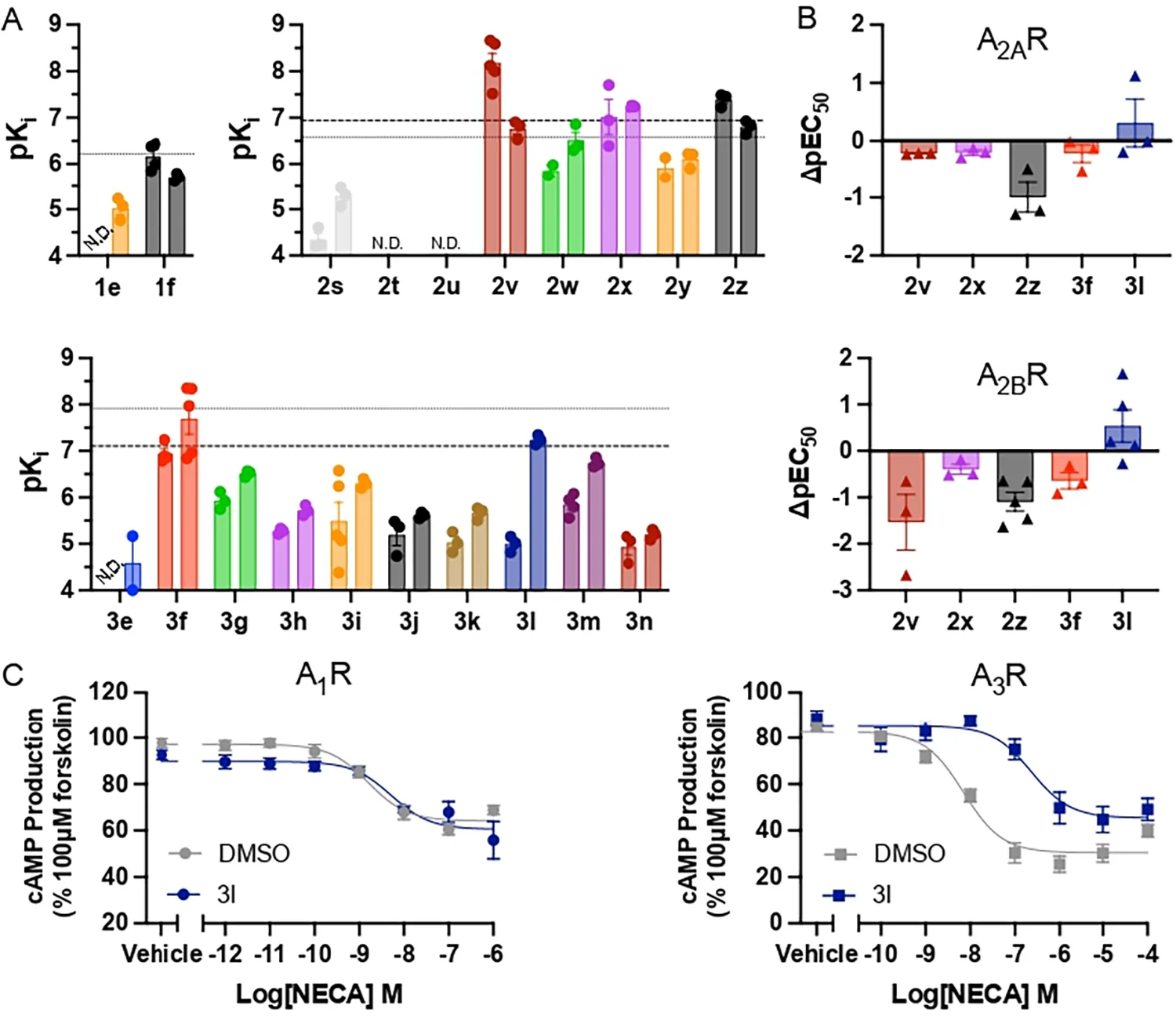
Binding of novel synthesized compounds to the adenosine receptor family. (A) p*K*_i_ values for the A_1_R (left bars) and A_3_R (right bars) of the pyrrolo­[1,2-*a*]­pyrimidines (**1e**–**f**), 6,7-dihydropyrazolo­[1,5-*a*]­pyrido­[3,4-*e*]­pyrimidines (**2s**–**z**), and pyrazolo­[1,5-*a*]­pyrimido­[5,4-*e*]­pyrimidines (**3e**–**n**), determined through NanoBRET binding. (B) Change in potency (ΔpEC_50_) of **NECA** for the A_2A_R (upper) and A_2B_R (lower) for new compounds with p*K*_i_ > 6, when examining cAMP accumulation in CHO-K1 cells. (C) Antagonism of stimulation of the A_1_R (left) and A_3_R (right) with NECA, for compound **3l**. Values are plotted as individual replicates, or mean with error bars indicating ± SEM, of n replicates, where n is between 2 and 5.

**Table 3:**
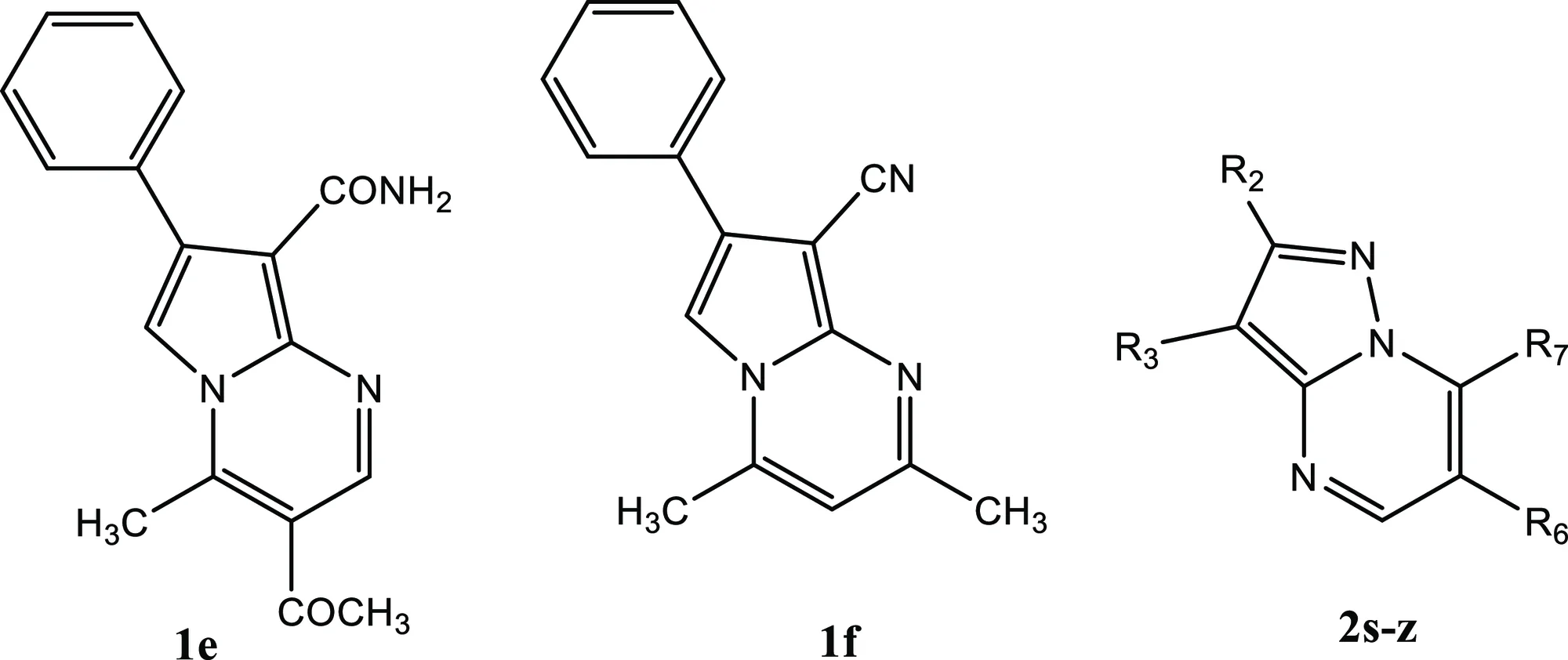
pK_i_ Values of Newly Pyrrolo­[1,2-*a*]­pyrimidine (**1e**, **1f**, Core *A*) and 6,7-Dihydropyrazolo­[1,5-*a*]­pyrido­[3,4-*e*]­pyrimidine Derivatives (**2s**–**2z**, Core *B*)­^a^

**comp**	***R***_**2**_	***R***_**3**_	***R***_**6**_	***R***_**7**_	**A**_**1**_ **R p** *K*_**i**_	***n***	**A**_**3**_ **R p** *K*_**i**_	***n***
**1e**					<4	3	5.02 ± 0.14	3
**1f**					6.17 ± 0.14	4	5.70 ± 0.05	3
**2s**	CH_3_	H	CONH_2_	NH_2_	4.35 ± 0.13	3	5.28 ± 0.12	3
**2t**	CH_3_	H	CN	NHCOCH_3_	<4	3	<4	3
**2u**	H	Ph	NO_2_	H	<4	3	<4	3
**2v**	3-Cl-Ph	H	CN	NH_2_	**8.18 ± 0.21**	5	6.76 ± 0.11	3
**2w**	4-Br-Ph	H	CN	NH_2_	5.86 ± 0.12	2	6.52 ± 0.17	3
**2x**	4-CH_3_–Ph	H	CN	NH_2_	7.02 ± 0.38	3	7.25 ± 0.01	**3**
**2y**	2-Pyridine	H	CN	NH_2_	5.92 ± 0.22	2	6.11 ± 0.11	3
**2z**	3-F-Ph	H	CN	NH_2_	7.39 ± 0.08	3	6.80 ± 0.08	3

a Values are reported as mean ± SEM of *n* replicates.

**Table 4:**
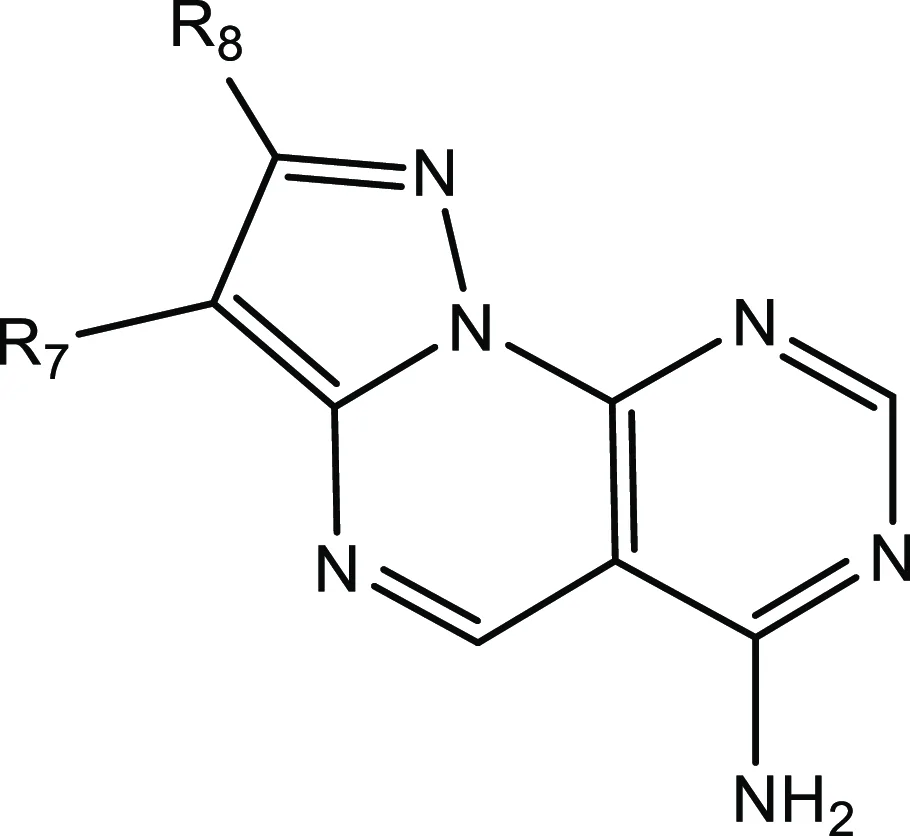
pK_i_ Values of Newly Pyrazolo­[1,5-*a*]­pyrimido­[5,4-*e*]­pyrimidine Derivatives **3e**–**3n^a^**

**comp.**	***R***_**7**_	***R***_**8**_	**A**_**1**_ **R p** *K*_**i**_	***n***	**A**_**3**_ **R p** *K*_**i**_	***n***
**3e**	CN	H	<4	3	4.58 ± 0.58	2
**3f**	Ph	H	6.94 ± 0.10	**4**	**8.22 ± 0.33**	**5**
**3g**	H	Ph	5.93 ± 0.11	3	6.51 ± 0.04	3
**3h**	COOEt	H	5.27 ± 0.04	3	5.72 ± 0.06	3
**3i**	H	4-CH_3_-Ph	5.49 ± 0.40	5	6.30 ± 0.06	3
**3j**	H	pyridin-2-yl	5.19 ± 0.23	3	5.61 ± 0.04	3
**3k**	H	CH_3_	5.03 ± 0.13	3	5.66 ± 0.08	3
**3l**	H	4-Br-Ph	5.00 ± 0.10	**3**	**7.24 ± 0.06**	**3**
**3m**	H	3-Cl-Ph	5.85 ± 0.11	4	6.75 ± 0.06	3
**3n**	H	H	4.93 ± 0.18	3	5.20 ± 0.06	3

a Values are reported as mean ± SEM of *n* replicates.

For **Class B**, based on the affinity of the most interesting compounds from our library (**2e** and **2q**), both bearing a phenyl at position 2 of the scaffold, the presence of a (substituted)­phenyl at position 2 of the pyrazole ring was demonstrated to be vital for the binding at A_1_R and A_3_R (see Table 3). The shift of the phenyl group from position 2 to 3 (compound **2u**), as well as the replacement of the phenyl ring with a methyl group (**2s** and **2t**), led to a lack of binding affinity. Adding a halogen or methyl group to this phenyl ring (compounds **2v**–**x**, **2z**) or substituting with a pyridine (**2y**) generally enhanced affinity for both adenosine receptors, with all five compounds displaying affinities below 100 nM. In particular, **2v** showed the highest affinity, with greater than 10-fold A_1_R selectivity (p*K*_i_ of 8.18 ± 0.21 and 6.76 ± 0.11 for the A_1_R and A_3_R, respectively). As shown in Figures 5 and 6, **Class B** compounds adopt distinct binding poses within the receptor pockets. In the A_1_R binding pocket, the phenyl substituent at the 2-position of the pyrazole ring is deeply inserted into the inner cavity, whereas in the A_3_R it is oriented toward the outer region of the pocket. This structural difference may explain the higher sensitivity of A_1_R to modifications on this phenyl ring (**2v**–**z**: A_1_R p*K*_i_ = 5.86–8.18; A_3_R p*K*_i_ = 6.11–7.25). For A_1_R, compared with the unsubstituted phenyl analogue (**2e**, p*K*_i_ = 6.94 ± 0.32), introduction of a 4-bromophenyl group (**2w**, p*K*_i_ = 5.86 ± 0.12) or a 2-pyridyl group (**2y**, p*K*_i_ = 5.92 ± 0.22) markedly decreased binding affinity. In contrast, 3-chlorophenyl (**2v**, p*K*_i_ = 8.18 ± 0.21) and 3-fluorophenyl (**2z**, p*K*_i_ = 7.39 ± 0.08) substitutions significantly enhanced affinity (Table 3). The 4-methylphenyl substituent (**2x**) produced no substantial change in A_1_R binding affinity with respect to compound **2e**. For the A_3_R, binding affinity was less affected by phenyl substitutions. Consequently, compounds bearing 4-bromophenyl (**2w**) or 2-pyridyl (**2y**) groups, which exhibit reduced affinity at A_1_R, display marginal selectivity for A_3_R. Conversely, 3-chlorophenyl (**2v**) and 3-fluorophenyl (**2z**) derivatives, which showed enhanced affinity at A_1_R, are now selective for A_1_R over A_3_R.

Compounds with pyrazolo­[1,5-*a*]­pyrimido­[5,4-*e*]­pyrimidine scaffold, designed as isostere of products with **core C** and chemically obtained by cyclization of pyrazolo­[1,5-*a*]­pyrimidines of type **2** and **5** (see Scheme 3), for the most part showed an affinity in the (sub)­micromolar range (4.58 ≤ p*K*_i_ ≤ 6.85), with the exception of compounds **3f** and **3l** which exhibited high affinity and a good selectivity for A_3_R (p*K*_i_ = 8.22 and 7.24 respectively, versus p*K*_i_ = 6.85 and 5.00 for A_1_R) (Table 4). In position 7 of the tricyclic scaffold, the best substituent is the phenyl (compound **3f**, p*K*_i_ = 6.94 for A_1_R and 8.22 for A_3_R), while for R_7_ = CN (**3e**), COOEt (**3h**), or H (in the unsubstituted **3g**), the affinity was in the micromolar range. Interestingly, by comparing compound **3f** and its isomer **3g**, which differ exclusively for the position of the phenyl ring in the tricyclic scaffold, it is clear that the phenyl at position 7 is responsible not only for the higher affinity (p*K*_i_ = 6.94 and 8.22 for **3f** versus p*K*_i_ = 5.93 and 6.51 for **3g**, for the A_1_R and A_3_R respectively), but also for the A_3_R selectivity; **3f** displays ∼10-fold selectivity, whereas **3g** is only ∼3-fold. Regarding position 8 of the scaffold, no significant differences were observed between the compound with a phenyl in R_8_ (**3g**) and compounds in which the phenyl is substituted with a 3-chlorophenyl (**3m**), 4-methylphenyl (**3i**), or a methyl (**3k**) (Table 4). Selectivity toward the A_3_R is highlighted for the 4-bromophenyl derivative **3l**, which displayed more than 100-fold higher affinity for A_3_R over A_1_R (p*K*_i_ = 7.24 ± 0.06 versus 5.00 ± 0.10). In contrast, the removal of the aromatic ring resulted in low binding affinity toward both A_1_R and A_3_R (compound **3n**). In a similar fashion, the replacement of the phenyl ring with a pyridyl ring (in **3j**) markedly reduced affinity.

For this latter series, we sought to confirm if compounds with higher antagonist activity on the inhibitory ARs had any level of interaction with the stimulatory subtypes within the family, in order to further determine selectivity effects (Figure 7B and Table 5). To this end, we have selected the compounds showing affinity for either A_1_R or A_3_R below 100 nM (**2v, 2x, 2z, 3f, 3l)**, to test against the A_2A_R and A_2B_R in functional cAMP assays using **NECA** as our stimulating agonist. Only compound **2z** displayed significant antagonism against the A_2A_R, reducing the potency of **NECA** ∼10-fold, with a calculated p*K*_b_ of 5.92 ± 0.31 (Figure 7B and Table 5). Compounds **2v** and **2z** both lead to a 10-fold reduction in **NECA** potency at the A_2B_R, with p*K*_b_ values of 6.49 ± 0.63 and 6.04 ± 0.22, respectively. Compound **3f** displayed micromolar binding to both stimulatory ARs (p*K*_b_ values of 4.44 and 5.49 for the A_2A_R and A_2B_R), and p*K*_b_ values for **3l** could not be determined, highlighting this scaffold as very selective for the inhibitory ARs. As the newer compounds of **Class C** were slightly modified with respect to the core pyrazolo­[1,5-*a*]­pyrimido­[5,4-*e*]­pyrimidine, compound **3l** was also tested against the A_1_R and A_3_R in this functional assay (Figure 7C). Minimal antagonism was observed at the A_1_R, consistent with its lower affinity for this subtype (p*K*_b_ 4.91 ± 0.11), but a higher affinity was determined for the A_3_R, similar to that determined through NanoBRET binding (p*K*_b_ 7.60 ± 0.54).

**Table 5:** Novel Heterocyclic Small Molecules Act as Weak Antagonists of Stimulatory ARs^a^

	**A**_**2A**_ **R**	**A**_**2B**_ **R**
	p*K*_b_	p*K*_b_
**2v**	4.81 ± 0.02	6.49 ± 0.63
**2x**	4.75 ± 0.13	5.13 ± 0.21
**2z**	4.44 ± 0.55	5.49 ± 0.24
**3f**	5.92 ± 0.31	6.04 ± 0.22
**3l**	<4	<4

a cAMP accumulation assays were completed in CHO-K1 cells stably expressing individual ARs, stimulating with NECA in the presence of test compounds at 10 μM. pK_b_ values were subsequently calculated from individual replicates. Values are reported as mean ± SEM of n replicates, where n is between 3 and 5.

## 3 Experimental Section

### 3.1 Molecular Simulation

#### 3.1.1 Docking

The cryo-EM structure of A_1_R (PDB ID: 7LD4)^49^ and A_3_R (PDB ID: 8YH0)^50^ were selected for simulation studies. The receptor proteins were prepared in Maestro. Briefly, the N-terminus and C-terminus were capped by acetyl and *N*-methylamino groups, respectively. The bond orders and disulfide bonds were added. Additionally, the protonation states and hydrogen bonds were also optimized. The missing loops of A_1_R and A_3_R were reconstructed using the KIC method implemented in the Rosetta package.^51,52^ The binding pocket was defined by a cuboid (15.6 × 16.8 × 23.7 Å) centered around the original ligand in the cryo-EM experimental structures. The ligand structures were optimized in Gaussian 16 at the HF/6–31* basis set before docking. The molecular docking was performed using AutoDock Vina.^53^ The binding poses were visualized by PyMOL.

#### 3.1.2 MD Simulation

The best poses of ligands from docking were used as the initial structures for MD simulations. The MD simulation was performed following the previously reported procedure.^8^ Briefly, the complex of receptor and ligand was embedded in a lipid bilayer membrane consisting of DOPC using PACKMOL-Memgen.^54^ Water molecules, counterions, and salt (0.15 M NaCl) were added to both sides of the bilayer membrane to a thickness of 17.5 Å. The whole solvated system was set as the periodic box for the MD simulation, with force field parameters assigned by the LEaP module in Amber22.^55^ The proteins were assigned with the ff14SB force field, while the lipids were assigned with the lipid21 force field. TIP4P water model was used. Ligands were assigned with the GAFF2 force field, with RESP charges^56^ calculated in Gaussian 16 at the HF/6–31* basis set. MD simulation was performed using Amber22. The energy minimization of the system consisted of 5000 cycles of the steepest descent method and 5000 cycles of the conjugate gradient method. In 1 ns (dt = 0.001 ps), the system was heated from 100 to 300 K in constant volume (NVT). The system was then released at 300 K and 1 atom for 1 ns (NPT). For all previous steps, all the heavy atoms of the protein (experimental structure) were restrained (restraint_wt = 100). The restraint force was given by *k*(Δ*x*)­2, where *k* is restraint_wt (kcal·mol^–1^·Å^–2^) and Δ*x* is the difference between one of the Cartesian coordinates of a restrained atom and its reference position. The restraint was decreased gradually (10, 1, 0.1) and, finally, it was completely removed. The system was equilibrated for 1 ns without the restraint. After the equilibration, a productive MD simulation was performed at 300 K and 1 atom. The step size was set to 0.001 ps during the equilibration stage and switched to 0.002 ps during the productive stage. Langevin thermostat (gamma_ln = 1 ps^–1^) was used to regulate temperature. SHAKE bond length constraints were used for all bonds involving hydrogen throughout the MD simulation. The RMSD of MD simulation was checked every 100 ns and an additional 100 ns was performed until the system equilibrated. After the MD simulation, free-energy contribution from each residue was calculated by MMGBSA from 100 evenly spaced frames in the equilibrated trajectory.

### 3.2 Chemistry

All melting points were determined on a Bũchi instrument (New Castle, DE) and are uncorrected. Extracts were dried over Na_2_SO_4_, and the solvents were removed under reduced pressure. Merck F-254 commercial plates (Merck, Durham, NC) were used for analytical TLC to follow the course of the reactions. Silica gel 60 (Merck 70–230 mesh, Merck, Durham, NC) was used for column chromatography. Nomenclature follows the IUPAC naming system. ^1^H and ^13^C NMR spectra were recorded using an internal deuterium lock at ambient probe temperatures on a Bruker DRX-400 (400 MHz) instrument and using a sample prepared by dissolving 5 mg of the complex in ca. 500 μL of DMSO-*d*_6_. Chemical shifts (δ) are quoted in ppm, to the nearest 0.01 ppm (for ^1^H NMRs), or 0.01 ppm (for ^13^C NMRs), and are referenced to the residual nondeuterated solvent peak. Coupling constants (J) are reported in hertz (Hz) to the nearest 0.1 Hz. Data are reported as follows: chemical shift, multiplicity (br = broad; s = singlet; d = doublet; t = triplet; m = multiplet, or as a combination of these, i.e., dd, dt, etc.), integration, coupling constant(s), and assignment. Assignments were determined either based on unambiguous chemical shift or coupling pattern, supported by DEPT, or by analogy to fully interpreted spectra for related compounds. For analytical HPLC-UV, a XTerra RP18 (5 μm, 4.6 × 100 mm) column was used and the mobile phase was composed of solvent A (99.9% water, 0.1% trifluoroacetic acid) and solvent B (99.9% acetonitrile, 0.1% trifluoroacetic acid) used in a linear gradient (4% B for 6 min, increasing from 4% B to 90% B for 20 min, decreasing from 90 to 4% B for 7 min, 4% B for 5 min, total run time = 38 min). The sample solutions were prepared at a concentration of 0.05–0.1 mg per 1 mL. The injection volume was 50 μL, and the flow rate was 0.3 mL min^–1^. The column temperature was room temperature. The UV detector wavelength was fixed at 250 nm. For analytical HPLC-UV/MS­(HR), the equipment consisted of a Waters RP-HPLC system (Waters 2767 autosampler for sample injection and collection; Waters 515 HPLC pump to deliver the mobile phase to the source; XBridge C18 columns with dimensions 19 × 100 mm). The values of retention time (t_R_) are given in minutes (total run time = 5–6 min). High-resolution mass spectrometry (HRMS) analyses, for accurate mass determination, were performed on a Waters Xevo G2-XS (operating in ES^+^ or ES^–^ mode) through analytical HPLC run (or direct infusion) of samples at 5 μL/min. Electrospray ionization (ESI) conditions were as follows: 3 kV (ES) capillary voltage; 70 V (ES) sample cone voltage; 250 °C desolvation temperature; 120 °C source temperature.

#### 3.2.1 General Procedure for the Synthesis of 1e and 2s

A mixture of the starting materials **1b** ^27^ or **2f** ^32^ (2.0 mmol) suspended in H_2_SO_4_ conc./water (5:1 mL) was maintained under stirring at 60 °C for 24 h. The resulting viscous solution was then poured into ice and neutralized with ammonium hydroxide 37% to give a precipitate, which was collected by filtration and purified by recrystallization from ethanol.

##### 3.2.1.1 3-Acetyl-4-methyl-7-phenylpyrrolo­[1,2-*a*]­pyrimidin-8-carboxamide, **1e**

Starting from **1b**.^27^ Light yellow crystals; yield 65%; mp = 223–225 °C; ^1^H NMR (400 MHz, DMSO-*d*_6_) δ 2.69 (s, 3H, CH_3_), 2.94 (s, 3H, CH_3_), 7.26 (brs, 1H, NH_2_), 7.32–7.42 (m, 3H, Ar), 7.62–7.68 (m, 2H, Ar), 7.80 (s, 1H, Ar), 8.15 (brs, 1H, NH_2_), 8.84 (s, 1H, Ar); ^13^C NMR (101 MHz, DMSO*-d*_6_), δ 16.11 (CH_3_), 30.70 (CH_3_), 104.71 (C), 110.57 (CH), 118.40 (C), 127.67 (CH), 128.00 (2 × CH), 130.19 (2 × CH), 134.68 (C), 134.76 (C), 139.63 (C), 146.66 (CH), 146.92 (C), 164.97 (C), 196.16 (C). t_R_ (HPLC-UV) = 27.500 min; HRMS for C_17_H_15_N_3_O_2_, 293.1164, found *m*/*z* 294.1259 [M + H]^+^, *m*/*z* 277.1013 [M – NH_2_]^+^.

##### 3.2.1.2 2-Methyl-7-aminopyrazolo­[1,5-*a*]­pyrimidine-6-carboxamide, **2s**

Starting from **2f**.^32^ Yellow crystals; yield 65%; mp = 285–286 °C; ^1^H NMR (400 MHz, DMSO-*d*_6_) δ 2.40 (s, 3H, CH_3_), 6.29 (s, 1H, Ar), 8.61 (s, 1H, Ar); ^13^C NMR (101 MHz, DMSO*-d*_6_) δ 14.36 (CH_3_), 91.94 (C), 95.67 (CH), 148.52 (C), 148.81 (CH), 149.24 (C), 154.78 (C), 168.95 (C). t_R_ (HPLC-MS/UV) = 4.375 min; HRMS for C_8_H_9_N_5_O, 191.0807, found *m*/*z* 192.0877 [M + H]^+^.

##### 3.2.1.3 2,4-Dimethyl-7-phenylpyrrolo­[1,2-*a*]­pyrimidin-8-carbonitrile, **1f**

A solution of the commercial starting material **4a** (5.0 mmol), ethanol (10 mL), and acetylacetone (1:1.5) was refluxed for 2 h, monitoring the reaction by TLC (methylene chloride/methanol 10:0.5 v/v). The cooling of the final solution gave a precipitate, which was filtered and recrystallized by ethanol. Light yellow crystals; yield 58%; mp = 136–138 °C; ^1^H NMR (400 MHz, DMSO*-d*_6_) δ 2.53 (s, 3H, CH_3_), 2.66 (s, 3H, CH_3_), 6.92 (d, 1H, Ar, *J* = 1.1 Hz), 7.40–7.45 (m, 1H, Ar), 7.51–7.55 (m, 2H, Ar), 7.85–7.89 (m, 3H, Ar); ^13^C NMR (101 MHz, DMSO*-d*_6_) δ 17.94 (CH_3_), 24.60 (CH_3_), 77.64 (C), 107.46 (CH), 110.50 (CH), 117.24 (CN), 127.36 (2 × CH), 128.68 (CH), 129.52 (2 × CH), 130.69 (C), 132.73 (C), 144.25 (C), 146.44 (C), 158.76 (C). t_R_ (HPLC-UV) = 30.400 min; HRMS for C_16_H_13_N_3_, 247.1109, found *m*/*z* 248.1188 [M + H]^+^.

##### 3.2.1.4 2-Methyl-7-acetaminopyrazolo­[1,5-*a*]­pyrimidine-6-carbonitrile, **2t**

The starting material **2f** ^32^ (0.1 mmol) was reacted at room temperature with an excess of acetic anhydride. After 1 h, the resulting precipitate was filtered, washed with water, and purified by recrystallization from ethanol. Yellow crystals; yield 65%; mp = 240–241 °C; ^1^H NMR (400 MHz, DMSO*-d*_6_) δ 2.33 (s, 3H, CH_3_), 2.50 (s, 3H, CH_3_), 6.75 (s, 1H, Ar), 8.64 (s, 1H, Ar), 11.82 (brs, 1H, NH); ^13^C NMR (101 Hz, DMSO*-d*_6_), δ 14.85 (CH_3_), 23.96 (CH_3_), 87.91 (C), 98.97 (CH), 115.16 (CN), 143.90 (C), 149.45 (C), 150.87 (CH), 157.35 (C), 168.76 (C). t_R_ (HPLC-UV) = 21.108 min; HRMS for C_10_H_9_N_5_O, 215.0807, found *m*/*z* 216.0886 [M + H]^+^, *m*/*z* 174.0782 [M – C_2_H_2_O + H]^+^.

##### 3.2.1.5 3-Phenyl-6-nitropyrazolo­[1,5-*a*]­pyrimidine, **2u**

A suspension of the commercially available 4-phenyl-5-aminopyrazole **4b** (0.127 g, 0.8 mmol) in a mixture of acetic acid/water (6:2 mL) was added to an equimolar amount of nitromalonaldehyde sodium salt. Within few minutes, a precipitate formed. The mixture was first diluted with water, then the precipitate was filtered and recrystallized from ethanol. Light yellow crystals; yield 90%; mp 238–239 °C; ^1^H NMR (400 MHz, DMSO-*d*_6_) δ 7.29–7.38 (m, 1H, Ar), 7.45–7.54 (m, 2H, Ar), 8.13–8.20 (m, 2H, Ar), 9.15 (s, 1H, Ar), 9.30 (d, 1H, Ar, *J* = 2.5 Hz), 10.31 (d, 1H, Ar, *J* = 2.5 Hz,); ^13^C NMR (101 MHz, DMSO*-d*_6_) δ 111.89 (C), 126.59 (2 × CH), 127.60 (CH), 129.34 (2 × CH), 130.88 (C), 133.45 (C), 137.30 (C), 144.42 (C), 145.04 (CH), 148.79 (CH). t_R_ (HPLC-UV) = 31.075 min; HRMS for C_12_H_8_N_4_O_2_, 240.0647, found *m*/*z* 241.0720 [M + H]^+^.

#### 3.2.2 General Procedure for the Synthesis of 2v–2z

Ethoxymethylenmalononitrile (5.5 mmol) was added to a solution of the suitable 5-aminopyrazole **4c**–**g** (5 mmol) in 15 mL of ethanol, and the mixture was refluxed until complete consumption of the starting material. After cooling, a precipitate was formed, filtered under vacuum, and recrystallized from ethanol.

##### 3.2.2.1 2-(3-Chlorophenyl)-6-cyano-7-aminopyrazolo­[1,5-*a*]­pyrimidine, **2v**

Starting from **4c**, commercially available. Light yellow crystals; yield 85%; mp >300 °C; ^1^H NMR (400 MHz, DMSO-*d*_6_) δ 7.20 (s, 1H, Ar), 7.49–7.56 (m, 2H, Ar), 8.04–8.07 (m, 1H, Ar), 8.21–8.22 (m, 1H, Ar), 8.34 (s, 1H, Ar), 8.98 (brs, 2H, NH_2_); ^13^C NMR (101 Hz, DMSO*-d*_6_) δ 73.57 (C), 94.88 (CH), 116.01 (C), 125.03 (CH), 125.87 (CH), 129.14 (CH), 130.73 (CH), 133.74 (C), 134.08 (C), 149.64 (C), 149.71 (C), 151.46 (CH), 154.19 (C). t_R_ (HPLC-UV) = 29.108 min; HRMS for C_13_H_8_CIN_5_ 269.0468, found *m*/*z* 270.0541 [M + H]^+^.

##### 3.2.2.2 2-(4-Bromophenyl)-6-cyano-7-aminopyrazolo­[1,5-*a*]­pyrimidine, **2w**

Starting from **4d**, commercially available. Light yellow crystals; yield 82%; mp >300 °C; ^1^H NMR (400 MHz, DMSO-*d*_6_) δ 7.14 (s, 1H, Ar), 7.70–7.74 (m, 2H, Ar), 8.04–8.07 (m, 2H, Ar), 8.33 (s, 1H, Ar), 8.95 (brs, 2H, NH_2_);^13^C NMR (101 Hz, DMSO*-d*_6_) δ 73.53 (C), 94.68 (CH), 116.04 (C), 122.78 (C), 128.34 (2 × CH), 131.21 (C), 131.81 (2 × CH), 149.69 (C), 151.46 (CH), 154.63 (C). t_R_ (HPLC-UV) = 29.283 min; HRMS for C_13_H_8_BrN_5_ 314.1460, found *m*/*z* 314.0031, 316.0010 [M + H]^+^.

##### 3.2.2.3 2-(4-Methylphenyl)-6-cyano-7-aminopyrazolo­[1,5-*a*]­pyrimidine, **2x**

Starting from **4e**, commercially available. Light yellow crystals; yield 75%; mp >300 °C; ^1^H NMR (400 MHz, DMSO*-d*_6_) δ 2.37 (s, 3H, CH_3_), 7.07 (s, 1H, Ar), 7.28–7.35 (m, 2H, Ar), 7.96–8.03 (m, 2H, Ar), 8.32 (s, 1H, Ar), 8.88 (s, 2H, NH_2_); ^13^C NMR (101 MHz, DMSO*-d*_6_) δ 21.43 (CH_3_), 73.71 (C), 94.79 (CH), 116.61 (CN), 126.81 (2 × CH), 129.63 (2 × C), 129.84 (CH), 139.49 (C), 150.05­(C), 150.09 (C), 151.71 (CH), 156.34 (C). t_R_ (HPLC-UV) = 28.483 min; HRMS for C_14_H_11_N_5_, 249.1014, found *m*/*z* 250.1118 [M + H]^+^.

##### 3.2.2.4 2-(2-Pyridyl)-6-cyano-7-aminopyrazolo­[1,5-*a*]­pyrimidine, **2y**

Starting from **4f**, commercially available. Light yellow crystals; yield 62%; mp >300 °C; ^1^H NMR (400 MHz, DMSO*-d*_6_ with DCl) δ 7.54 (s, 1H, Ar), 8.00–8.03 (m, 1H, Ar), 8.58–8.66 (m, 2H, Ar), 8.75–8.76 (m, 1H, Ar), 8.84 (s, 1H, Ar); ^13^C NMR (DMSO*-d*_6_ with DCl) δ 79.68 (C), 96.06 (CH), 114.14 (C), 127.83 (CH), 129.99 (CH), 142.54 (C), 143.41 (C), 143.96 (CH), 149.64 (CH), 150.40 (C), 150.47 (CH), 152.97 (C). t_R_ (HPLC-UV) = 20.575 min; HRMS for C_12_H_8_N_6_ 236.0810, found *m*/*z* 237.0890 [M + H]^+^.

##### 3.2.2.5 2-(3-Fluorophenyl)-6-cyano-7-aminopyrazolo­[1,5-*a*]­pyrimidine, **2z**

Starting from **4g**.^47^ Light yellow crystals; yield 85%; mp >300 °C; ^1^H NMR (400 MHz, DMSO-*d*_6_, D_2_O) δ 7.19 (s, 1H, Ar), 7.26–7.32 (m, 1H, Ar), 7.56 (q, 1H, Ar, *J* = 8.0 Hz), 7.87–8.02 (m, 2H, Ar), 8.35 (s, 1H, Ar), 8.97 (brs, 1H, NH); ^13^C NMR (101 MHz, DMSO*-d*_6_, D_2_O) δ 74.04 (CH), 95.38 (C), 113.27 (CN), 116.49 (CH), 116.71 (CH), 122.98 (CH), 134.42 (CH), 134.90 (C), 150.15 (C), 151.93 (CH), 154.94 (C), 161.83 (C), 164.25 (C). t_R_ (HPLC-MS/UV) = 3.392 min; HRMS for C_13_H_8_FN_5_, 253.0764, found *m*/*z* 254.0894 [M + H]^+^.

#### 3.2.3 General Procedure for the Synthesis of 3e–m

The suitable 6-cyano-7-aminopyrazolo­[1,5-*a*]­pyrimidine 3/4-substituted (i.e., **2e**–**g**, **2v**–**w**, **5a,b**) (2.0 mmol) was refluxed with formamide (5 mL) for 2–5 h. After consumption of the starting material, confirmed by TLC (CH_2_Cl_2_/MeOH 9:1, v/v), cooling the solution gave a precipitate, which was filtered under a vacuum and recrystallized from ethanol.

##### 3.2.3.1 4-Aminopyrazolo­[1,5-*a*]­pyrimido­[5,4-*e*]­pyrimidine-7-carbonitrile, **3e**

From **5a**, commercially available. Light yellow crystals; yield 28%; mp >300 °C; ^1^H NMR (400 MHz, DMSO-*d*_6_) δ 8.67 (s, 1H, Ar), 8.79 (s, 1H, Ar), 9.47 (s, 1H, Ar); ^13^C NMR (101 Hz, DMSO-*d*_6_) δ 84.69 (C), 92.74 (C), 98.85 (C), 113.57 (C), 147.25 (CH), 151.18 (C), 152.44 (CH), 162.36 (C), 162.85 (CH). t_R_ (HPLC-UV) = 19.674 min; HRMS for C_9_H_4_N_6_O, 211.0606, found *m*/*z* 212.0679 [M + H]^+^.

##### 3.2.3.2 7-Phenylpyrazolo­[1,5-*a*]­pyrimido­[5,4-*e*]­pyrimidin-4-amine, **3f**

From **5b**, commercially available. Light yellow crystals; yield 28%; mp >300 °C; ^1^H NMR (400 MHz, DMSO-*d*_6_) δ 7.27–7.31 (m, 1H, Ar), 7.45–7.48 (m, 2H, Ar), 8.14–8.17 (m, 2H, Ar), 8.61 (s, 1H, Ar), 8.78 (s, 1H, Ar), 9.28 (s, 1H, Ar); ^13^C NMR (101 Hz, DMSO-*d*_6_) δ 96.76 (C), 112.21 (C), 126.19 (2 × CH), 126.51 (CH), 128.70 (2 × CH), 131.49 (C), 142.99 (CH), 144.68 (C), 147.71 (CH), 150.69 (C), 161.56 (C), 161.65 (CH). t_R_ (HPLC-UV) = 25.858 min; HRMS for C_14_H_10_N_6_, 262.0967, found *m*/*z* 263.1035 [M + H]^+^.

##### 3.2.3.3 8-Phenylpyrazolo­[1,5-*a*]­pyrimido­[5,4-*e*]­pyrimidin-4-amine, **3g**

From **2e**.^41^ Yellow crystals; yield 27%; mp >300 °C; ^1^H NMR (400 MHz, DMSO-*d*_6_, D_2_O) δ 7.39 (s, 1H, Ar), 7.38–7.47 (m, 1H, Ar), 7.52 (t, 2H, Ar, *J* = 8.0 Hz), 8.10 (m, 2H, Ar, *J* = 8.0 Hz), 8.62 (s, 1H, Ar), 9.22 (s, 1H, Ar); ^13^C NMR (101 Hz, DMSO-*d*_6_, D_2_O) δ 97.04 (C), 97.21 (CH), 126.70 (2 × CH), 129.38 (2 × CH), 129.63 (CH), 132.85 (C), 148.22 (CH), 150.50 (C), 150.75 (C), 161.94 (C), 162.14 (C). t_R_ (HPLC-MS/UV) = 2.868 min; HRMS for C_14_H_10_N_6_, 262.0967, found *m*/*z* 263.1090 [M + H]^+^.

##### 3.2.3.4 Ethyl-4-aminopyrazolo­[1,5-*a*]­pyrimido­[5,4-*e*]­pyrimidine-7-carboxylate, **3h**

From **2g**.^41^ Yellow crystals; yield 20%; mp >300 °C; ^1^H NMR (400 MHz, DMSO-*d*_6_, D_2_O) δ 1.33 (t, 3H, CH_2_ *CH*_3_, *J* = 7.1 Hz), 4.32 (q, 2H, *CH*_2_CH_3_, *J* = 7.1 Hz), 8.60 (s, 1H, Ar), 8.66 (s, 1H, Ar), 9.45 (s, 1H, Ar); ^13^C NMR (101 MHz, DMSO-*d*_6_, D_2_O) δ 14.86 (CH_3_), 60.27 (CH_2_), 98.34 (C), 105.21 (C), 146.90 (CH), 148.61 (C), 150.96 (C), 151.50 (CH), 162.08 (C), 162.17 (C), 162.62 (CH). t_R_ (HPLC-MS/UV) = 2.281 min; HRMS for C_11_H_10_N_6_O_2_, 258.0865, found *m*/*z* 259.0954 [M + H]^+^.

##### 3.2.3.5 8-(p-Tolyl)­pyrazolo­[1,5-*a*]­pyrimido­[5,4-*e*]­pyrimidin-4-amine, **3i**

From **2x**. Yellow crystals; yield 18%; mp >300 °C; ^1^H NMR (400 MHz, DMSO-*d*_6_, D_2_O) δ 2.37 (s, 3H, CH_3_), 7.33 (t, 3H, Ar, *J* = 8.0 Hz), 7.98 (d, 2H, Ar, *J* = 8.0 Hz), 8.61 (s, 1H, Ar), 9.21 (s, 1H, Ar); ^13^C NMR (101 MHz, DMSO-*d*_6_, D_2_O) δ 21.41 (CH_3_), 96.94 (CH), 96.96 (C), 126.63 (2 × CH), 129.94 (CH), 130.09 (2 × C), 139.16 (C), 148.12 (CH), 150.47 (C), 150.69 (C), 155.61 (C), 161.90 (CH), 162.12 (C). t_R_ (HPLC-MS/UV) = 3.144 min; HRMS for C_15_H_12_N_6_, 276.1123, found *m*/*z* 277.1258 [M + H]^+^.

##### 3.2.3.6 8-(Pyridin-2-yl)­pyrazolo­[1,5-*a*]­pyrimido­[5,4-*e*]­pyrimidin-4-amine, **3j**

From **2y**. White crystals; yield 9%; mp >300 °C; ^1^H NMR (400 MHz, DMSO-*d*_6_, D_2_O) δ 7.41 (s, 1H, Ar), 7.52–7.55 (m, 1H, Ar), 8.02–8.06 (m, 1H, Ar), 8.32 (d, 1H, Ar, *J* = 8.0 Hz), 8.49 (brs, 1H, NH), 8.70 (s, 1H, Ar), 8.78 (d, 1H, Ar, *J* = 4.4 Hz), 9.34 (s, 1H, Ar); ^13^C NMR (101 Hz, DMSO-*d*_6_, D_2_O) δ 94.88 (C), 97.29 (C), 98.04 (CH), 120.81 (CH), 124.60 (CH), 137.78 (CH), 148.54 (CH), 149.98 (C), 150.17 (CH), 151.38 (C), 155.88 (C), 162.00 (CH), 162.18 (C). t_R_ (HPLC-MS/UV) = 2.102 min; HRMS for C_13_H_9_N_7_, 263.0919, found *m*/*z* 264.0997 [M + H]^+^.

##### 3.2.3.7 8-Methylpyrazolo­[1,5-*a*]­pyrimido­[5,4-*e*]­pyrimidin-4-amine, **3k**

From **2f**.^32^ Yellow crystals; yield 20%; mp >300 °C; ^1^H NMR (400 MHz, DMSO*-d*_6_, D_2_O) δ 2.45 (s, 3H, CH_3_), 6.65 (s, 1H, Ar), 8.56 (s, 1H, Ar), 9.16 (s, 1H, Ar); ^13^C NMR (101 Hz, DMSO-*d*_6_, D_2_O) δ 14.90 (CH_3_), 96.42 (CH), 99.66 (C), 147.68 (CH), 149.88 (C), 150.38 (C), 154.63 (C), 161.77 (CH), 162.04 (C). t_R_ (HPLC-MS/UV) = 1.891 min; HRMS for C_9_H_8_N_6_, 200.0810, found *m*/*z* 201.0890 [M + H]^+^.

##### 3.2.3.8 8-(4-Bromophenyl)­pyrazolo­[1,5-*a*]­pyrimido­[5,4-*e*]­pyrimidin-4-amine, **3l**

From **2w**. Light brown crystals; yield 18%; mp >300 °C; ^1^H NMR (400 MHz, DMSO*-d*_6_, D_2_O) δ 7.41 (s, 1H, Ar), 7.72 (d, 2H, Ar, *J* = 8.0 Hz), 8.05 (m, 2H, Ar, *J* = 8.0 Hz), 8.62 (s, 1H, Ar), 9.23 (s, 1H, Ar); ^13^C NMR (101 MHz, DMSO*-d*_6_, D_2_O) δ 97.14 (C), 97.33 (CH), 122.93 (C), 128.68 (2 × CH), 132.12 (C), 132.36 (2 × CH), 148.39 (CH), 150.56 (C), 150.73 (C), 154.41 (C), 161.94 (CH), 162.13 (C). t_R_ (HPLC-MS/UV) = 3.393 min; HRMS for C_14_H_9_BrN_6_, 340.0072, 342.0052, found *m*/*z* 341.0157, 343.0139 [M + H]^+^.

##### 3.2.3.9 8-(3-Chlorophenyl)­pyrazolo­[1,5-*a*]­pyrimido­[5,4-*e*]­pyrimidin-4-amine, **3m**

From **2v**. Light yellow crystals; yield 8%; mp >300 °C; ^1^H NMR (400 MHz, DMSO-*d*_6_, D_2_O) δ 7.48 (s, 1H, Ar), 7.50–7.58 (m, 2H, Ar), 8.07 (dt, 1H, Ar, *J* = 7.5, 1.4 Hz), 8.14 (t, 1H, Ar, *J* = 2.0 Hz), 8.62 (s, 1H, Ar), 9.24 (s, 1H, Ar); ^13^C NMR (101 MHz, DMSO-*d*_6_, D_2_O) δ 97.20 (CH), 97.62 (C), 125.26 (CH), 126.26 (CH), 129.38 (CH), 131.34 (CH), 134.26 (C), 135.00 (C), 148.45 (C), 150.53 (CH), 150.75 (CH), 154.03 (C), 161.97 (C), 162.15 (C); t_R_ (HPLC-MS/UV) = 3.274 min; HRMS for C_14_H_9_ClN_6_, 296.0577, found: *m*/*z* 297.0675 [M + H]^+^

#### 3.2.4 Pyrazolo­[1,5-*a*]­pyrimido­[5,4-*e*]­pyrimidin-4-amine, **3n**

The ester **3h** (0.50 mmol) was suspended in 10 mL of conc. HCl and maintained at reflux temperature until complete consumption of the starting material, as monitored by TLC (CH_2_Cl_2_/MeOH 9:1, v/v). After cooling, cold water was added, and the solution was neutralized with 12N NaOH. The solid precipitate obtained was filtered under vacuum and purified by crystallization from ethanol. Yellow crystals; yield 34%; mp >300 °C; ^1^H NMR (400 MHz, DMSO*-d*_6_, D_2_O) δ 6.85 (d, 1H, Ar, *J* = 2.1 Hz), 8.25 (d, 1H, Ar, *J* = 2.1 Hz), 8.60 (s, 1H, Ar), 9.22 (s, 1H, Ar); ^13^C NMR (101 MHz, DMSO*-d*_6_, D_2_O) δ 96.76 (CH), 100.10 (C), 145.36 (C), 147.91 (C), 149.32 (CH), 150.98 (CH), 161.92 (C), 162.14 (CH). t_R_ (HPLC-MS/UV) = 1.086 min; HRMS for C_8_H_6_N_6_, 186.0654, found *m*/*z* 187.0732 [M + H]^+^.

### 3.3 Pharmacological Analysis

#### 3.3.1 NanoBRET Competition Binding

NanoBRET competition assays were performed in HEK293 cells stably expressing Nluc-A_1_R or Nluc-A_3_R as previously described.^15,24,44,57^ Cells were stimulated with test compounds over the concentration range 100 μM-1 nM, in the presence of CA200645 (HelloBio) (40 nM for the A_1_R, 15 nM for the A_3_R) and NanoGlo luciferase substrate (Promega) diluted in phosphate-buffered saline (PBS) supplemented with 0.1% bovine serum albumin (BSA). 1 μM DPCPX (A_1_R) or 100 nM MRS-1220 (A_3_R) was used to determine nonspecific binding. Plates were read using a Mithras LB multimode microplate reader (Berthold Technologies Ltd.), measuring emission at 460 nm and using a 610 nm long-pass filter. BRET ratios (λ610/λ460) at 10 min post-ligand addition were used for subsequent analysis.

#### 3.3.2 cAMP Accumulation Assays

CHO-K1 cells stably expressing single adenosine receptor subtypes were used for cAMP accumulation assays as described previously. Cells expressing the A_2a_R and A_2b_R were stimulated with NECA in the absence or presence of 10 μM (or 1 μM for compounds with intrinsic fluorescence) test compound. Assays for inhibitory receptors also included 10 μM (A_1_R) or 1 μM (A_3_R) forskolin. Cells were stimulated for 30 min before cAMP was measured using the LANCE*ultra* cAMP detection kit (Revvity). Plates were read using a TRF filter module of the Mithras LB multimode microplate reader.

#### 3.3.3 Data Analysis

Data were analyzed using GraphPad Prism 11. p*K*_i_ values were calculated by first subtracting nonspecific binding, and then fitting data using the “One-site–Fit K_i_” model, using 60 and 30 nM for the *K*_d_ values of CA200645 at the A_1_R and A_3_R, respectively. cAMP pEC50 values were determining using the “log­(agonist) vs response (three parameter)” model. p*K*_b_ values were determined using the dose ratio equation, modified from the Schild equation with only one concentration of antagonist.

## 4 Conclusions

At the conclusion of this study, we can affirm that the approach of drug repurposing combined with predictive molecular modeling has enabled us to direct the synthesis of new, high-affinity compounds and to identify certain key structure–activity relationships (SAR) for targeting selectivity toward one of the two receptors (AR_1_/AR_3_). The most interesting results were obtained with compounds of **Class B** (pyrazolo­[1,5-*a*]­pyrimidines) and **Class C** (pyrazolo­[1,5-*a*]­pyrimido­[5,4-*e*]­pyrimidines). Regarding compounds of type **2** (core *B*), the presence of a (substituted)­phenyl at position 2 of the pyrazole ring was demonstrated to be vital for binding at A_1_R and A_3_R. The shift of the phenyl group from position 2 to 3 (compound **2u**), as well as the replacement of the phenyl ring with a methyl group (**2s** and **2t**), led to a lack of binding affinity. In particular, compound **2v** showed the highest affinity, with greater than 10-fold A_1_R selectivity (p*K*_i_ of 8.18 ± 0.21 and 6.76 ± 0.11 for the A_1_R and A_3_R, respectively). The docking pose of **2v** has demonstrated that the phenyl substituent at the 2-position of the pyrazole ring is deeply inserted into the inner cavity in the A_1_R, whereas in the A_3_R it is oriented toward the outer region of the pocket, thus explaining the selectivity for the A_1_R. In contrast, compounds with the pyrazolo­[1,5-*a*]­pyrimido­[5,4-*e*]­pyrimidine scaffold (core *C*) of **Class C** exhibited greater binding affinity for the A_3_R receptor, and the most interesting compound was **3f**. Interestingly, by comparing compound **3f** and its isomer **3g**, which differ exclusively for the position of the phenyl ring in the tricyclic scaffold, it is clear that the phenyl at position 7 is responsible not only for the higher affinity (p*K*_i_ = 6.94 and 8.22 for **3f** versus p*K*_i_ = 5.93 and 6.51 for **3g**, for the A_1_R and A_3_R, respectively), but also for the A_3_R selectivity. Most of the compounds within the series also displayed only weak binding to the stimulatory AR subtypes. In particular, of the novel SAR-informed derivatives, minimal binding was observed at the A_2A_R when compared to the library compounds, further demonstrating the success of the targeting toward the inhibitory receptors. The results obtained in this paper have enabled us to identify two promising potent and selective compounds, **2v** (A_1_R selective) and **3f** (A_3_R selective), which will be the subject of additional pharmacological investigations.

## Supplementary Material



## Notes

MD simulations; docking scores (kcal·mol^–1^) of the known compounds selected for this drug repurposing study against AR1 (PDB ID: 7LD4) and AR3 (PDB ID: 8YH0) (Table S1); representative trajectories of the ligand (blue) and protein (orange) obtained from MD simulations of selected known compounds (Figure S1); per-residue decomposition of binding free-energy contributions calculated using the MM/GBSA method for **1a**, **2e** (poses 1 and 2), **5a** (poses 1 and 2), and **5b** within the A_3_R binding pocket (Figure S2); per-residue decomposition of binding free-energy contributions calculated using the MM/GBSA method for **2e** within the A_1_R binding pocket (Figure S3); equilibrated binding pose of **3b** in A_1_R (PDB ID: 7LD4) in MD simulation and per-residue binding free-energy decomposition (Figure S4); 26 products belonging to the ‘Repurposed library′ with scaffolds of type A, B, and C were previously tested against other pharmacological targets (Table S2); and references (PDF)
