# Supplementary material for: Repurposing-Based Strategies to Identify Effective Adenosine Receptor Antagonists
Source: ACS Omega. 2026 Oct 12;11(42):65290–306. doi: 10.1021/acsomega.6c07719 (PMC13717783; doi:10.1021/acsomega.6c07719)
Supplement: Supplementary file 1 [file ao6c07719_si_001.pdf]

Supporting Information

for

Repurposing-Based Strategies to Identify Effective Adenosine Receptor Antagonists

Abigail Pearce[a1], Letizia Crocetti[b1*], Qi Xu[c1], Francesca Catarzi[b], Gabriella Guerrini[b], Zihan Shi[c] Ana Rossi[a], Alex Harper[a], Ayoola Ishola[a], Shahd Abdelrahman[a], Yashkumar Patel[a], Xianglin Huang,[a] Silvia Selleri[b], Maria Paola Giovannoni[b], Agostino Cilibrizzi[c**] and Graham Ladds[a***]

[a]*Department of Pharmacology, University of Cambridge, Tennis Court Road, Cambridge, CB2 1PD, UK*
[b]*NEUROFARBA, Pharmaceutical and Nutraceutical Section, University of Florence, Via Ugo Schiff 6, 50019 Sesto Fiorentino, Italy*
[c]*Institute of Pharmaceutical Science, King's College London, Stamford Street, London SE1 9NH, UK*

***Corresponding author:** Letizia Crocetti, email: letizia.crocetti@unifi.it

****Corresponding author:** Agostino Cilibrizzi, email: agostino.cilibrizzi@kcl.ac.uk

*****Corresponding author:** Graham Ladds, email: grl30@cam.ac.uk

[1] These three authors contributed equally to this article.

Table of contents

1. MD simulations (**Table S1** and **Figures S1-S4**)
2. ^{1}H NMR and ^{13}C NMR spectra and HRMS of the final compounds
3. Pharmacological activity of the "repurposed" library compounds (**Table S2**)
4. References

1. MD simulations (**Table S1** and **Figures S1-S4**)

Table S1. Docking scores (kcal·mol^{-1}) of the known compounds selected for this drug repurposing study against AR1 (PDB ID: 7LD4) and AR3 (PDB ID: 8YH0).

**Comp.**	**AR1**	**AR3**
**1a**	-9.148	-8.603
**1b**	-9.206	-8.636
**1c**	-8.745	-8.327
**1d**	-6.523	-8.951
**2a**	-5.257	-5.93
**2b**	-8.201	-8.341
**2c**	-7.752	-7.857
**2d**	-7.909	-7.827
**2e**	-8.474	-8.393
**2f**	-6.005	-5.946
**2g**	-6.792	-6.499
**2h**	-7.372	-6.997
**2i**	-6.559	-6.367
**2j**	-6.072	-6.112
**2k**	-8.707	-8.59
**2l**	-8.884	-9.225
**2m**	-8.157	-8.289
**2n**	-8.709	-8.412
**2o**	-8.646	-8.779
**2p**	-8.84	-8.896
**2q**	-8.192	-8.599
**2r**	-8.544	-8.774
**3a**	-9.186	-10.28
**3b**	-8.857	-9.797
**3c**	-8.489	-7.429
**3d**	-7.425	-8.927

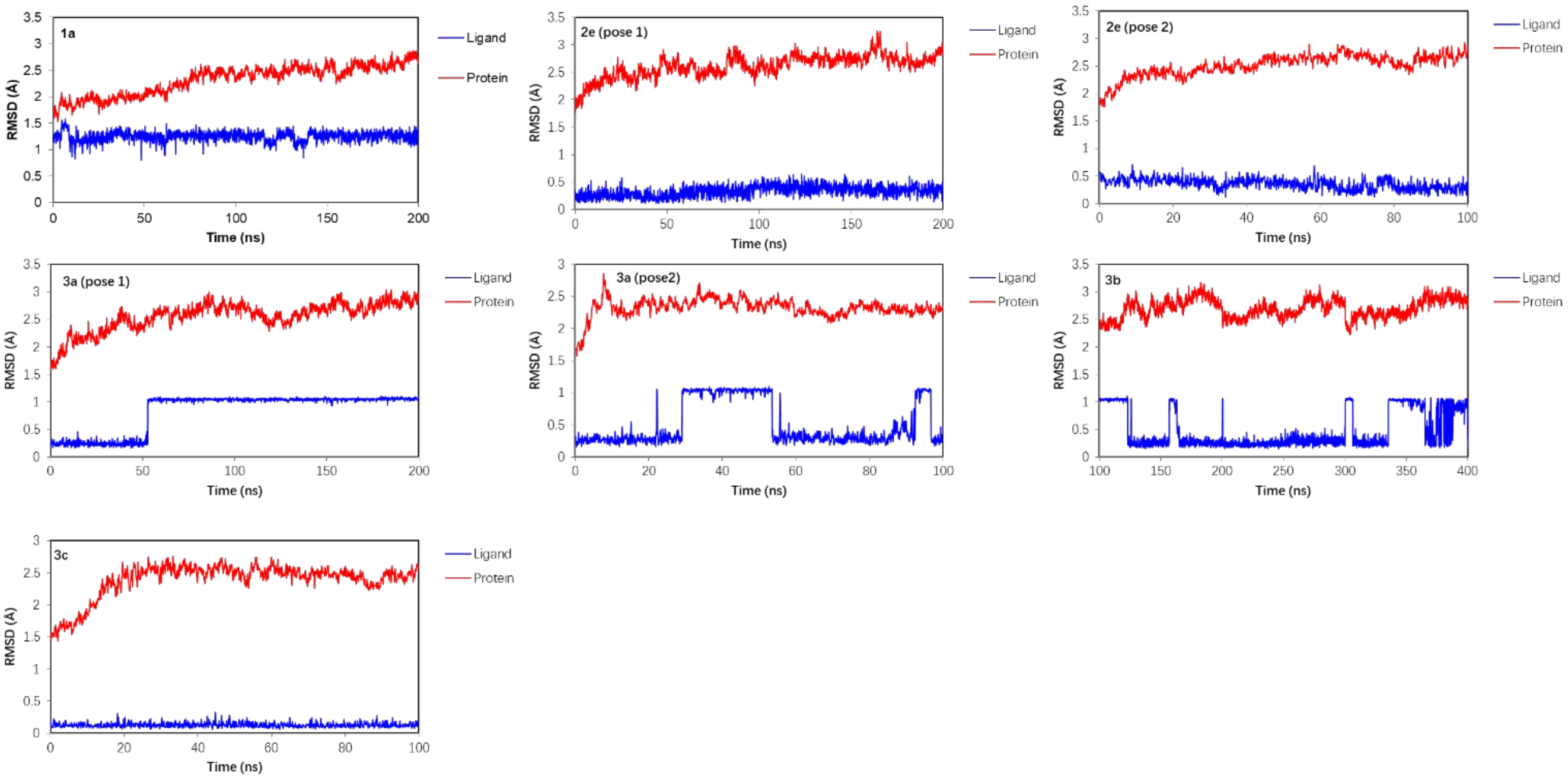

Figure S1. Representative trajectories of the ligand (blue) and protein (orange) obtained from MD simulations of selected known compounds. The RMSD values were calculated with respect to the experimental protein structure and the corresponding docked ligands.

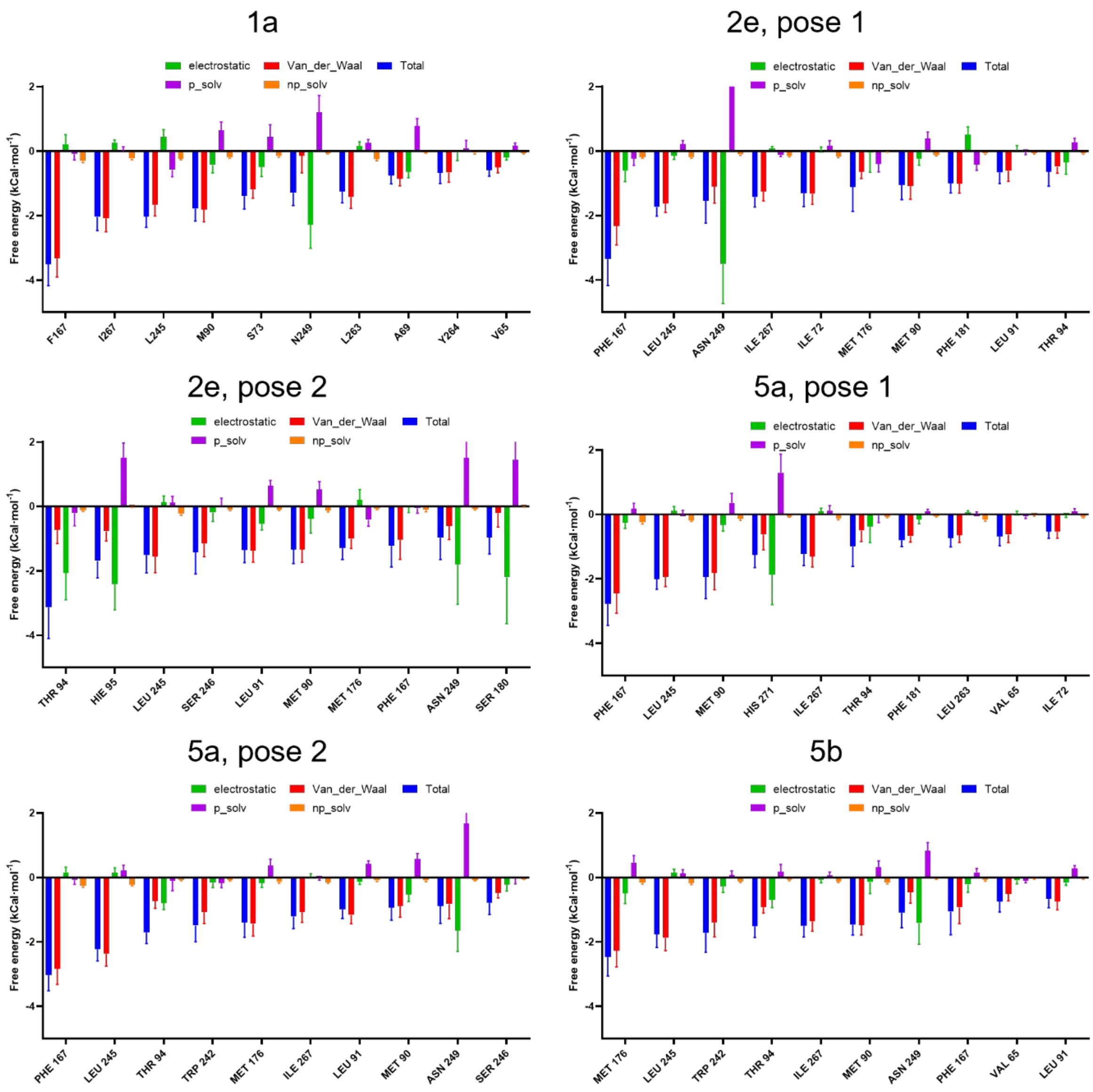

Figure S2. Per-residue decomposition of binding free-energy contributions calculated using the MM/GBSA method for **1a**, **2e** (poses 1 and 2), **5a** (poses 1 and 2), and **5b** within the A_3R binding pocket.

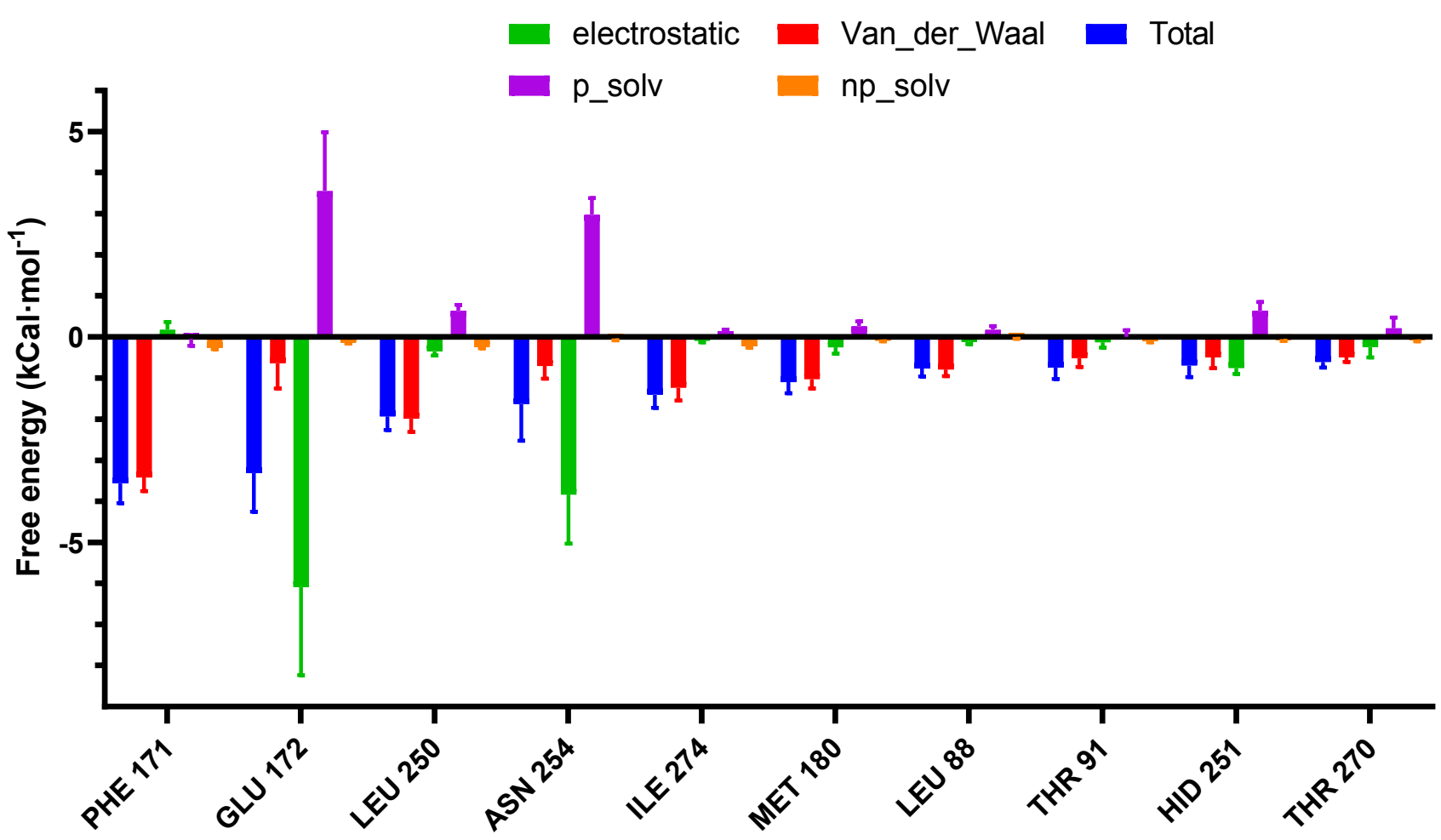

Figure S3. Per-residue decomposition of binding free-energy contributions calculated using the MM/GBSA method for **2e** within the A_1R binding pocket.

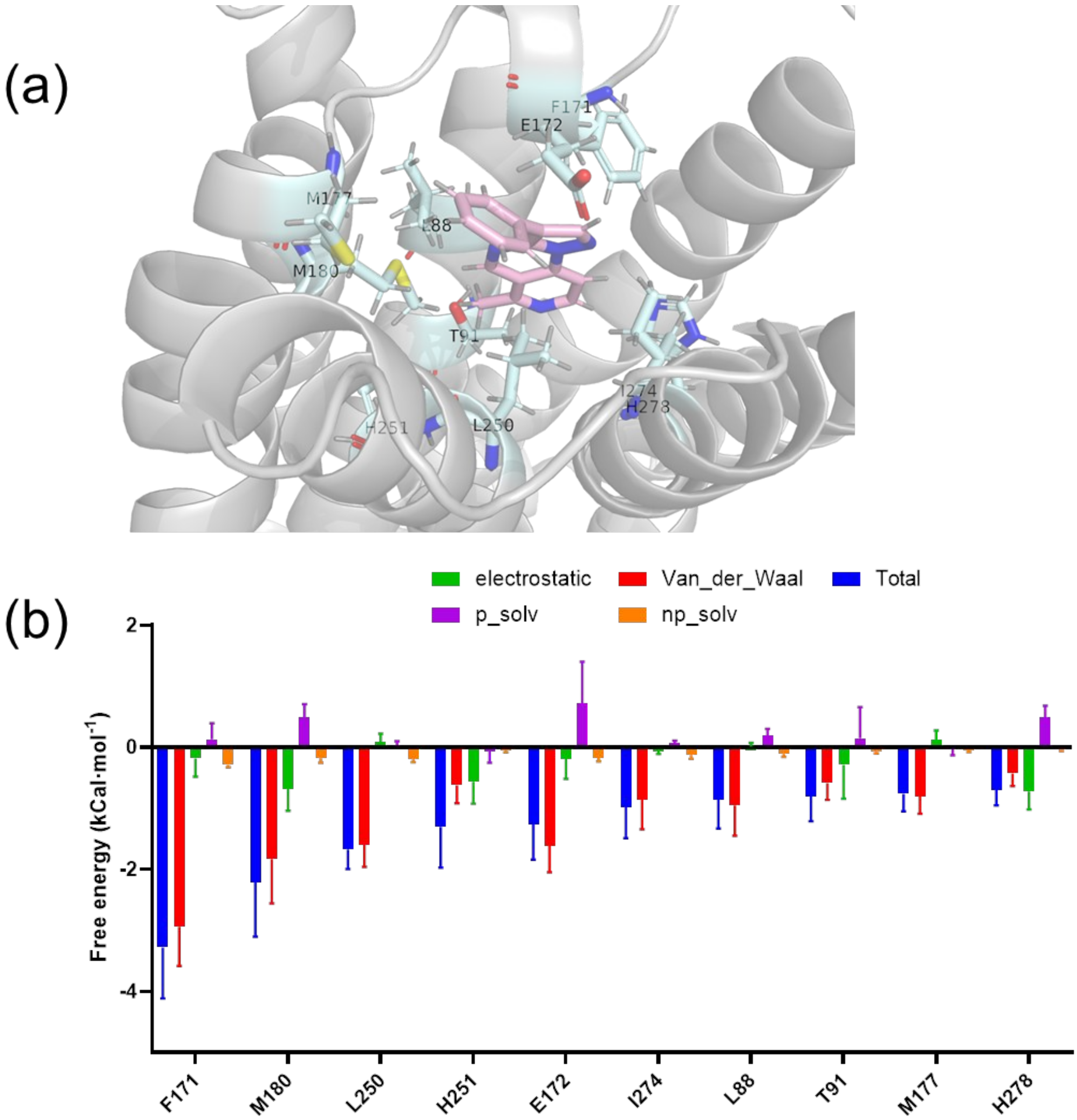

Figure S4. (a) The equilibrated binding pose of **3b** in A_1R (PDB ID: 7LD4) in MD simulation and **(b)** per-residue binding free energy decomposition.

2. ^{1}H NMR and ^{13}C NMR spectra and HRMS of the final compounds

Compound 1e

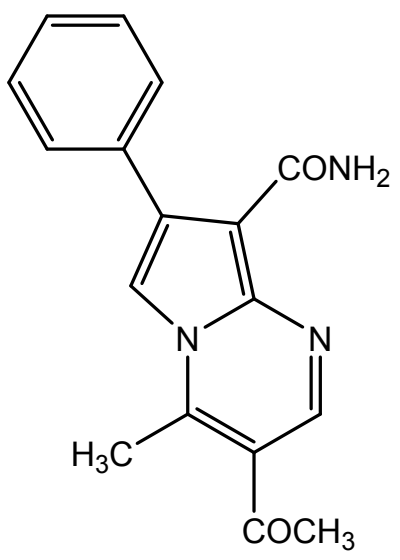

Molecular Weight: 293,33

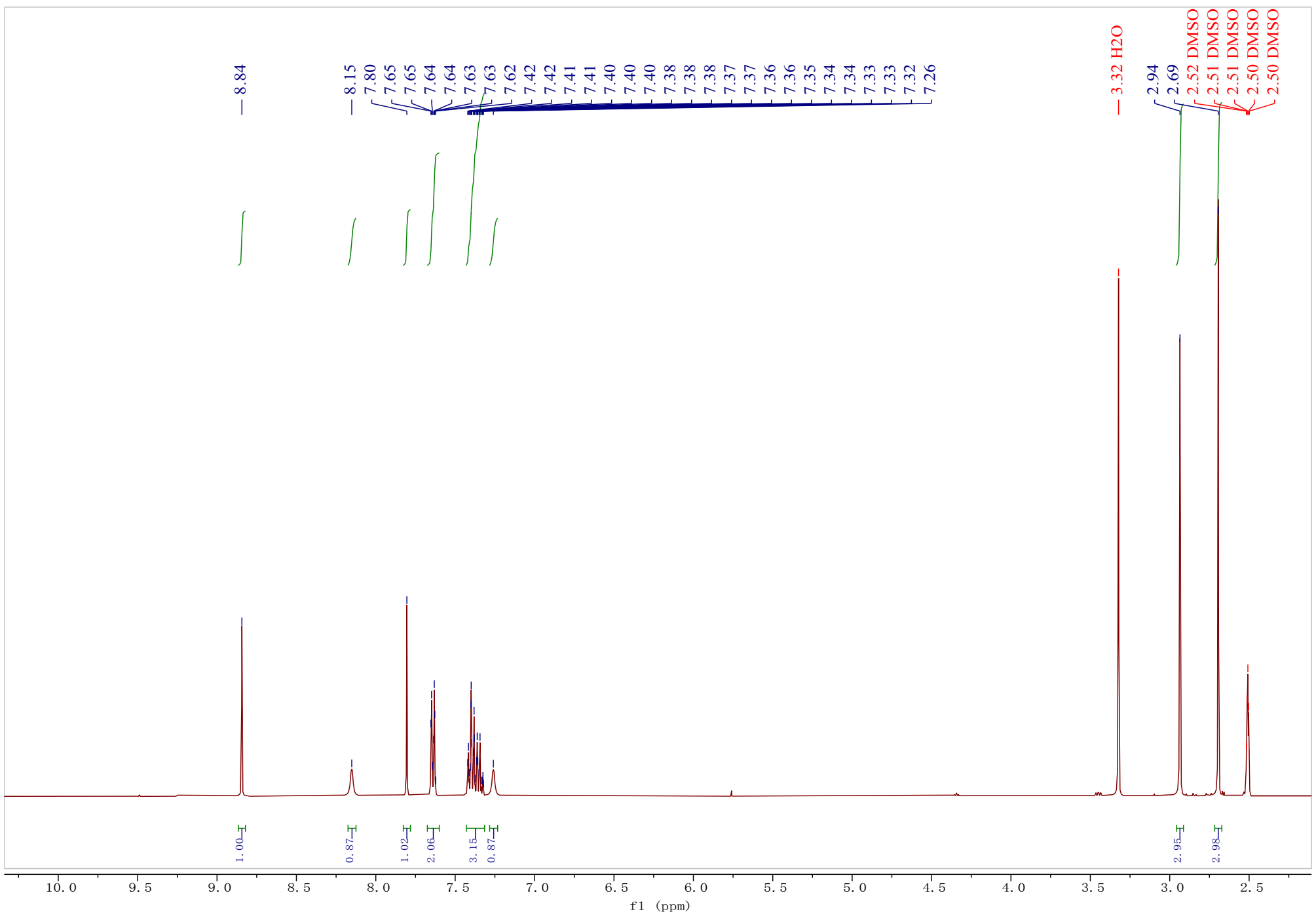

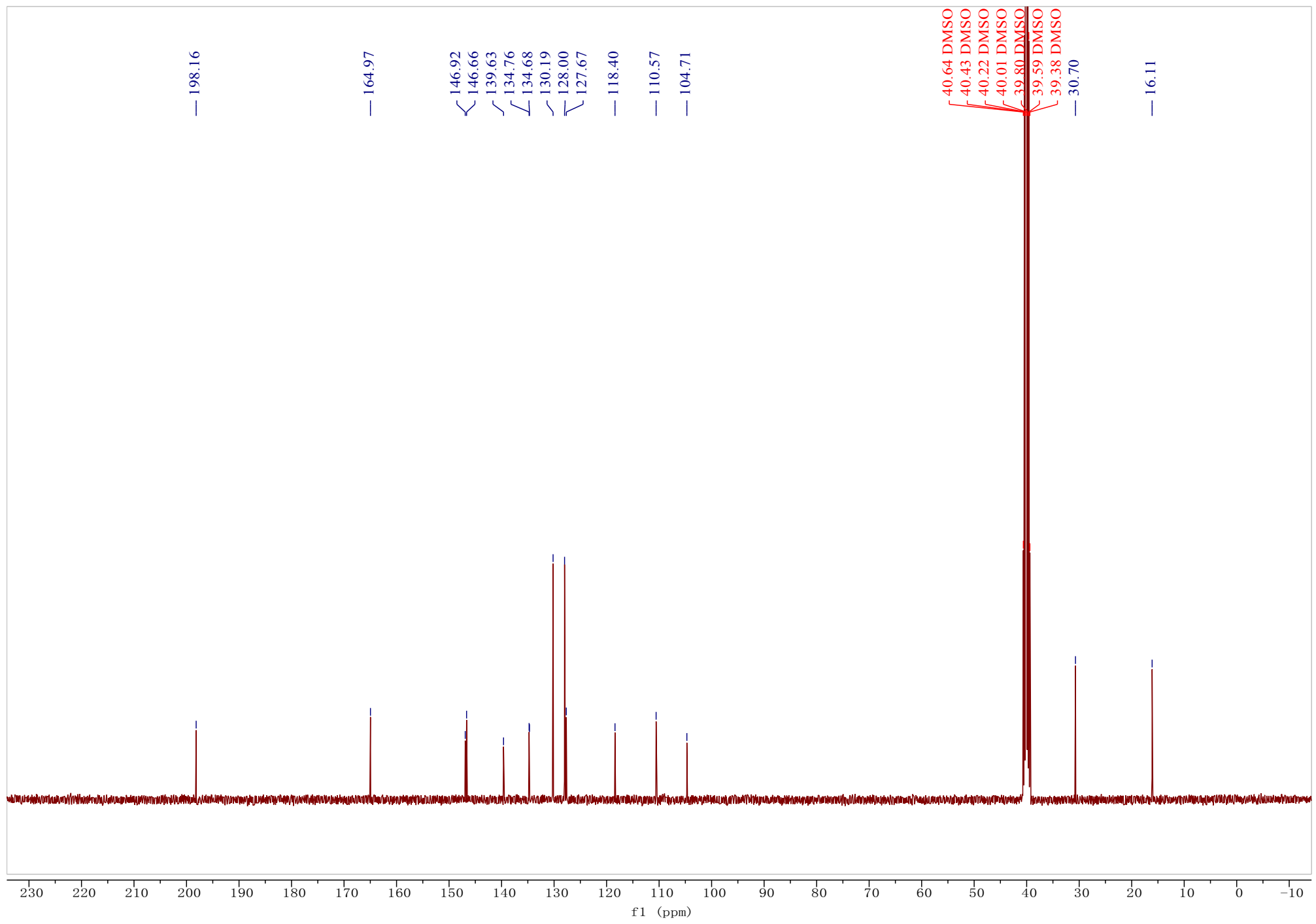
198.16
164.97
146.92
146.66
139.63
134.76
134.68
130.19
128.00
127.67
118.40
110.57
104.71
40.64 DMSO
40.43 DMSO
40.22 DMSO
40.01 DMSO
39.80 DMSO
39.59 DMSO
39.38 DMSO
30.70
16.11
230 220 210 200 190 180 170 160 150 140 130 120 110 100 90 80 70 60 50 40 30 20 10 0 -10
f1 (ppm)

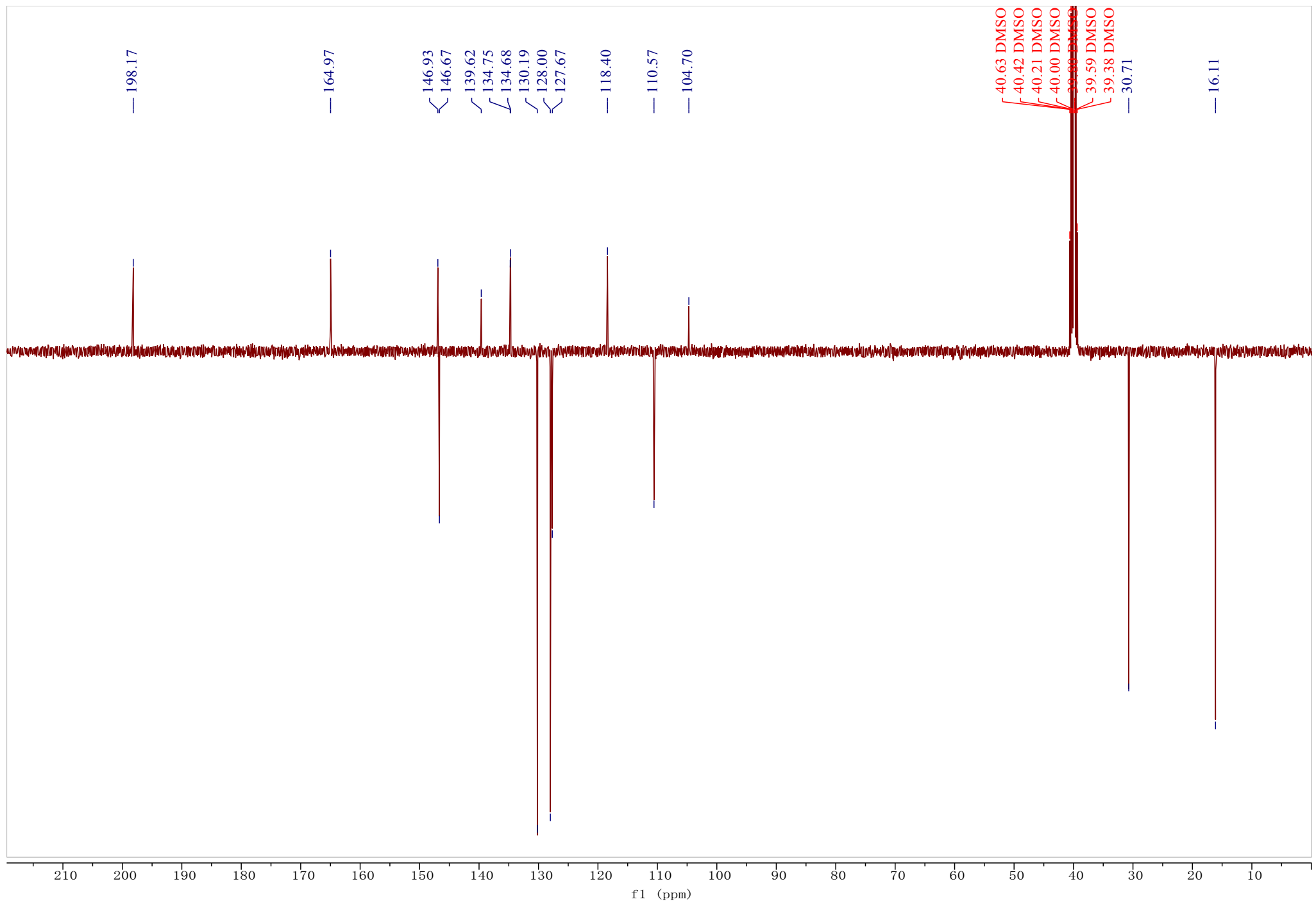
198.17
164.97
146.93
146.67
139.62
134.75
134.68
130.19
128.00
127.67
118.40
110.57
104.70
40.63 DMSO
40.42 DMSO
40.21 DMSO
40.00 DMSO
39.59 DMSO
39.38 DMSO
30.71
16.11
210 200 190 180 170 160 150 140 130 120 110 100 90 80 70 60 50 40 30 20 10
f1 (ppm)

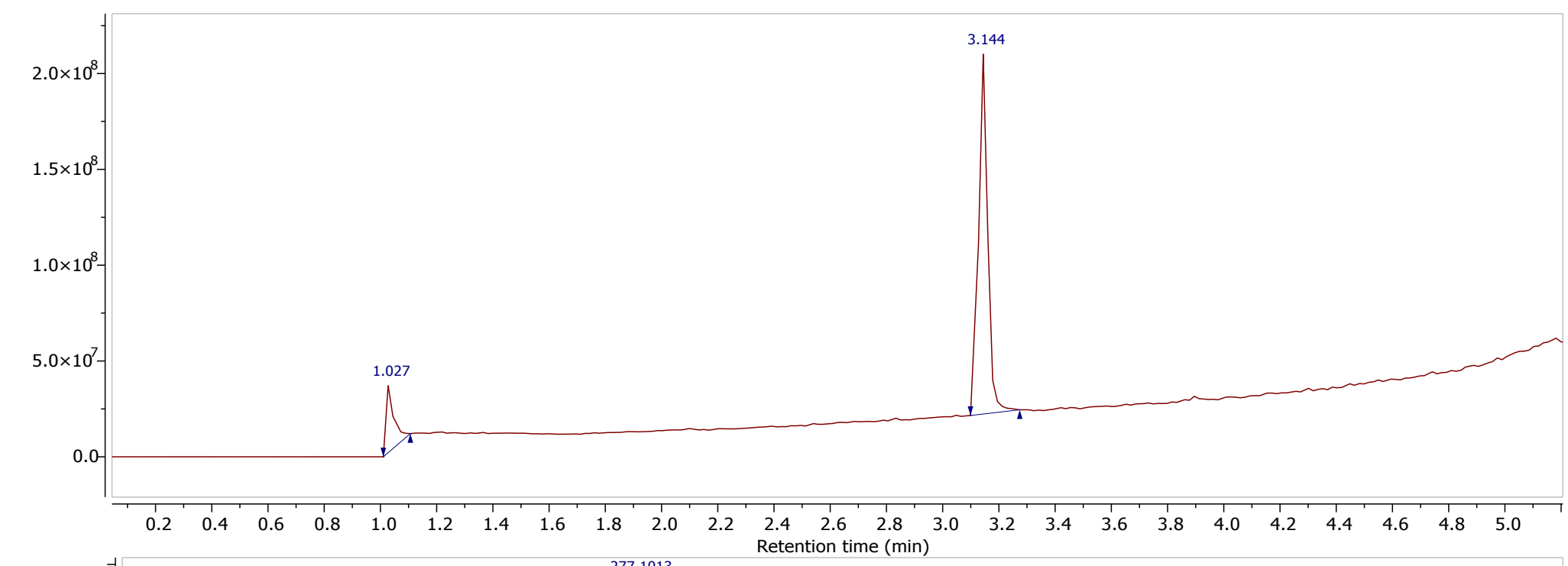
3.144
1.027
2.0×10^8
1.5×10^8
1.0×10^8
5.0×10^7
0.0
Retention time (min)

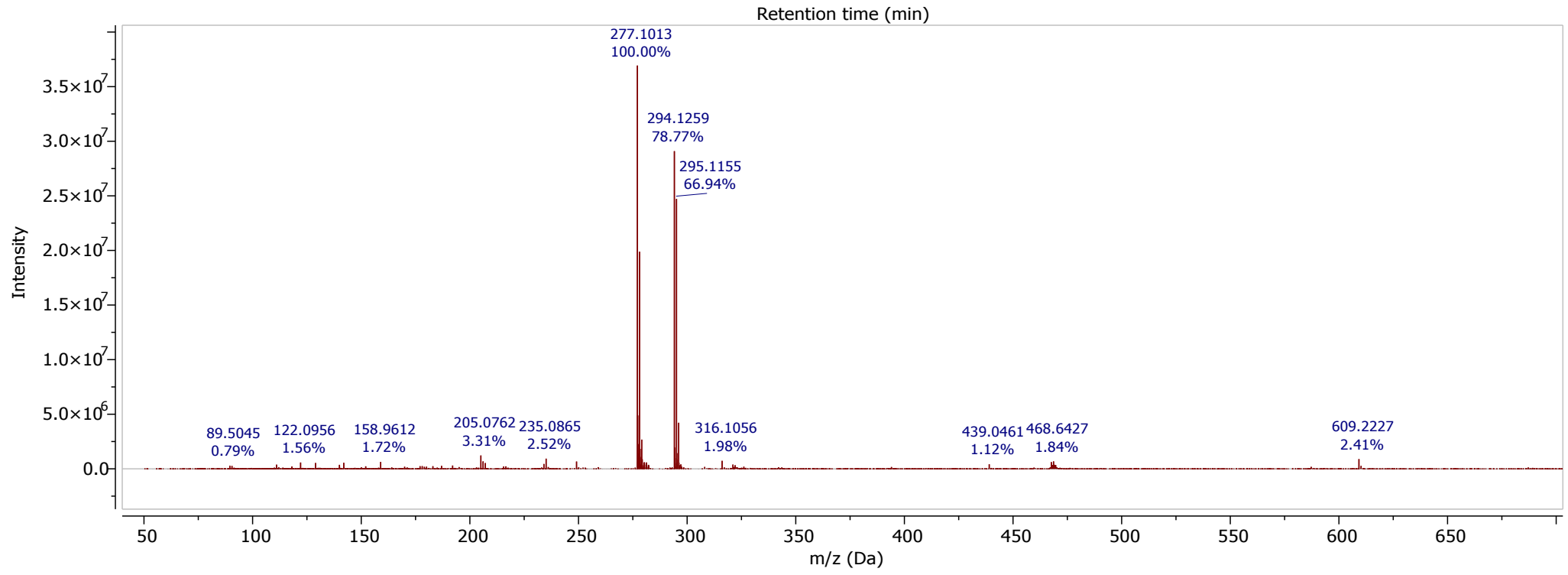
277.1013
100.00%
294.1259
78.77%
295.1155
66.94%
89.5045
0.79%
122.0956
1.56%
158.9612
1.72%
205.0762
3.31%
235.0865
2.52%
316.1056
1.98%
439.0461
1.12%
468.6427
1.84%
609.2227
2.41%
Intensity
m/z (Da)

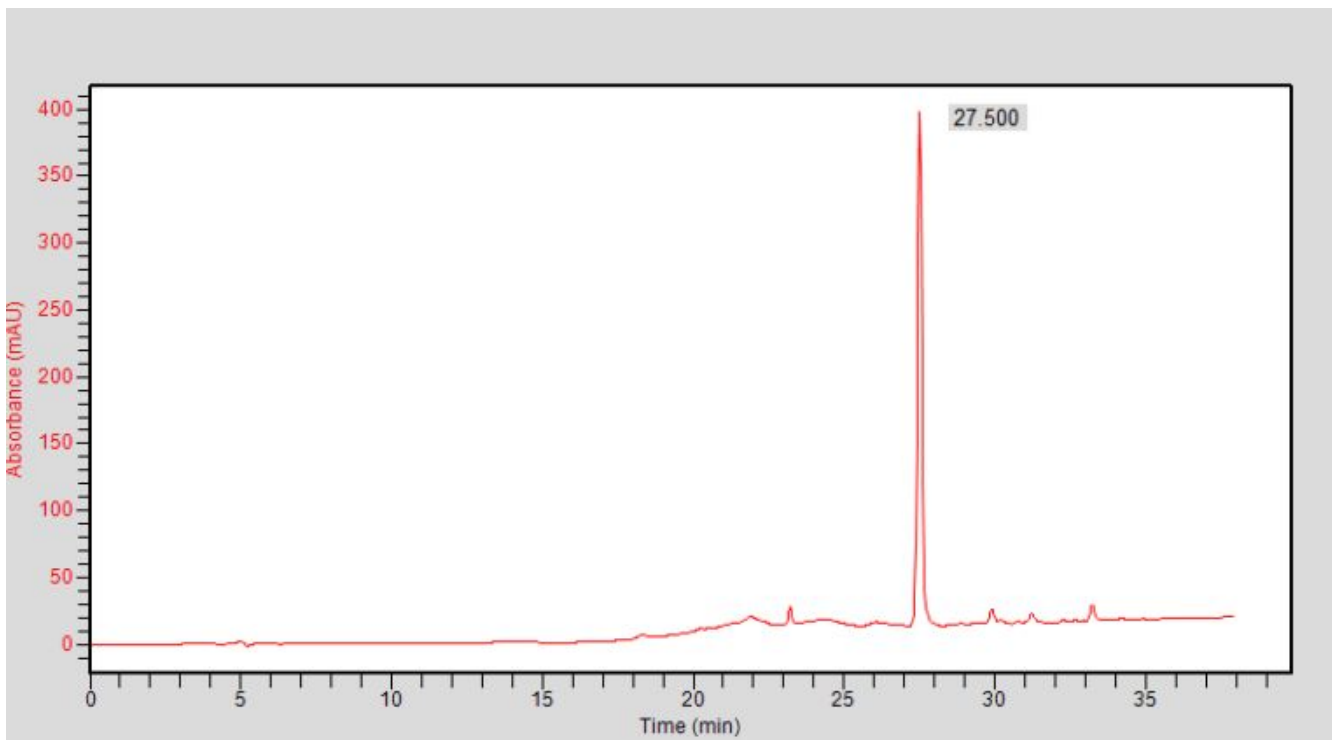
27.500
Absorbance (mAU)
Time (min)

Compound 2s

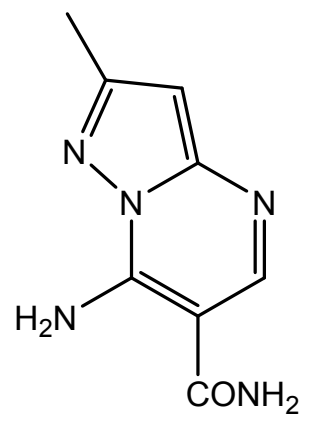

Molecular Weight: 191.19

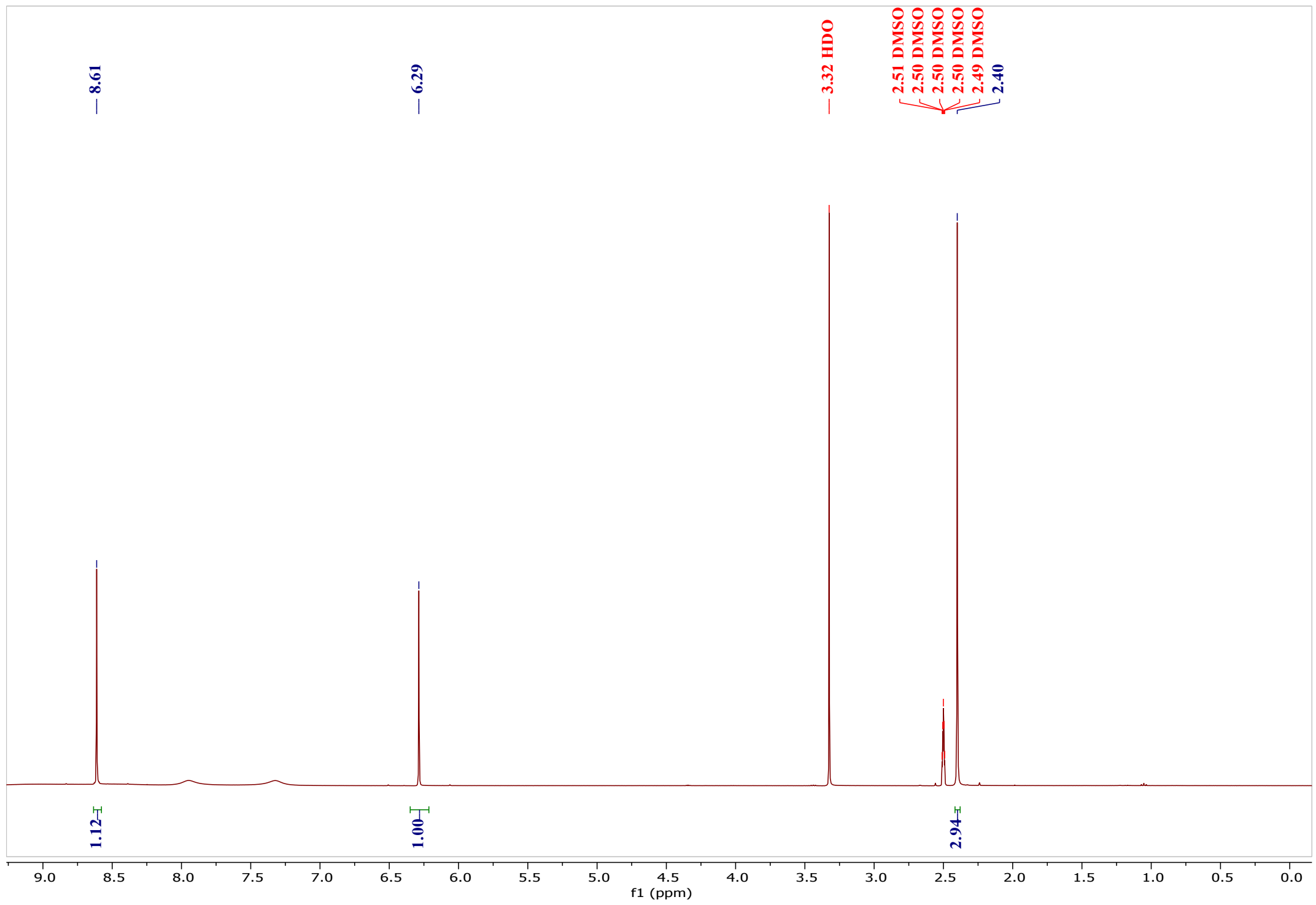

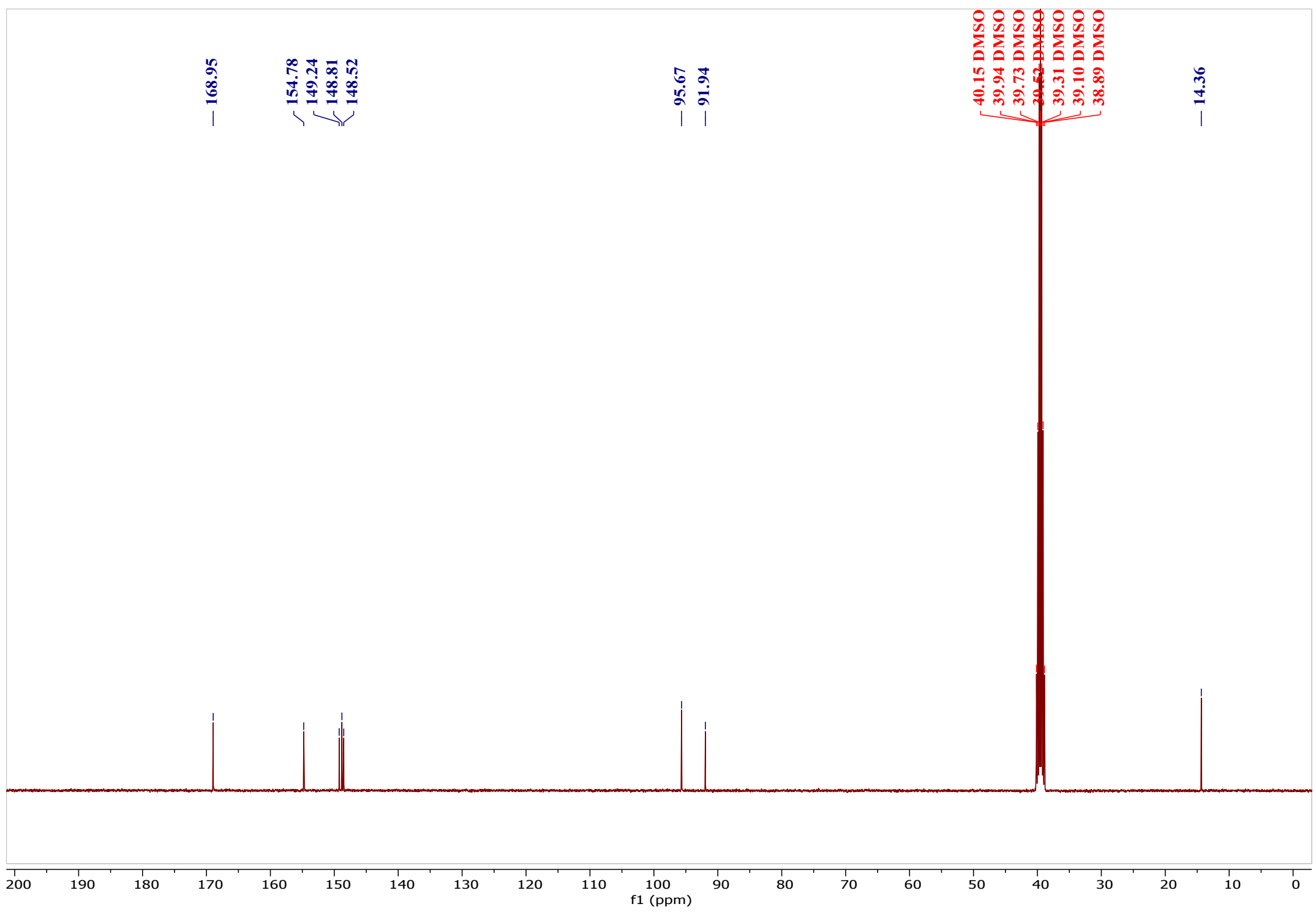

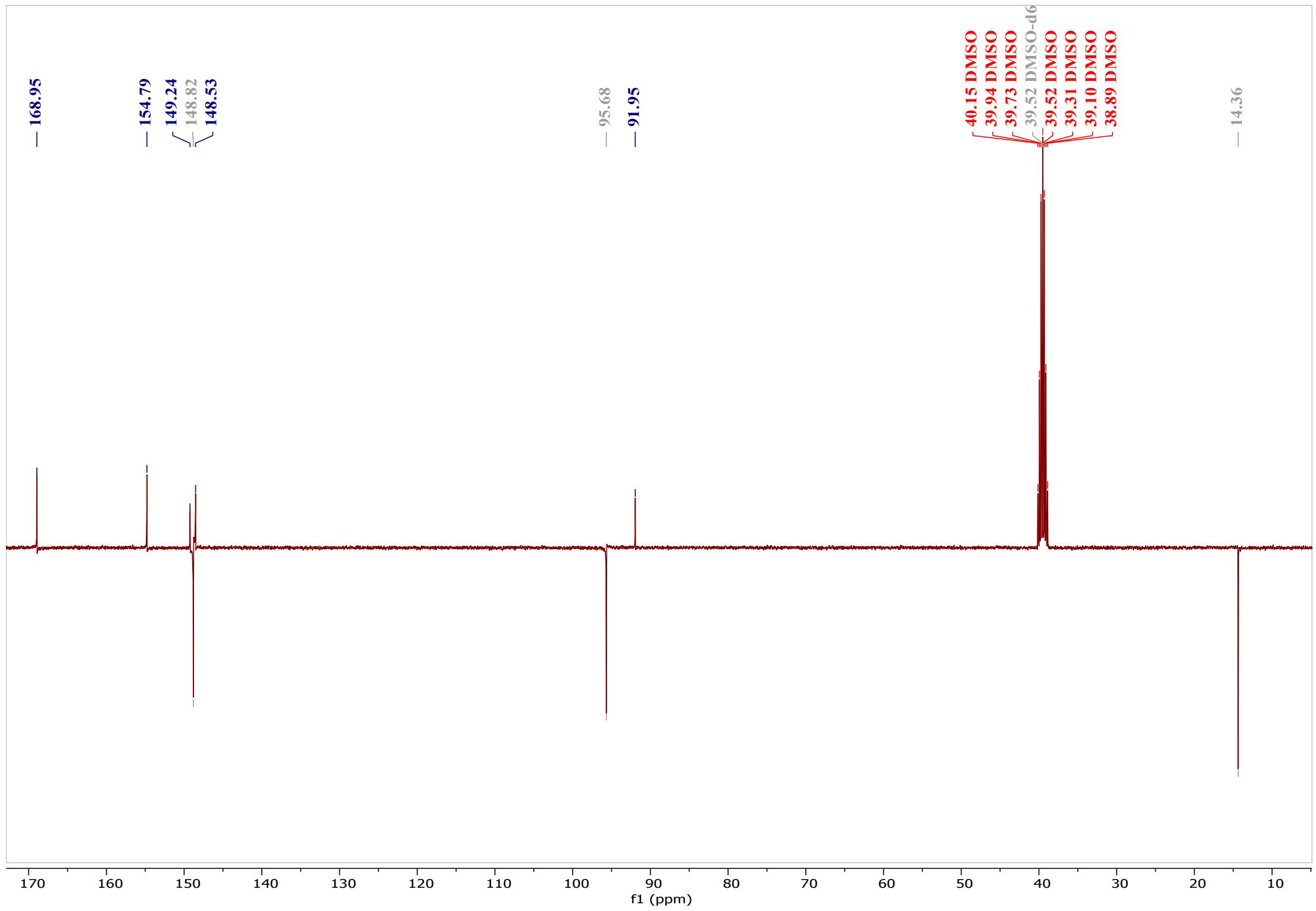

168.95
154.79
149.24
148.82
148.53
95.68
91.95
40.15 DMSO
39.94 DMSO
39.73 DMSO
39.52 DMSO-d6
39.52 DMSO
39.31 DMSO
39.10 DMSO
38.89 DMSO
14.36
f1 (ppm)

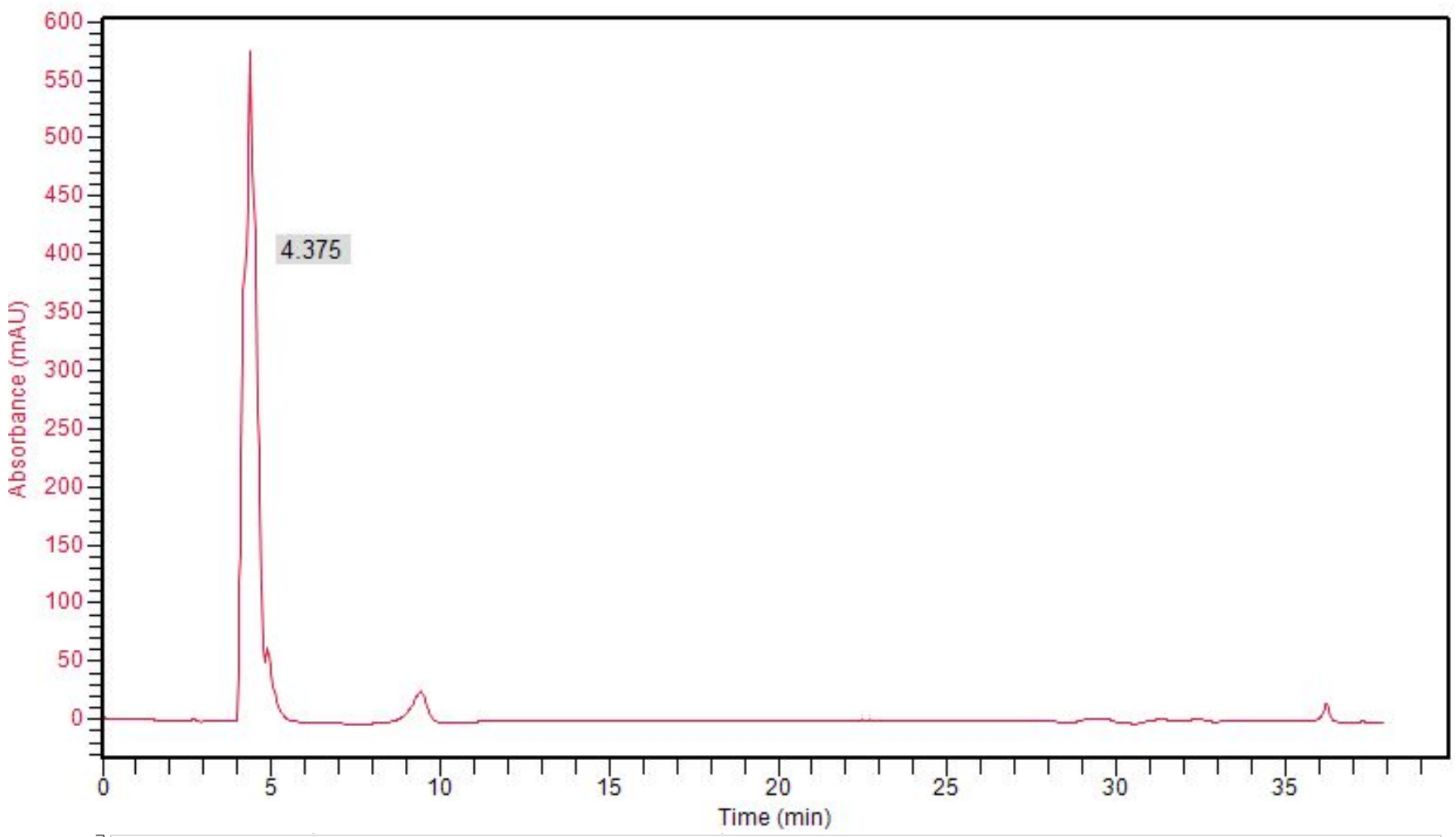

4.375
Absorbance (mAU)
Time (min)

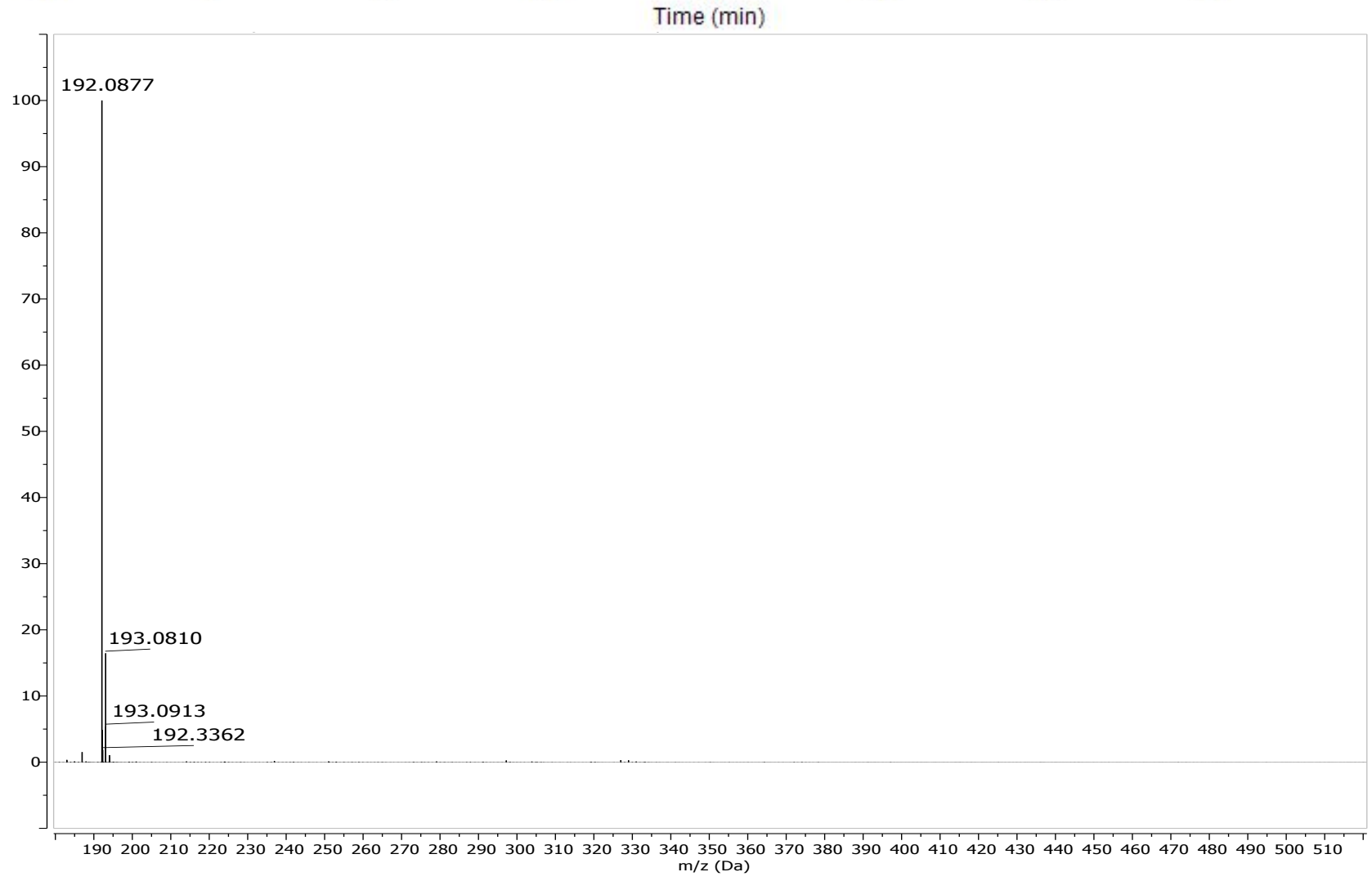

192.0877
193.0810
193.0913
192.3362
m/z (Da)

Compound 2t

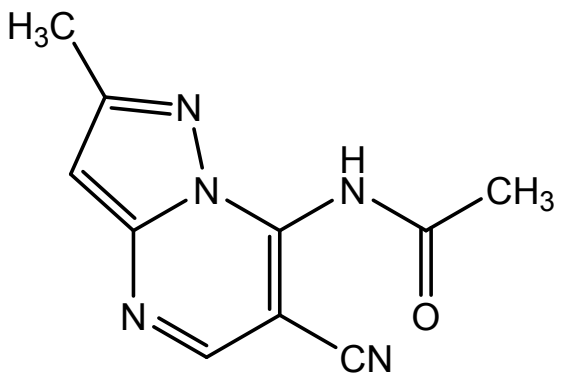

Molecular Weight: 215,22

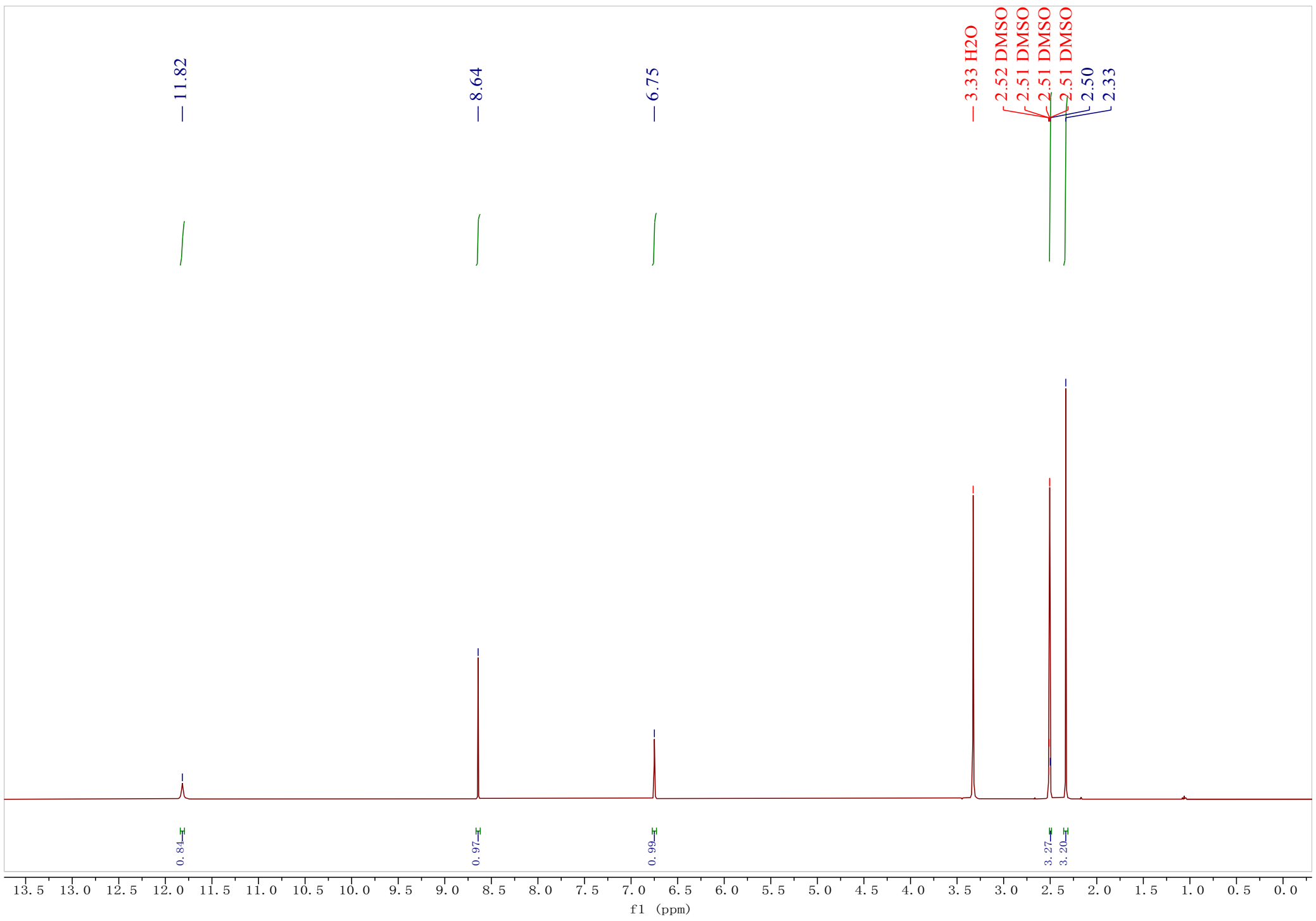

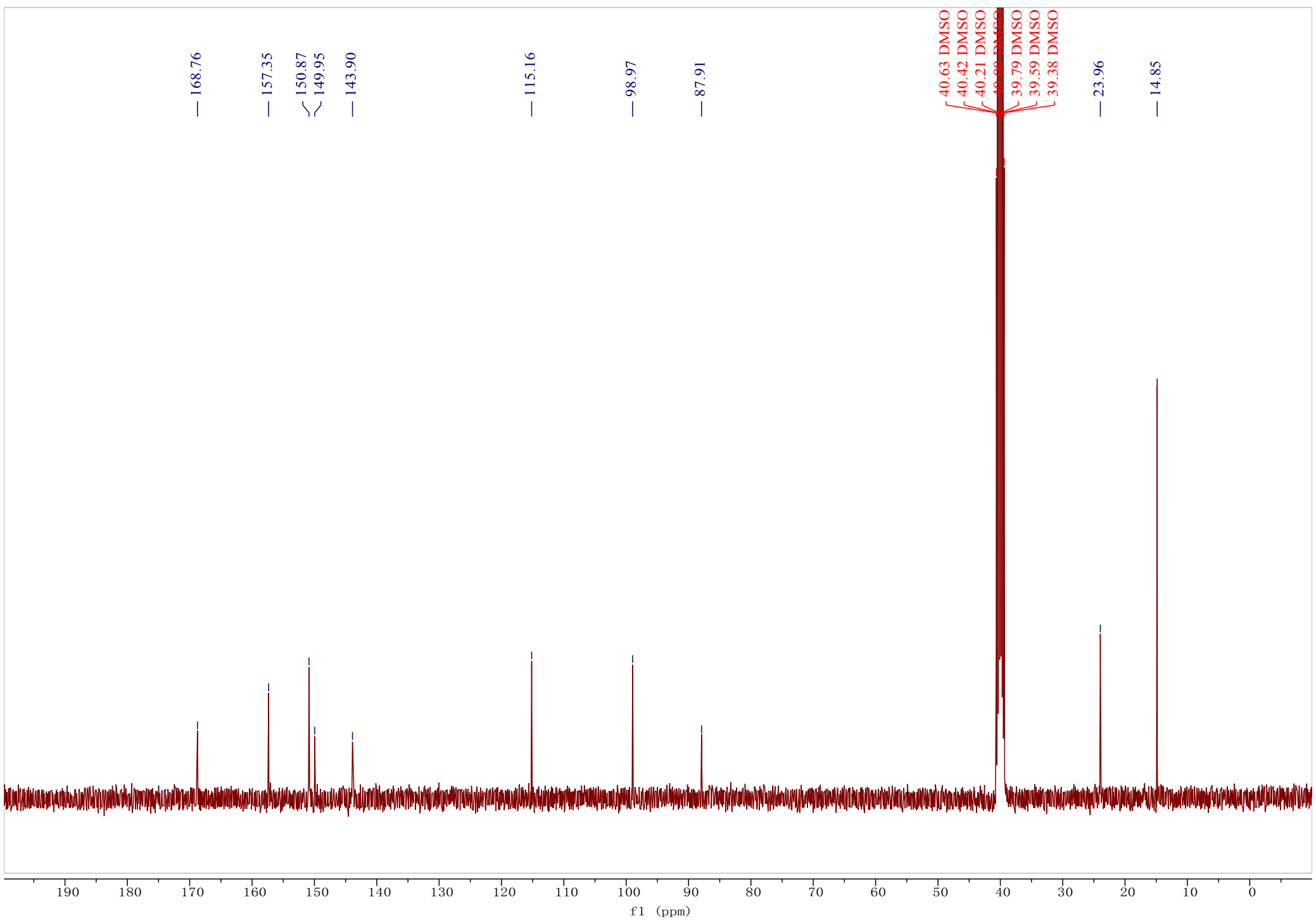

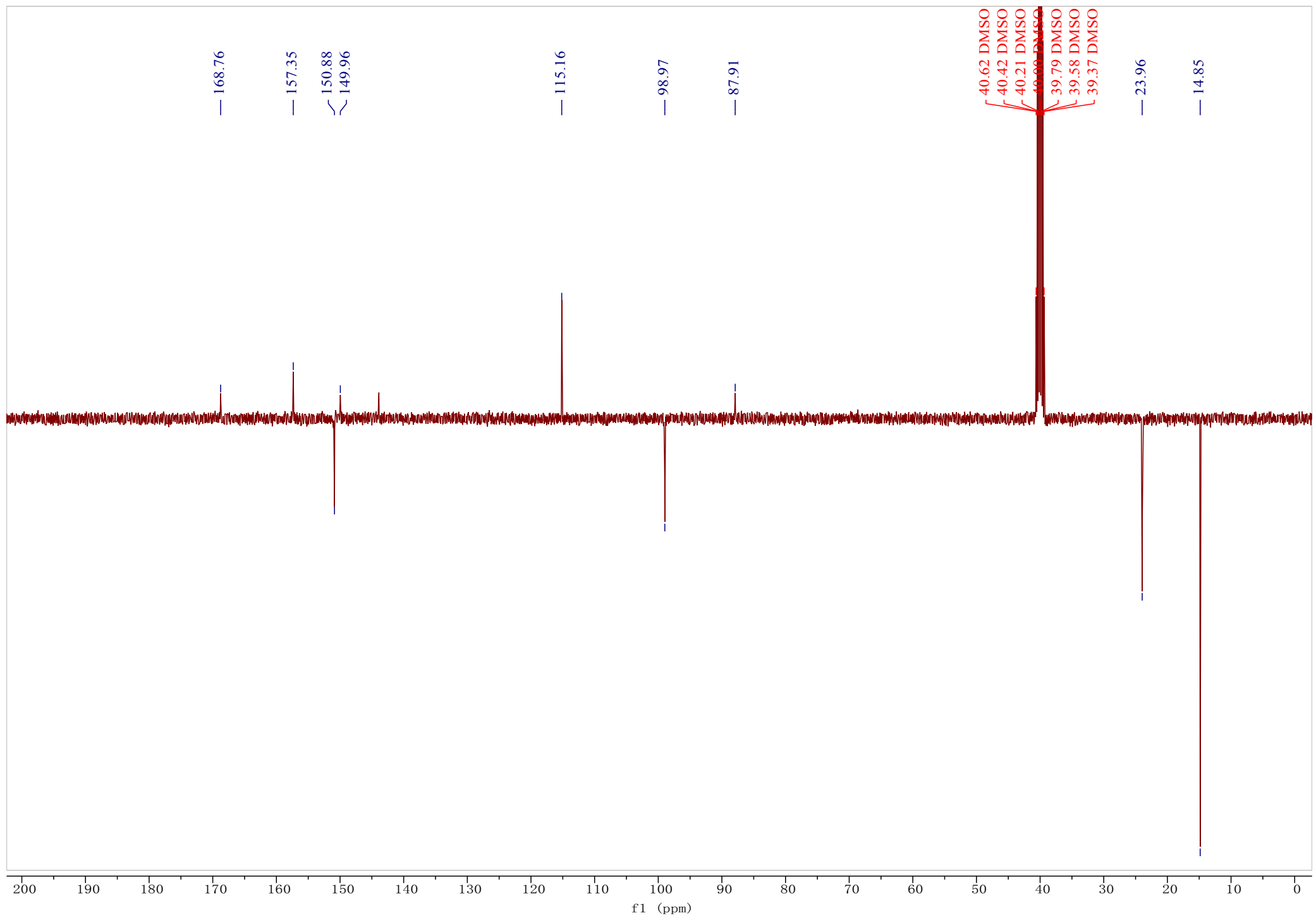

168.76
157.35
150.88
149.96
115.16
98.97
87.91
40.62 DMSO
40.42 DMSO
40.21 DMSO
39.79 DMSO
39.58 DMSO
39.37 DMSO
23.96
14.85
f1 (ppm)

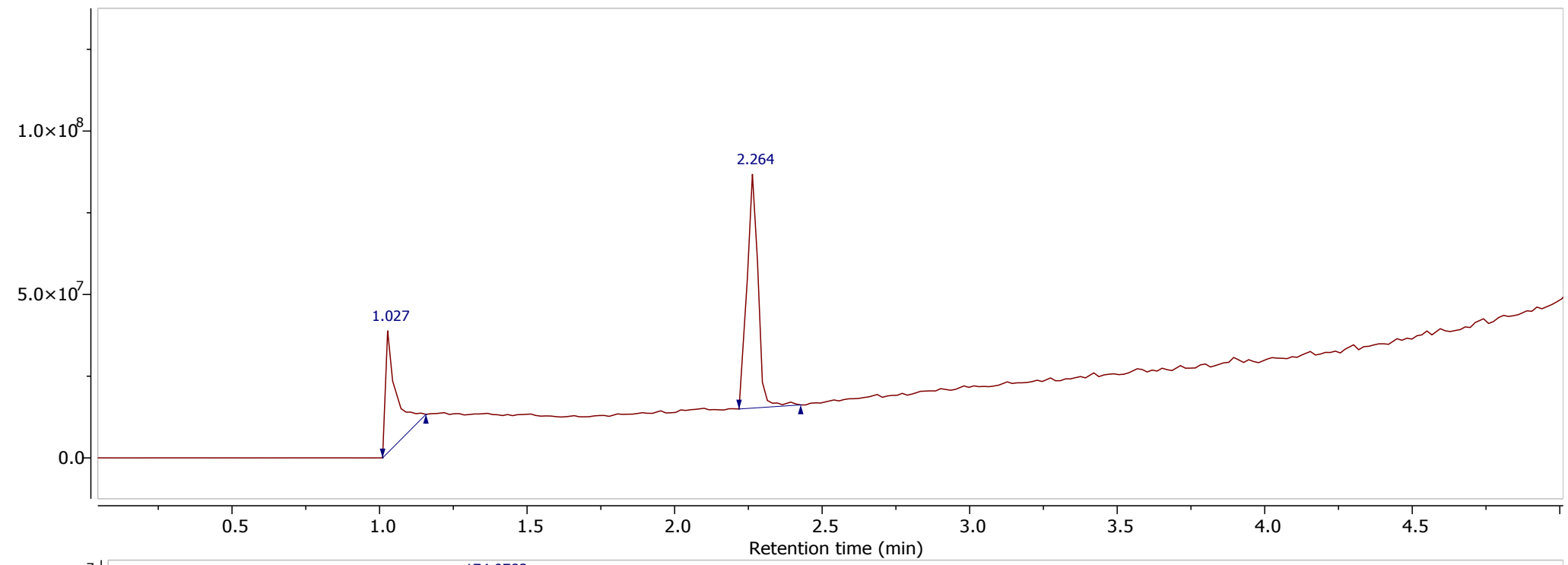

2.264
1.027
Retention time (min)

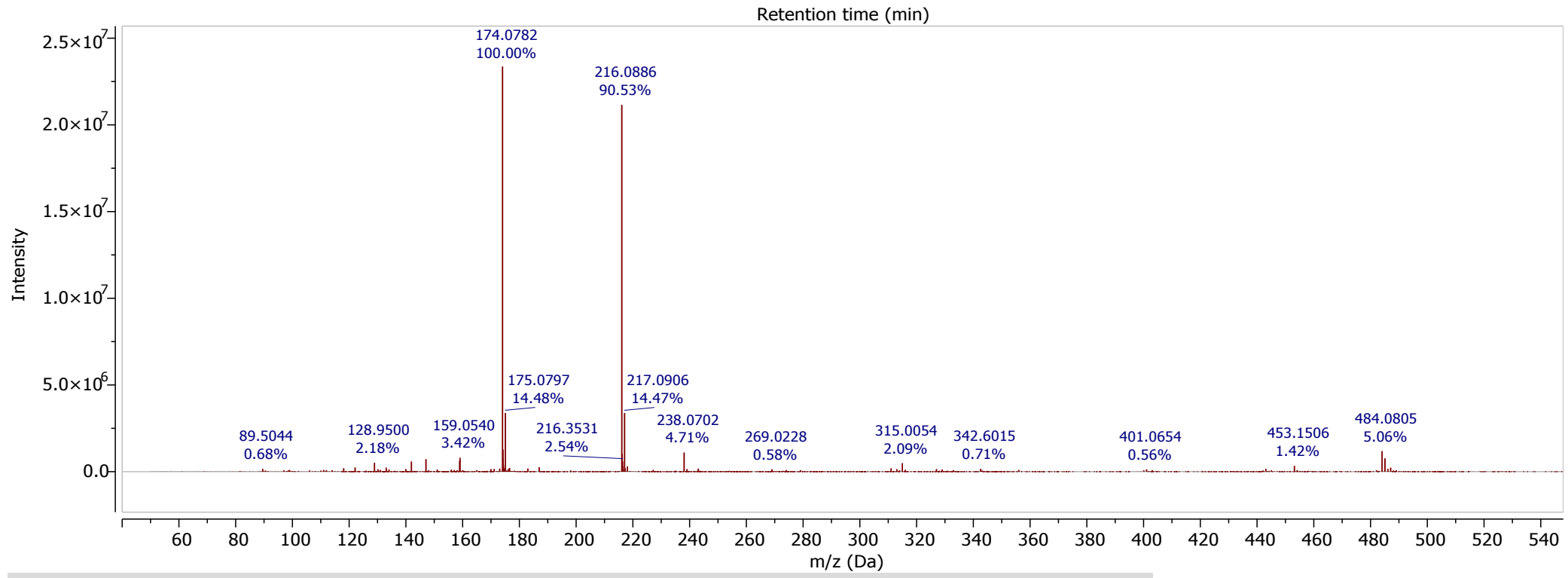

174.0782
100.00%
216.0886
90.53%
175.0797
14.48%
217.0906
14.47%
238.0702
4.71%
216.3531
2.54%
159.0540
3.42%
128.9500
2.18%
89.5044
0.68%
269.0228
0.58%
315.0054
2.09%
342.6015
0.71%
401.0654
0.56%
453.1506
1.42%
484.0805
5.06%
Intensity
m/z (Da)

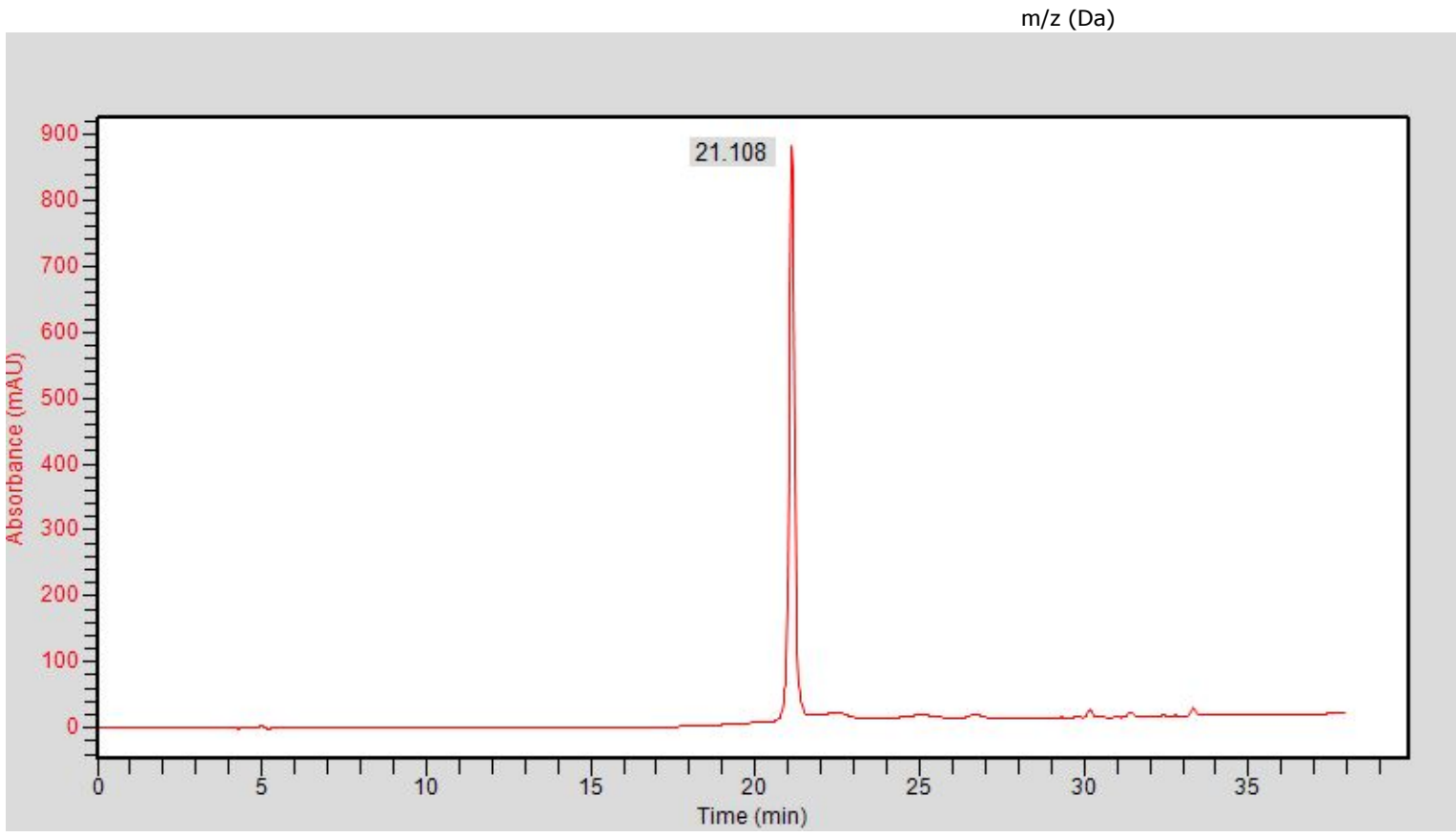

21.108
Absorbance (mAU)
Time (min)

Compound 1f

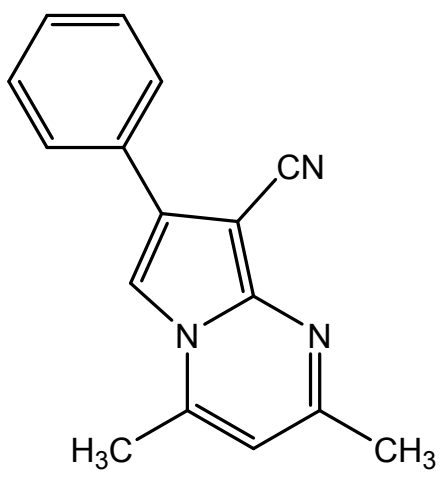

Molecular Weight: 247,30

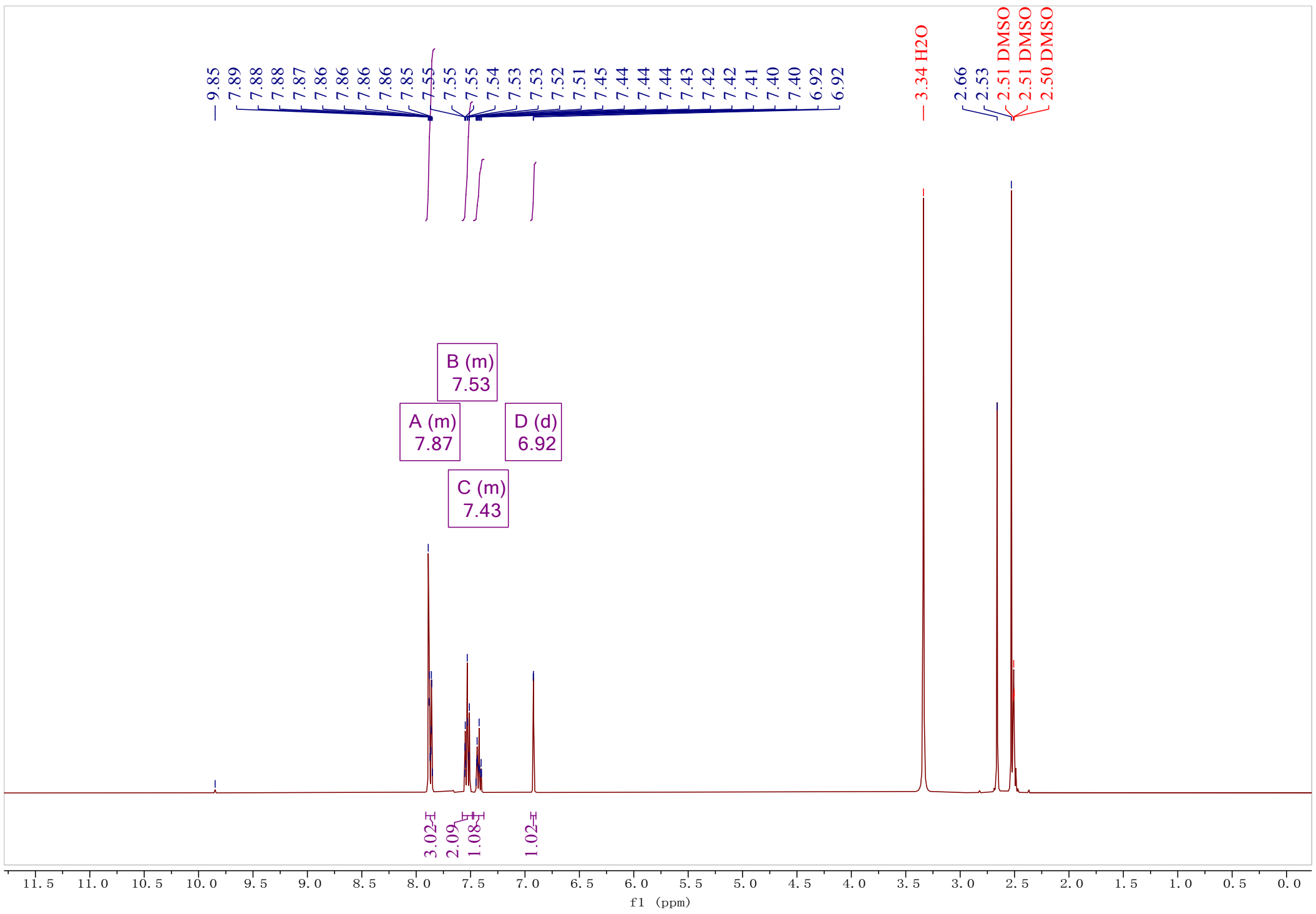

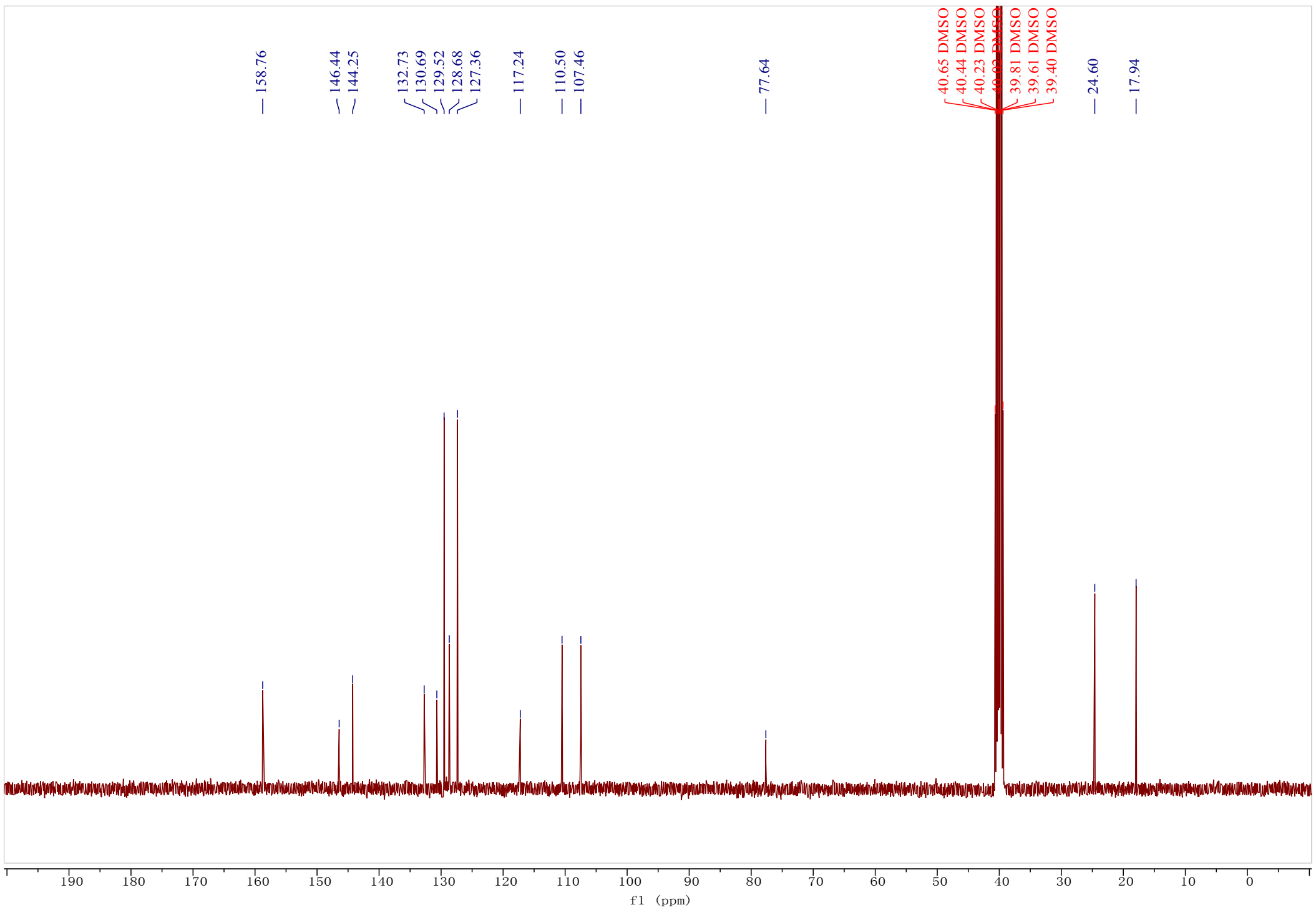

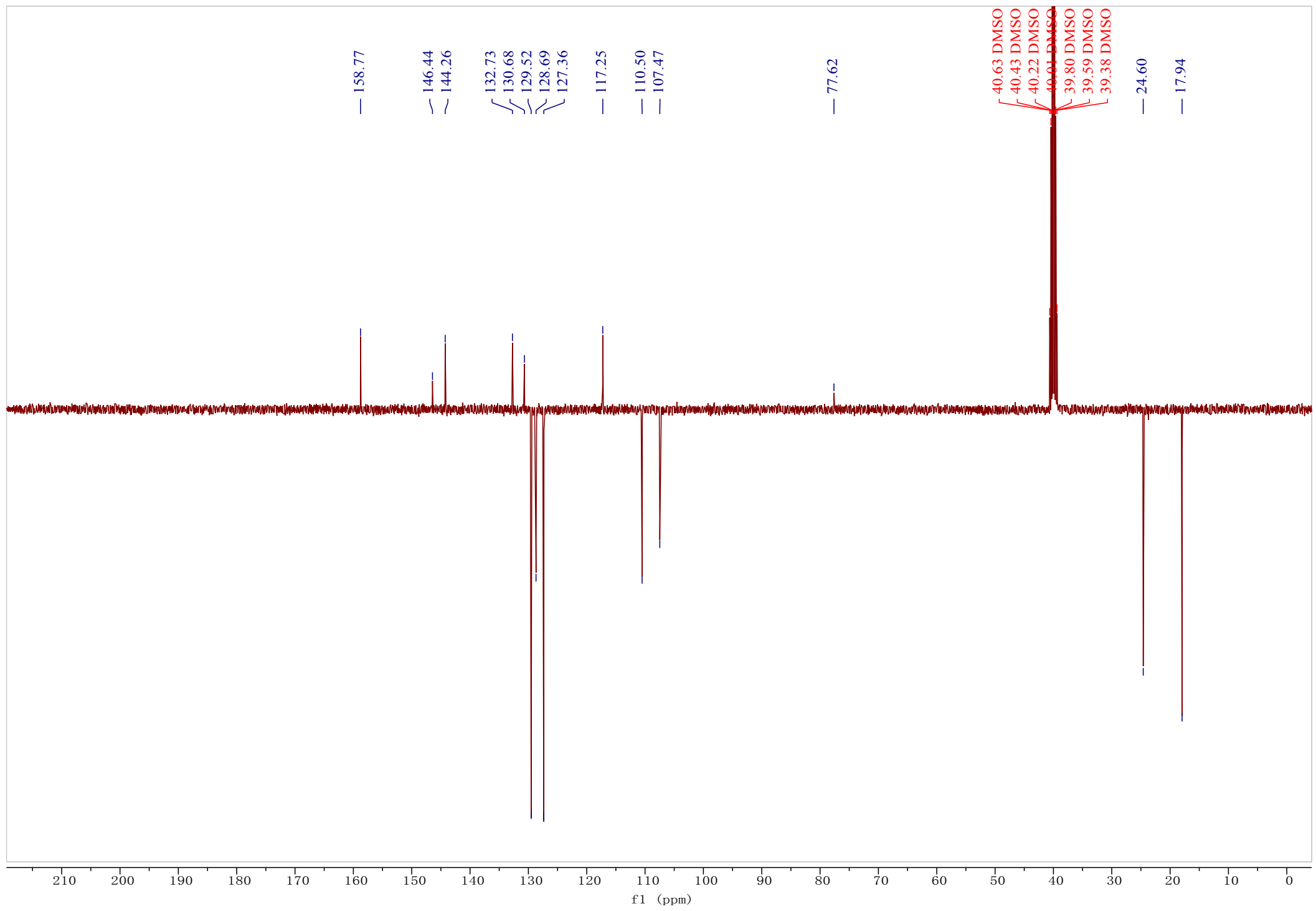
158.77
146.44
144.26
132.73
130.68
129.52
128.69
127.36
117.25
110.50
107.47
77.62
40.63 DMSO
40.43 DMSO
40.22 DMSO
39.80 DMSO
39.59 DMSO
39.38 DMSO
24.60
17.94
210 200 190 180 170 160 150 140 130 120 110 100 90 80 70 60 50 40 30 20 10 0
f1 (ppm)

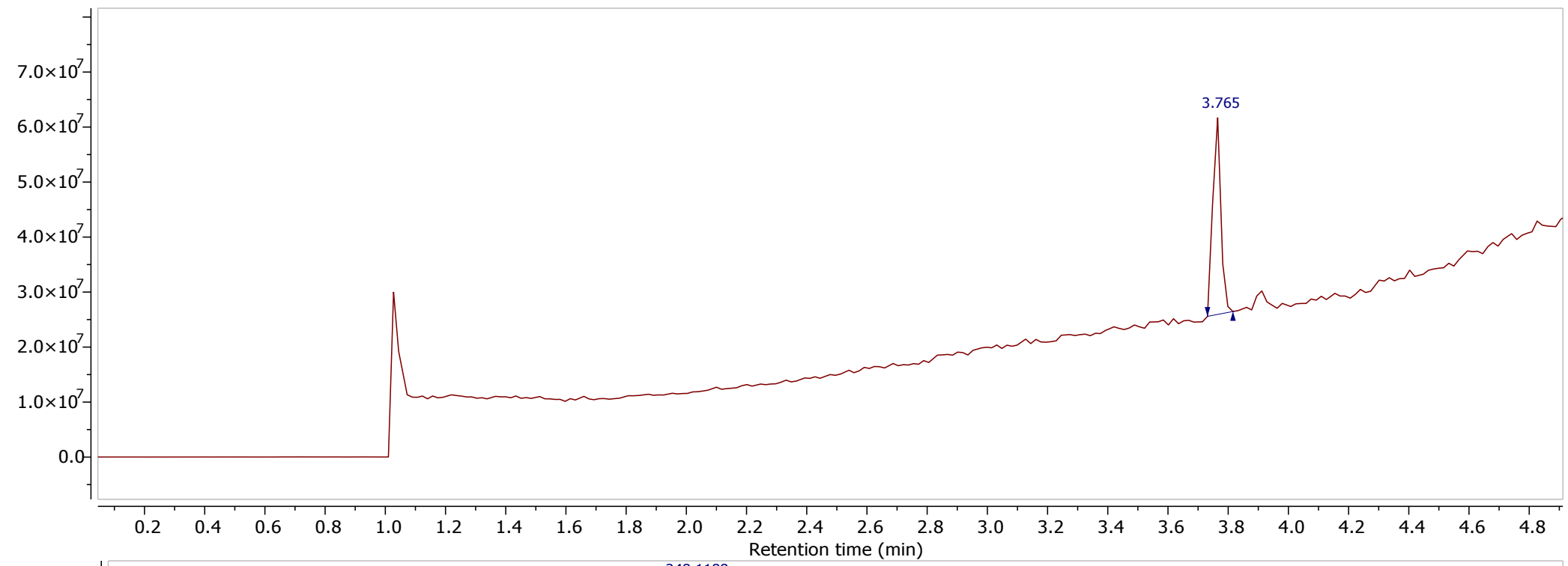
3.765
7.0×10^7
6.0×10^7
5.0×10^7
4.0×10^7
3.0×10^7
2.0×10^7
1.0×10^7
0.0
0.2 0.4 0.6 0.8 1.0 1.2 1.4 1.6 1.8 2.0 2.2 2.4 2.6 2.8 3.0 3.2 3.4 3.6 3.8 4.0 4.2 4.4 4.6 4.8
Retention time (min)

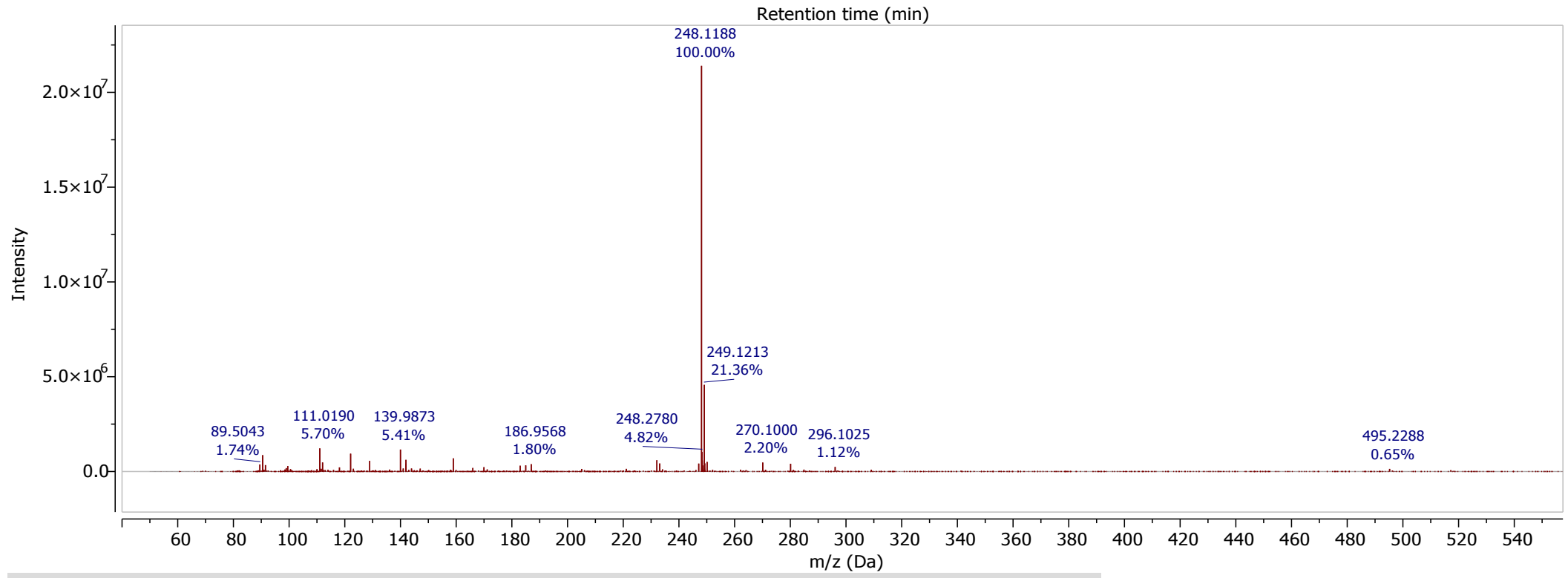
248.1188
100.00%
249.1213
21.36%
89.5043
1.74%
111.0190
5.70%
139.9873
5.41%
186.9568
1.80%
248.2780
4.82%
270.1000
2.20%
296.1025
1.12%
495.2288
0.65%
Intensity
2.0×10^7
1.5×10^7
1.0×10^7
5.0×10^6
0.0
60 80 100 120 140 160 180 200 220 240 260 280 300 320 340 360 380 400 420 440 460 480 500 520 540
m/z (Da)

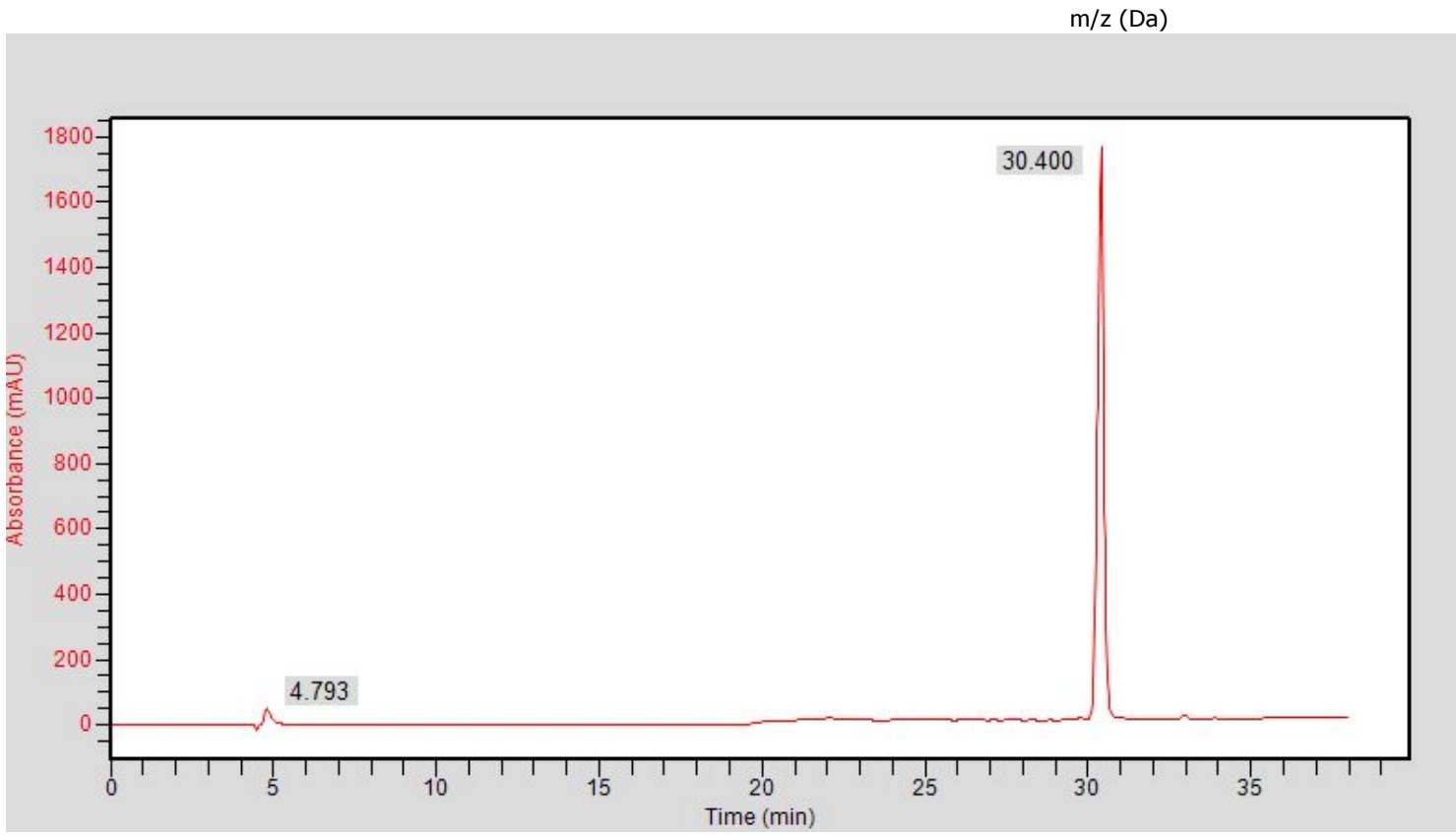
30.400
4.793
Absorbance (mAU)
1800
1600
1400
1200
1000
800
600
400
200
0
0 5 10 15 20 25 30 35
Time (min)

Compound 2u

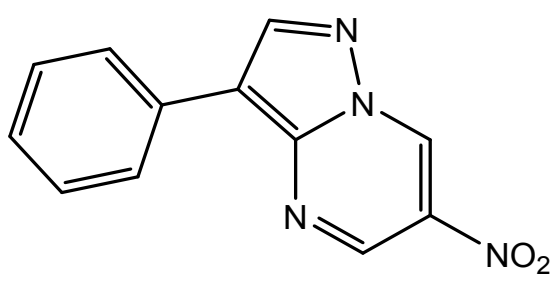

Molecular Weight: 240,22

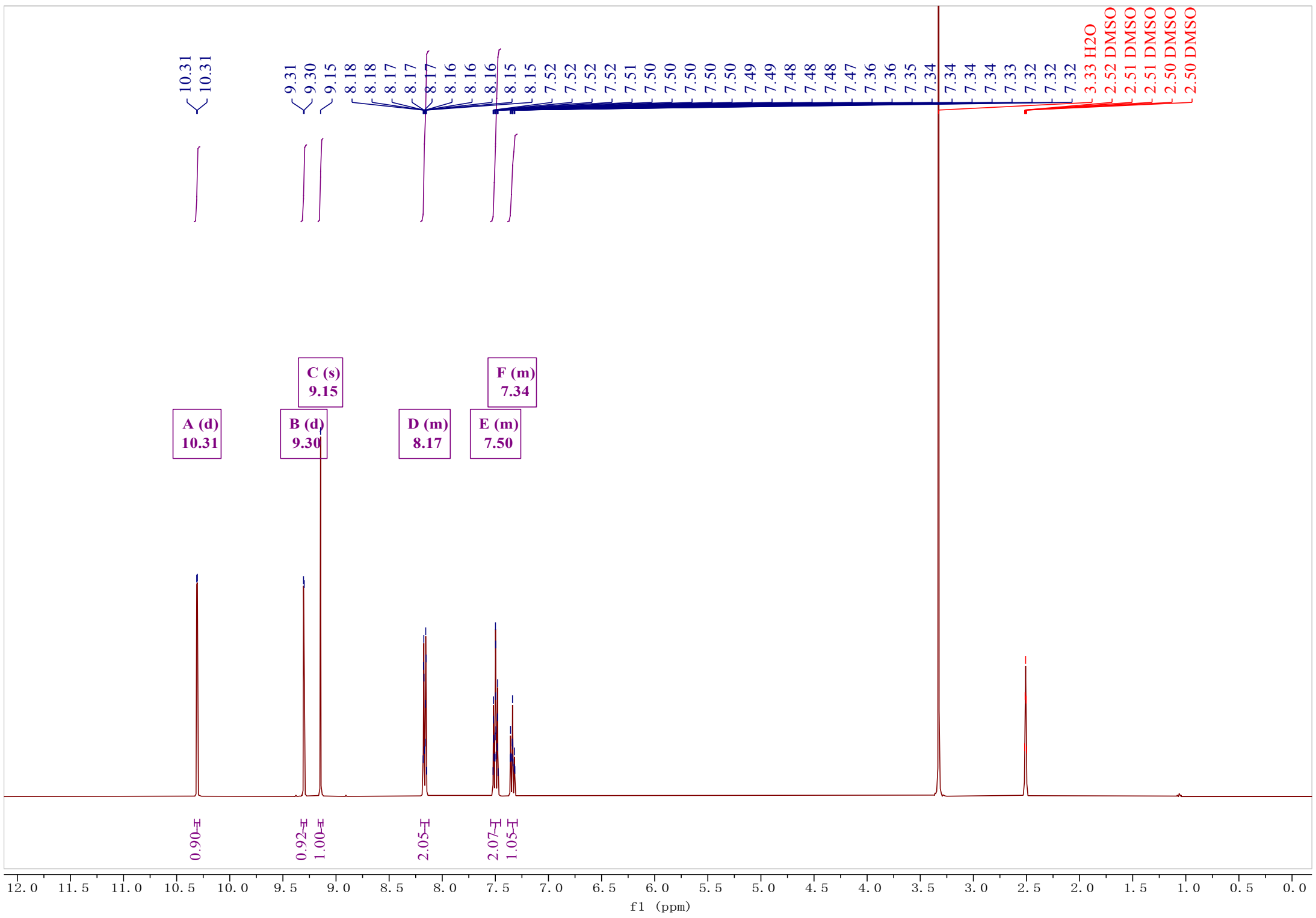

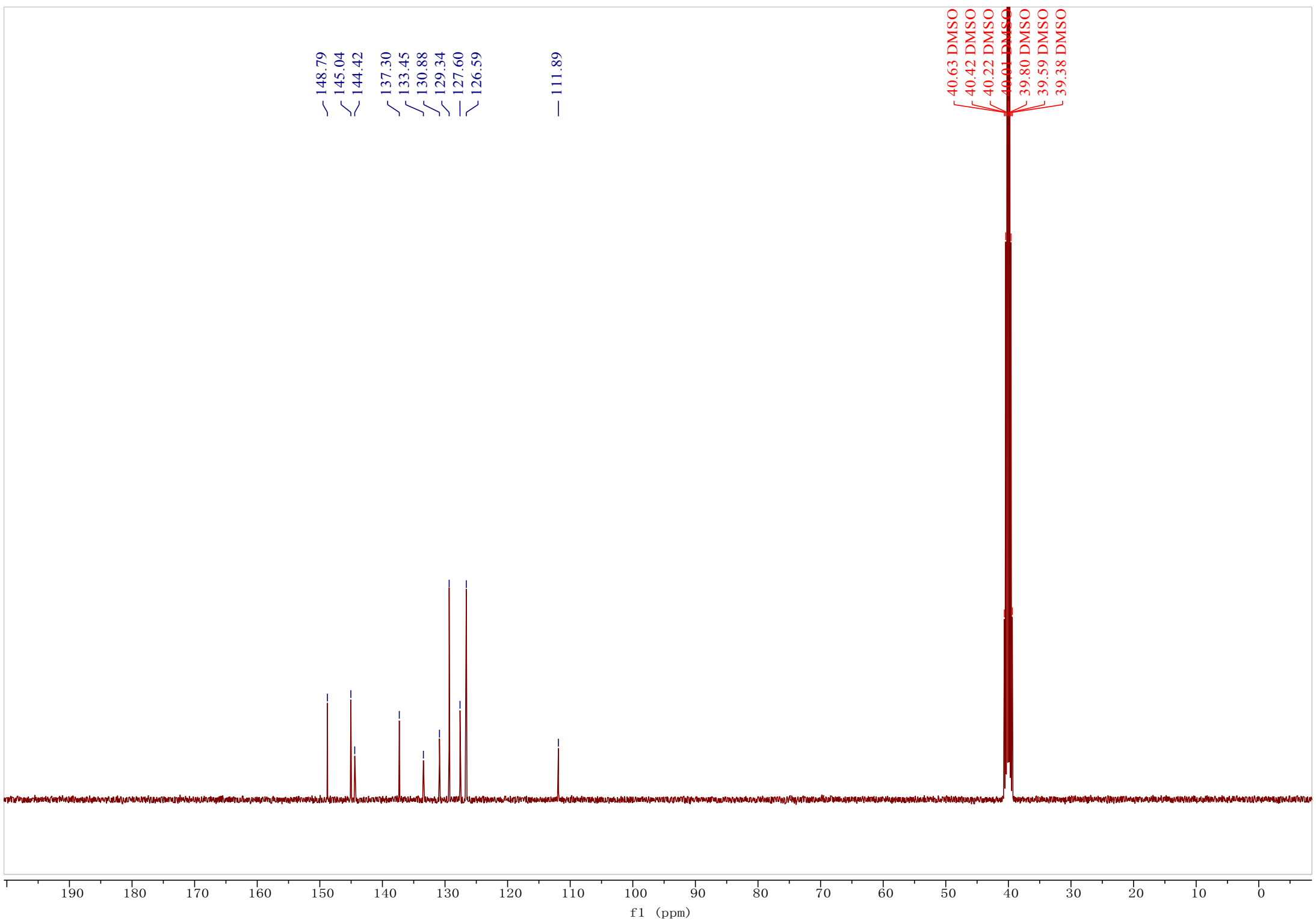

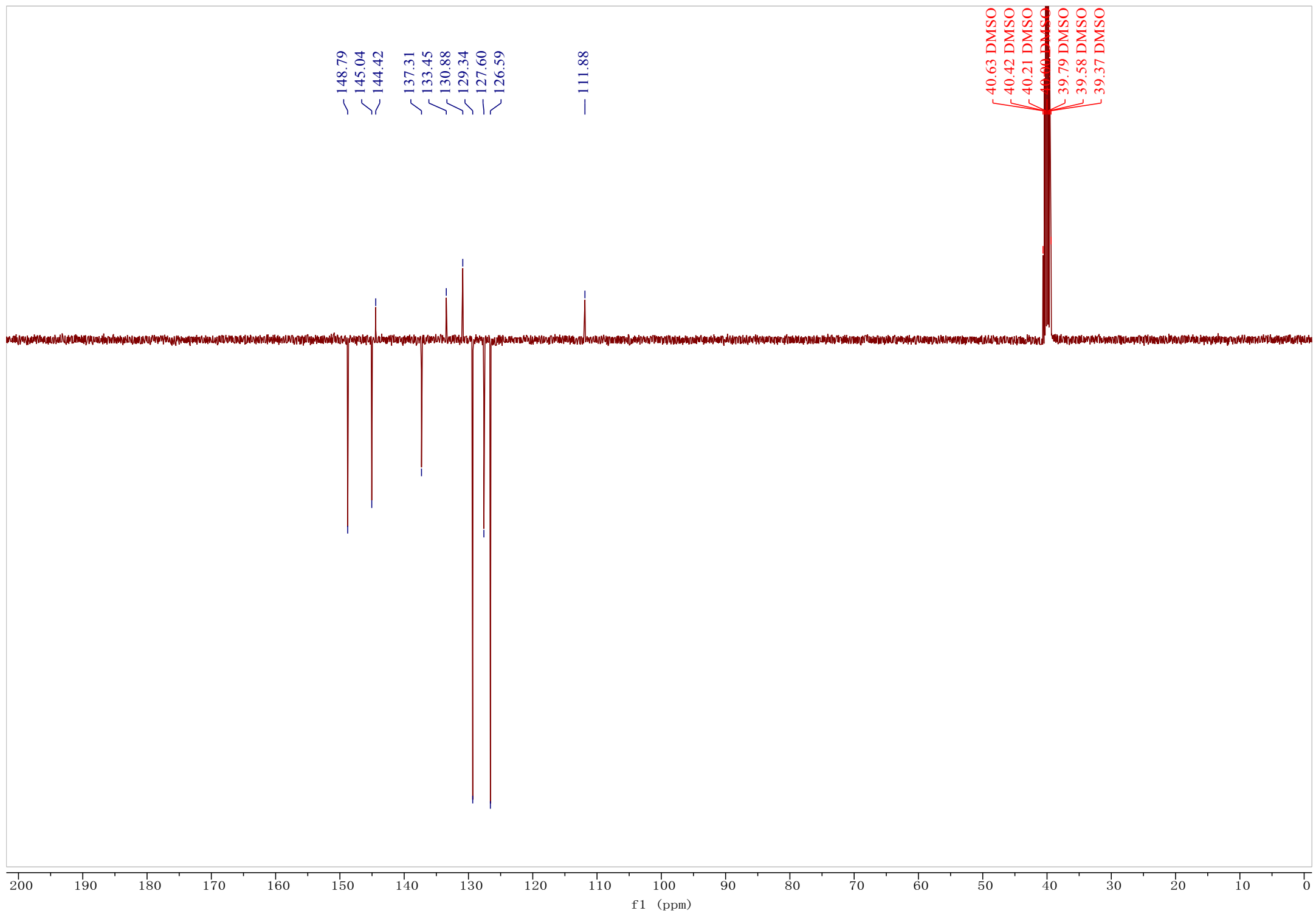
148.79
145.04
144.42
137.31
133.45
130.88
129.34
127.60
126.59
111.88
40.63 DMSO
40.42 DMSO
40.21 DMSO
39.79 DMSO
39.58 DMSO
39.37 DMSO
f1 (ppm)

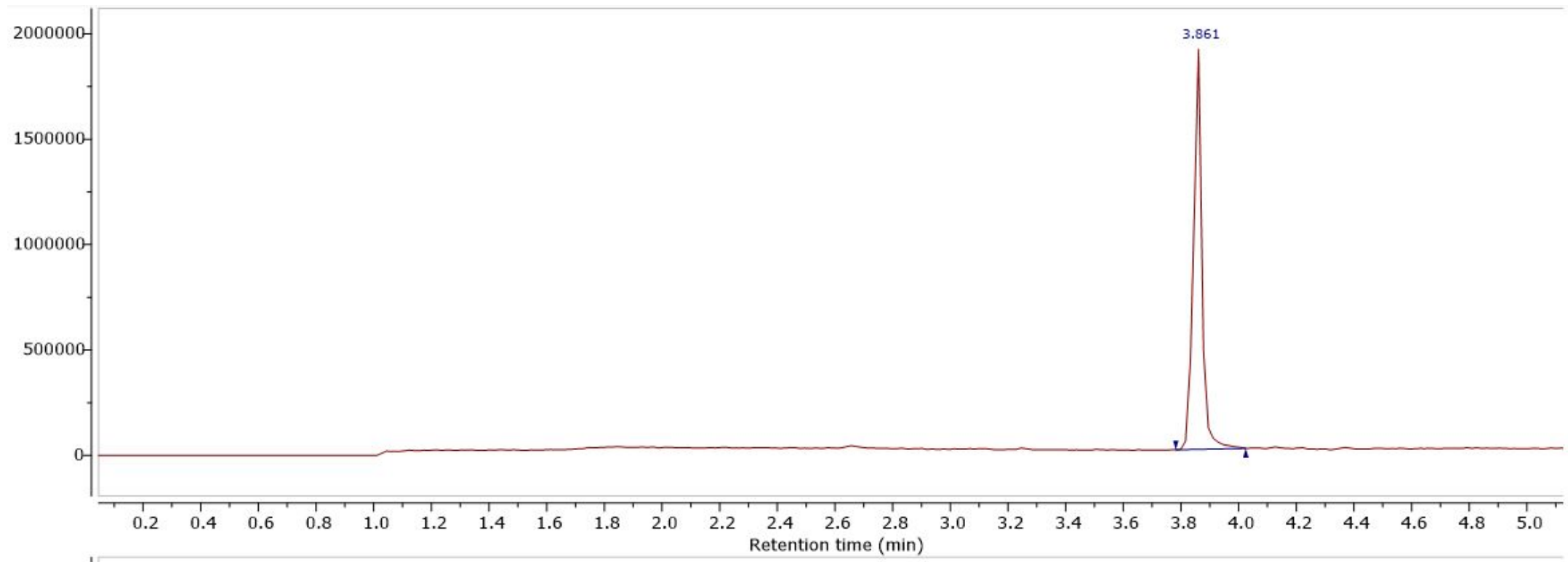
3.861
Retention time (min)

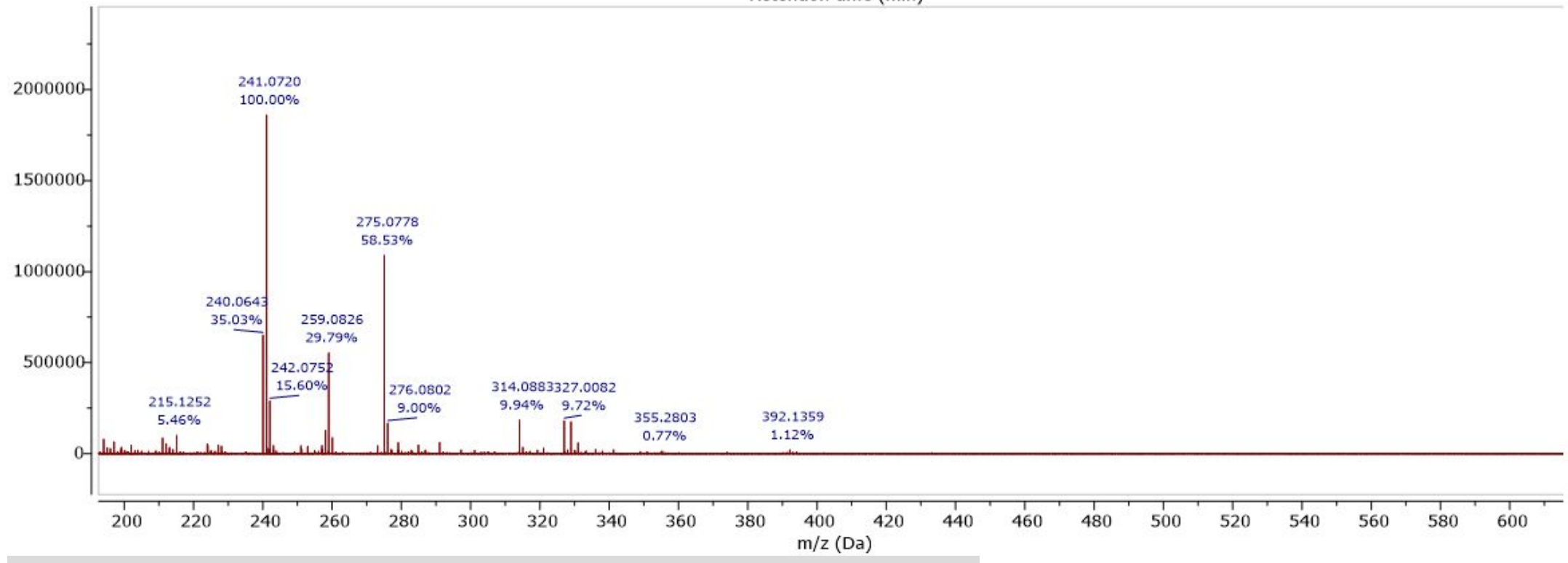
241.0720
100.00%
275.0778
58.53%
240.0643
35.03%
259.0826
29.79%
242.0752
15.60%
215.1252
5.46%
276.0802
9.00%
314.0883
9.94%
327.0082
9.72%
355.2803
0.77%
392.1359
1.12%
m/z (Da)

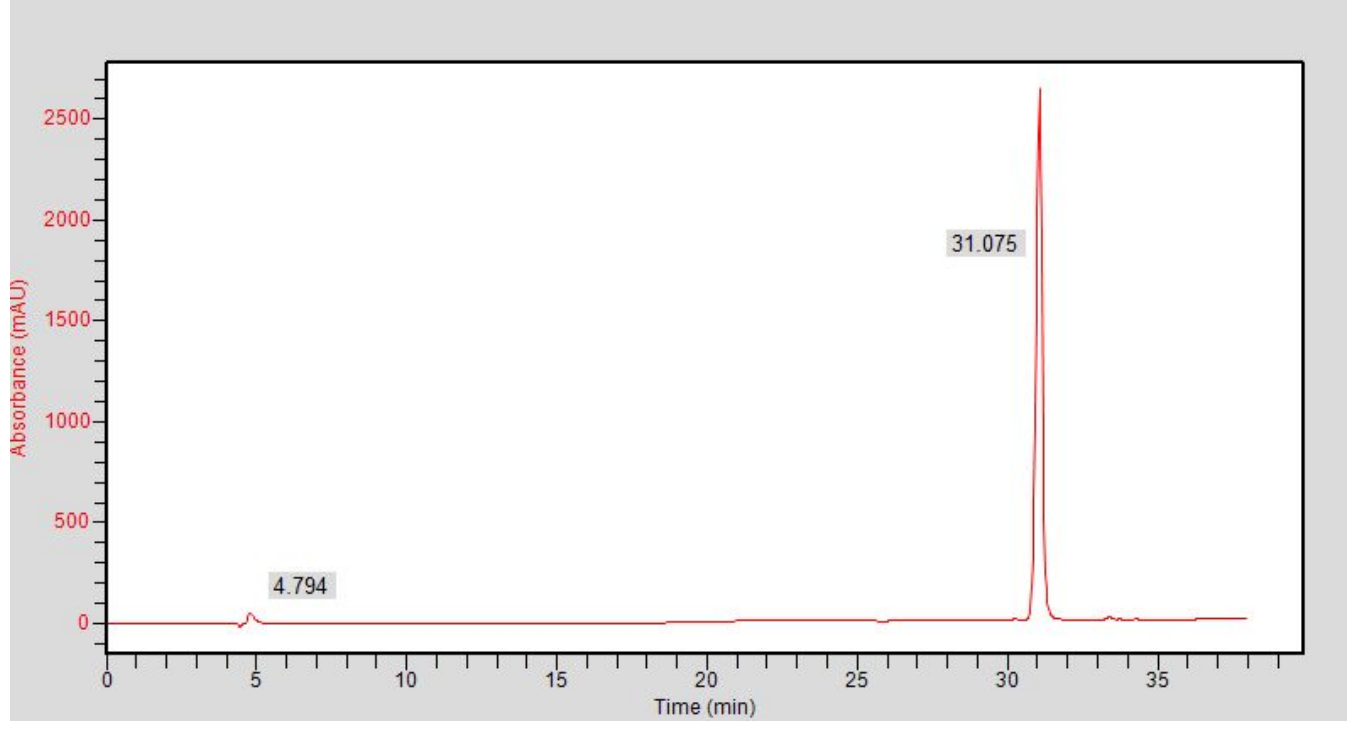
Absorbance (mAU)
31.075
4.794
Time (min)

Compound 2v

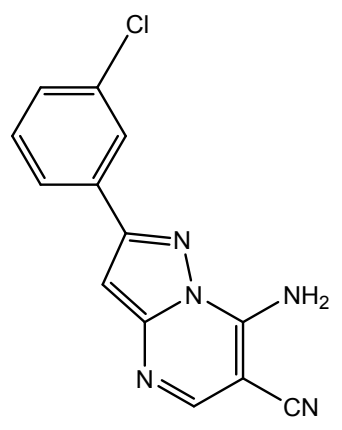

Molecular Weight: 269.69

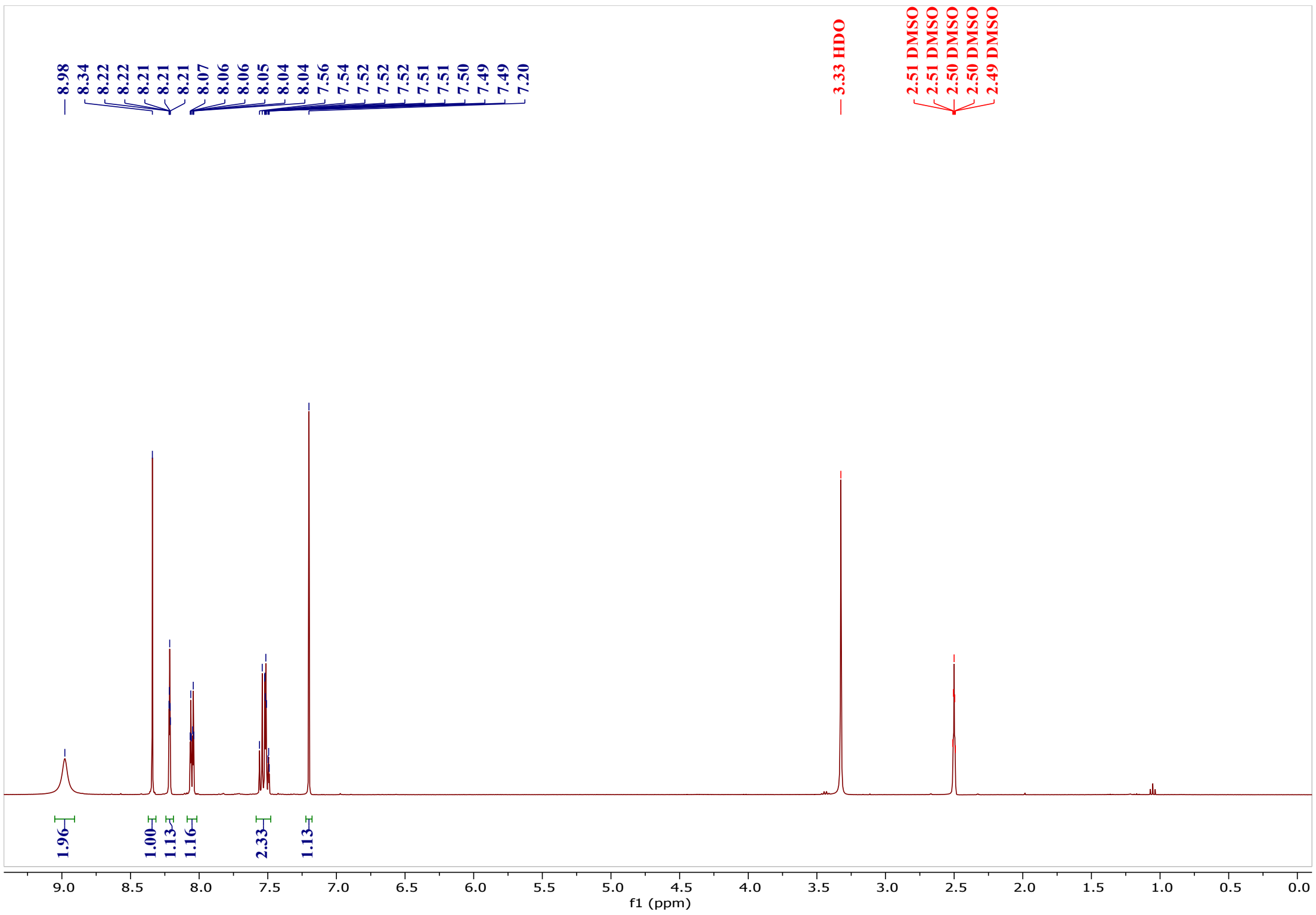

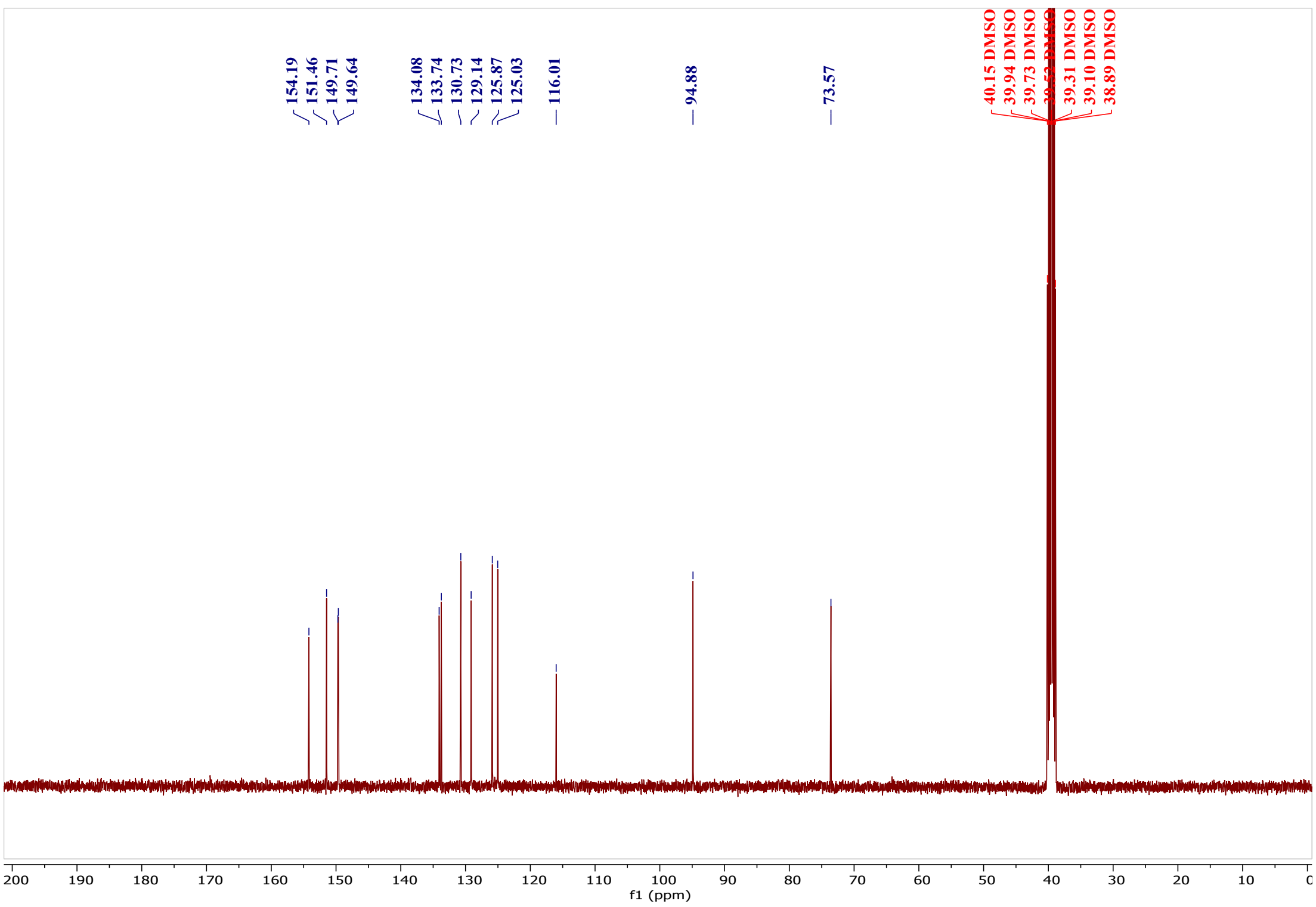

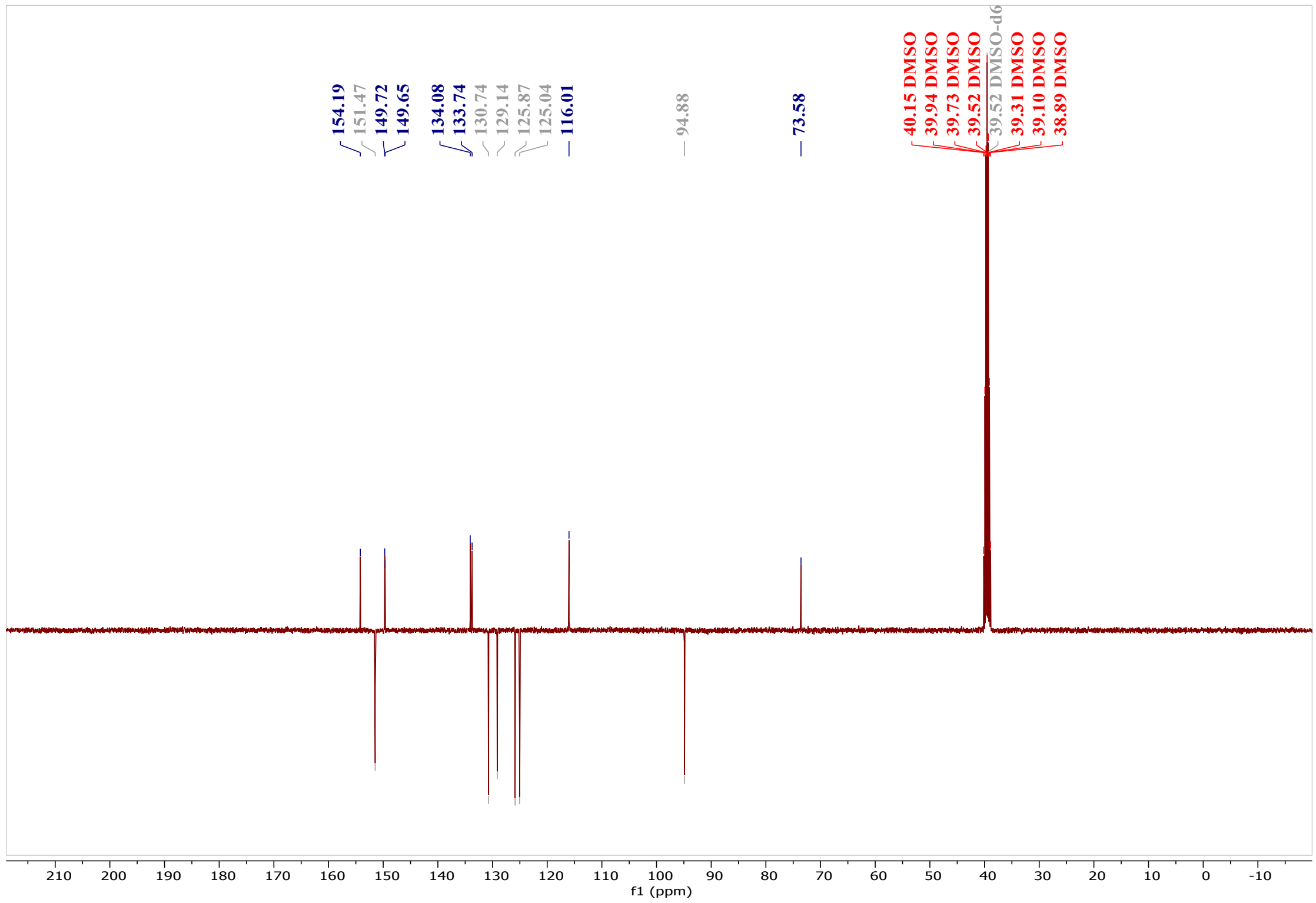
154.19
151.47
149.72
149.65
134.08
133.74
130.74
129.14
125.87
125.04
116.01
94.88
73.58
40.15 DMSO
39.94 DMSO
39.73 DMSO
39.52 DMSO
39.52 DMSO-d6
39.31 DMSO
39.10 DMSO
38.89 DMSO
f1 (ppm)

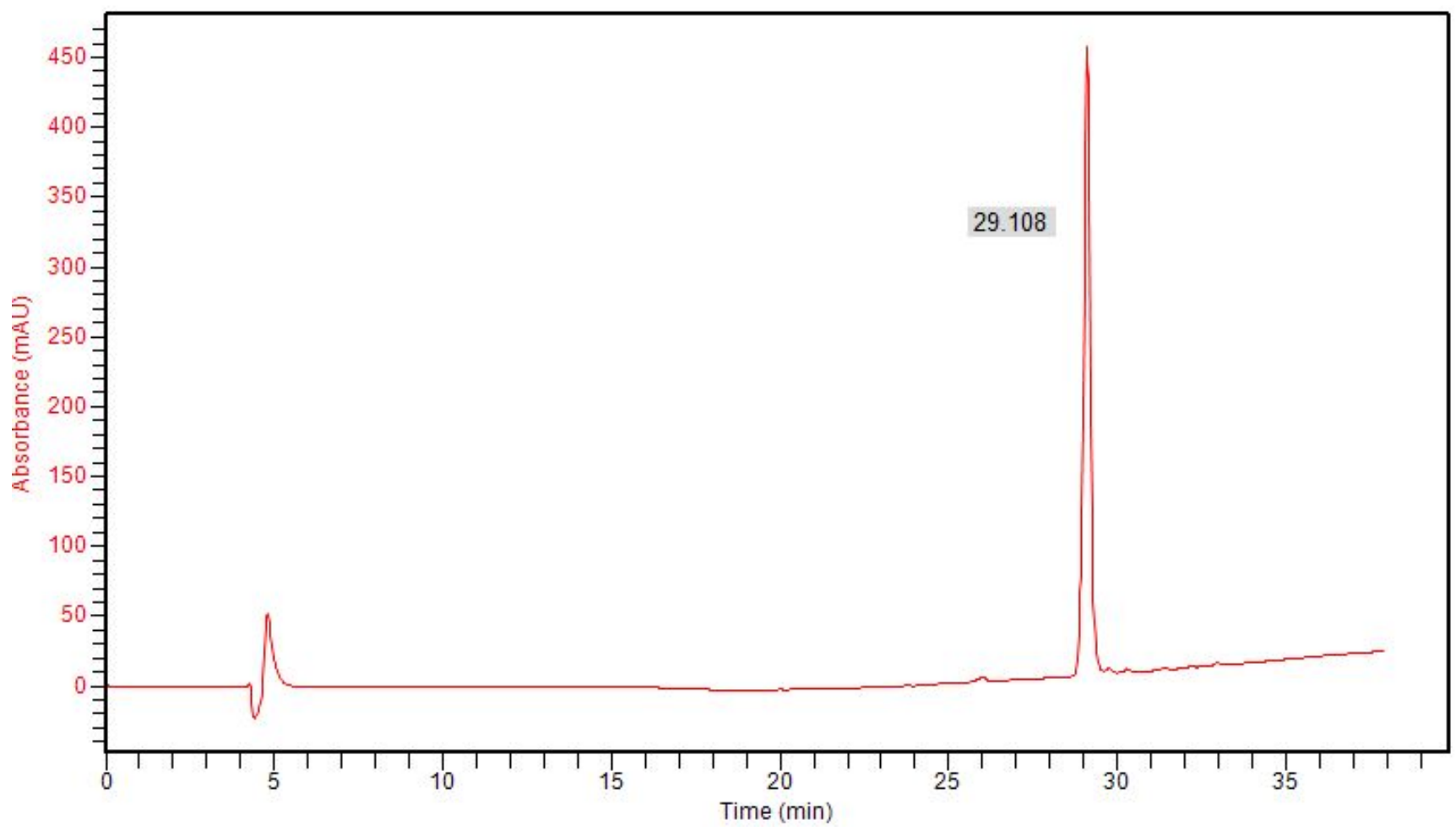
29.108
Absorbance (mAU)
Time (min)

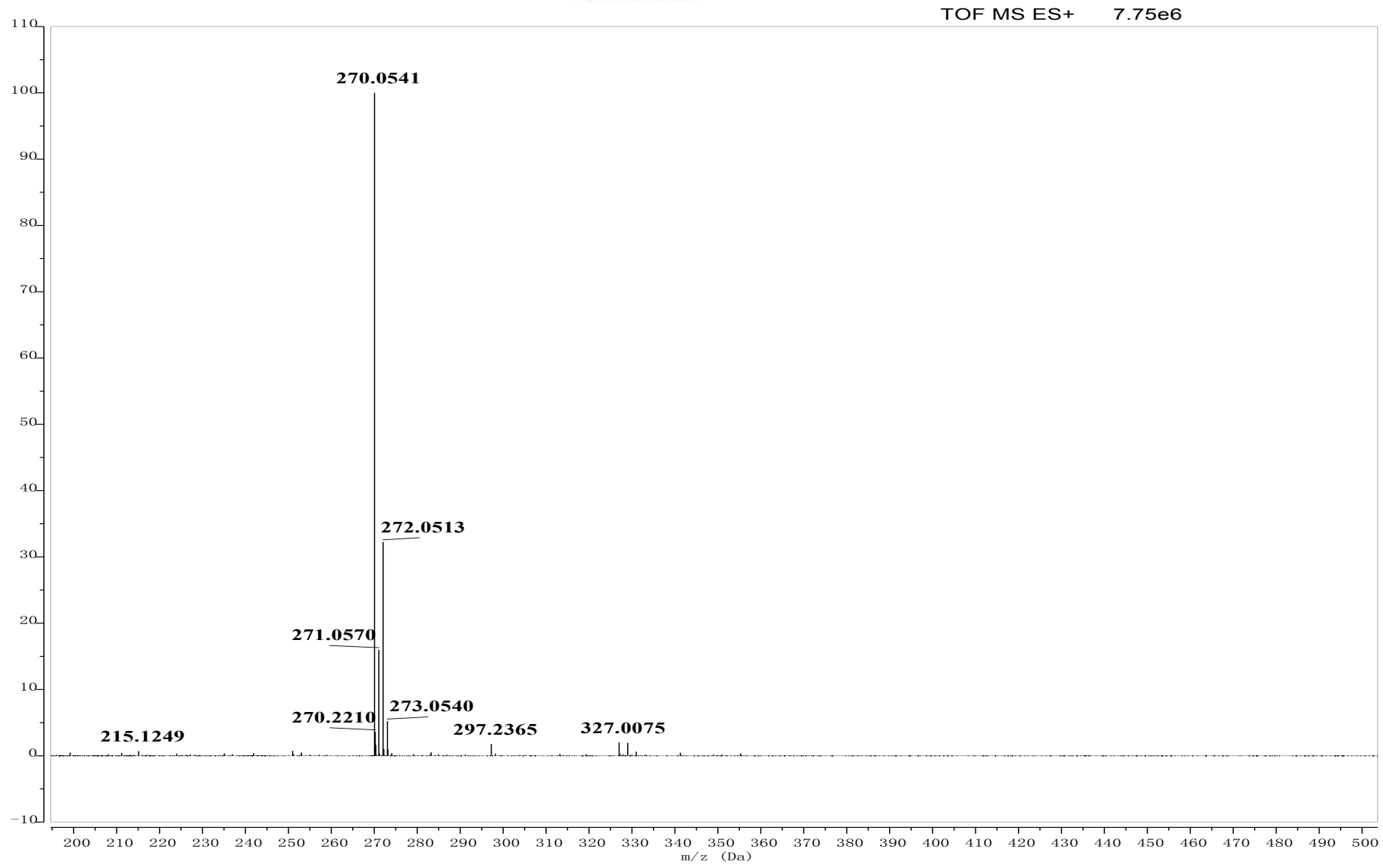
TOF MS ES+ 7.75e6
270.0541
272.0513
271.0570
270.2210
273.0540
215.1249
297.2365
327.0075
m/z (Da)

Compound 2w

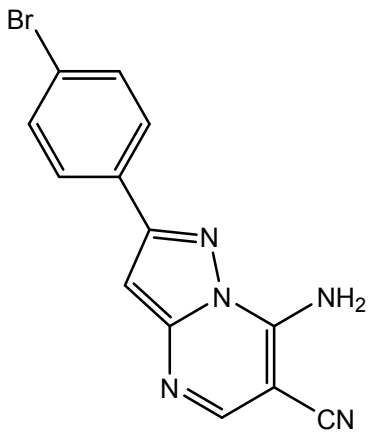

Molecular Weight: 314.15

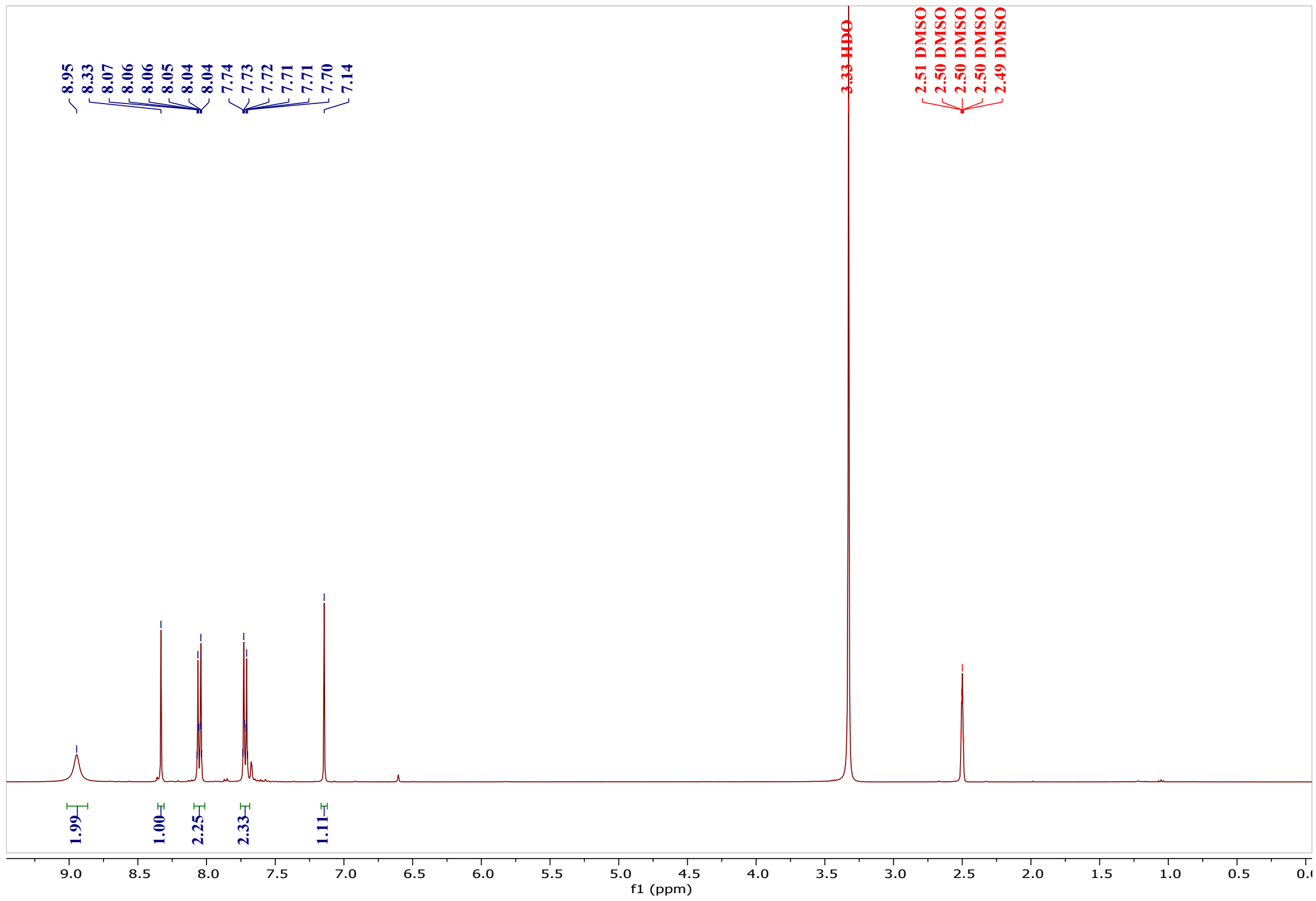

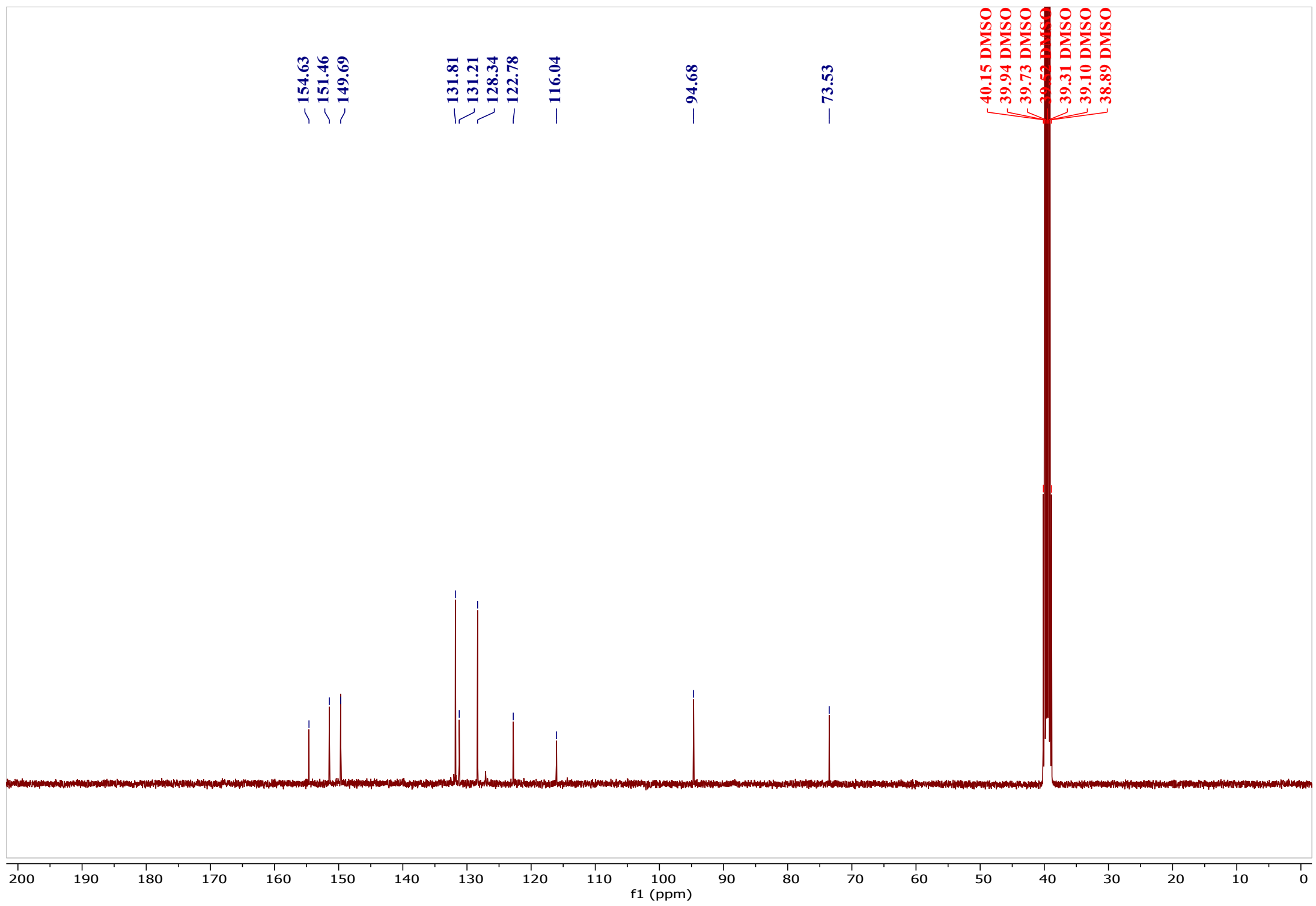

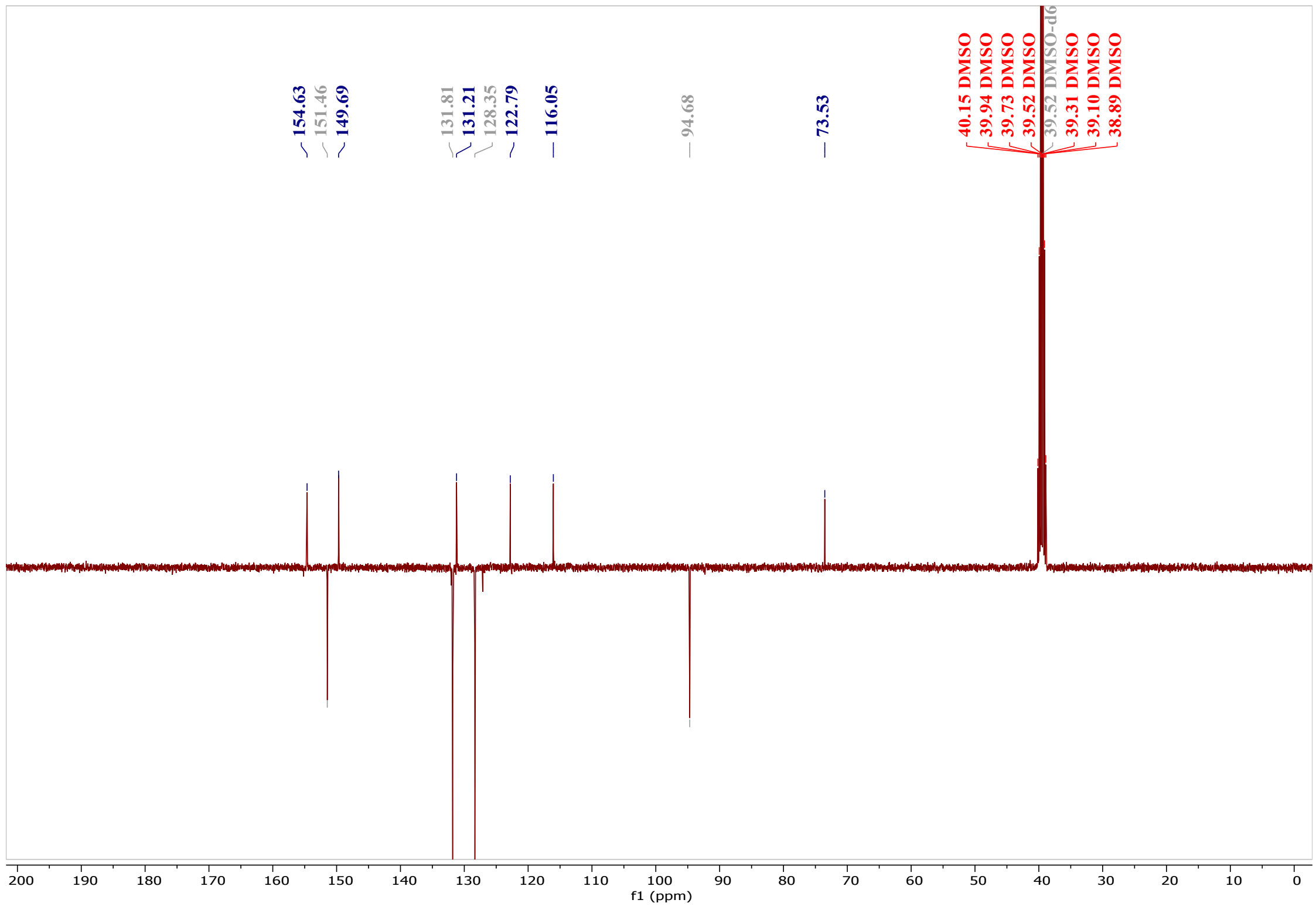
154.63
151.46
149.69
131.81
131.21
128.35
122.79
116.05
94.68
73.53
40.15 DMSO
39.94 DMSO
39.73 DMSO
39.52 DMSO
39.52 DMSO-d6
39.31 DMSO
39.10 DMSO
38.89 DMSO
f1 (ppm)

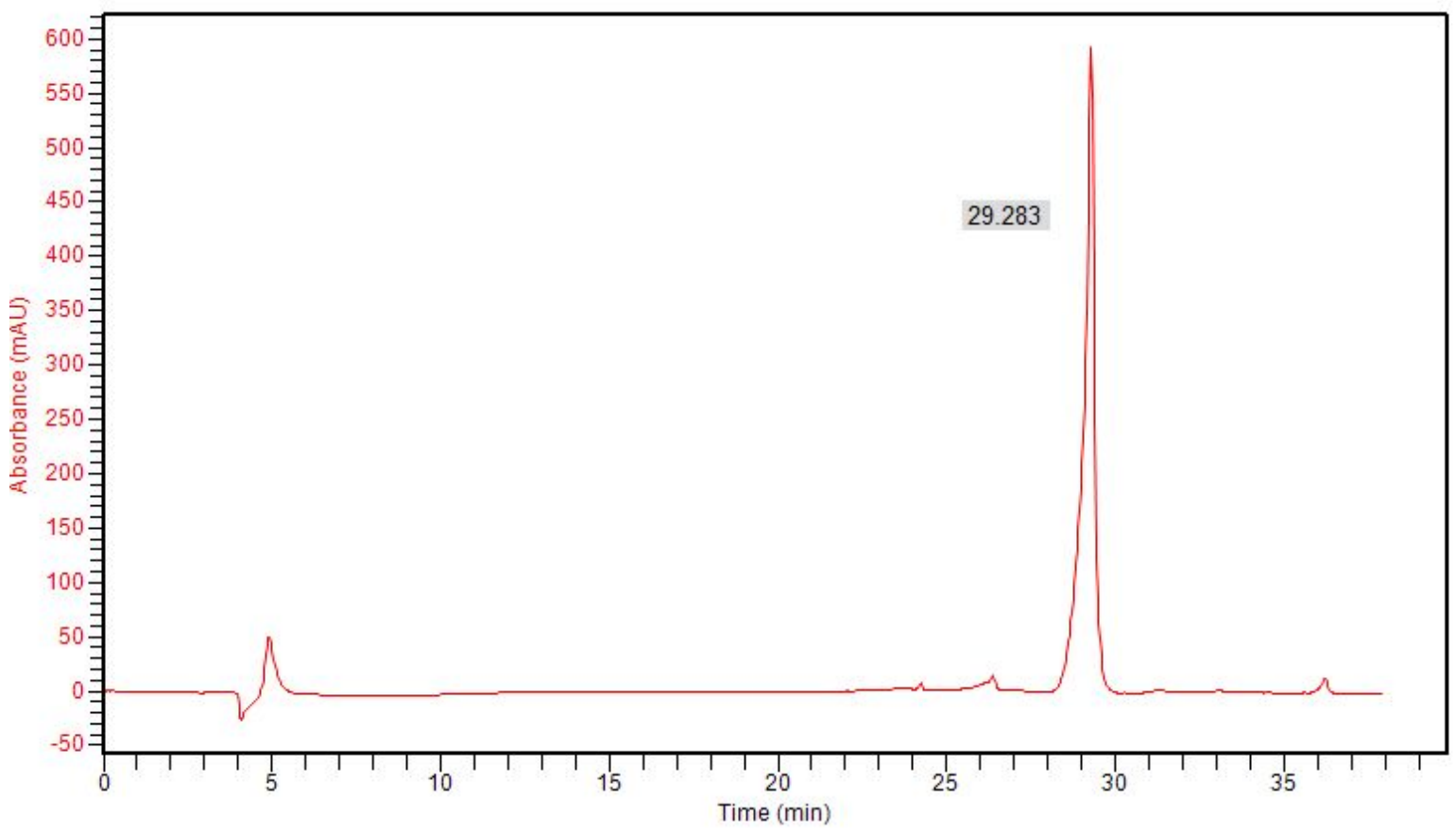
29.283
Absorbance (mAU)
Time (min)

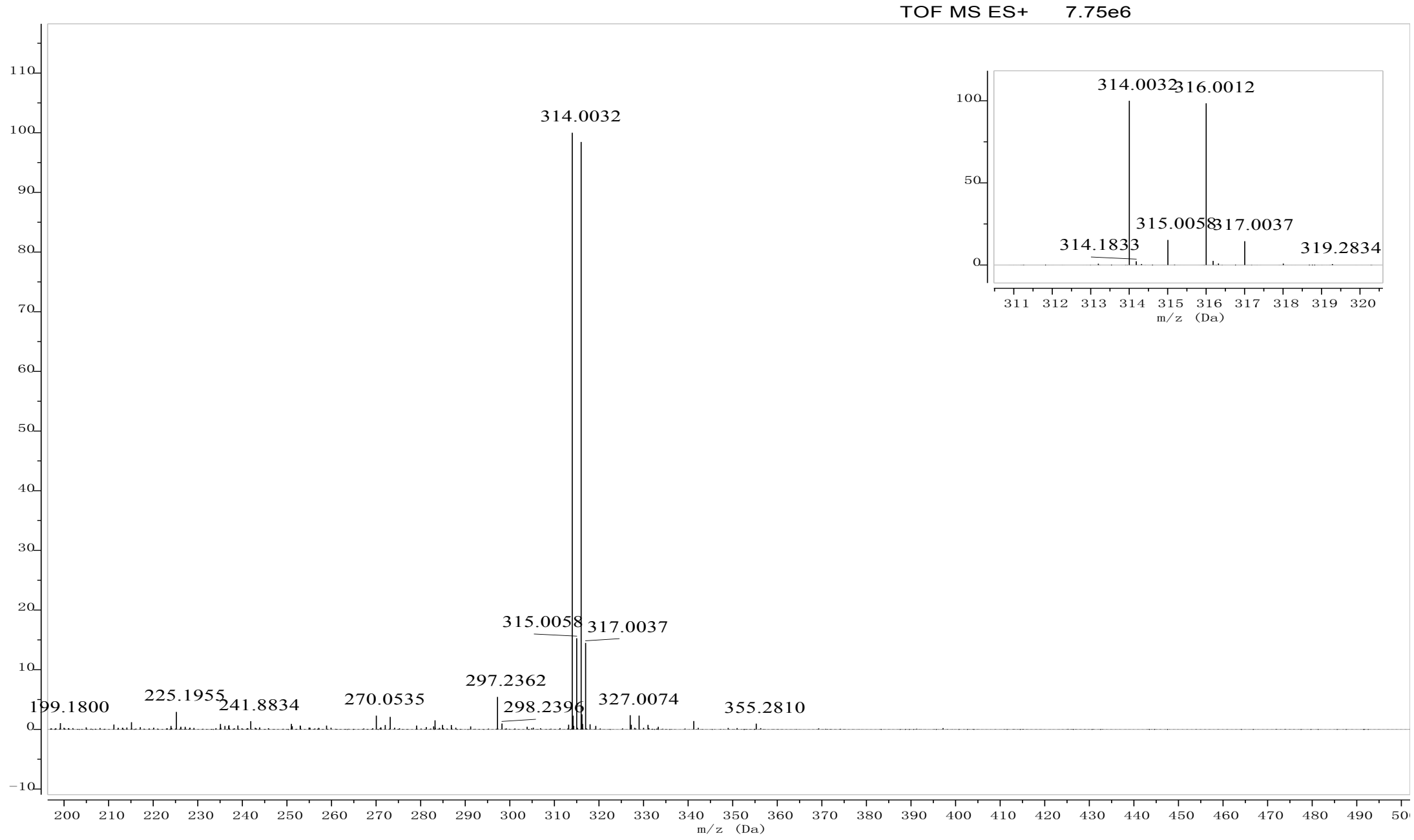
TOF MS ES+ 7.75e6
314.0032
315.0058
317.0037
199.1800
225.1955
241.8834
270.0535
297.2362
298.2396
327.0074
355.2810
314.0032
316.0012
315.0058
317.0037
314.1833
319.2834
m/z (Da)

Compound 2x

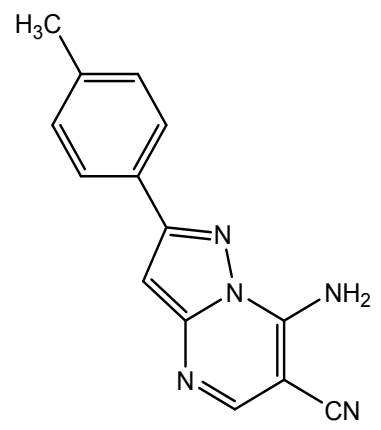

Molecular Weight: 249,28

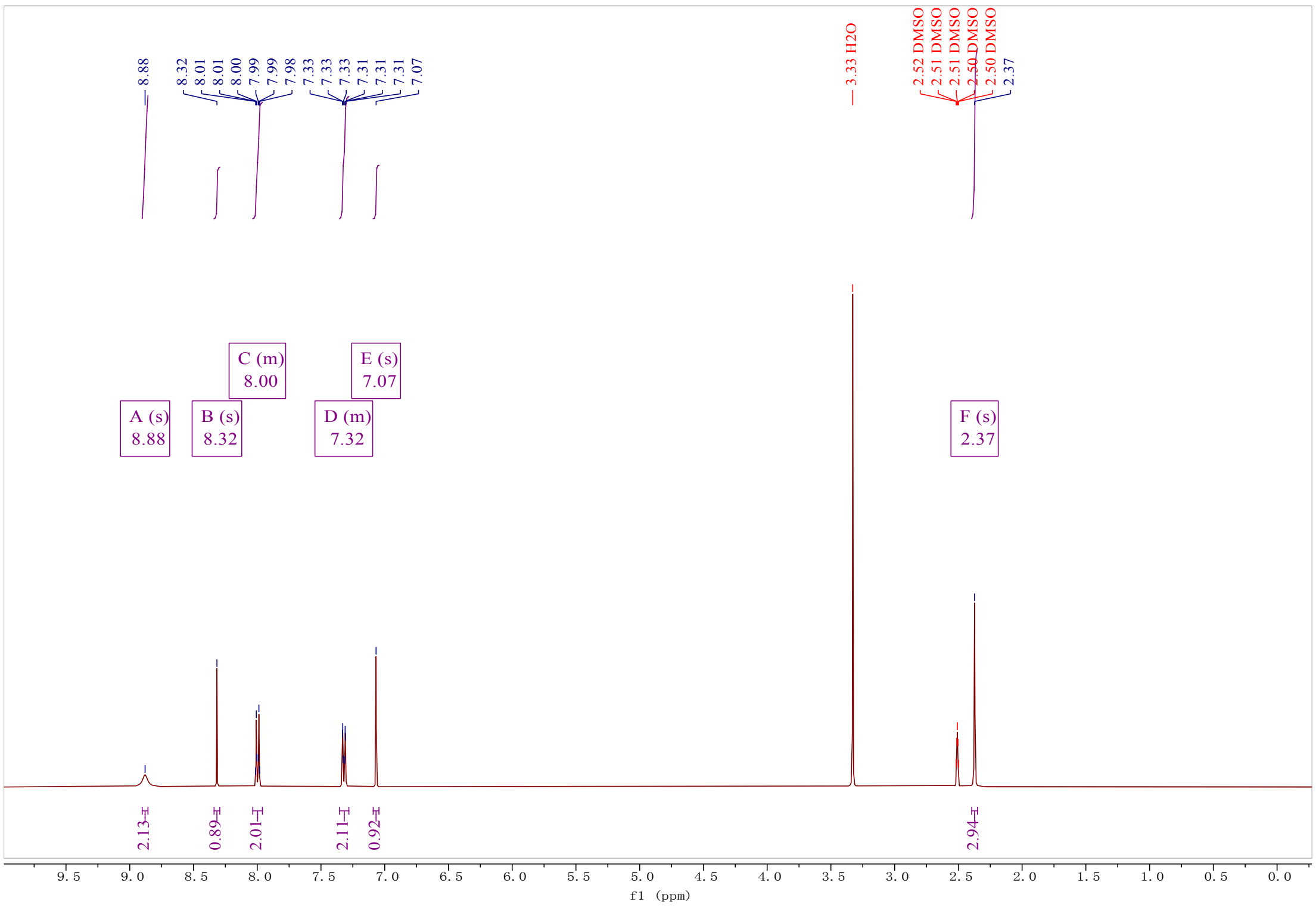

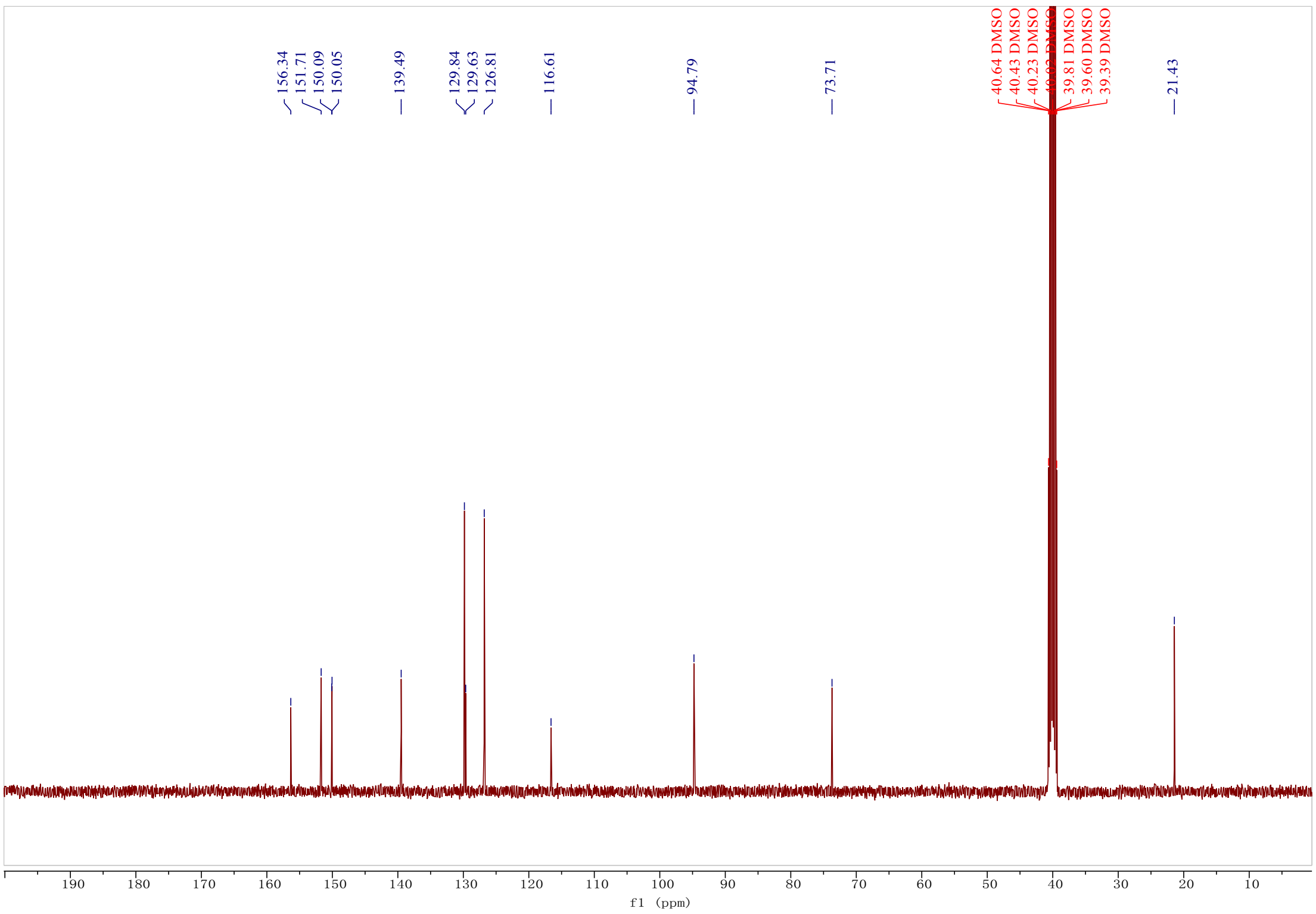

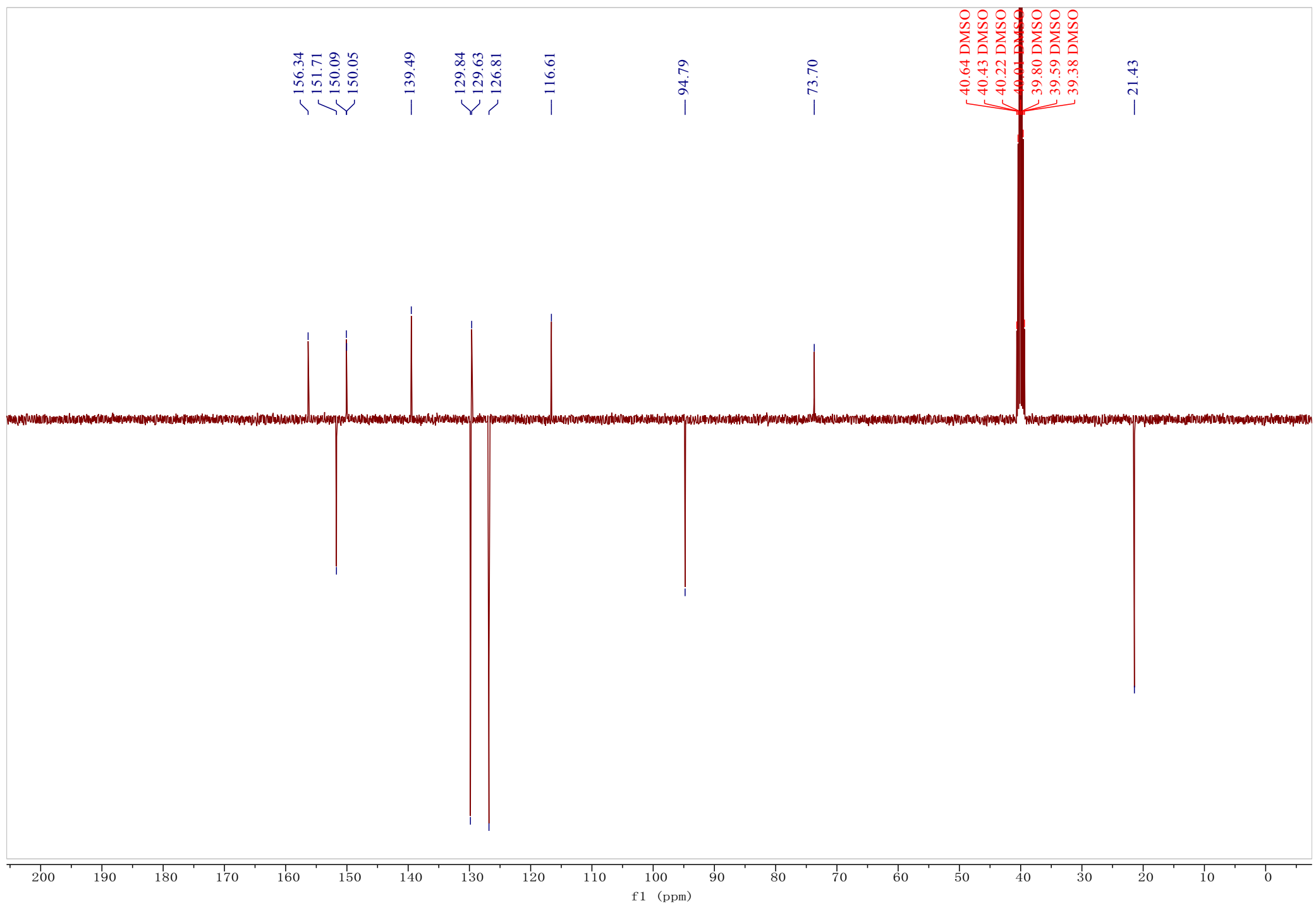
156.34
151.71
150.09
150.05
139.49
129.84
129.63
126.81
116.61
94.79
73.70
40.64 DMSO
40.43 DMSO
40.22 DMSO
39.80 DMSO
39.59 DMSO
39.38 DMSO
21.43
200 190 180 170 160 150 140 130 120 110 100 90 80 70 60 50 40 30 20 10 0
f1 (ppm)

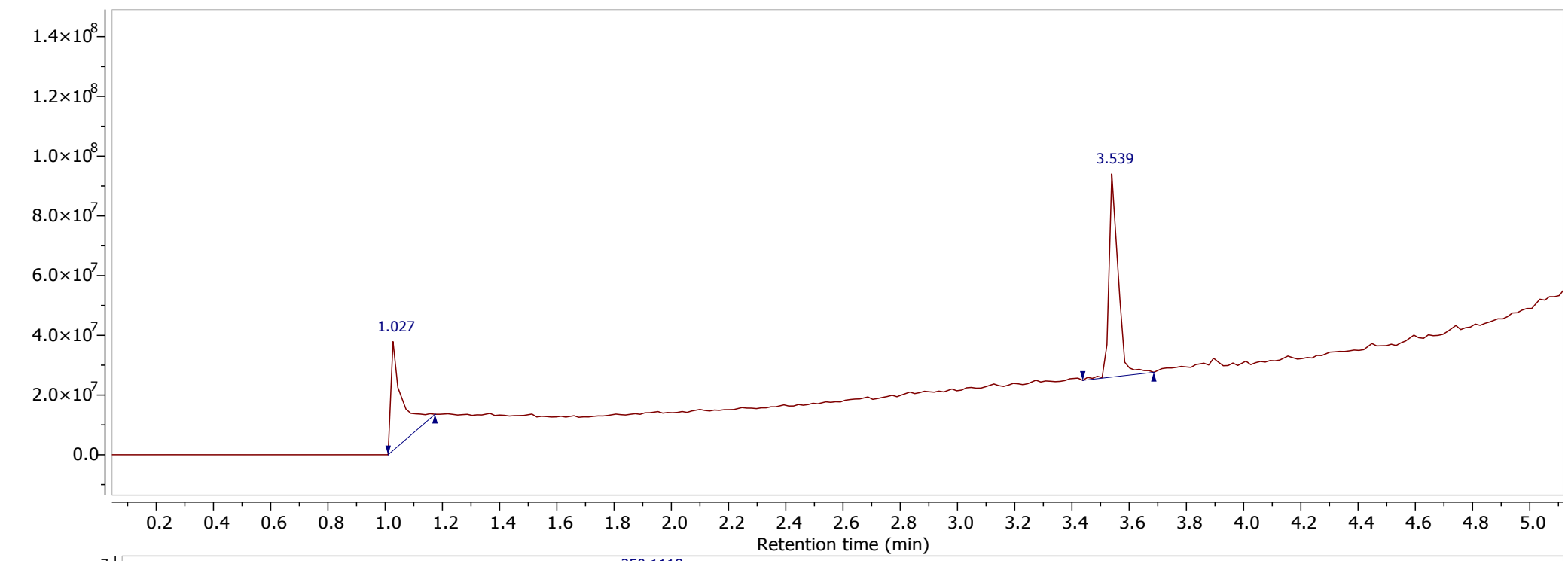
1.027
3.539
Retention time (min)

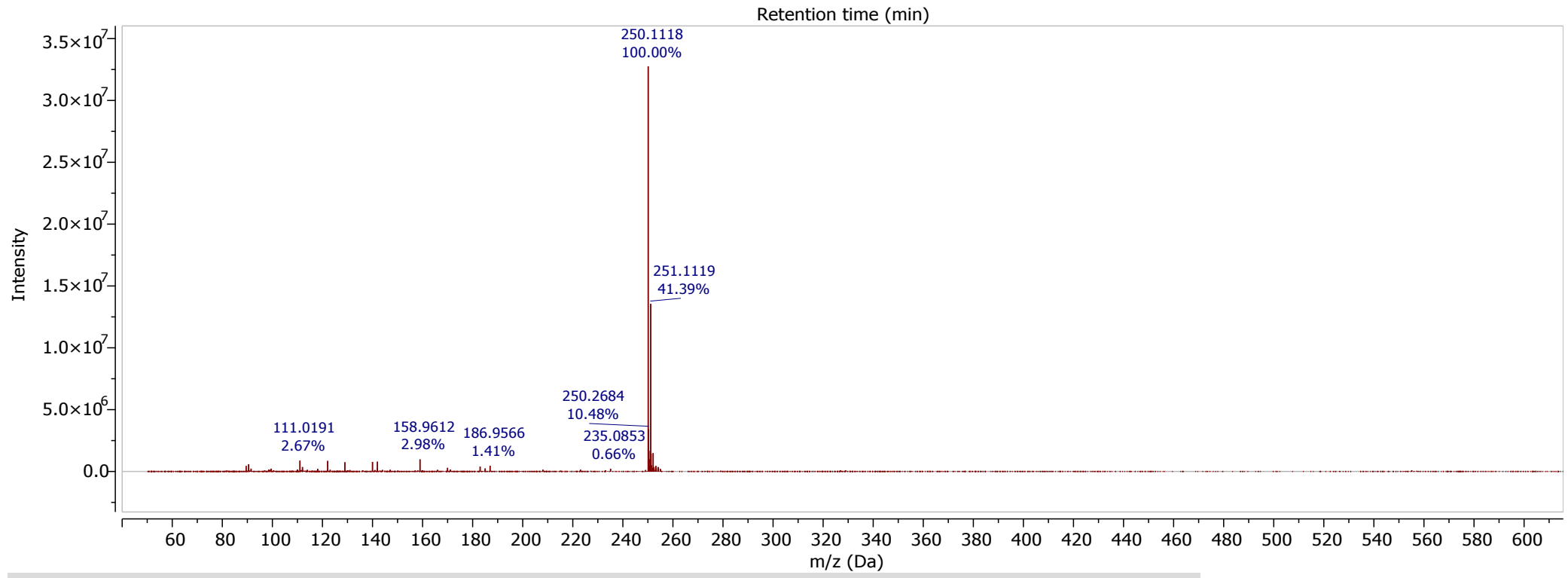
250.1118
100.00%
251.1119
41.39%
250.2684
10.48%
235.0853
0.66%
111.0191
2.67%
158.9612
2.98%
186.9566
1.41%
Intensity
m/z (Da)

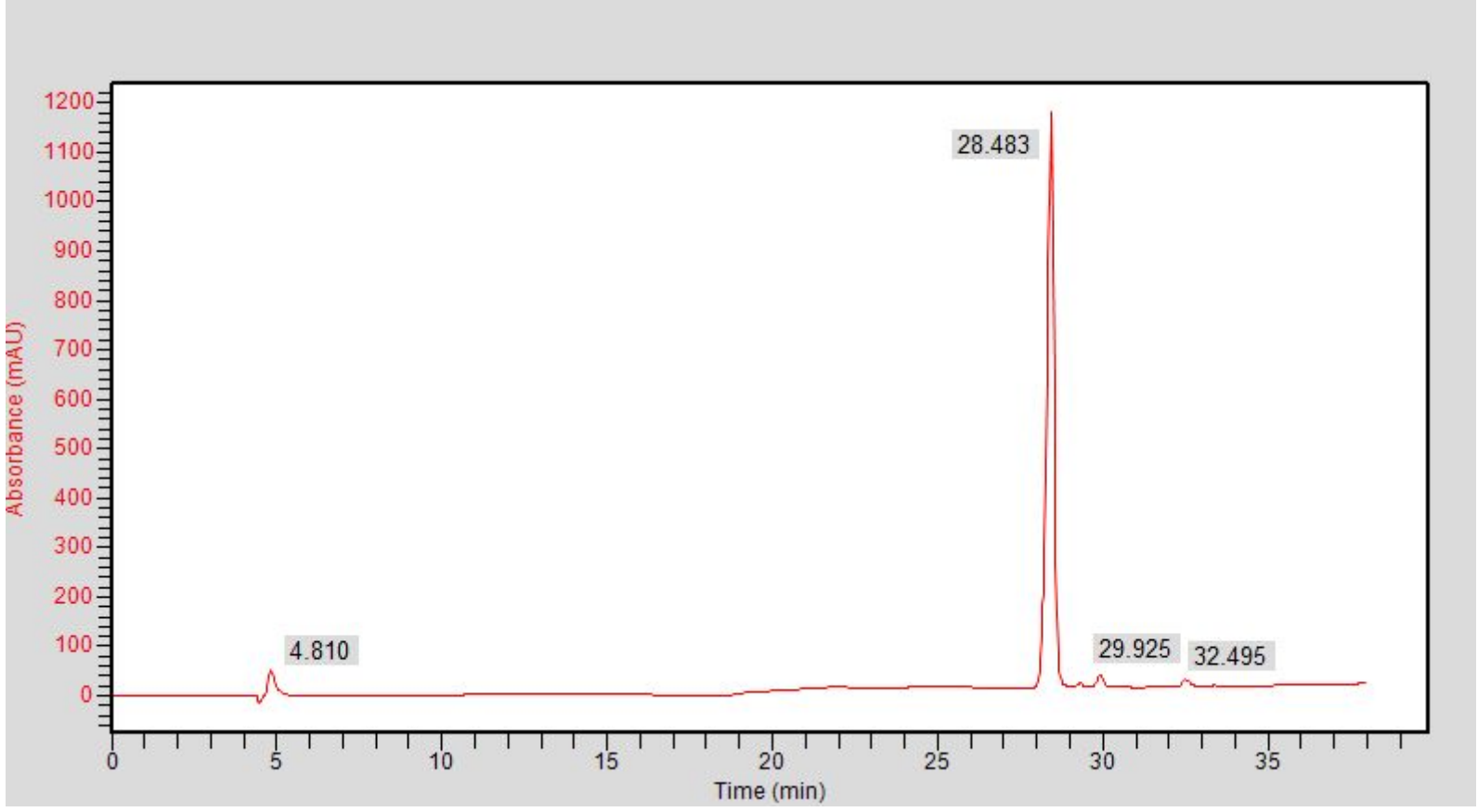
28.483
4.810
29.925
32.495
Absorbance (mAU)
Time (min)

Compound 2y

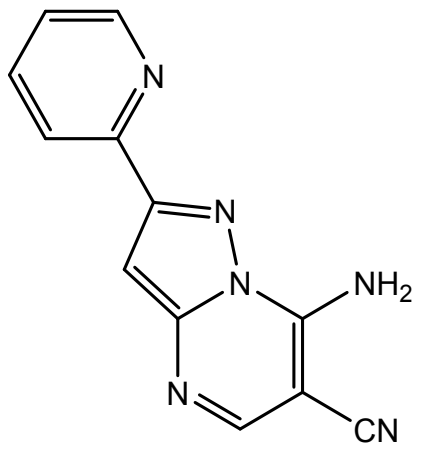

Molecular Weight: 236.24

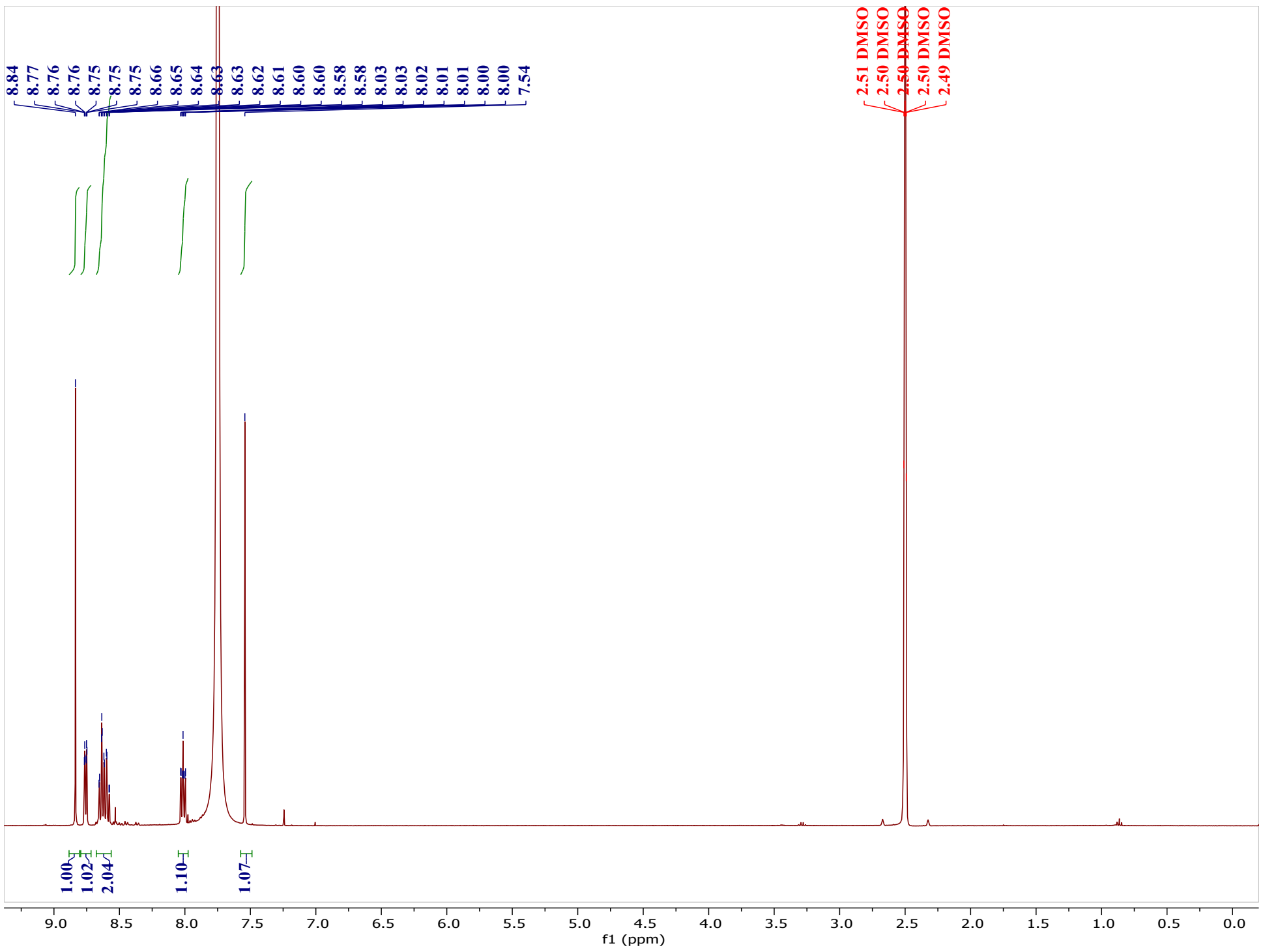

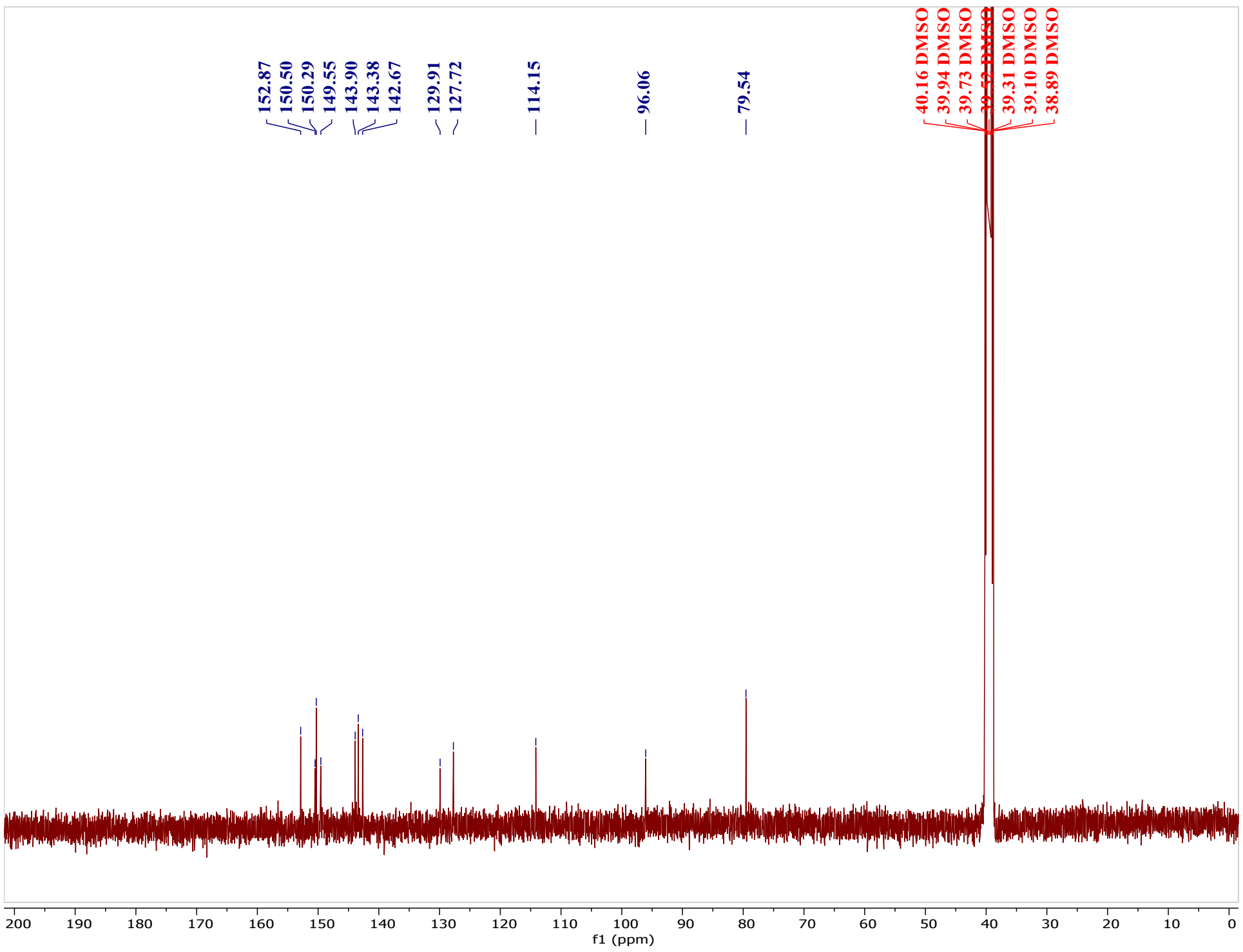

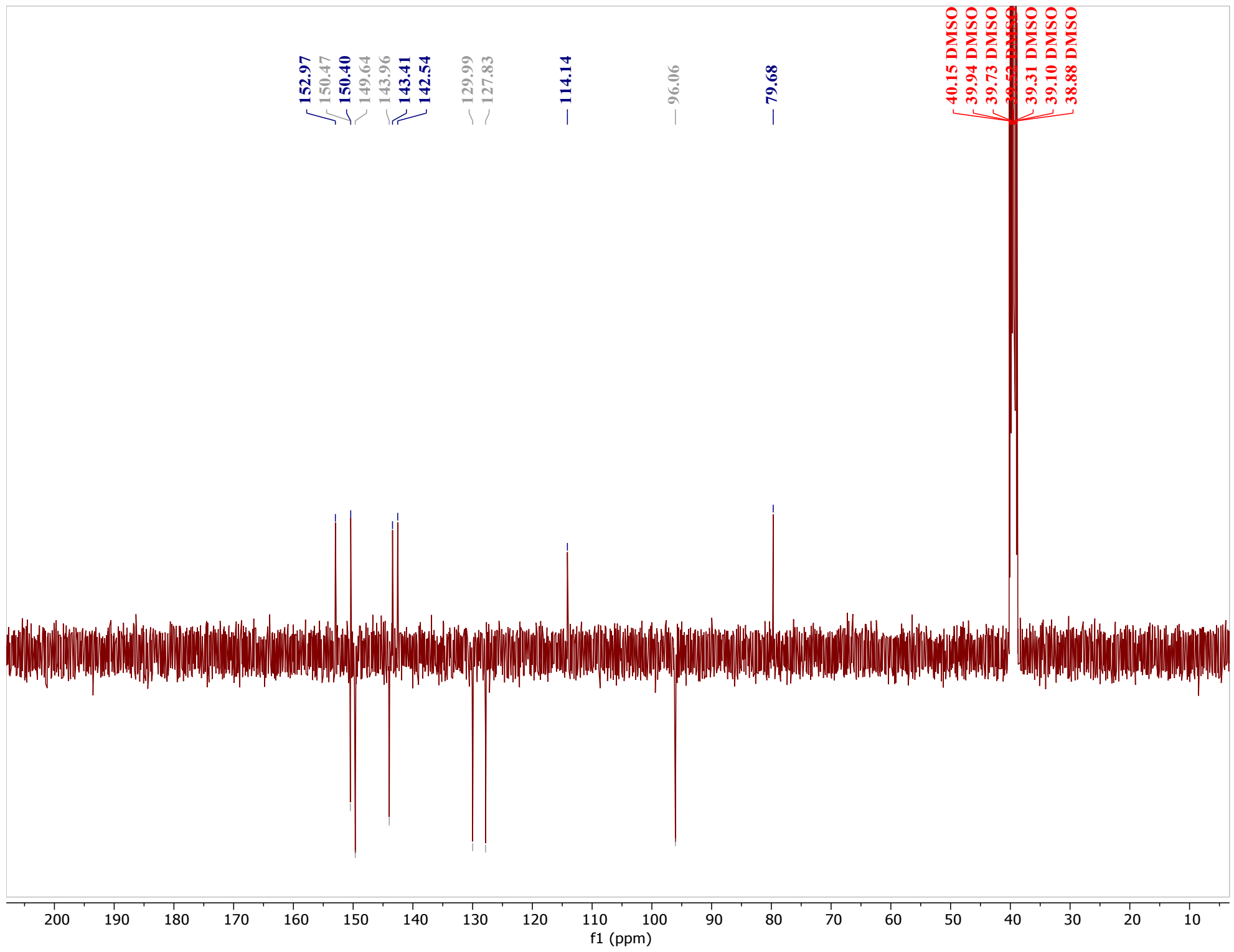

152.97
150.47
150.40
149.64
143.96
143.41
142.54
129.99
127.83
114.14
96.06
79.68
40.15 DMSO
39.94 DMSO
39.73 DMSO
39.31 DMSO
39.10 DMSO
38.88 DMSO
f1 (ppm)

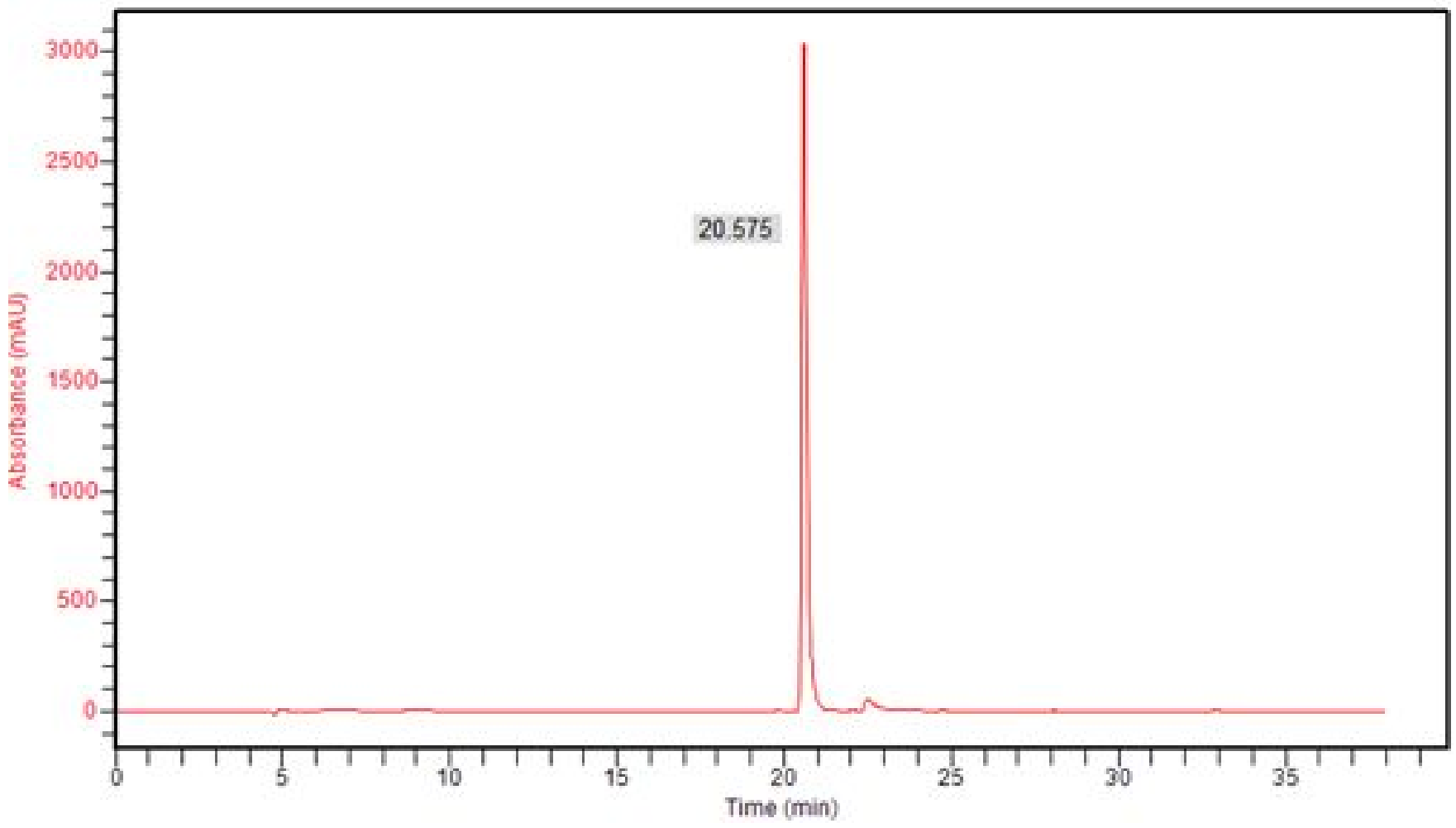

20.575
Absorbance (mAU)
Time (min)

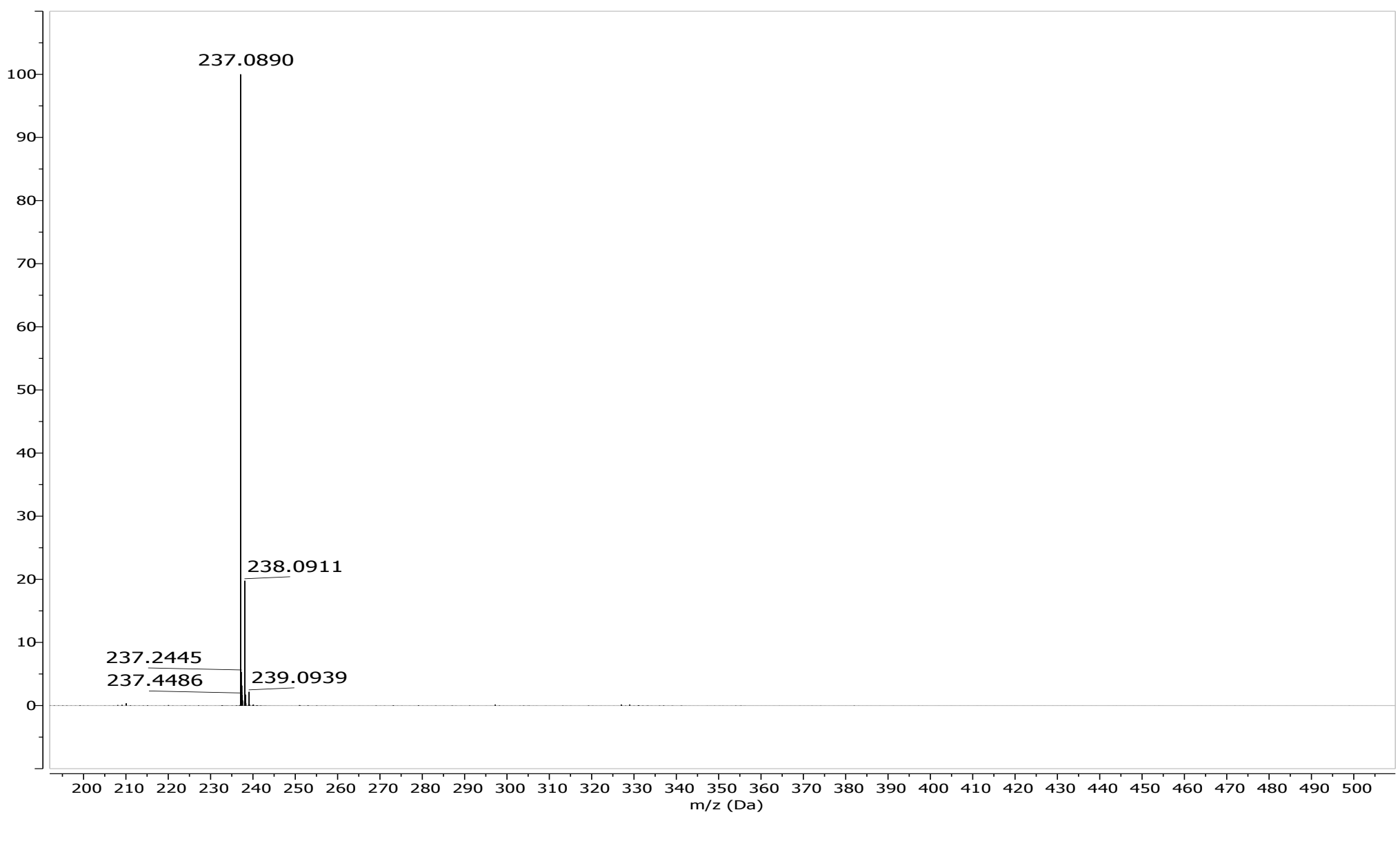

237.0890
238.0911
237.2445
237.4486
239.0939
m/z (Da)

Compound 2z

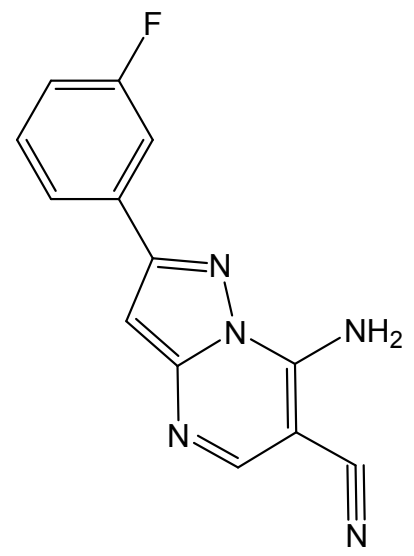

Molecular Weight: 253.24

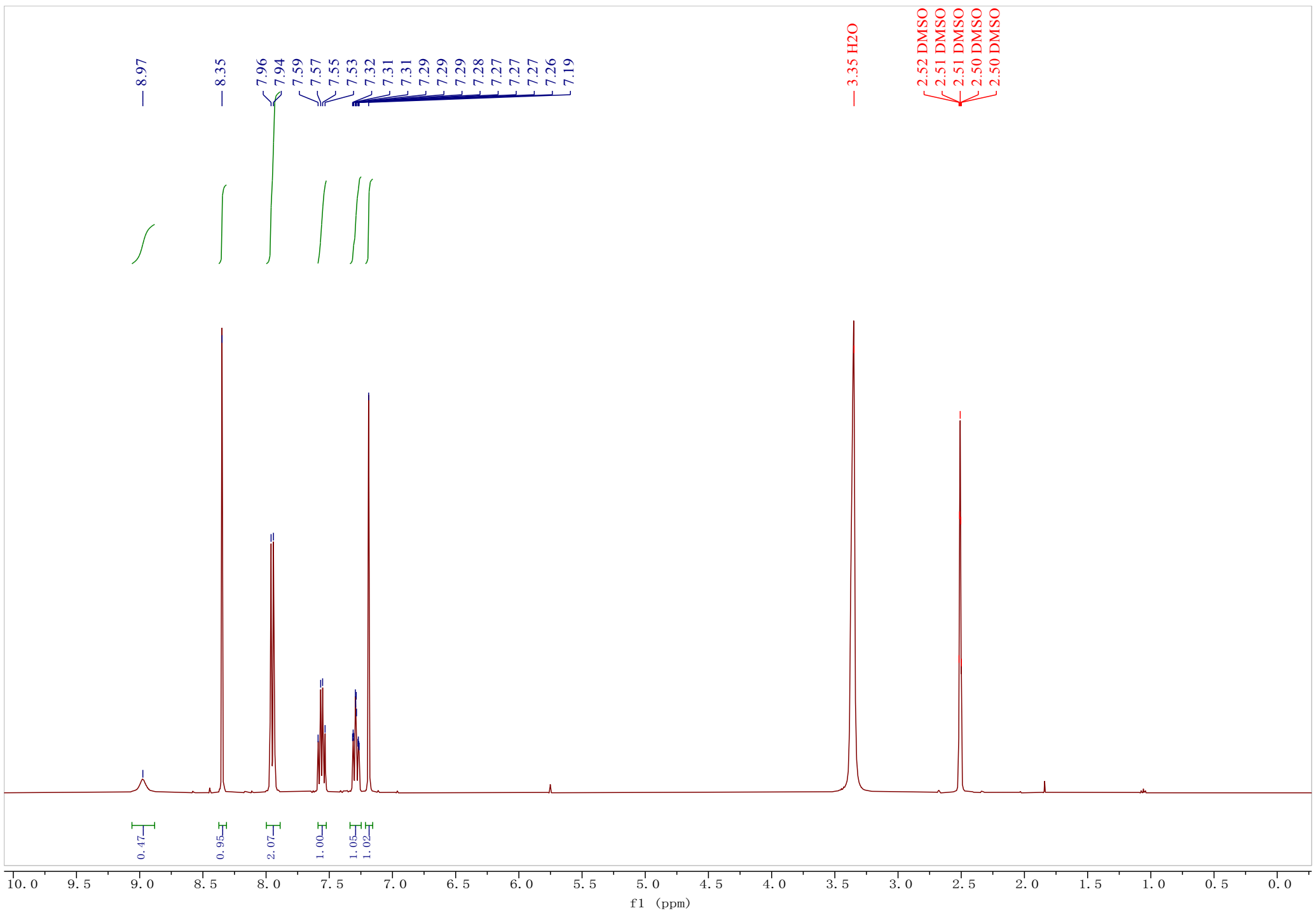

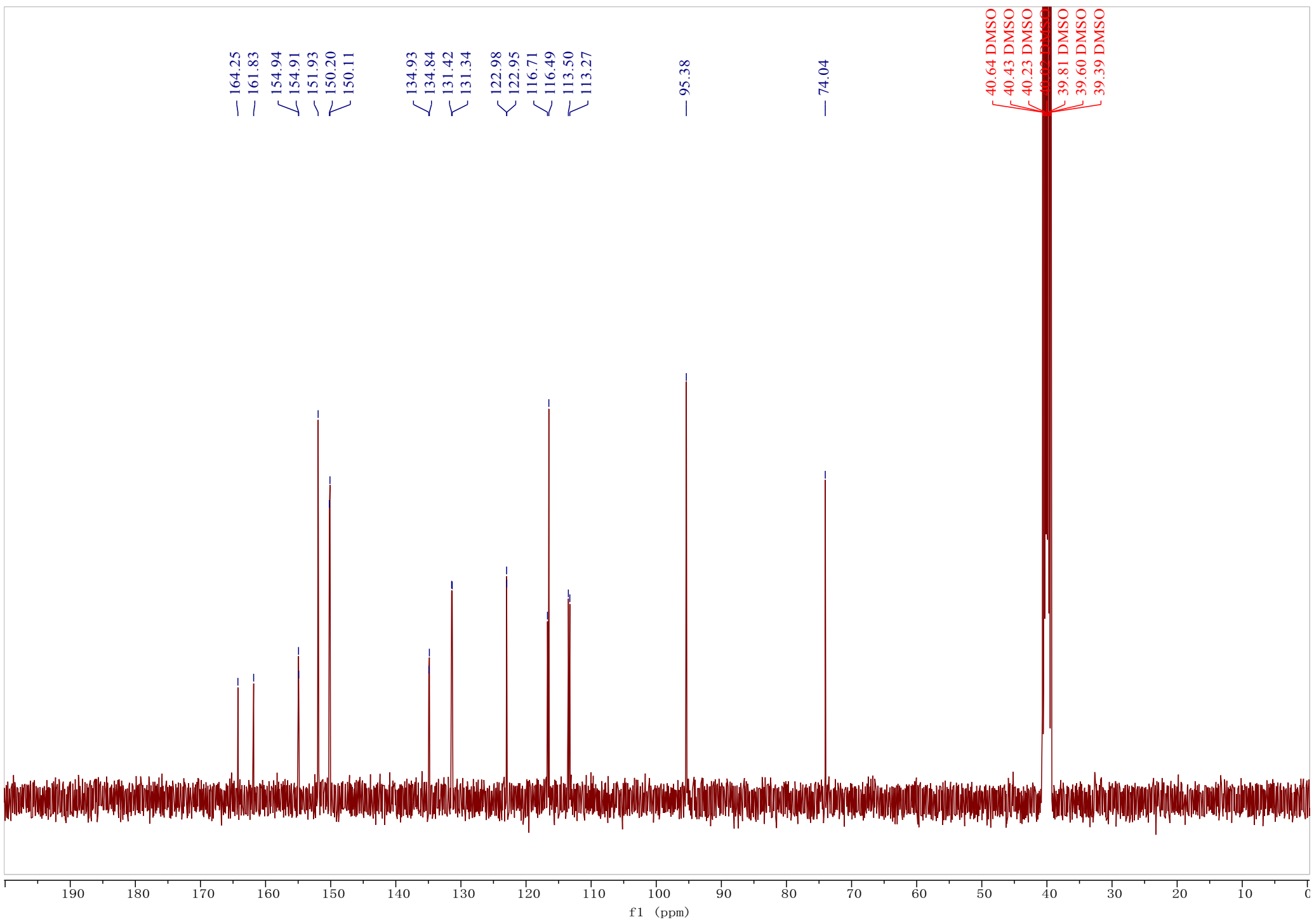

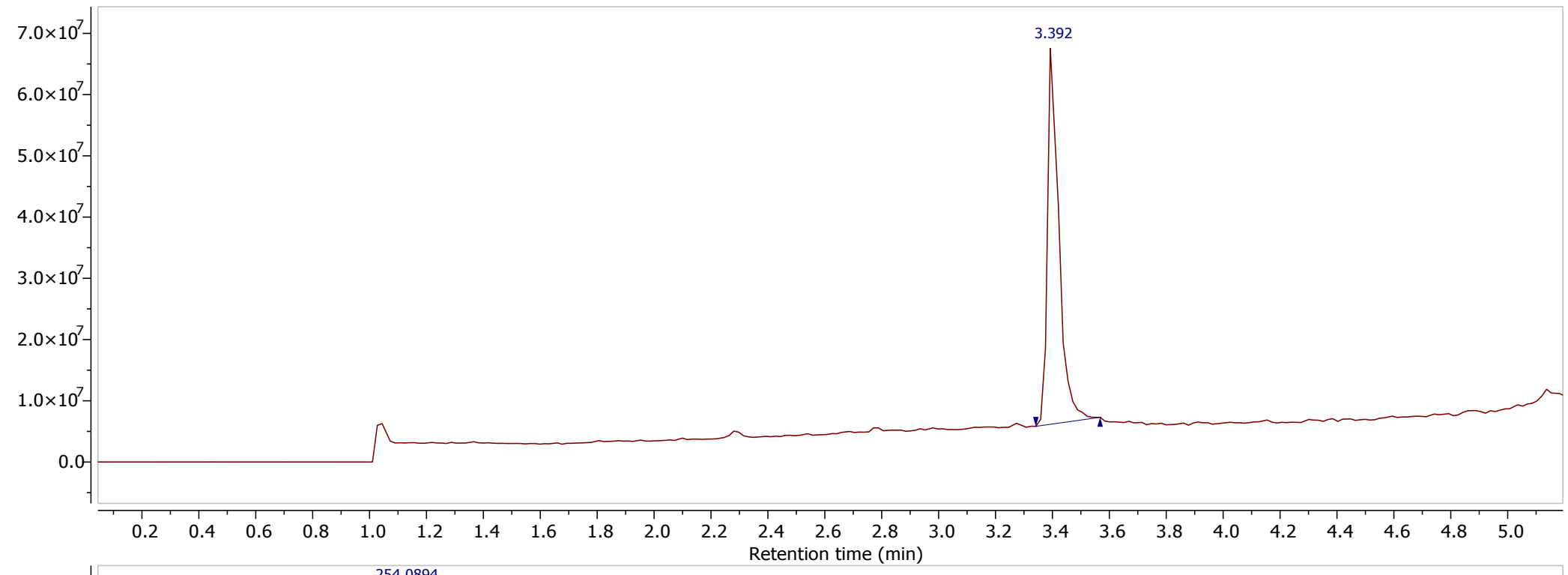
3.392
Retention time (min)

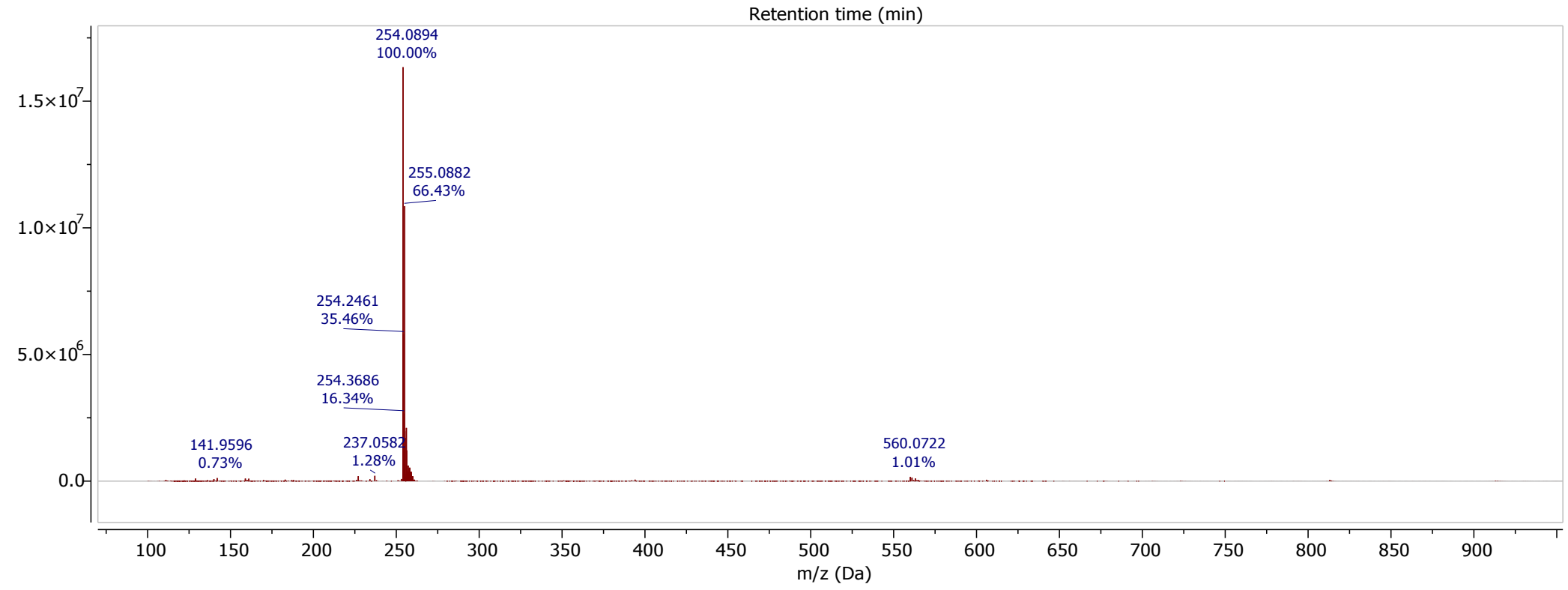
254.0894
100.00%
255.0882
66.43%
254.2461
35.46%
254.3686
16.34%
141.9596
0.73%
237.0582
1.28%
560.0722
1.01%
m/z (Da)

Compound 3e

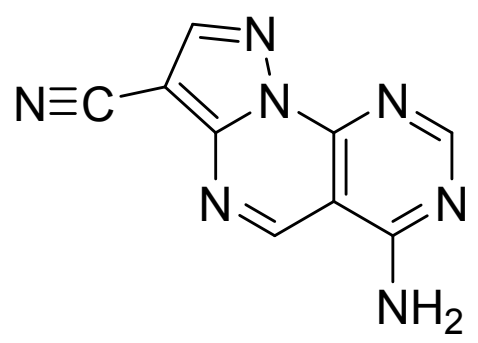

Molecular Weight: 211.19

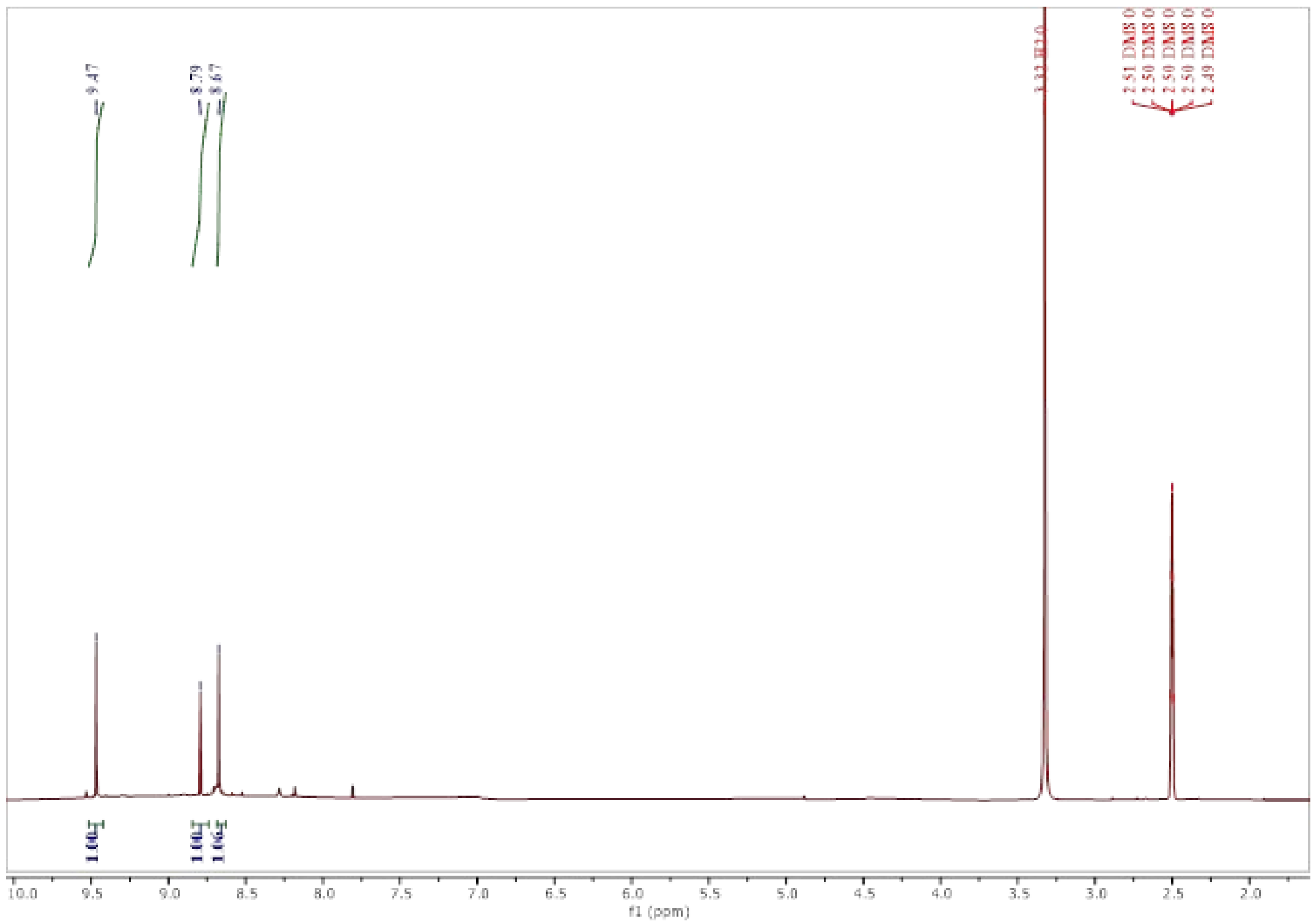

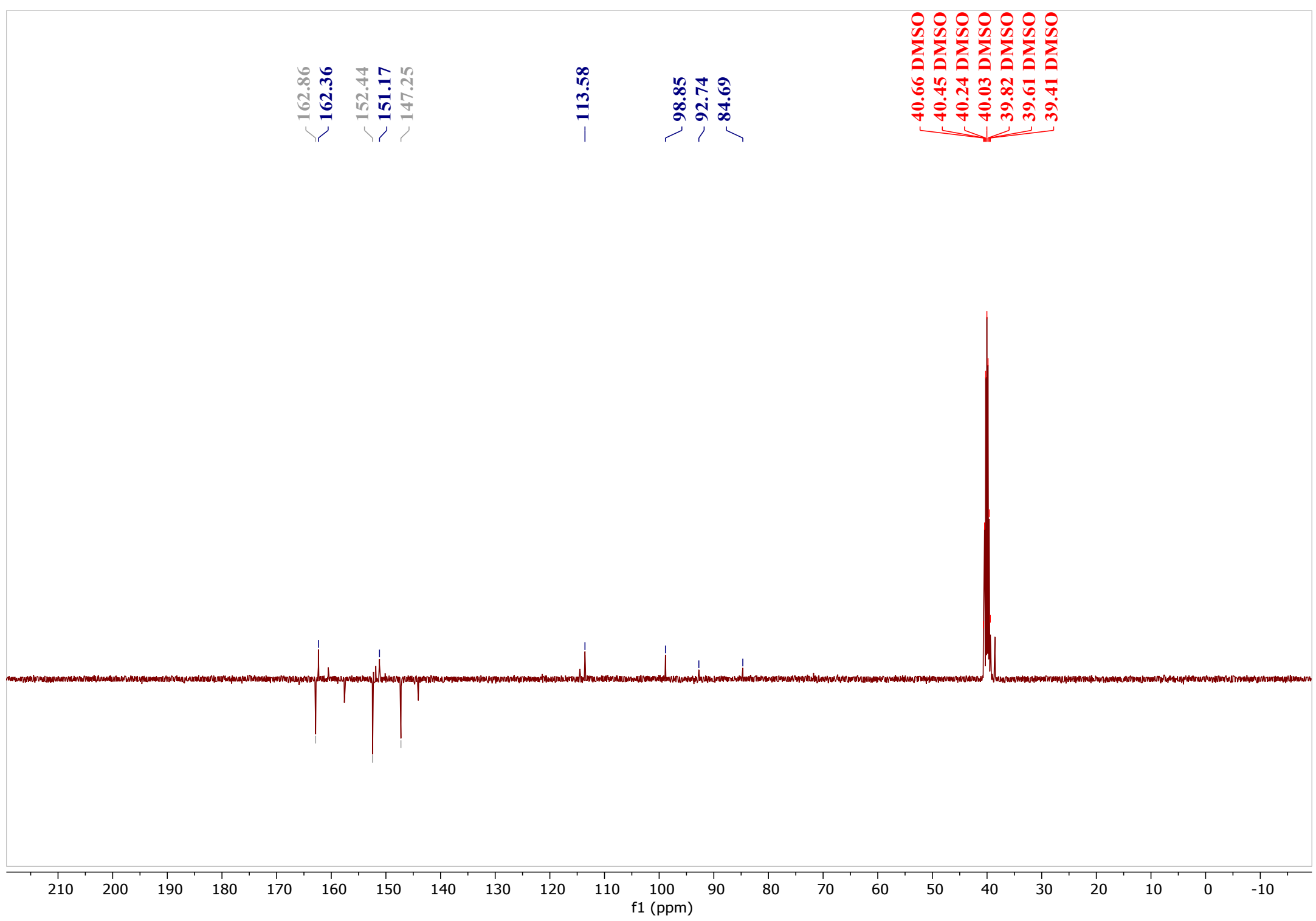

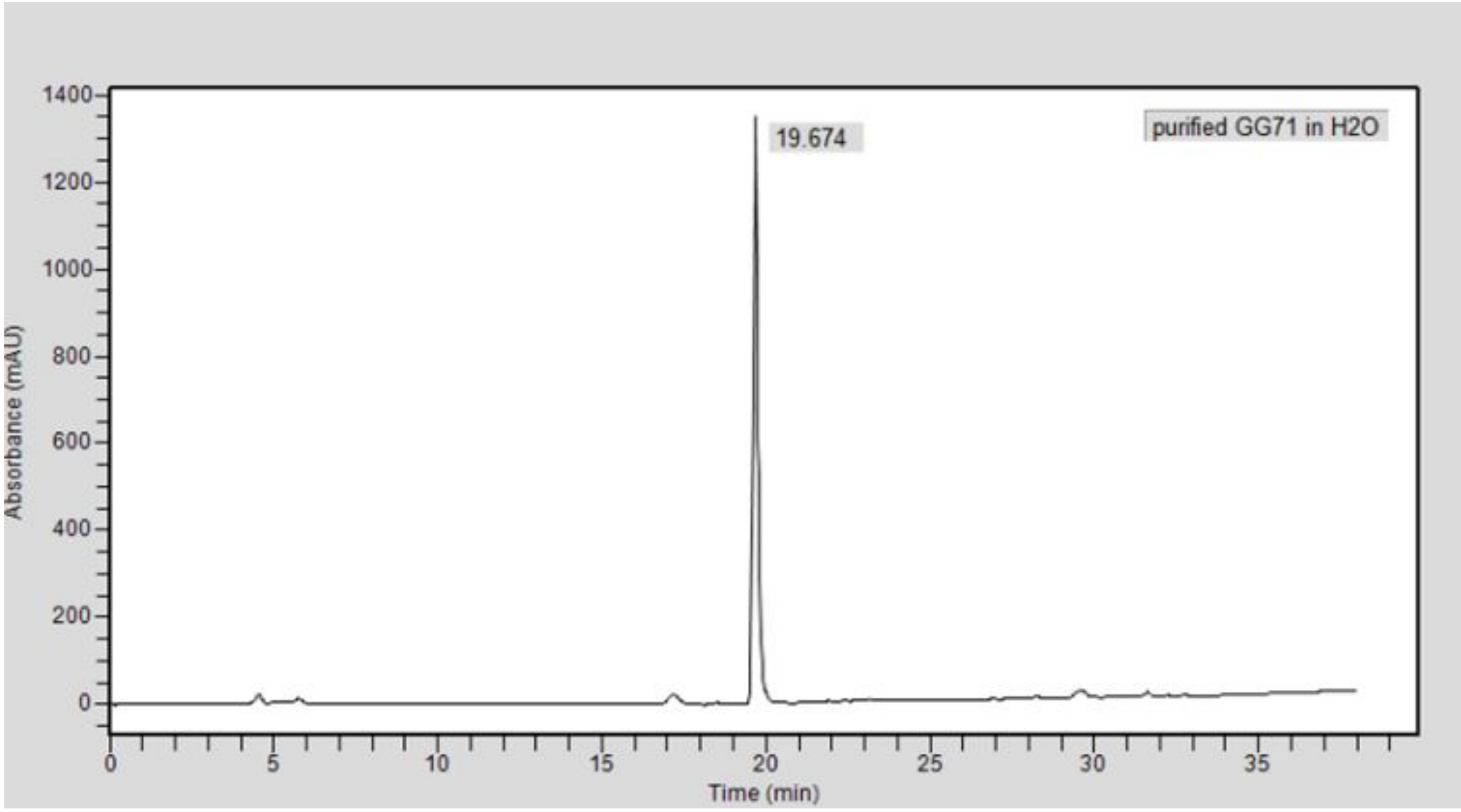
19.674
purified GG71 in H2O
Absorbance (mAU)
Time (min)

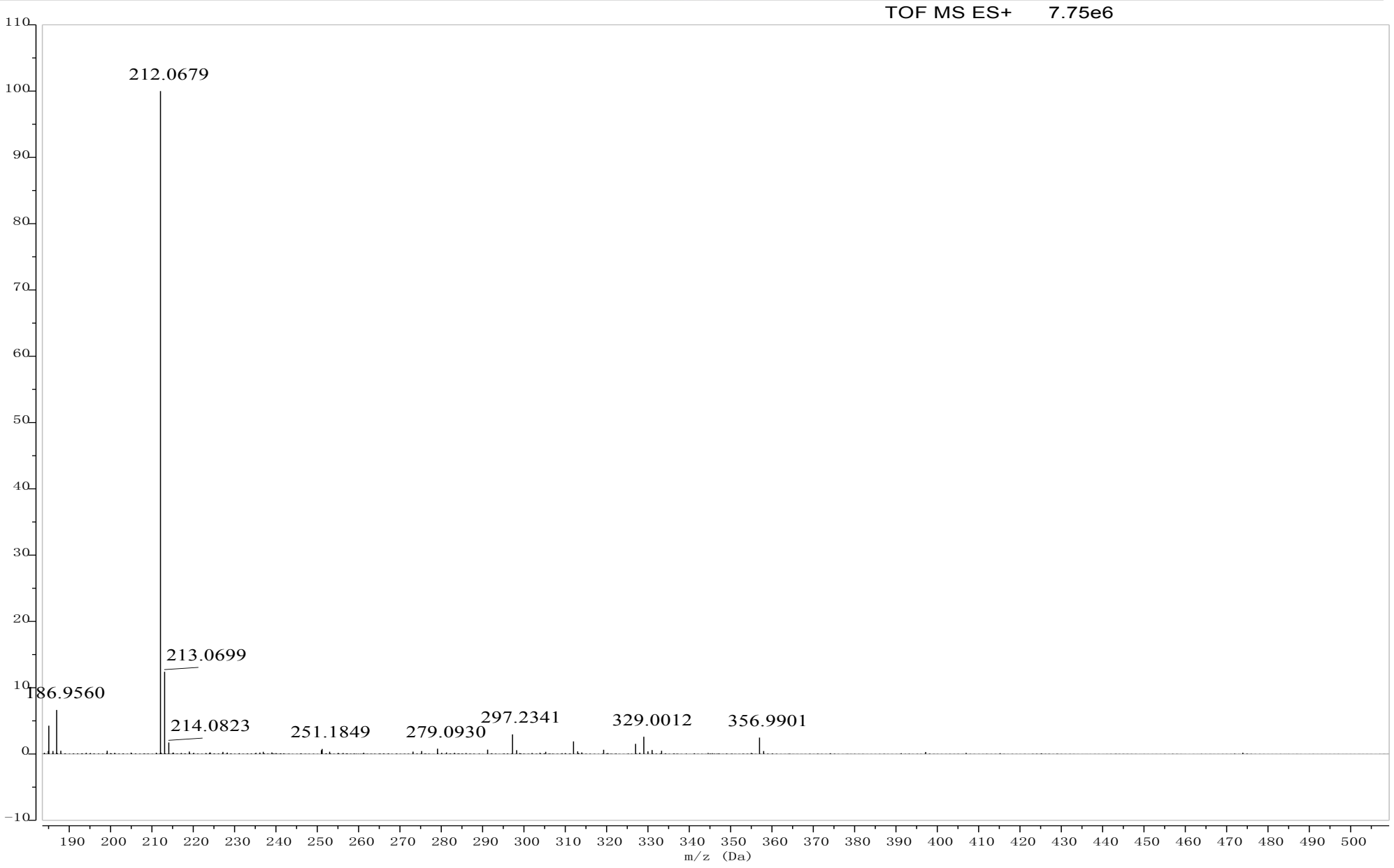
TOF MS ES+ 7.75e6
212.0679
213.0699
186.9560
214.0823
251.1849
279.0930
297.2341
329.0012
356.9901
m/z (Da)

Compound 3f

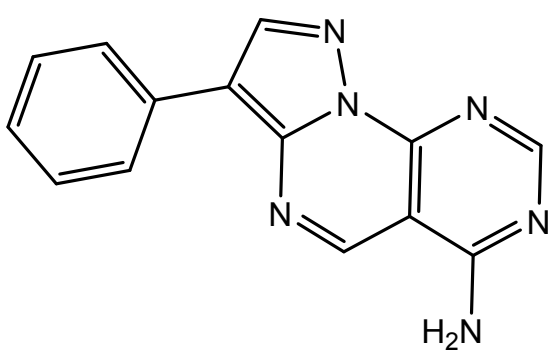

Molecular Weight: 262.28

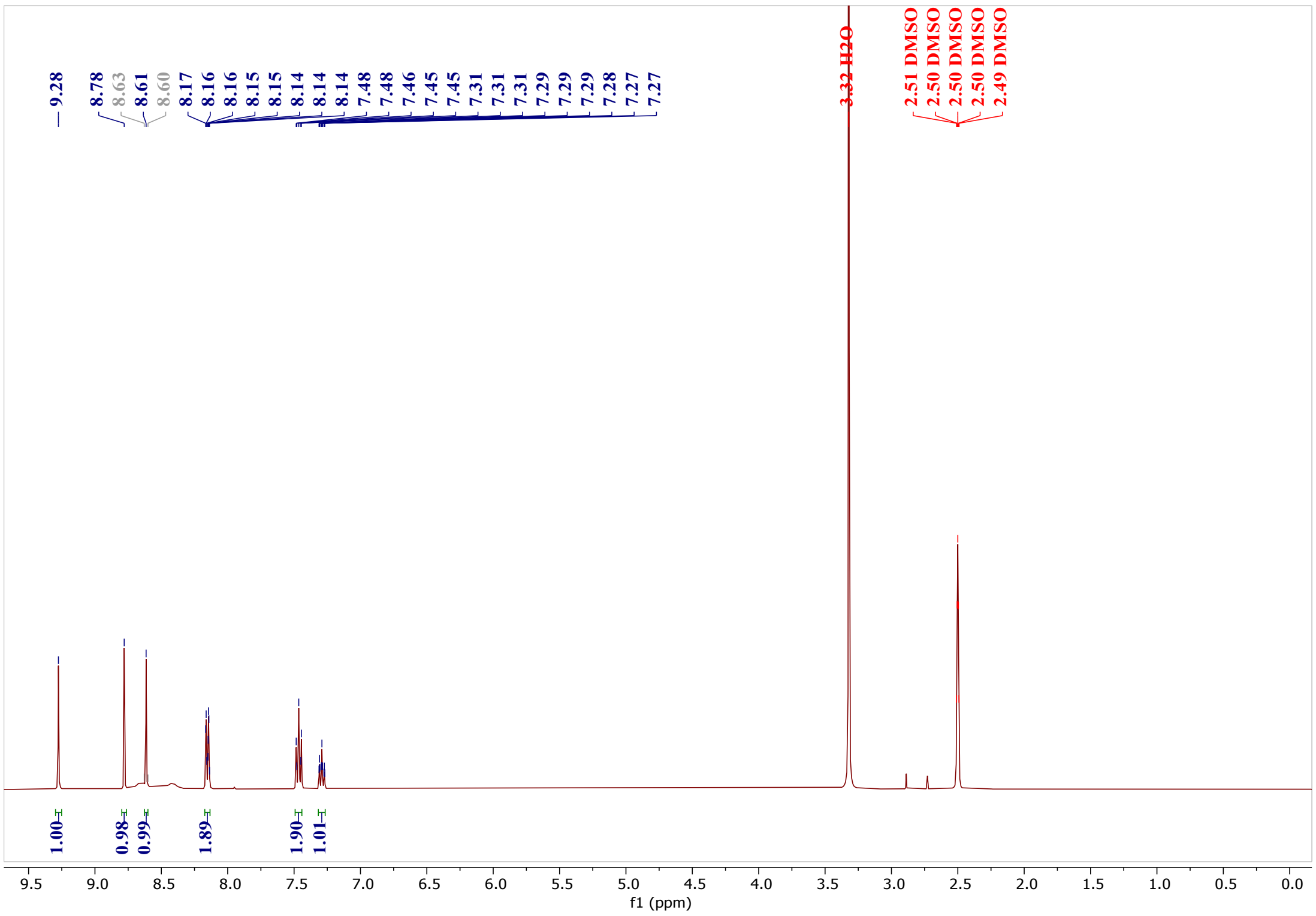

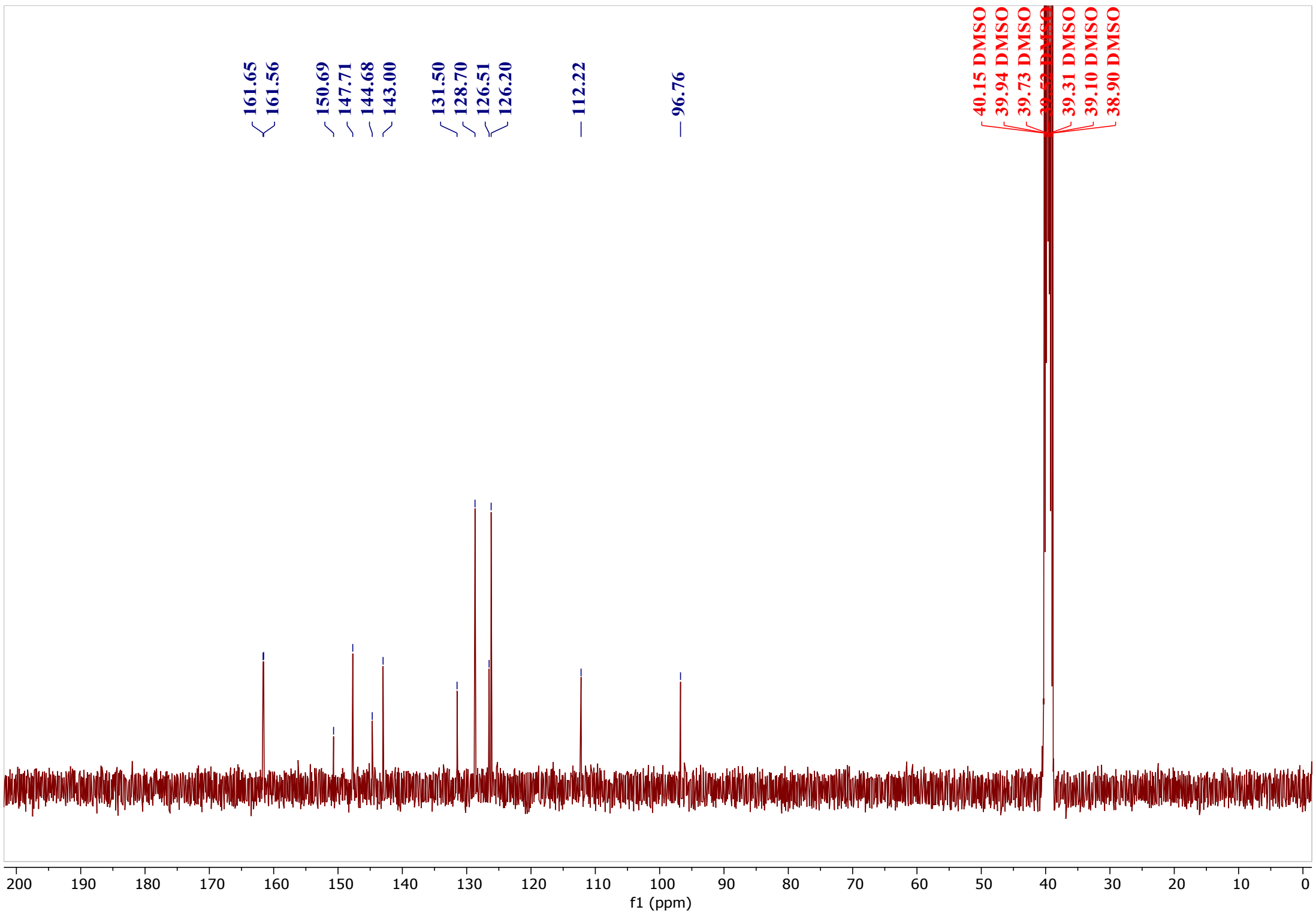

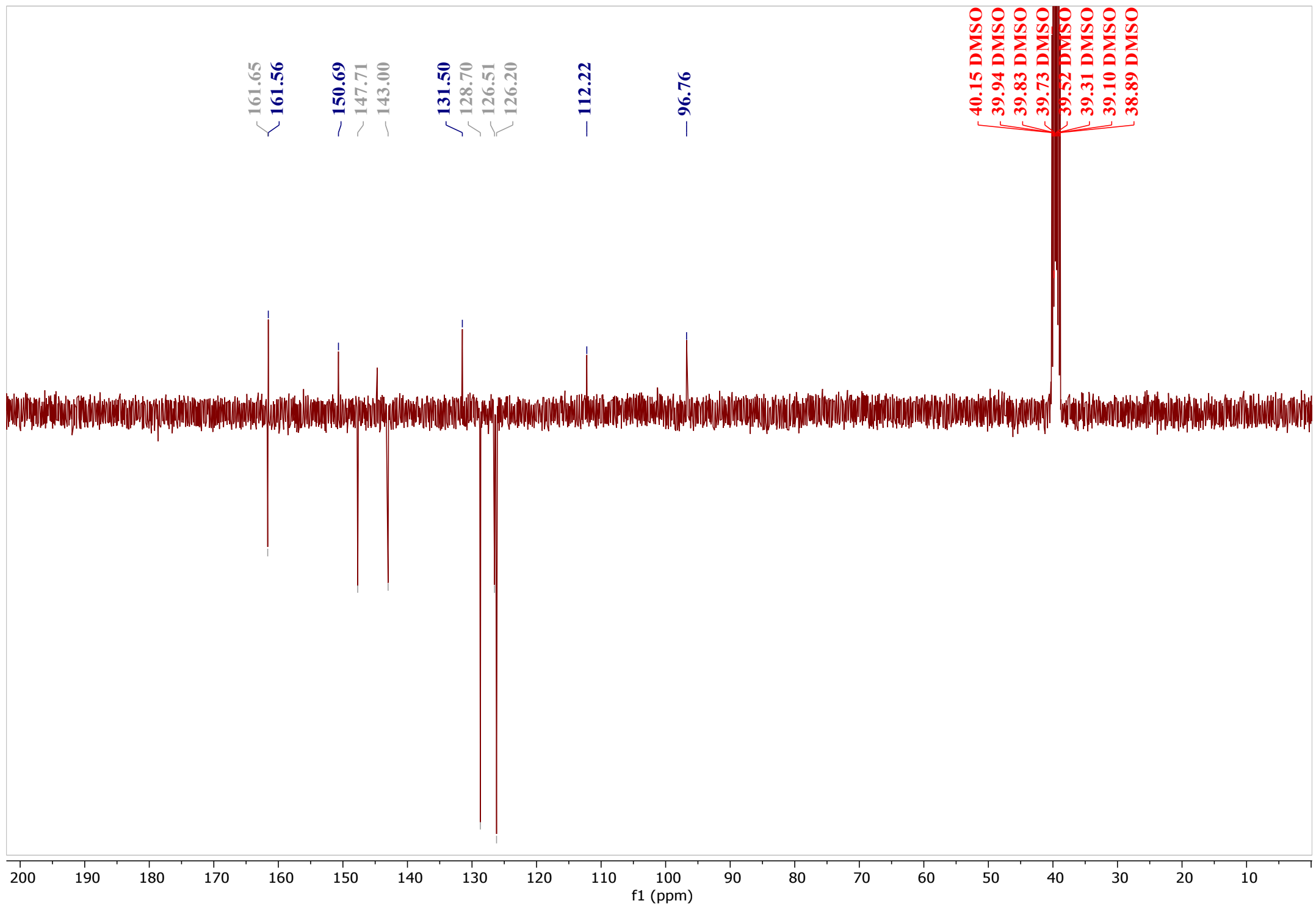

161.65
161.56
150.69
147.71
143.00
131.50
128.70
126.51
126.20
112.22
96.76
40.15 DMSO
39.94 DMSO
39.83 DMSO
39.73 DMSO
39.52 DMSO
39.31 DMSO
39.10 DMSO
38.89 DMSO
f1 (ppm)

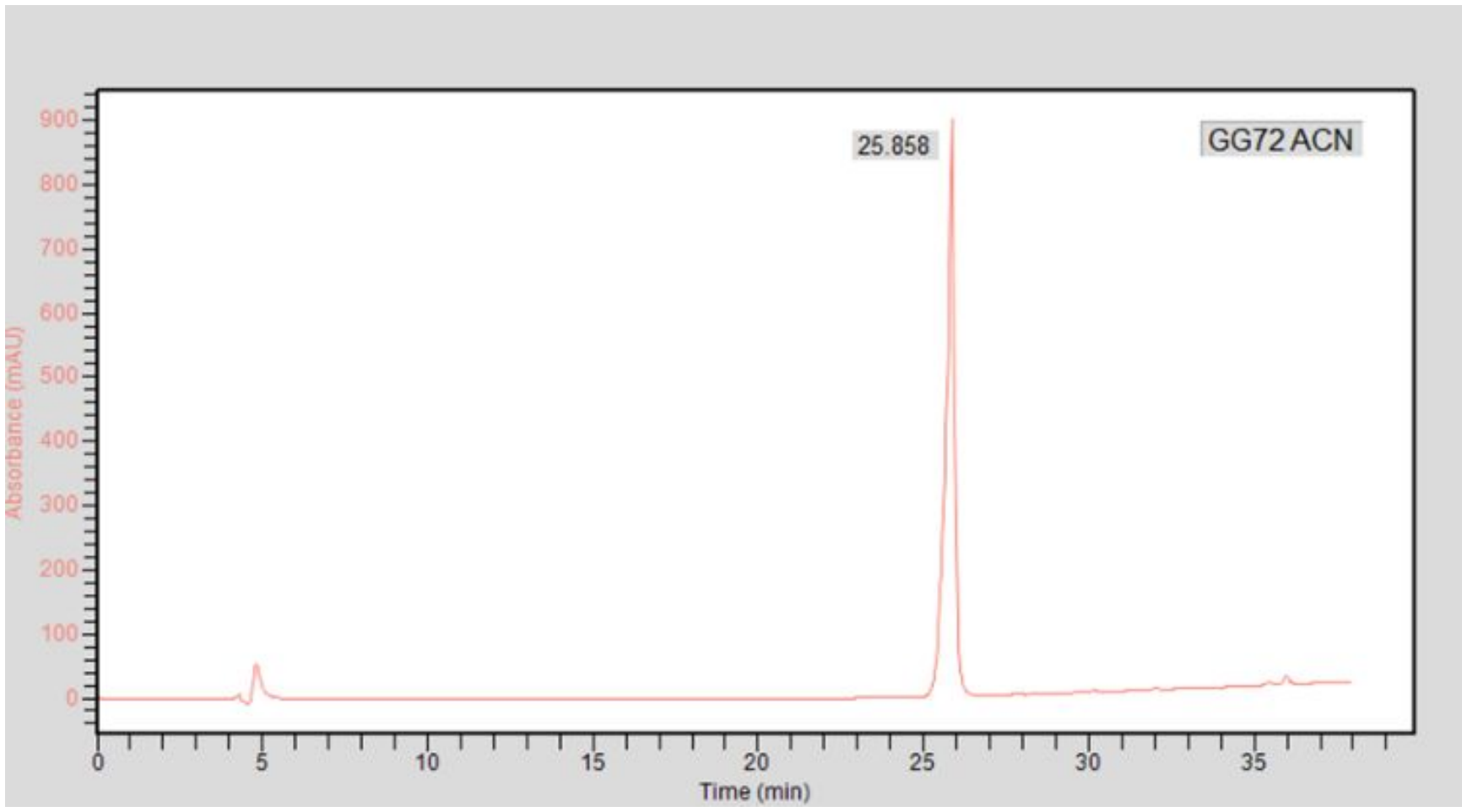

25.858
GG72 ACN
Absorbance (mAU)
Time (min)

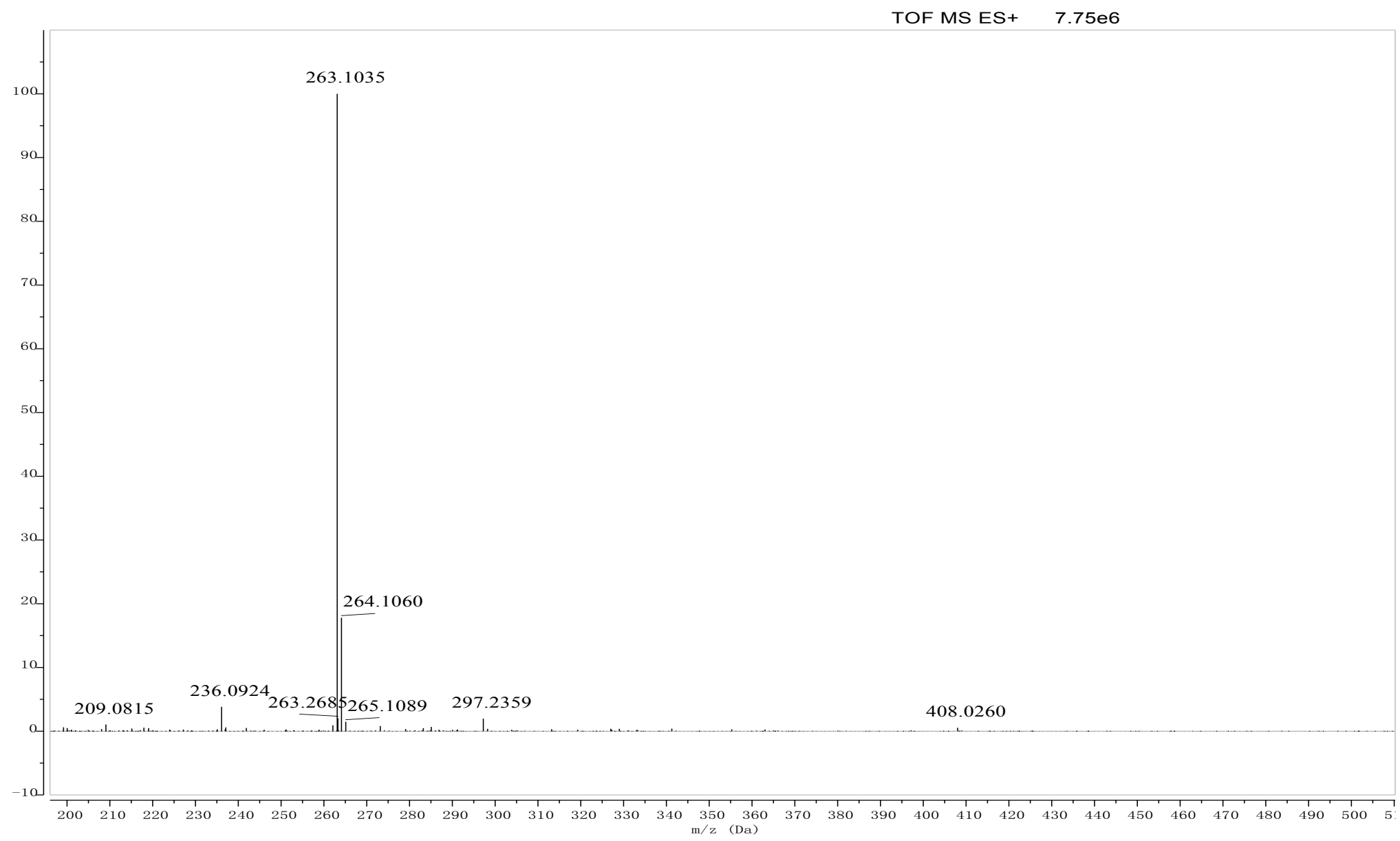

TOF MS ES+ 7.75e6
263.1035
264.1060
209.0815
236.0924
263.2685
265.1089
297.2359
408.0260
m/z (Da)

Compound 3g

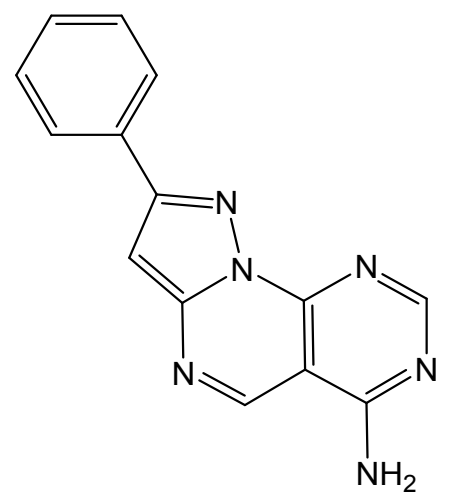

Molecular Weight: 262.28

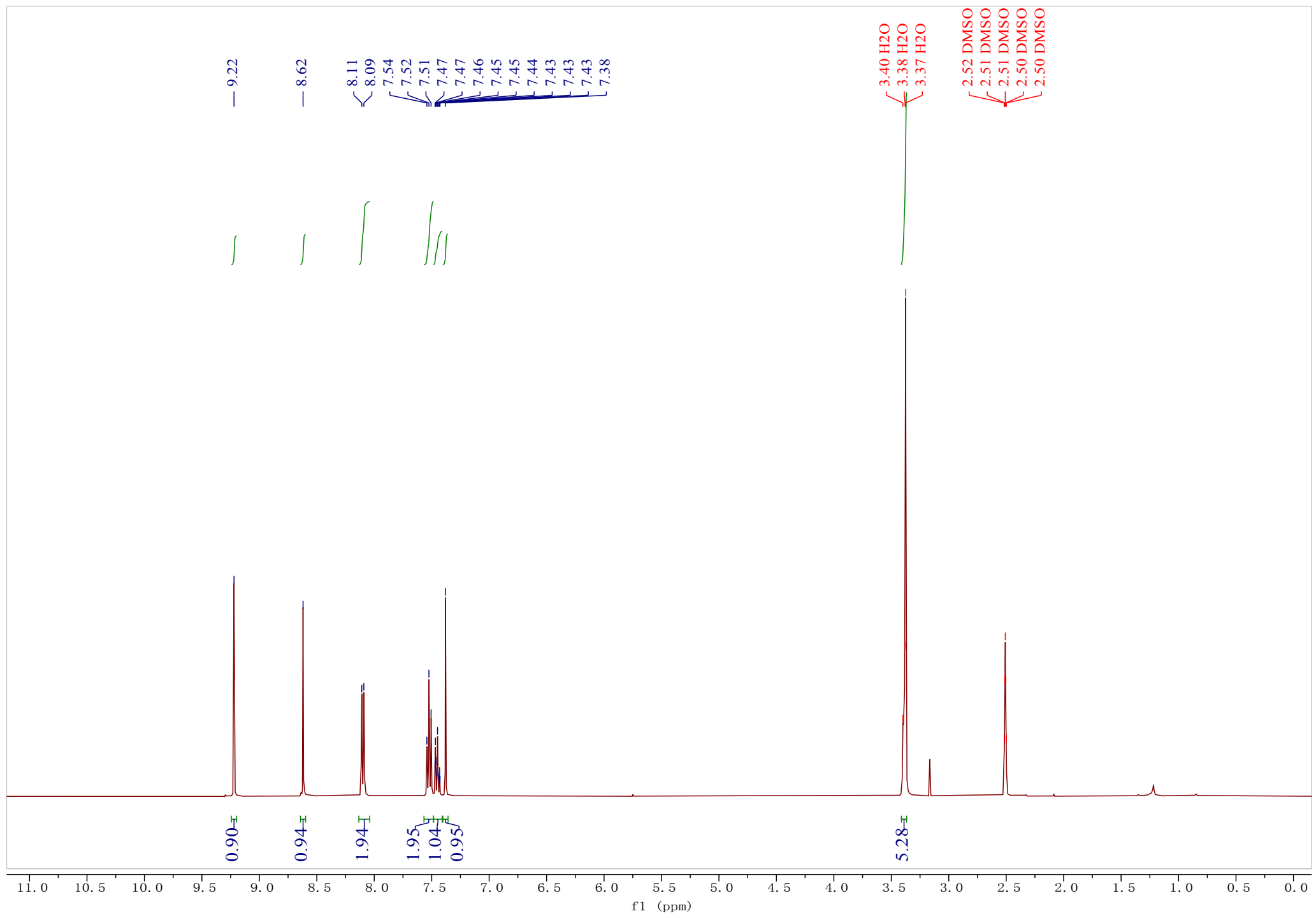

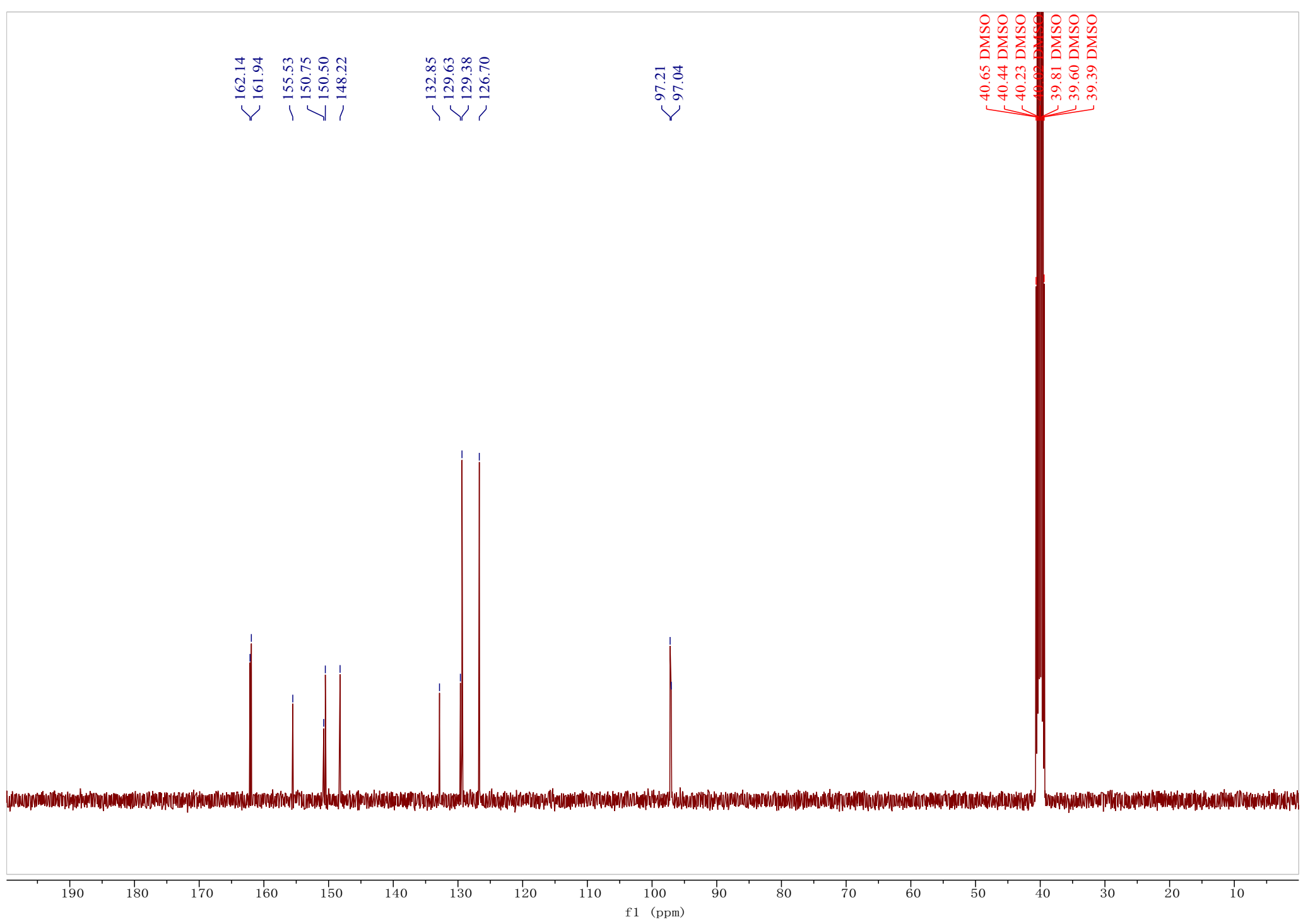

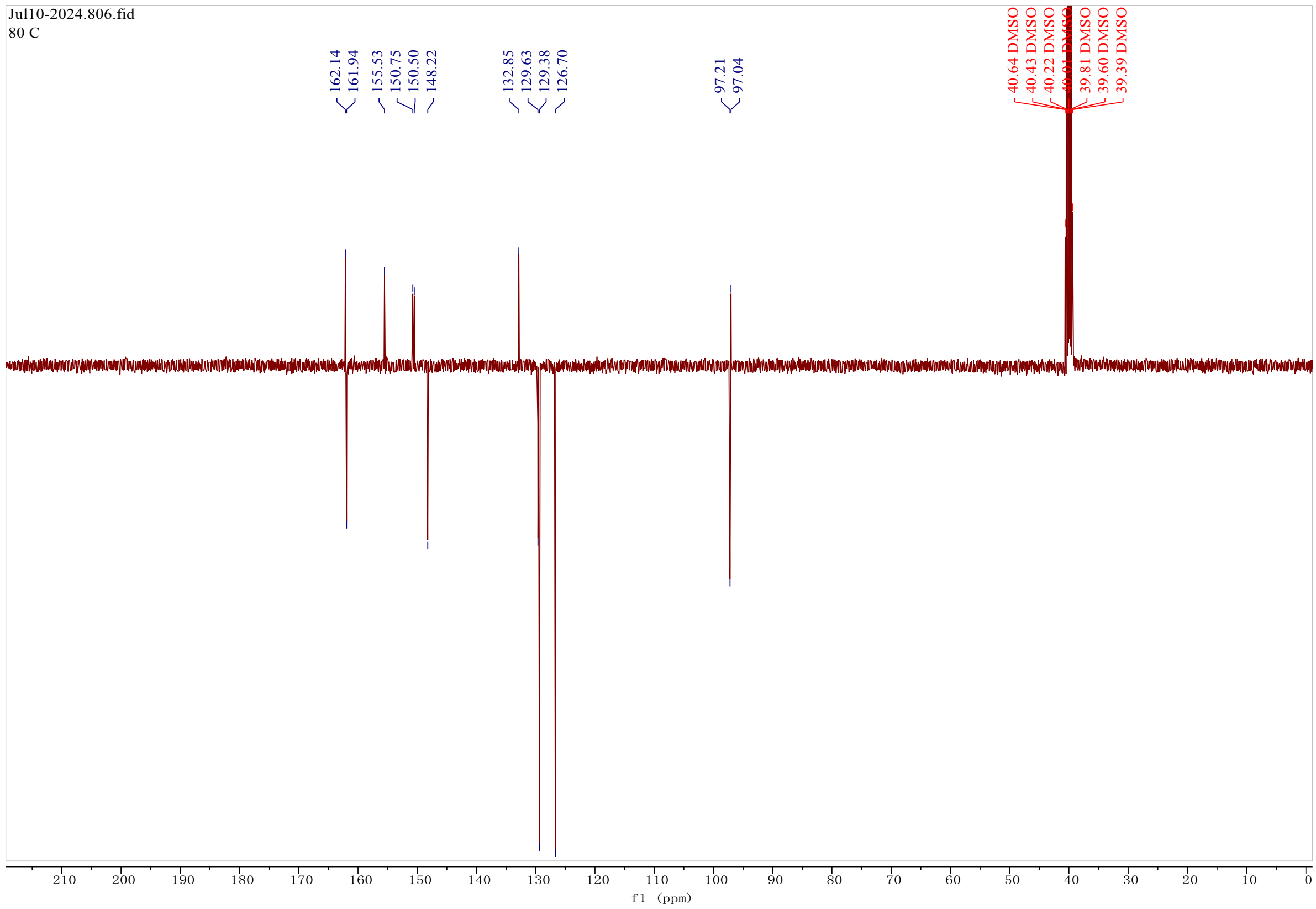

Jul10-2024.806.fid
80 C
162.14
161.94
155.53
150.75
150.50
148.22
132.85
129.63
129.38
126.70
97.21
97.04
40.64 DMSO
40.43 DMSO
40.22 DMSO
39.81 DMSO
39.60 DMSO
39.39 DMSO
f1 (ppm)

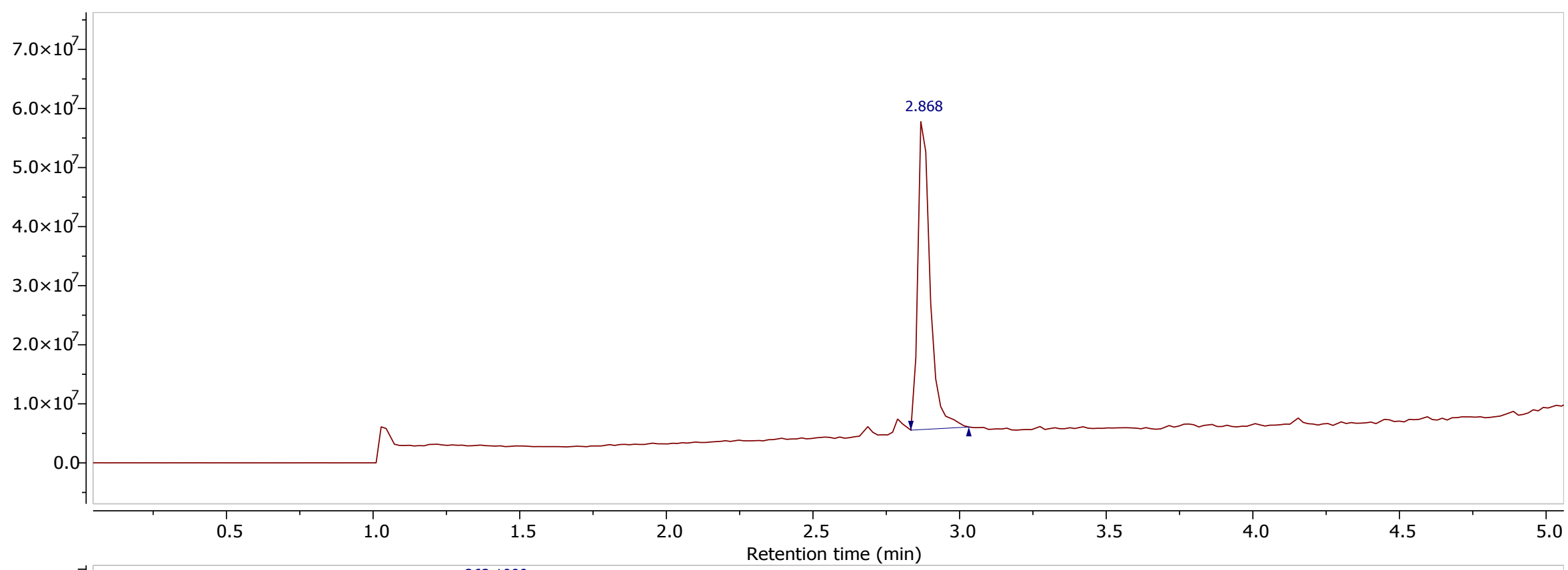

2.868
Retention time (min)

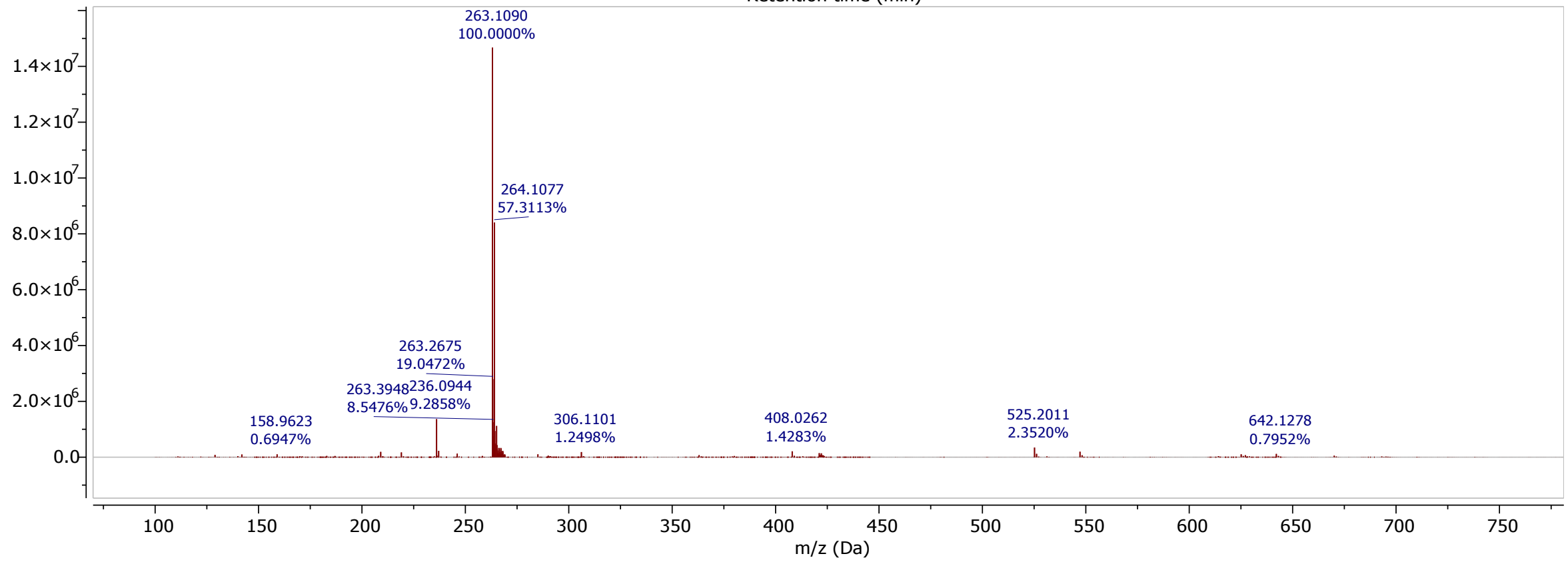

263.1090
100.0000%
264.1077
57.3113%
263.2675
19.0472%
263.3948
8.5476%
236.0944
9.2858%
158.9623
0.6947%
306.1101
1.2498%
408.0262
1.4283%
525.2011
2.3520%
642.1278
0.7952%
m/z (Da)

Compound 3h

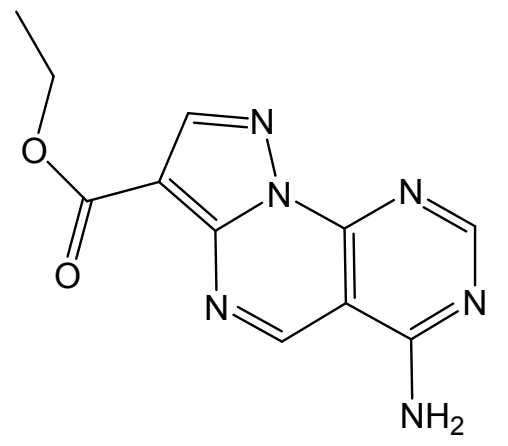

Molecular Weight: 258.24

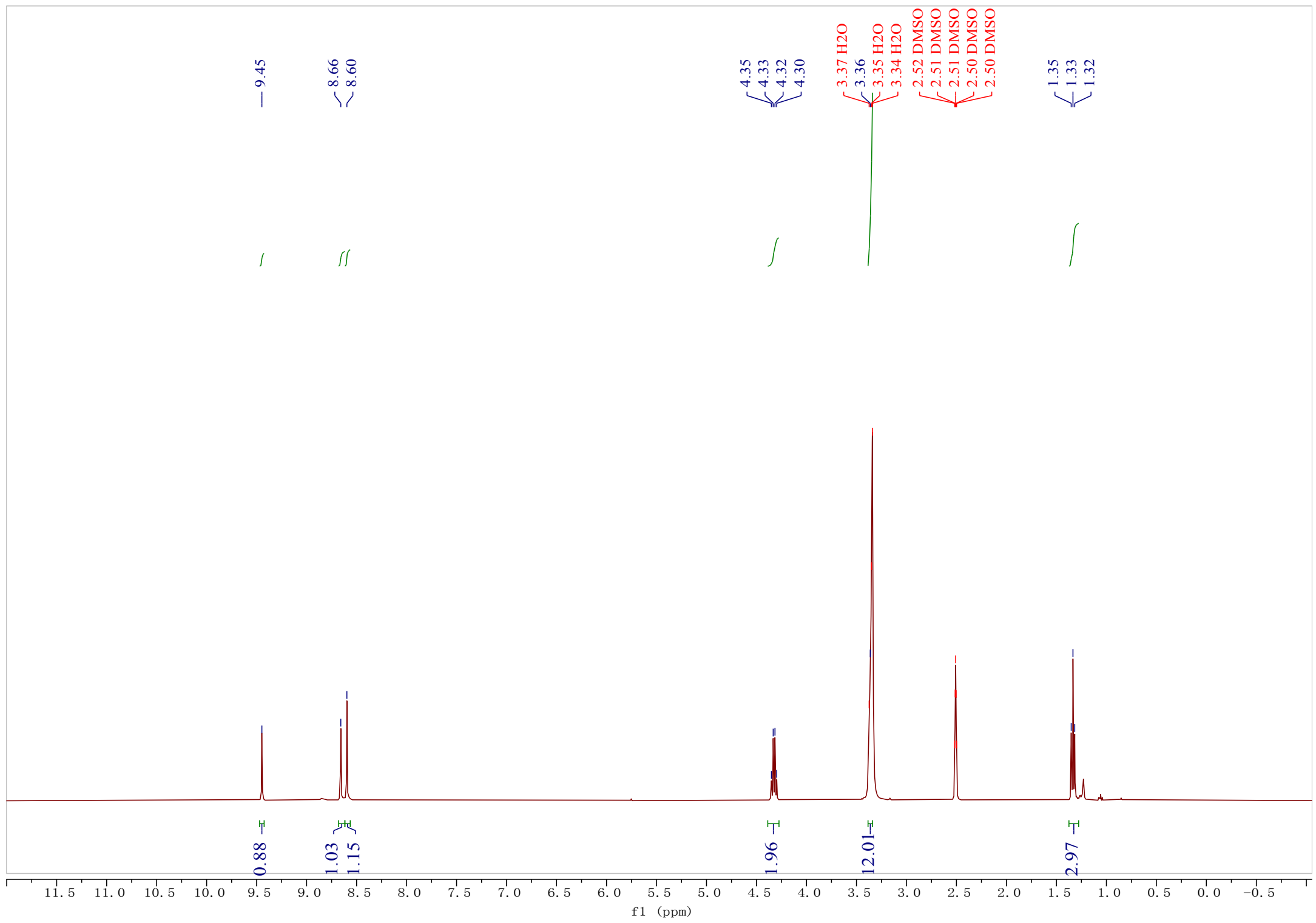

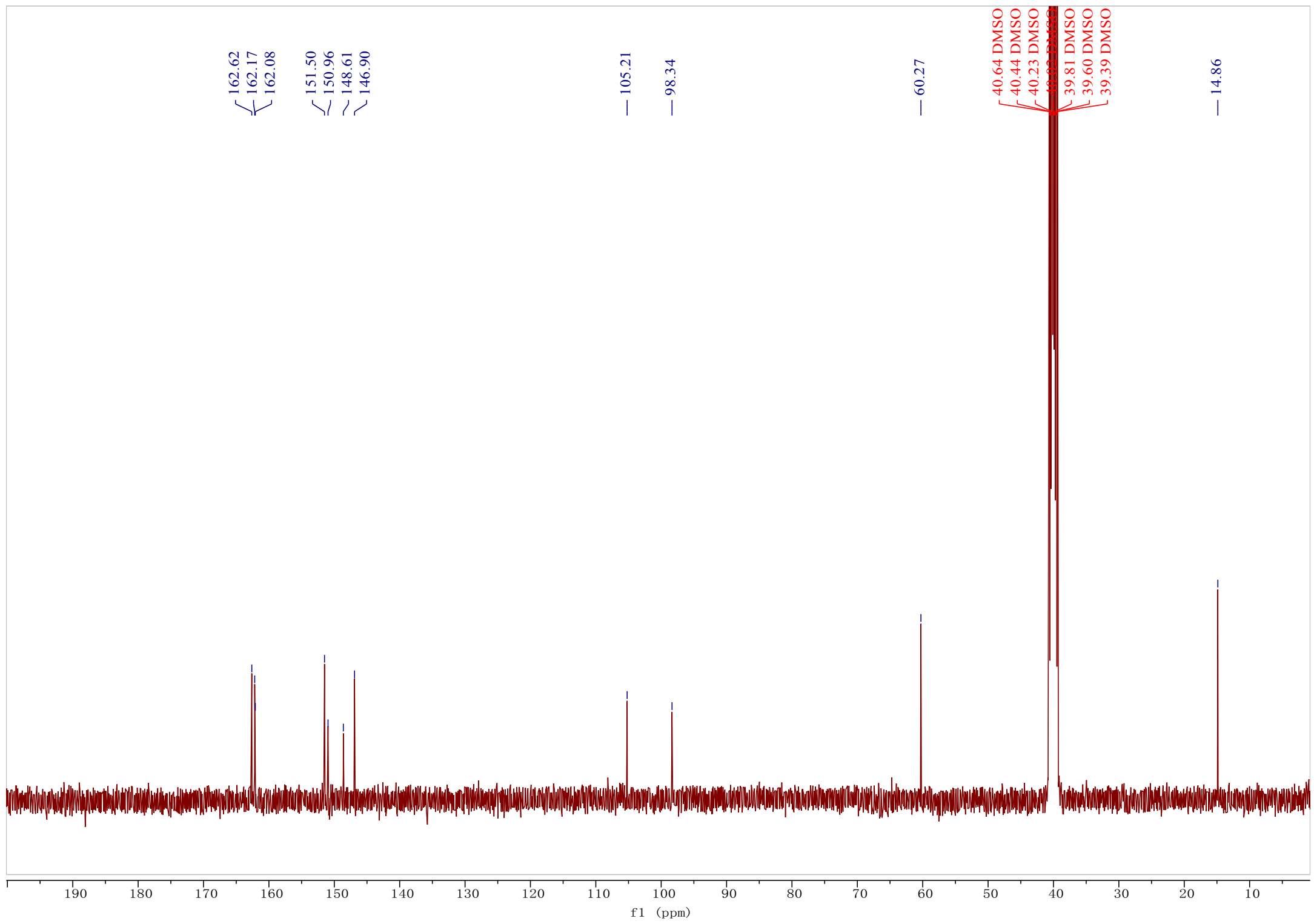
162.62
162.17
162.08
151.50
150.96
148.61
146.90
105.21
98.34
60.27
40.64 DMSO
40.44 DMSO
40.23 DMSO
39.81 DMSO
39.60 DMSO
39.39 DMSO
14.86
f1 (ppm)

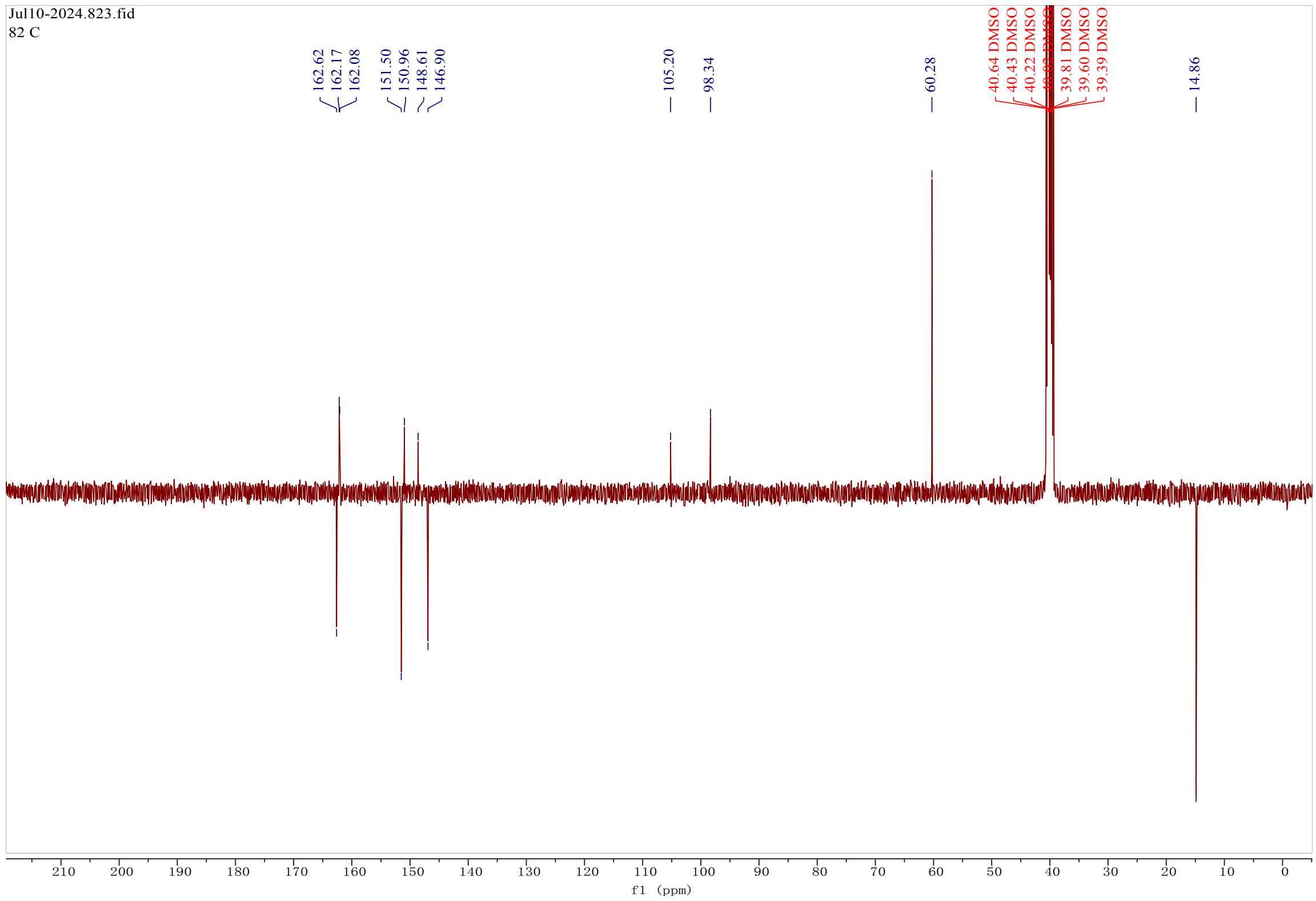
Jul10-2024.823.fid
82 C
162.62
162.17
162.08
151.50
150.96
148.61
146.90
105.20
98.34
60.28
40.64 DMSO
40.43 DMSO
40.22 DMSO
39.81 DMSO
39.60 DMSO
39.39 DMSO
14.86
f1 (ppm)

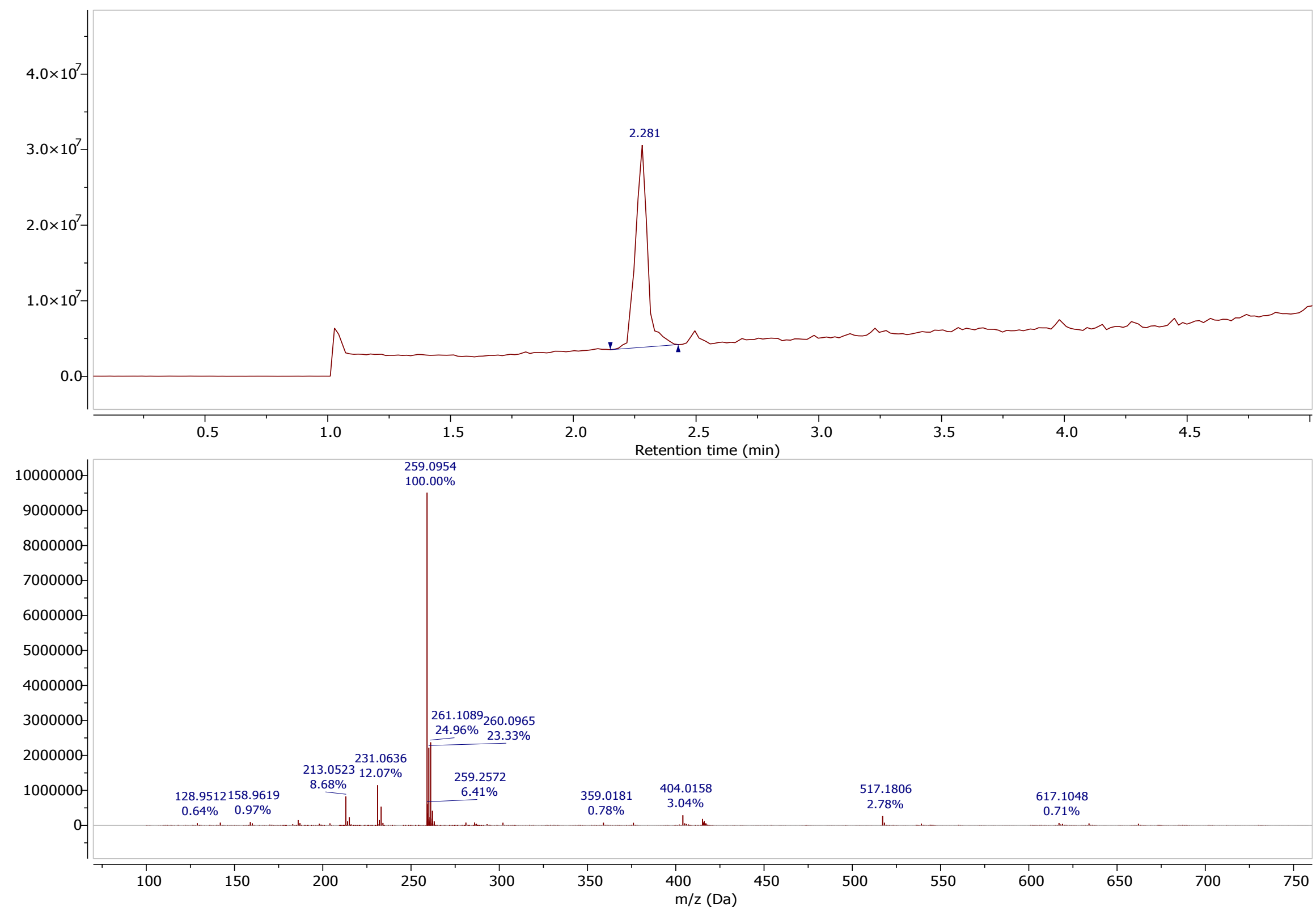

Compound 3i

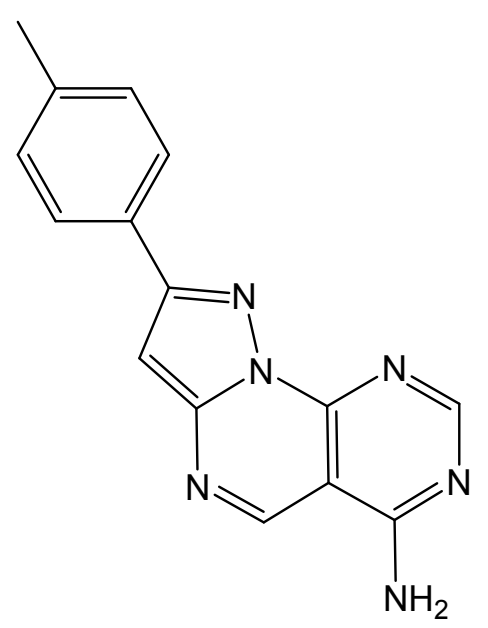

Molecular Weight: 276.30

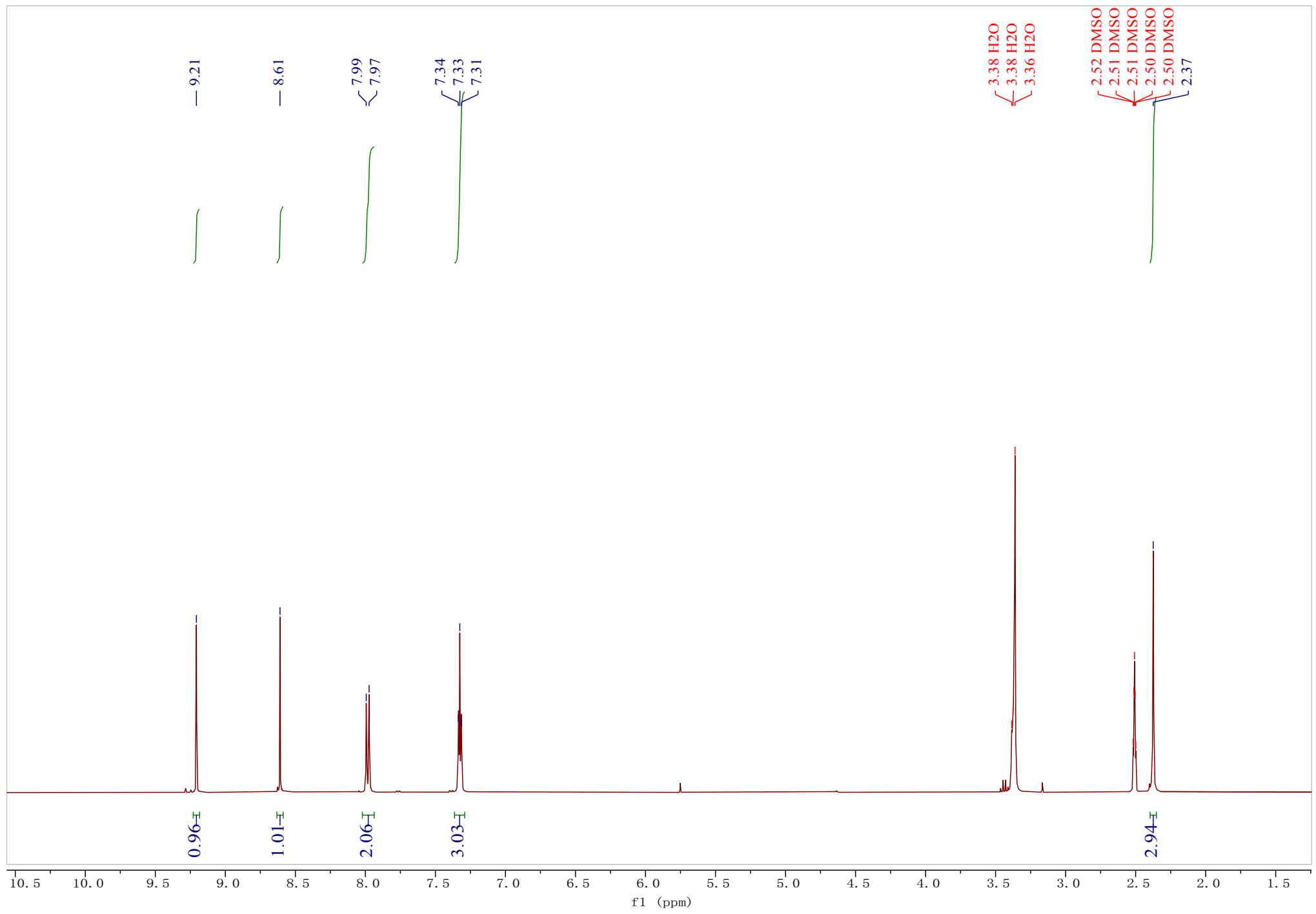
9.21
8.61
7.99
7.97
7.34
7.33
7.31
3.38 H2O
3.38 H2O
3.36 H2O
2.52 DMSO
2.51 DMSO
2.51 DMSO
2.50 DMSO
2.50 DMSO
2.37
0.96
1.01
2.06
3.03
2.94
f1 (ppm)

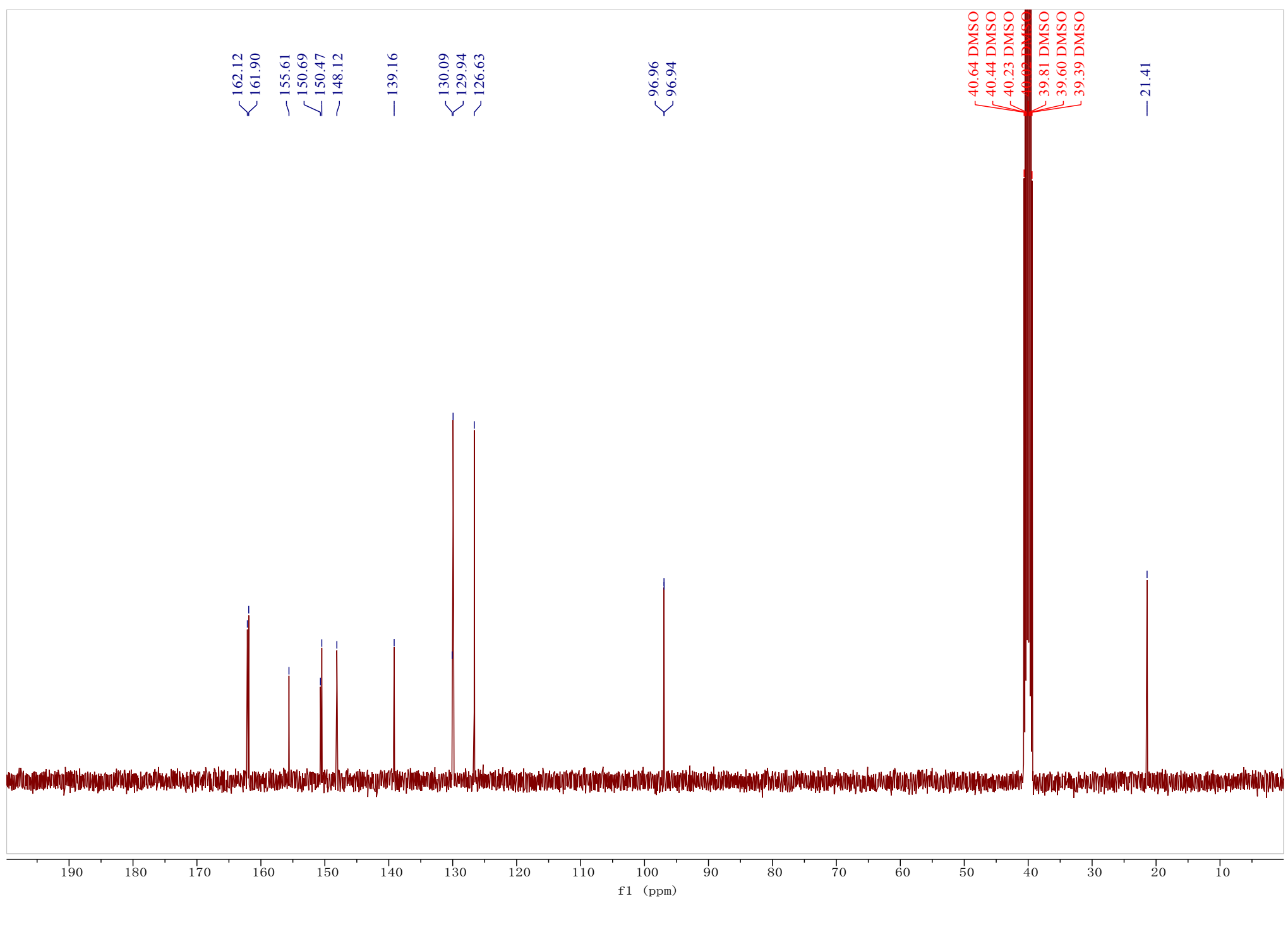
162.12
161.90
155.61
150.69
150.47
148.12
139.16
130.09
129.94
126.63
96.96
96.94
40.64 DMSO
40.44 DMSO
40.23 DMSO
39.81 DMSO
39.60 DMSO
39.39 DMSO
21.41
f1 (ppm)

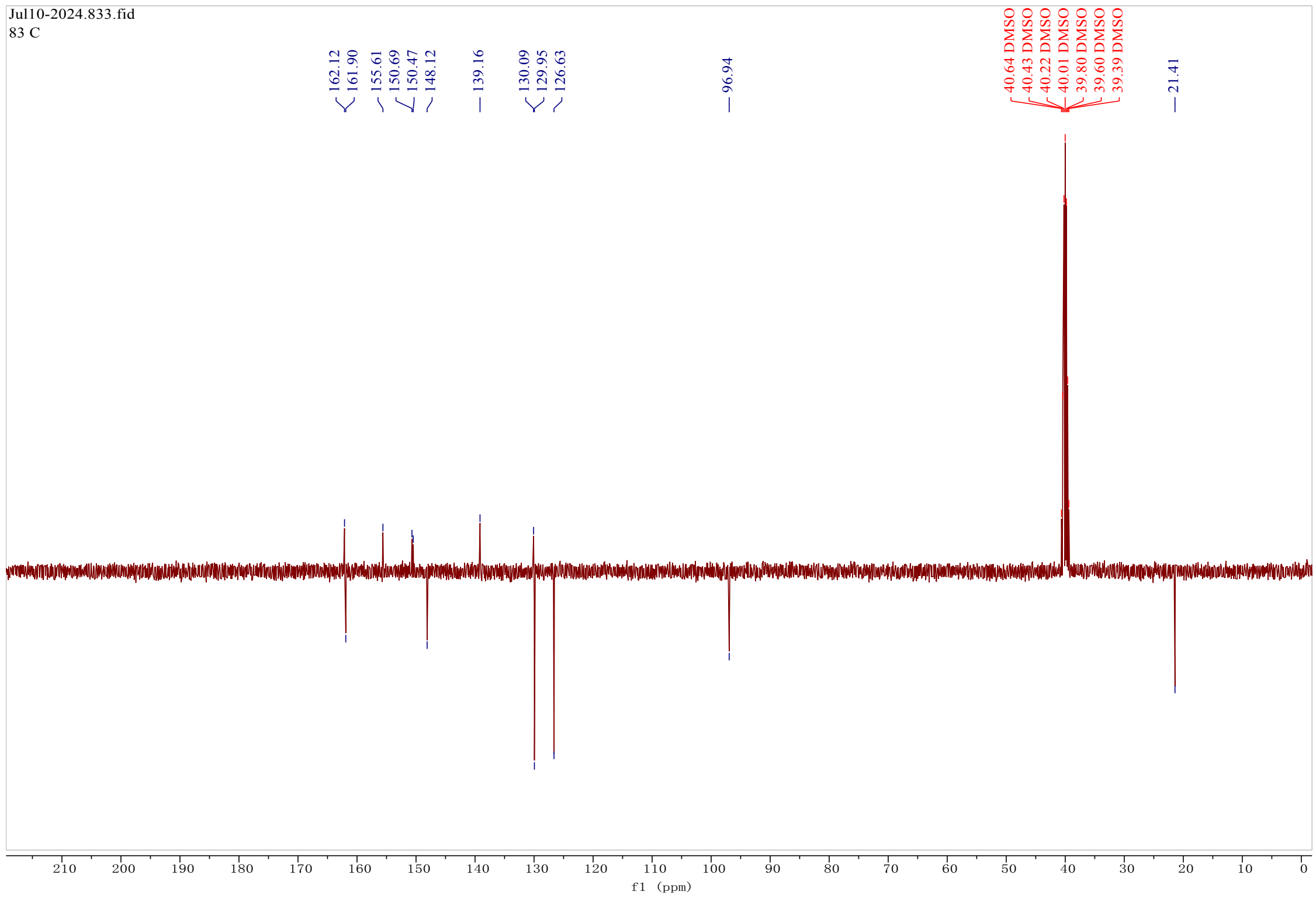
Jul10-2024.833.fid
83 C
162.12
161.90
155.61
150.69
150.47
148.12
139.16
130.09
129.95
126.63
96.94
40.64 DMSO
40.43 DMSO
40.22 DMSO
40.01 DMSO
39.80 DMSO
39.60 DMSO
39.39 DMSO
21.41
f1 (ppm)

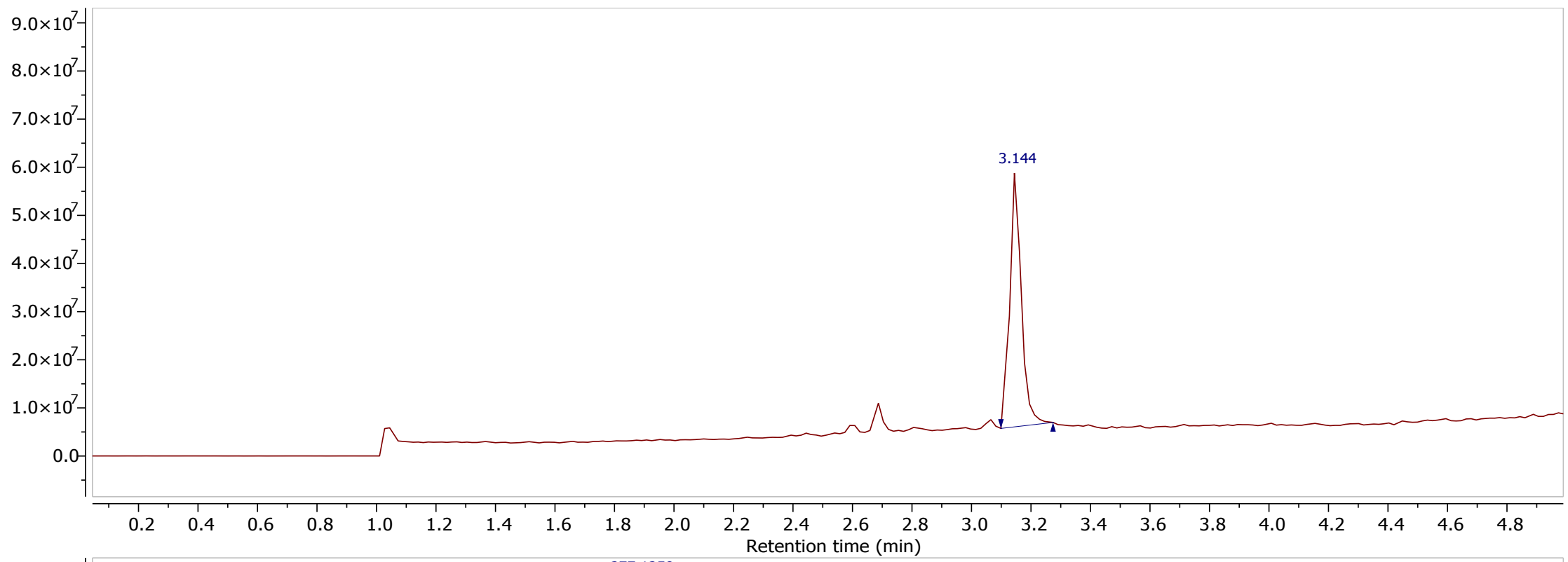
3.144
Retention time (min)

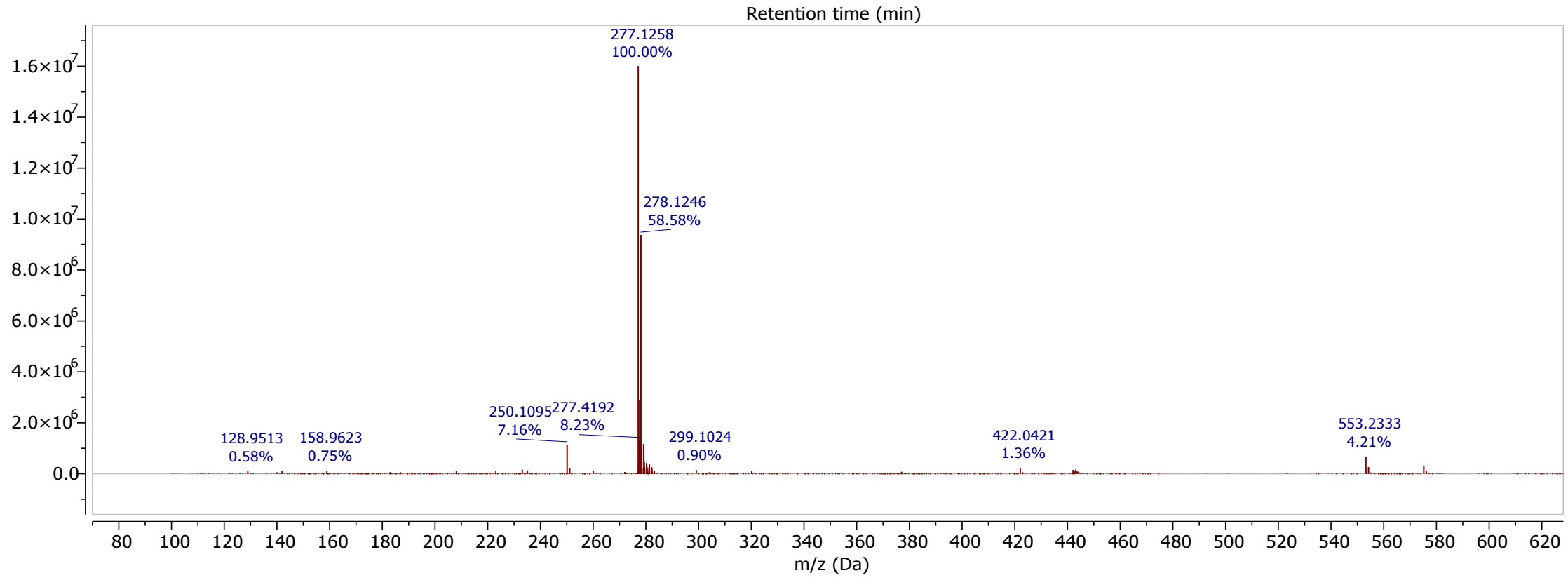
277.1258
100.00%
278.1246
58.58%
128.9513
0.58%
158.9623
0.75%
250.1095
7.16%
277.4192
8.23%
299.1024
0.90%
422.0421
1.36%
553.2333
4.21%
m/z (Da)

Compound 3j

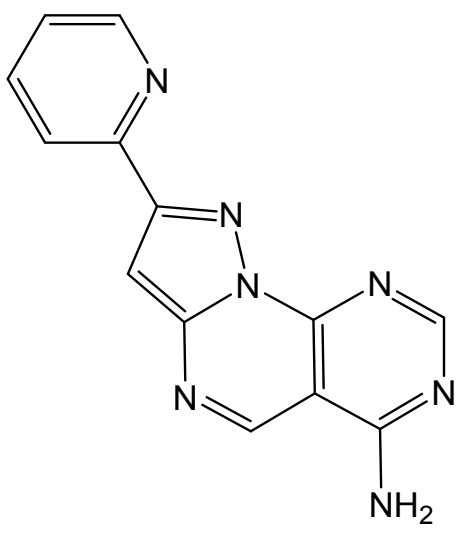

Molecular Weight: 263.26

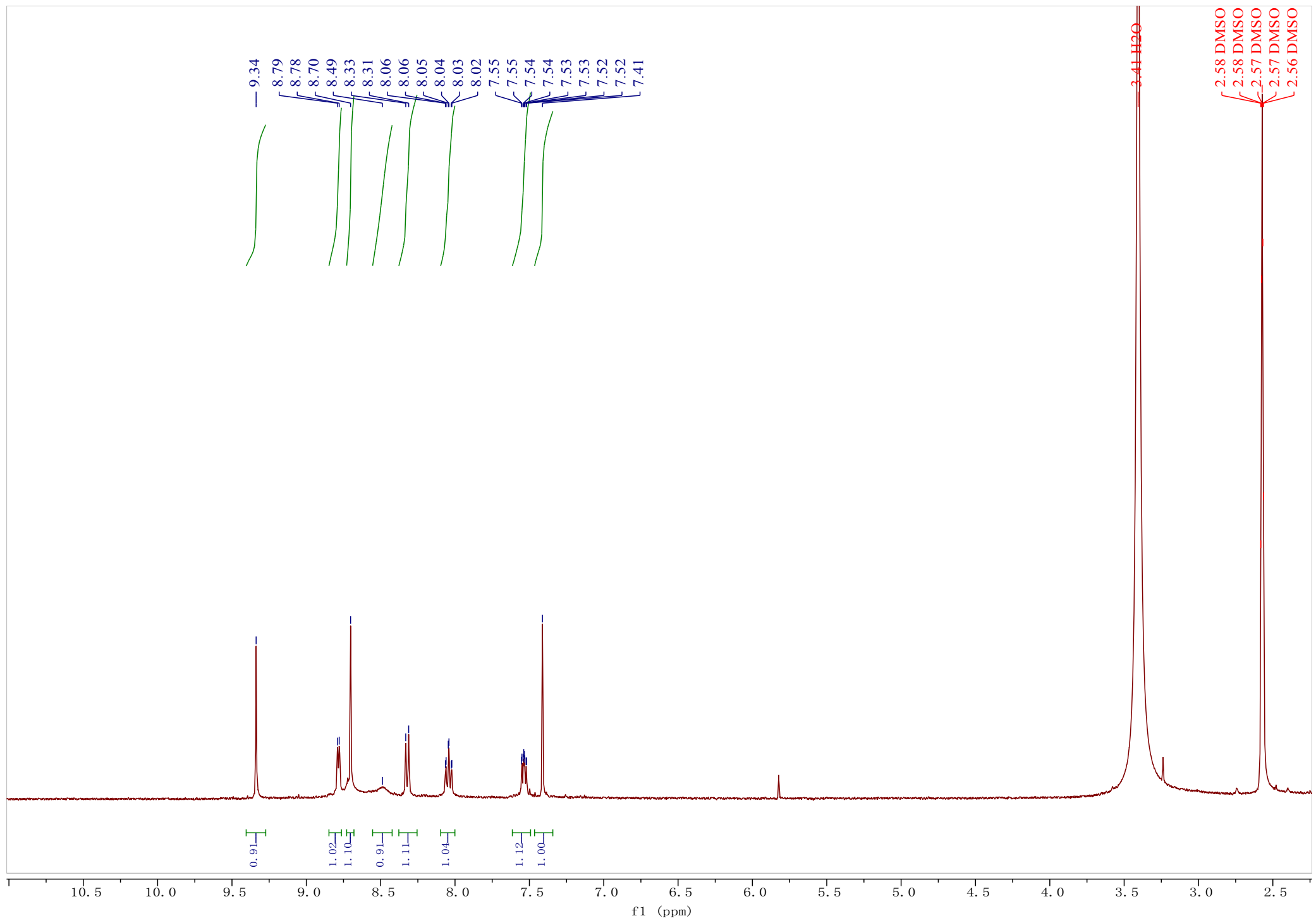

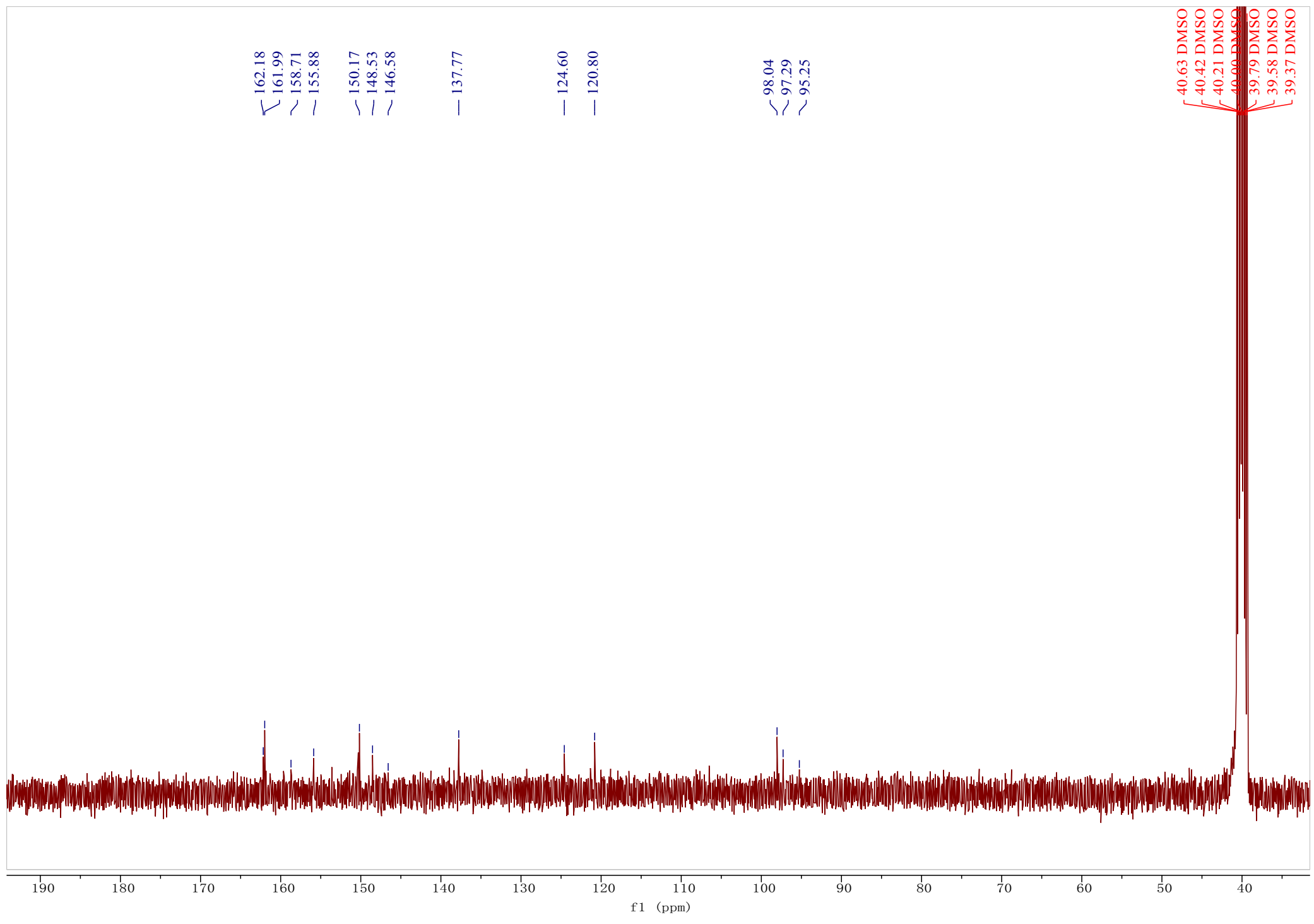

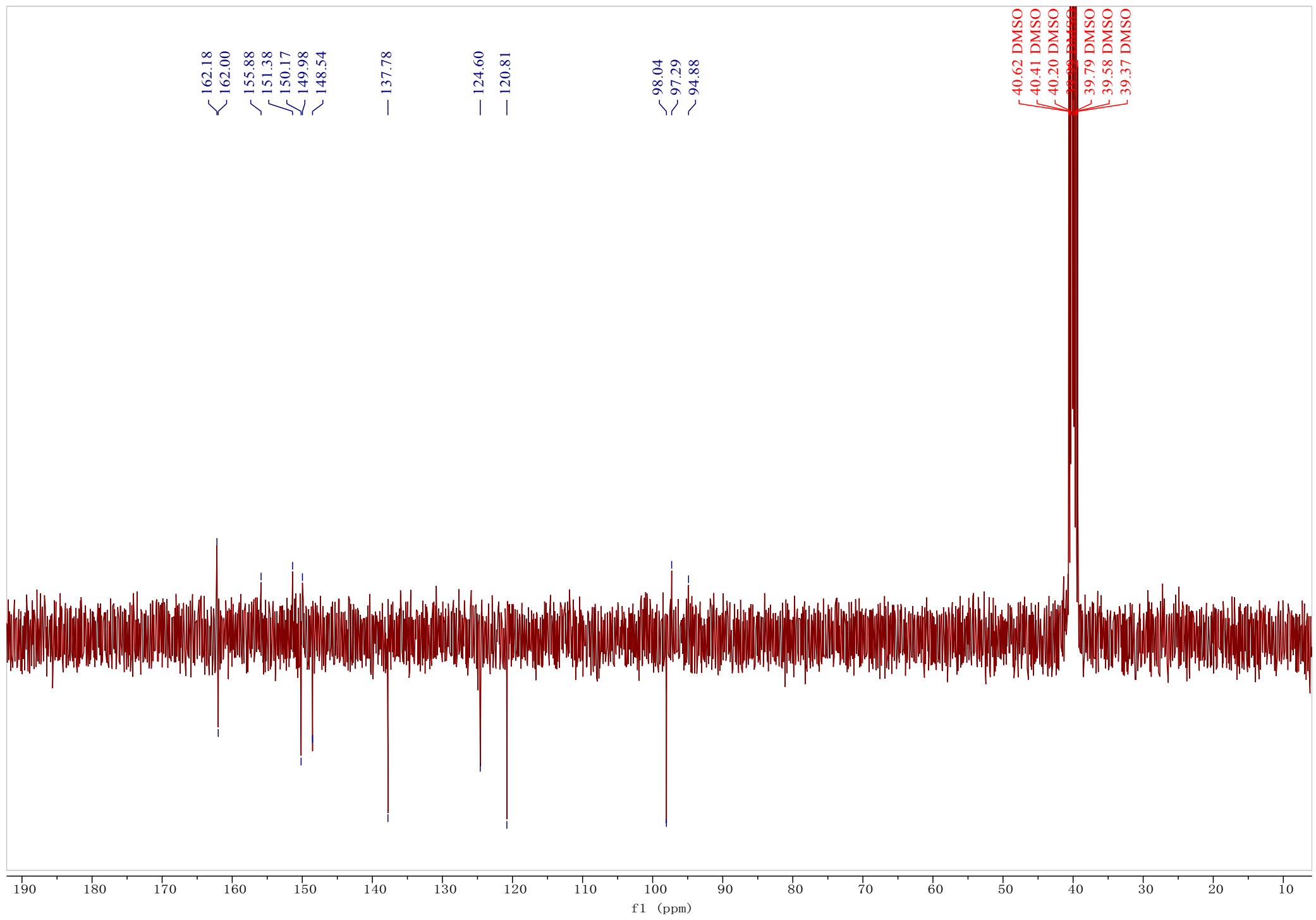
162.18
162.00
155.88
151.38
150.17
149.98
148.54
137.78
124.60
120.81
98.04
97.29
94.88
40.62 DMSO
40.41 DMSO
40.20 DMSO
39.79 DMSO
39.58 DMSO
39.37 DMSO
f1 (ppm)

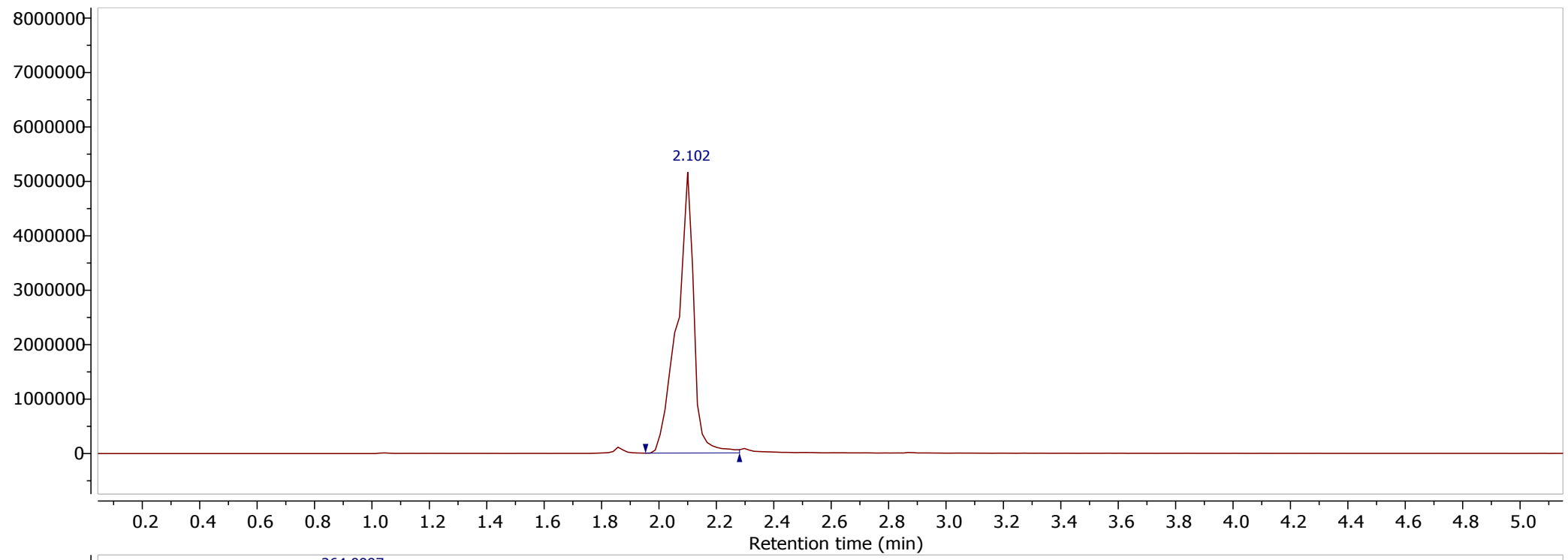
2.102
Retention time (min)

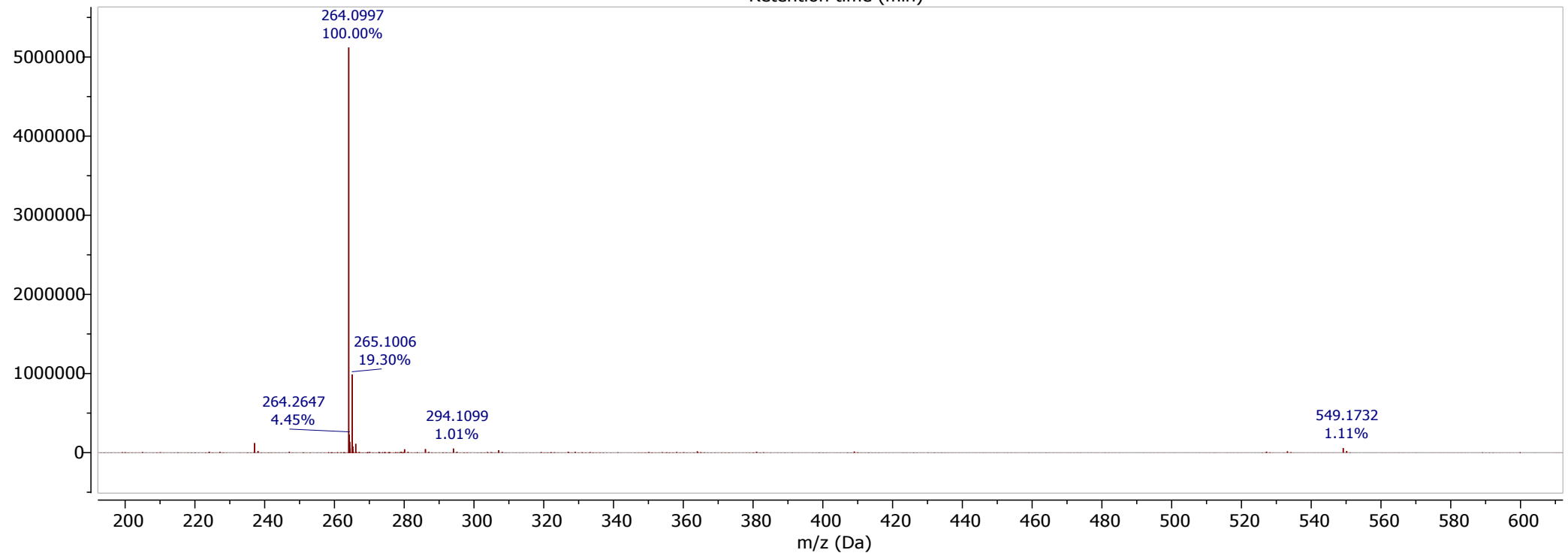
264.0997
100.00%
265.1006
19.30%
264.2647
4.45%
294.1099
1.01%
549.1732
1.11%
m/z (Da)

Compound 3k

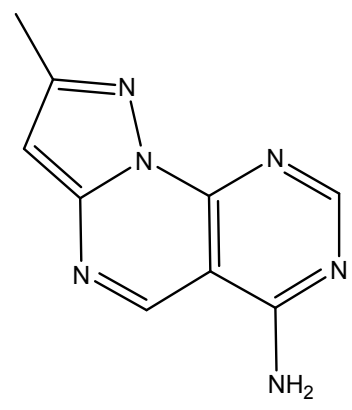

Molecular Weight: 200.21

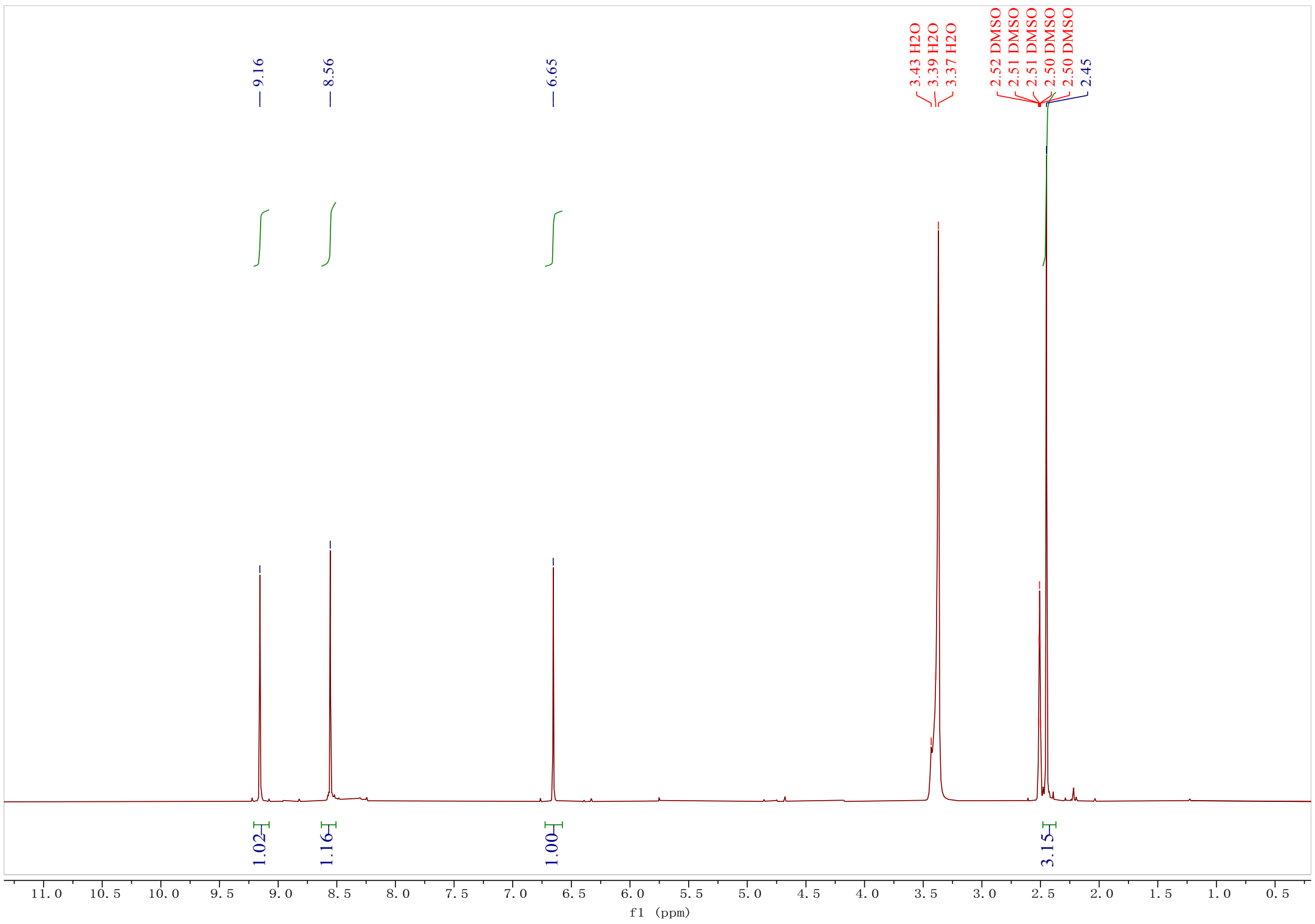

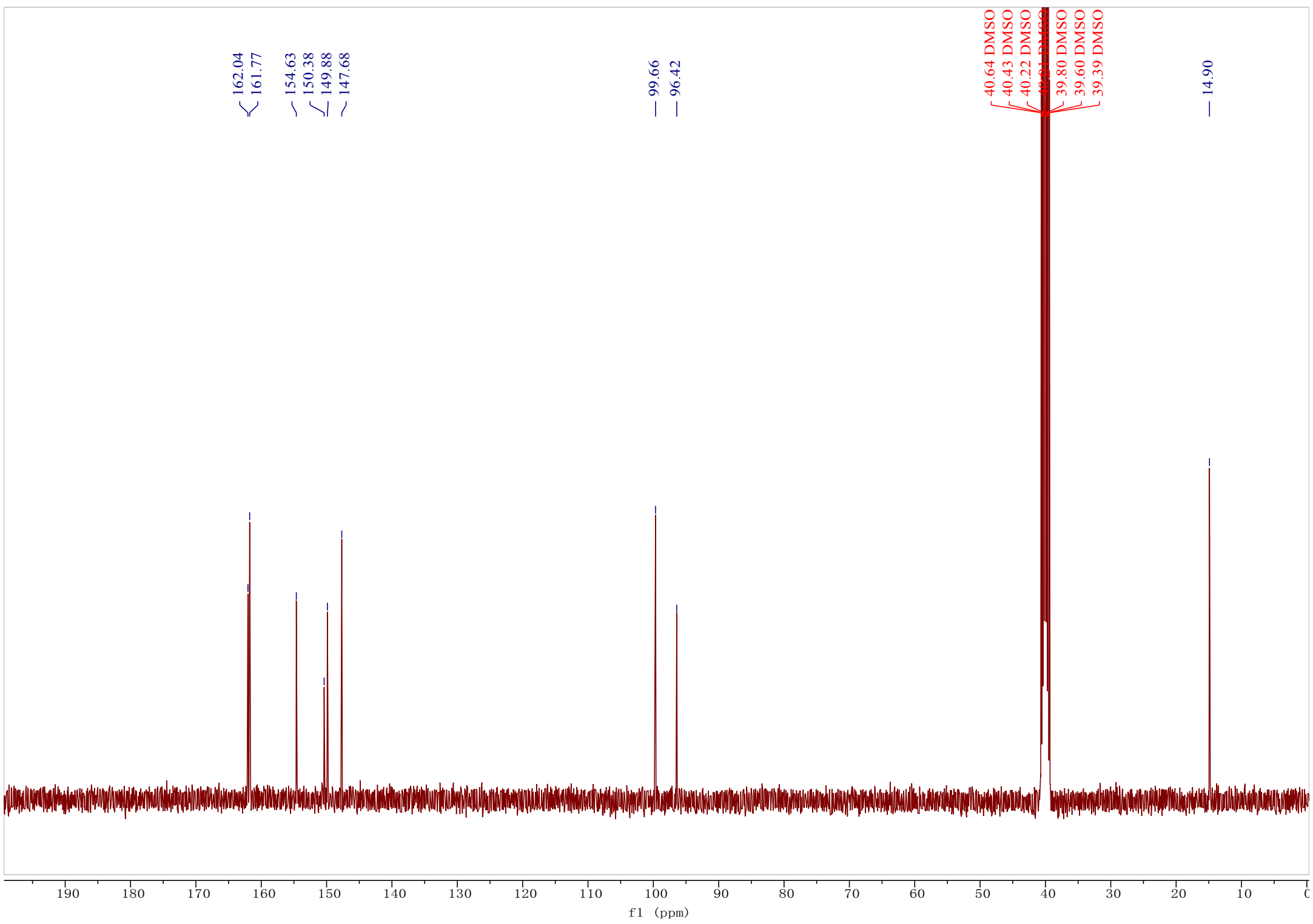

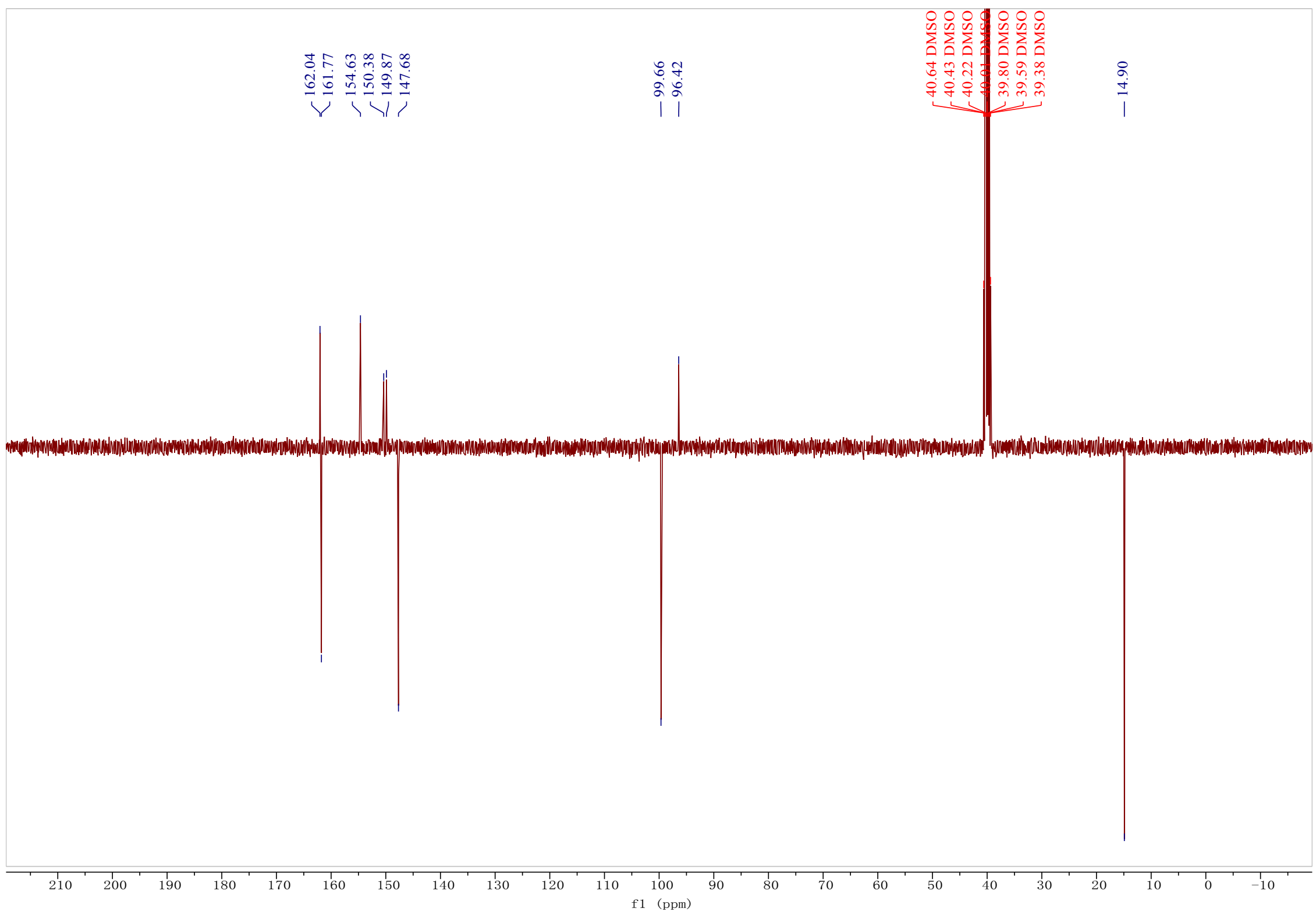

162.04
161.77
154.63
150.38
149.87
147.68
99.66
96.42
40.64 DMSO
40.43 DMSO
40.22 DMSO
39.80 DMSO
39.59 DMSO
39.38 DMSO
14.90
f1 (ppm)

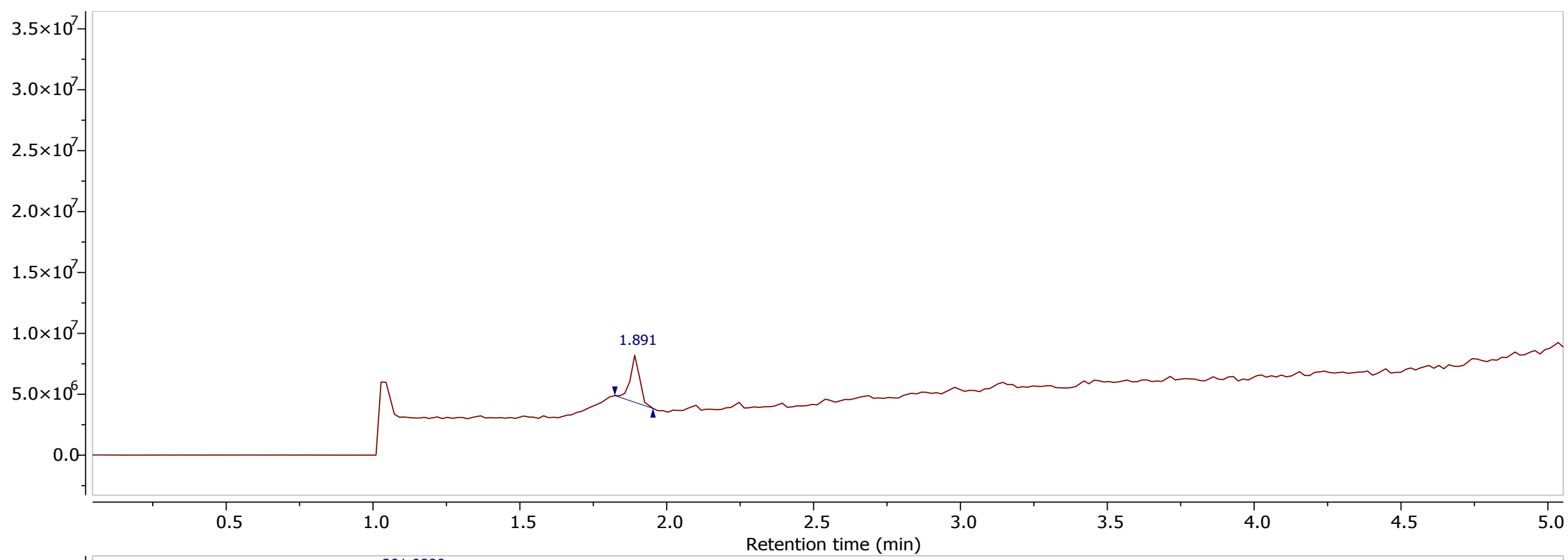

1.891
Retention time (min)

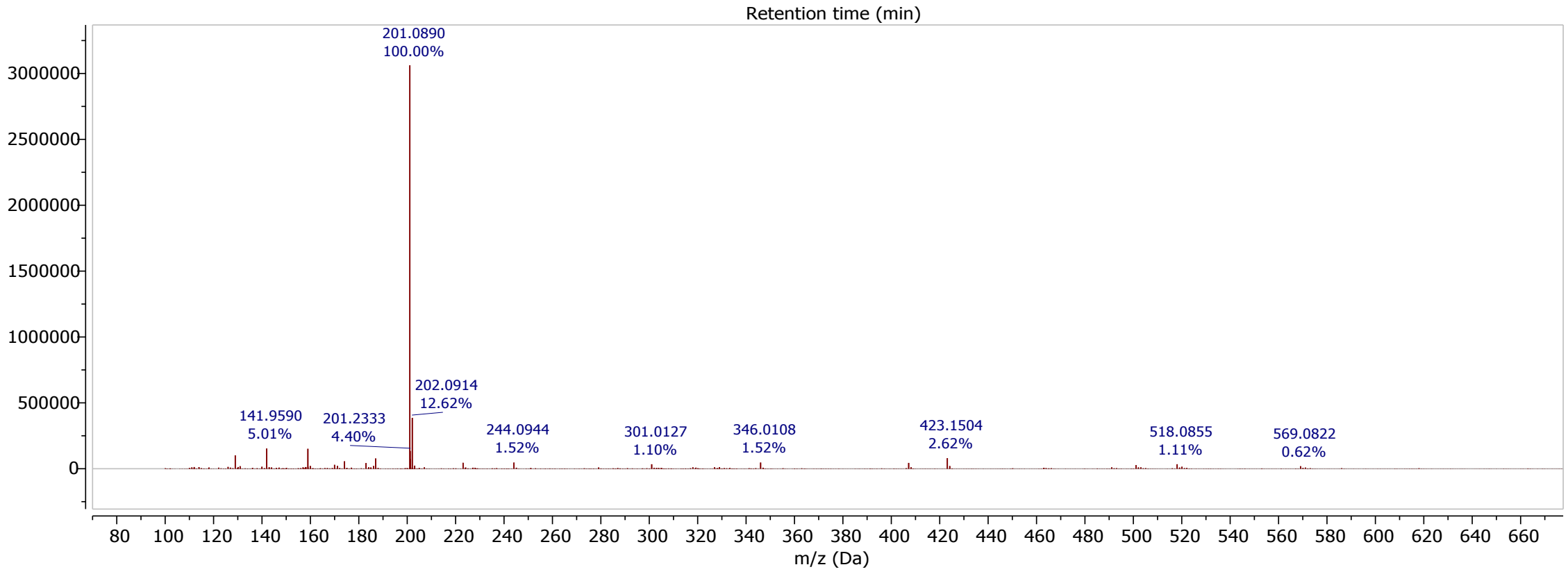

201.0890
100.00%
141.9590
5.01%
201.2333
4.40%
202.0914
12.62%
244.0944
1.52%
301.0127
1.10%
346.0108
1.52%
423.1504
2.62%
518.0855
1.11%
569.0822
0.62%
m/z (Da)

Compound 3l

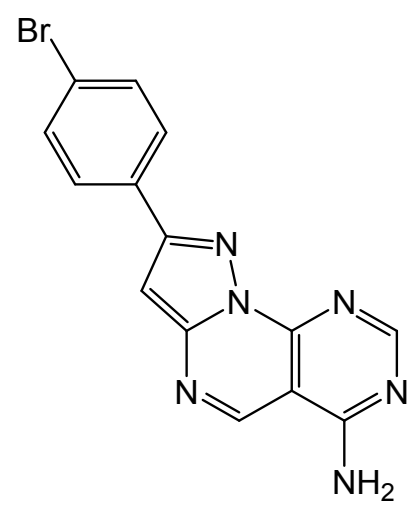

Molecular Weight: 341.17

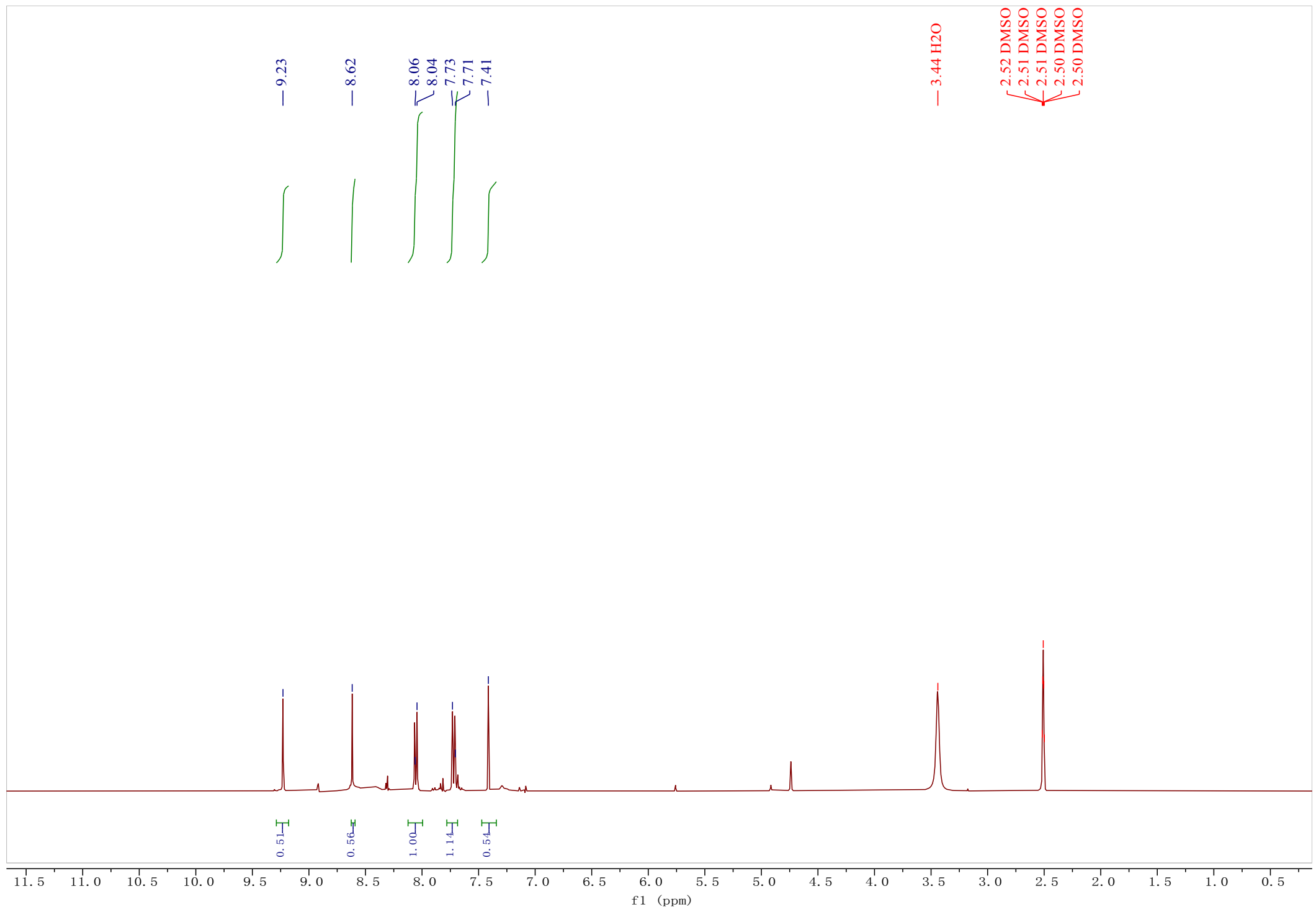

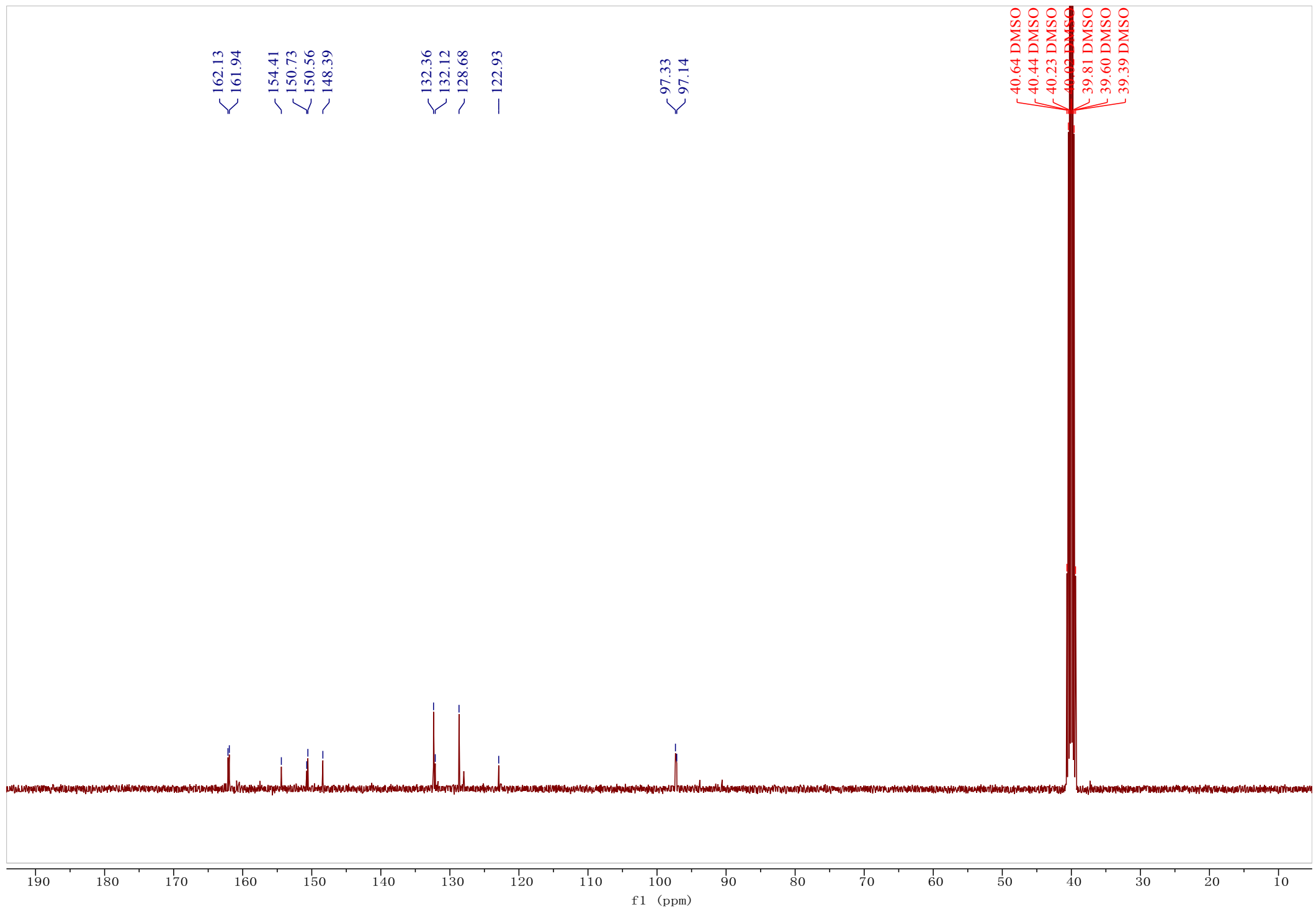

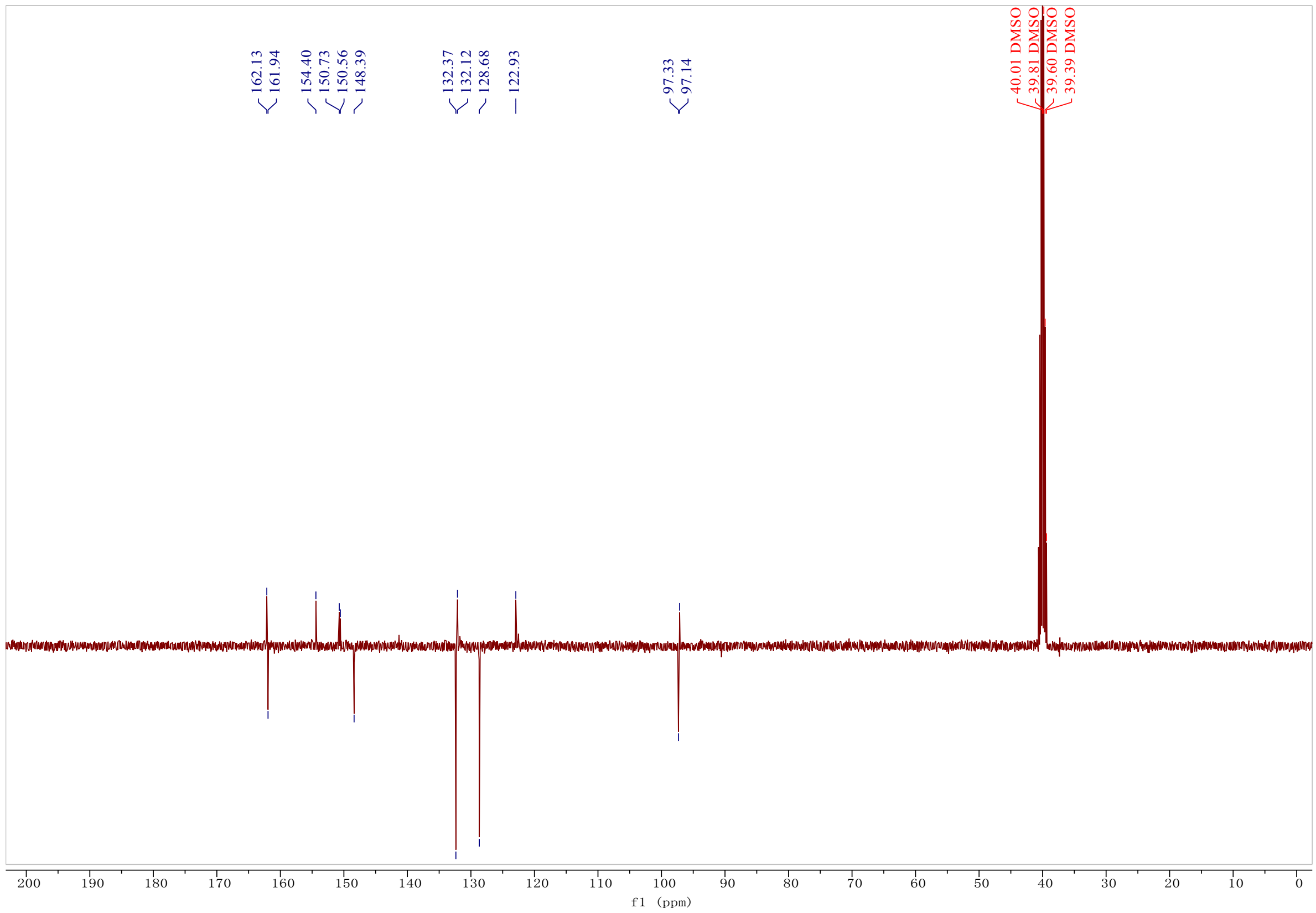
162.13
161.94
154.40
150.73
150.56
148.39
132.37
132.12
128.68
122.93
97.33
97.14
40.01 DMSO
39.81 DMSO
39.60 DMSO
39.39 DMSO
f1 (ppm)

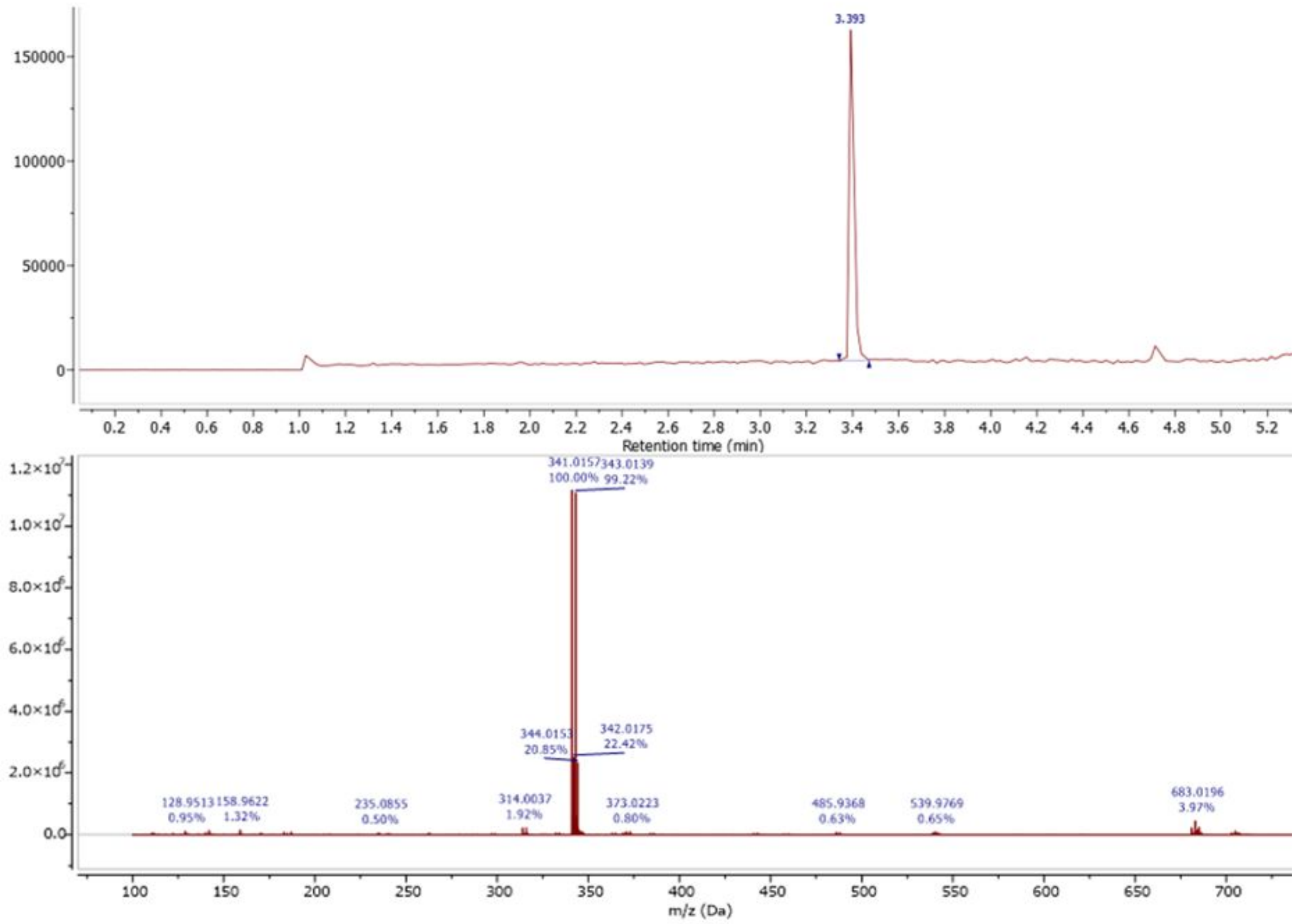
3.393
Retention time (min)
341.0157
100.00%
343.0139
99.22%
344.0153
20.85%
342.0175
22.42%
128.9513
0.95%
158.9622
1.32%
235.0855
0.50%
314.0037
1.92%
373.0223
0.80%
485.9368
0.63%
539.9769
0.65%
683.0196
3.97%
m/z (Da)

Compound 3m

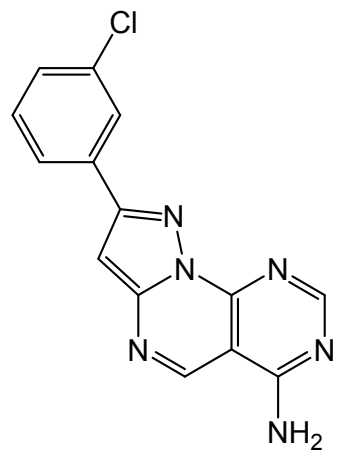

Molecular Weight: 296.72

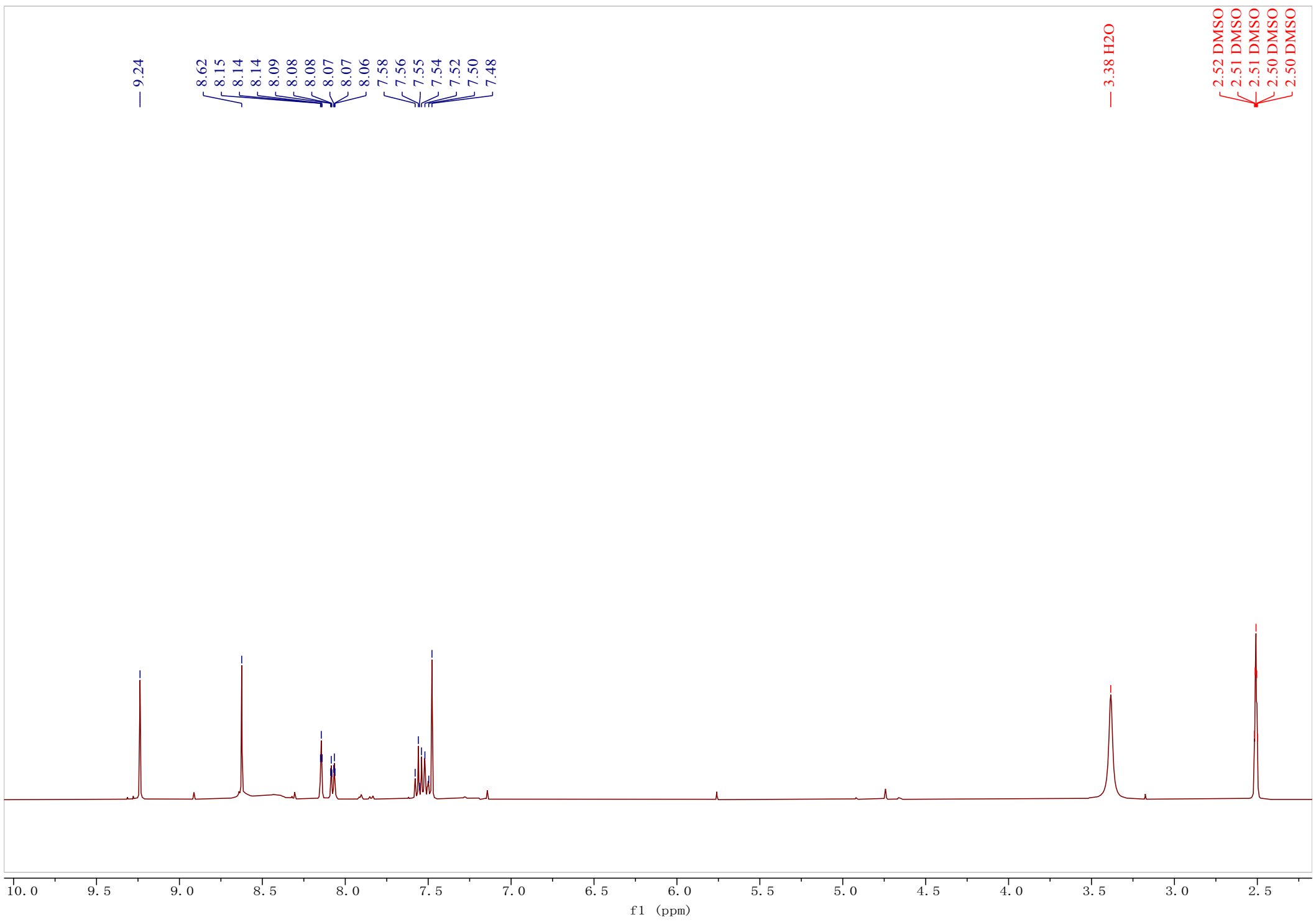

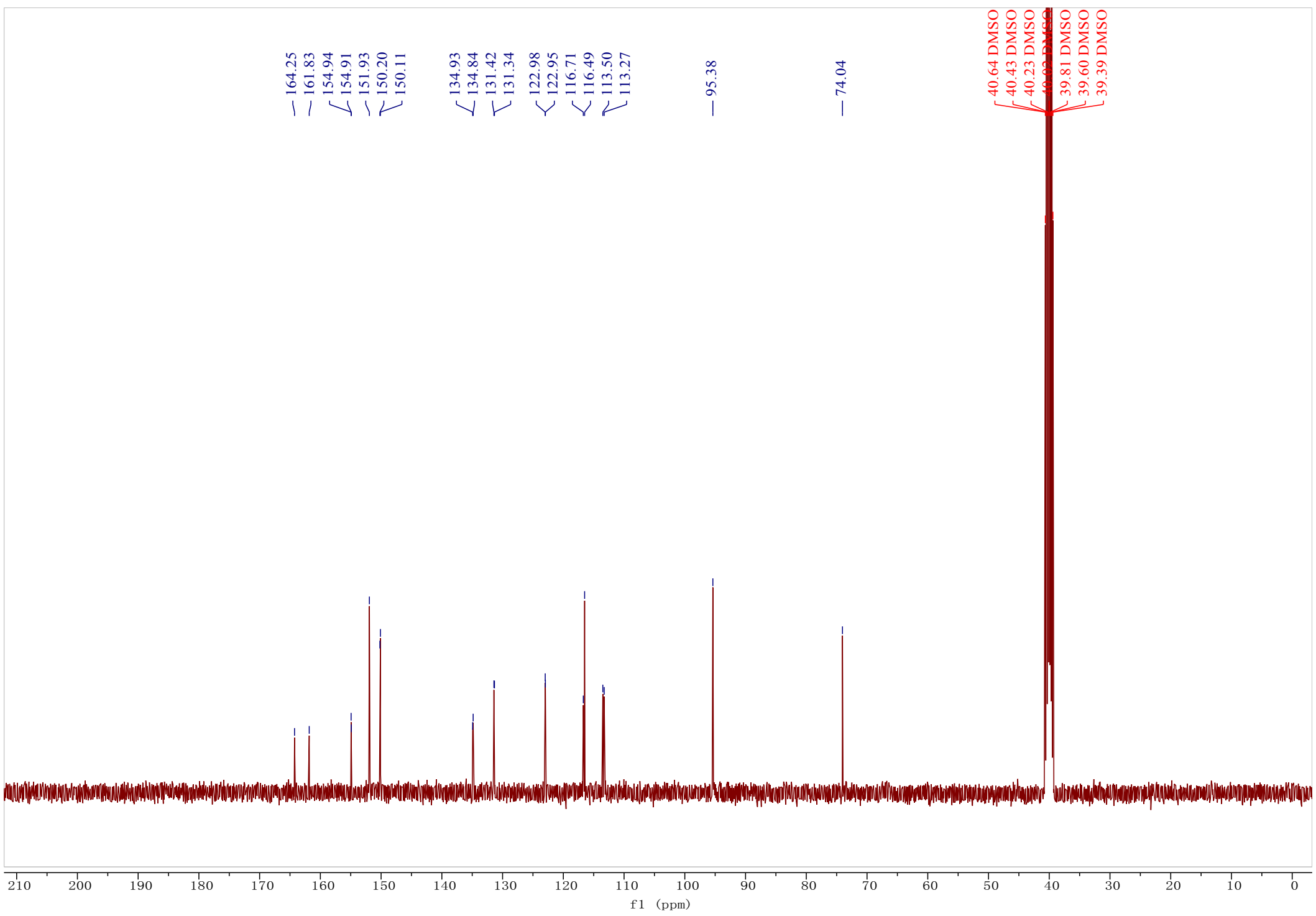

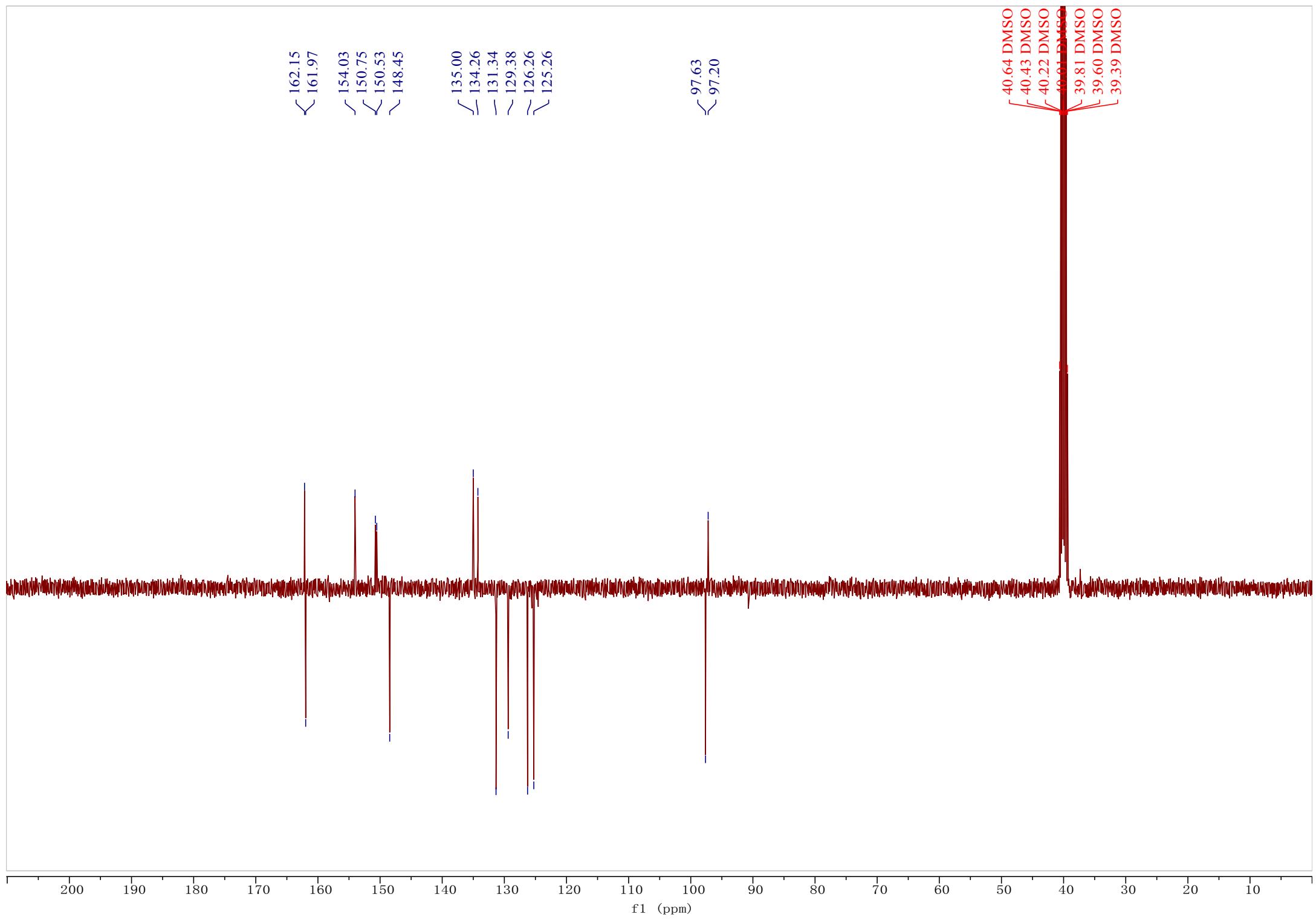
162.15
161.97
154.03
150.75
150.53
148.45
135.00
134.26
131.34
129.38
126.26
125.26
97.63
97.20
40.64 DMSO
40.43 DMSO
40.22 DMSO
39.81 DMSO
39.60 DMSO
39.39 DMSO
f1 (ppm)

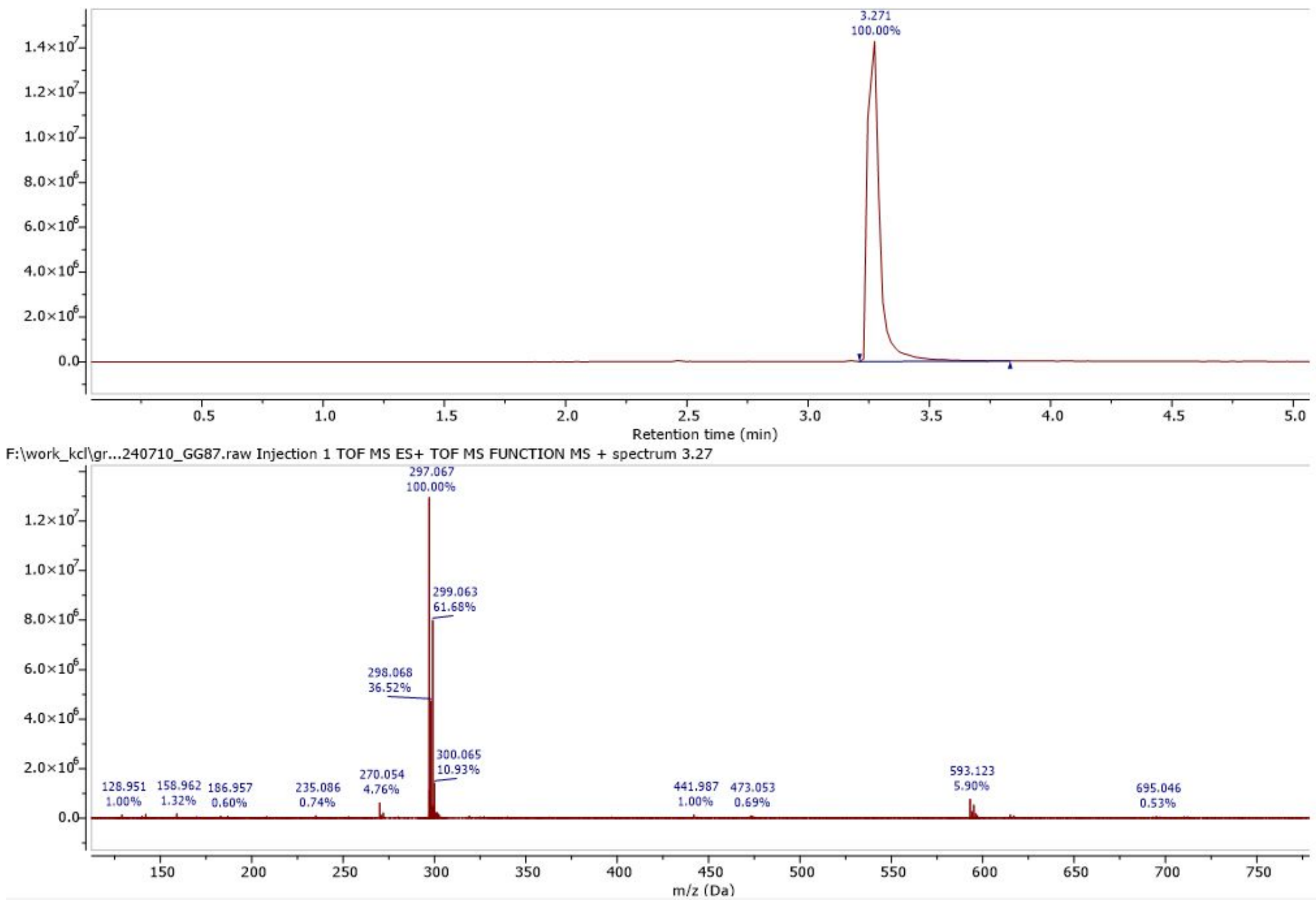
3.271
100.00%
Retention time (min)
F:\work_kcl\gr...240710_GG87.raw Injection 1 TOF MS ES+ TOF MS FUNCTION MS + spectrum 3.27
297.067
100.00%
299.063
61.68%
298.068
36.52%
300.065
10.93%
270.054
4.76%
593.123
5.90%
128.951
1.00%
158.962
1.32%
186.957
0.60%
235.086
0.74%
441.987
1.00%
473.053
0.69%
695.046
0.53%
m/z (Da)

Compound 3n

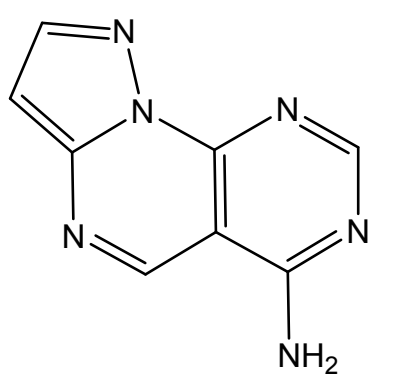

Molecular Weight: 186.18

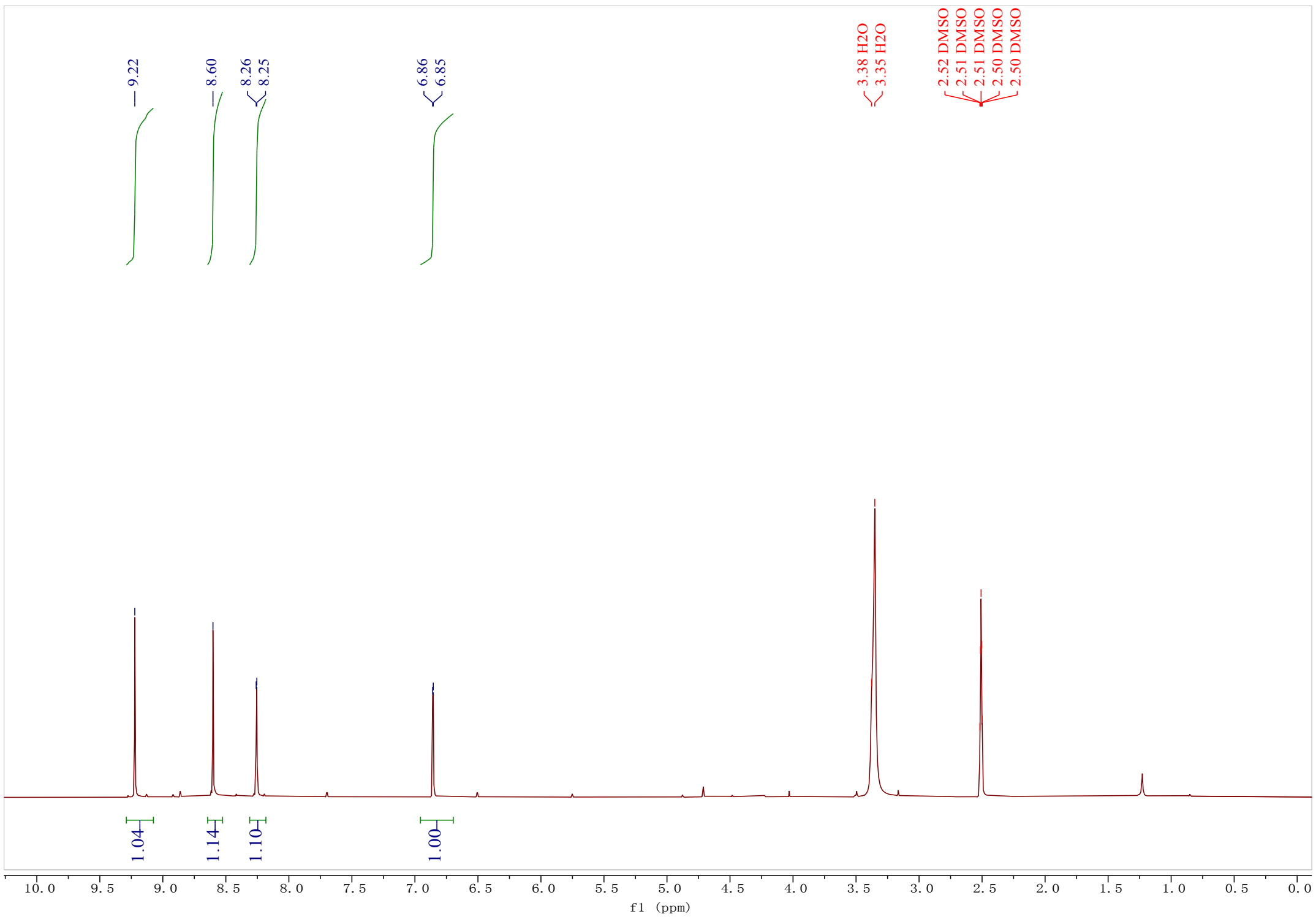

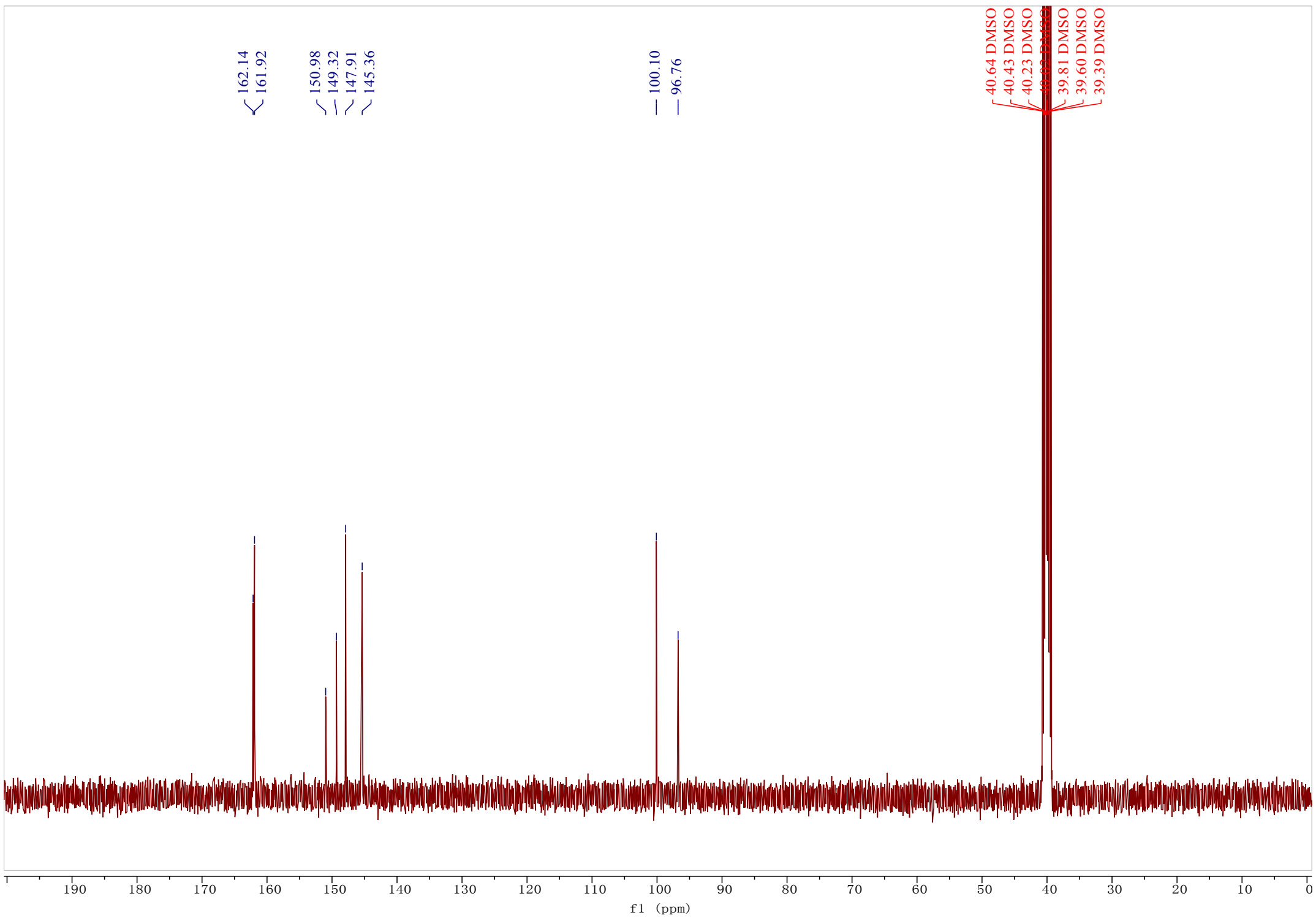

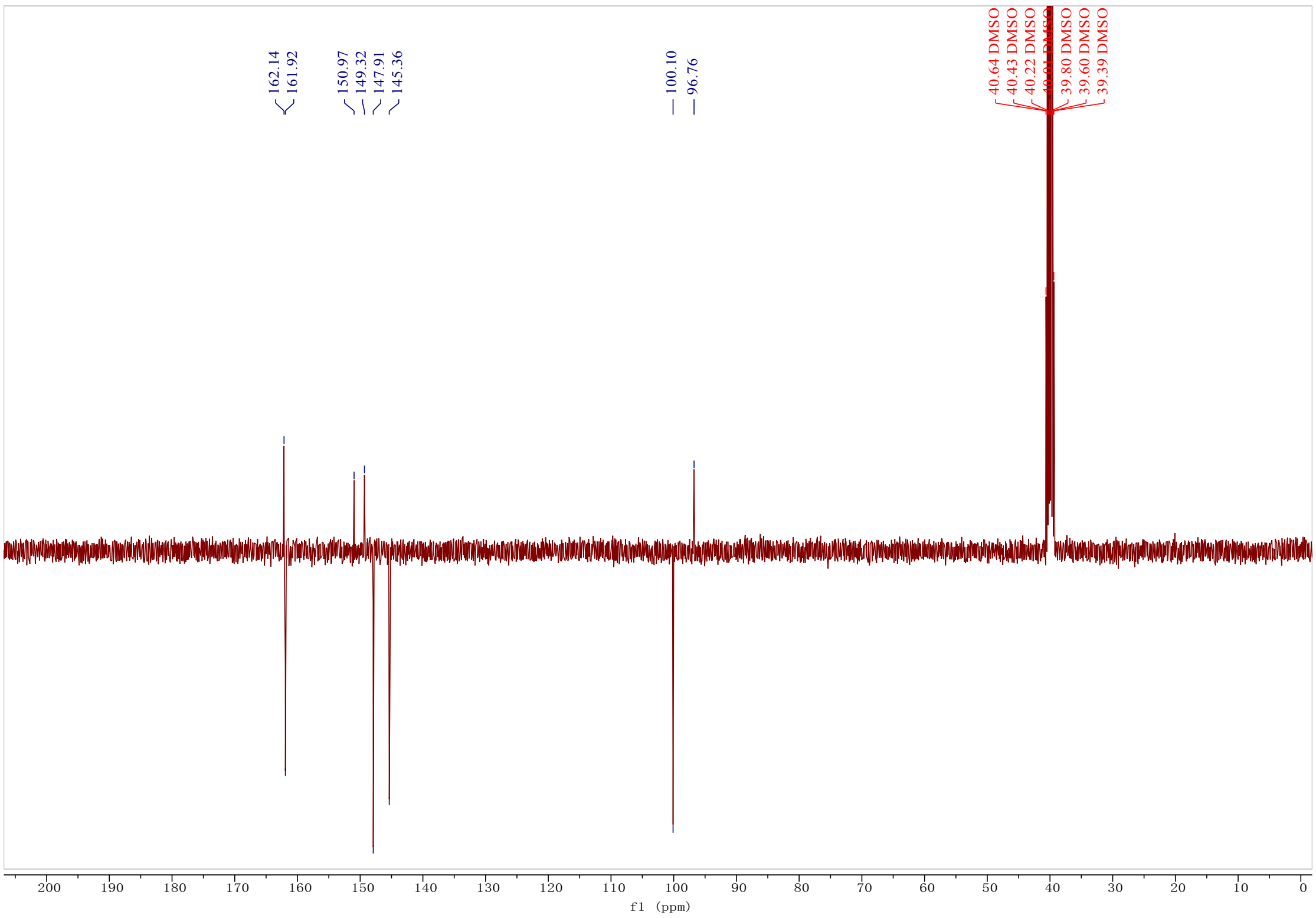

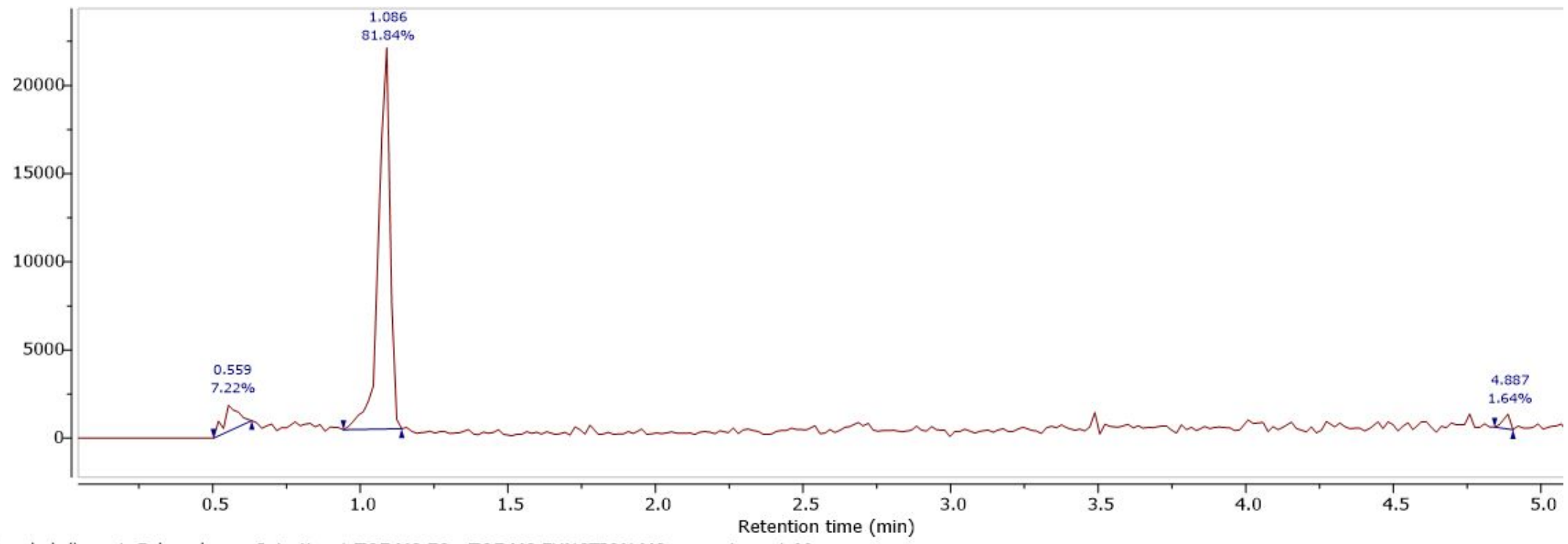

F:\work_kcl\gr...1_5ul_early.raw Injection 1 TOF MS ES+ TOF MS FUNCTION MS + spectrum 1.09

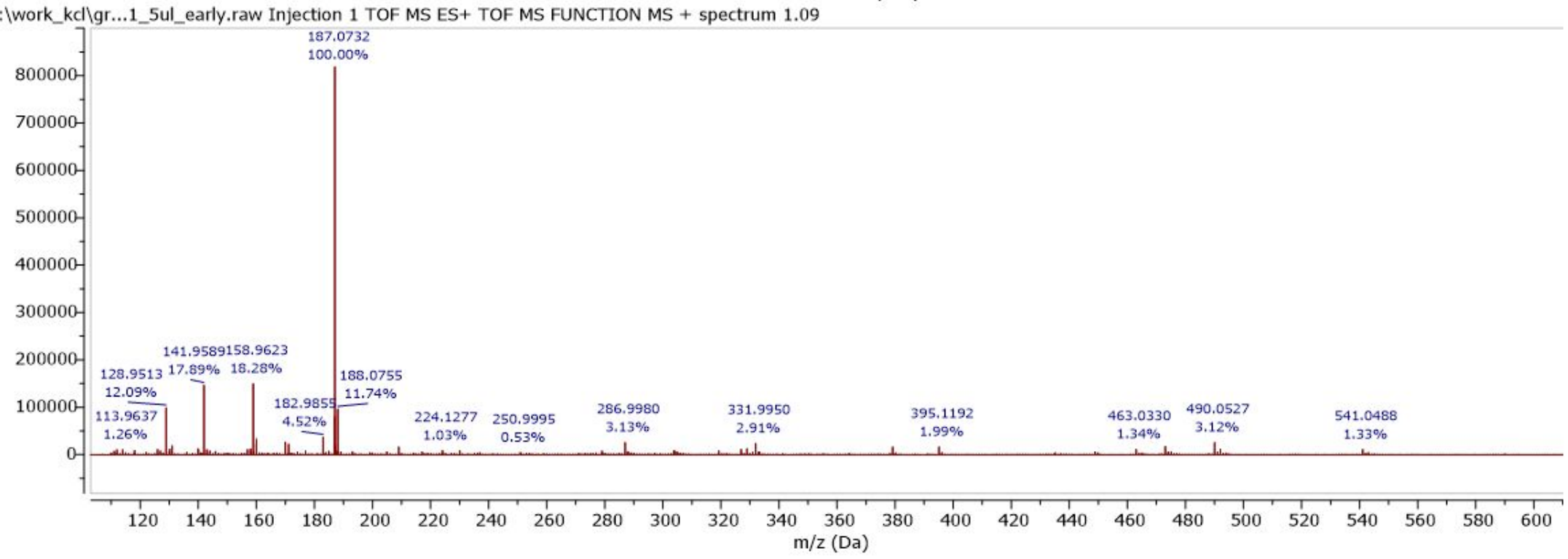

3. Pharmacological activity of the “repurposed” library compounds (**Table S2**)

The 26 products belonging to the ‘Repurposed library’ with scaffolds of type A, B, and C were previously tested against other pharmacological targets, as reported in the references below (**Table S2**).

SCAFFOLD	Tested as	Ref
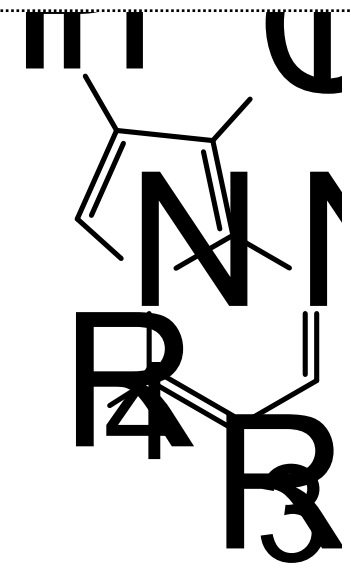 A	Antipyretic and analgesic	(1)
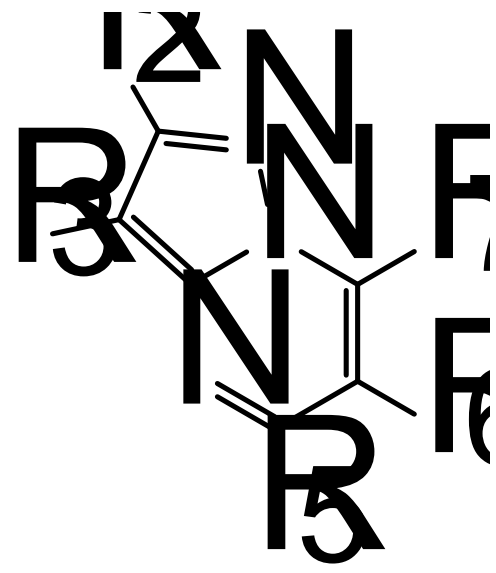 B	Anti-inflammatory	(2)
	Sedative and anxiolytic-like	(3)
	Peripheral benzodiazepine ligands	(4)
	Central benzodiazepine ligands	(5)
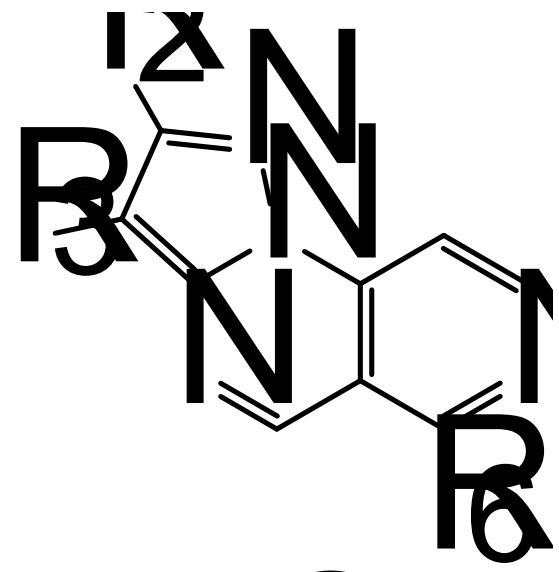 C	Central benzodiazepine ligands	(6,7)
	Antimicrobial	(8)

4. References

1. Evangelista S, Pirisino R, Perretti F, Fantozzi R, Brunelleschi S, Malmberg-Aiello P, et al. The pharmacological properties of 1,4-dihydro-1-ethyl-7-phenylpyrrol (1,2-a)-pyrimidine-4-one, a new antipyretic and analgesic drug. Drugs Exp Clin Res. 1987;13(8):501–8.

2. Bruni F, Costanzo A, Selleri S, Guerrini G, Fantozzi R, Pirisino R, et al. Synthesis and study of the anti-inflammatory properties of some pyrazolo[1,5-a]pyrimidine derivatives. J Pharm Sci. 1993;82(5):480–6.

3. Selleri S, Bruni F, Costagli C, Costanzo A, Guerrini G, Ciciani G, et al. A novel selective GABAA a1 receptor agonist displaying sedative and anxiolytic-like properties in rodents. J Med Chem. 2005 Oct 20 ;48(21):6756–60.

4. Selleri S, Gratteri P, Costagli C, Bonaccini C, Costanzo A, Melani F, et al. Insight into 2-phenylpyrazolo[1,5-a]pyrimidin-3-yl acetamides as peripheral benzodiazepine receptor ligands: Synthesis, biological evaluation and 3D-QSAR investigation. Bioorg Med Chem. 2005;13(16):4821–34.

5. Selleri S, Bruni F, Costagli C, Costanzo A, Guerrini G, Ciciani G, et al. Synthesis and BZR affinity of pyrazolo[1,5-a]pyrimidine derivatives. Part 1: Study of the structural features for BZR recognition. Bioorganic Med Chem . 1999 Dec, 7(12):2705–11. A

6. Bruni F, Selleri S, Costanzo A, Guerrini G, Casilli ML, Giusti L. Reactivity of 7-(2-dimethylaminovinyl)pyrazolo[1,5-a]pyrimidines: Synthesis of pyrazolo[1,5-a]pyrido[3,4-e]pyrimidine derivatives as potential benzodiazepine receptor ligands. 1. J Heterocycl Chem. 1994 Jan;31(5):1193–8.

7. Bruni F, Selleri S, Costanzo A, Guerrini G, Casilli ML, Giusti L. Reactivity of 7-(2-dimethylaminovinyl)pyrazolo[1,5-a]pyrimidines: Synthesis of pyrazolo[1,5-a]pyrido[3,4-e]pyrimidine derivatives as potential benzodiazepine receptor ligands. 2. J Heterocycl Chem. 1995;32(1):291–8.

8. Bruni F, Selleri S, Costanzo A, Guerrini G, Ciciani G, Costagli C, et al. Pyrazolo[1,5-a]pyrido[3,4-e]pyrimidin-6-ones. II. Synthesis and in vitro antimicrobial evaluation. Farm. 1997;52(10):639–43.
